# Supplementary figures and images for: FABP4 as a therapeutic host target controlling SARS-CoV-2 infection (part 1 of 2)
Source: EMBO Mol Med. 2025 Jan 22;17(3):414–40. doi: 10.1038/s44321-024-00188-x (PMC11904229; doi:10.1038/s44321-024-00188-x)

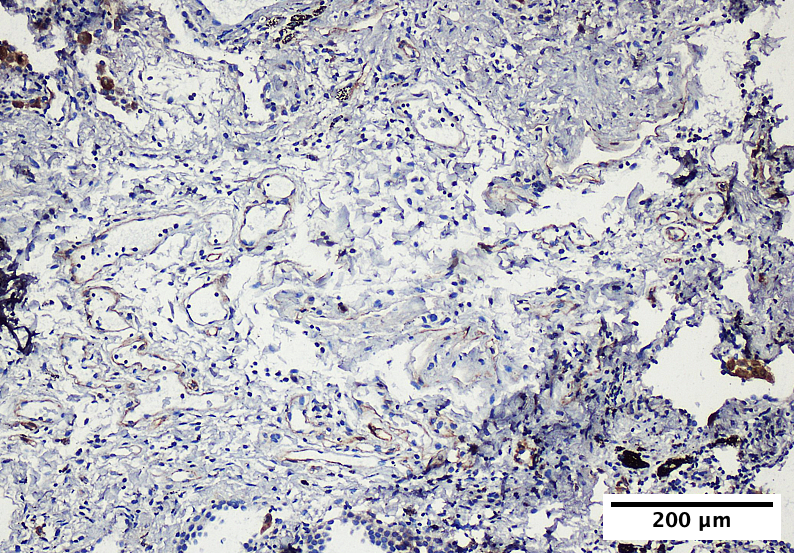

Supplement: Supplementary file 6 — Source data Fig. 1 [file 44321_2024_188_MOESM6_ESM.zip › Figure 1/1A/PATIENT 3_low magnification.png]

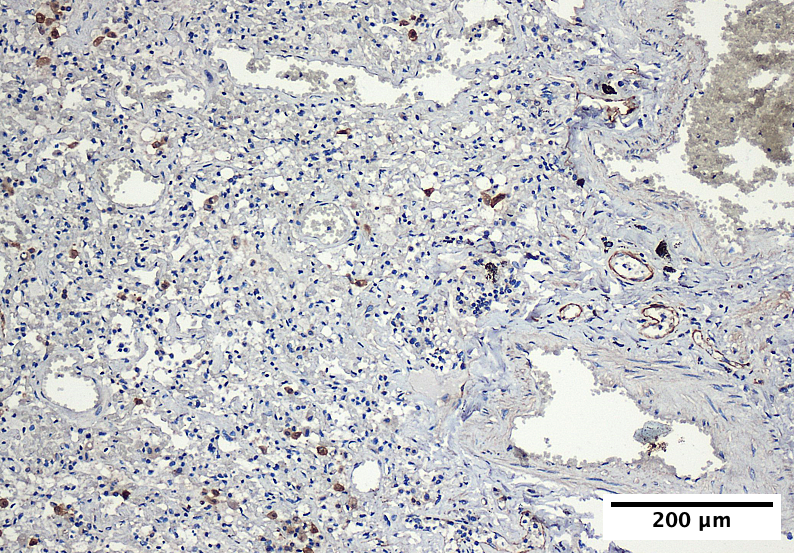

Supplement: Supplementary file 6 — Source data Fig. 1 [file 44321_2024_188_MOESM6_ESM.zip › Figure 1/1A/PATIENT 2_low magnification.png]

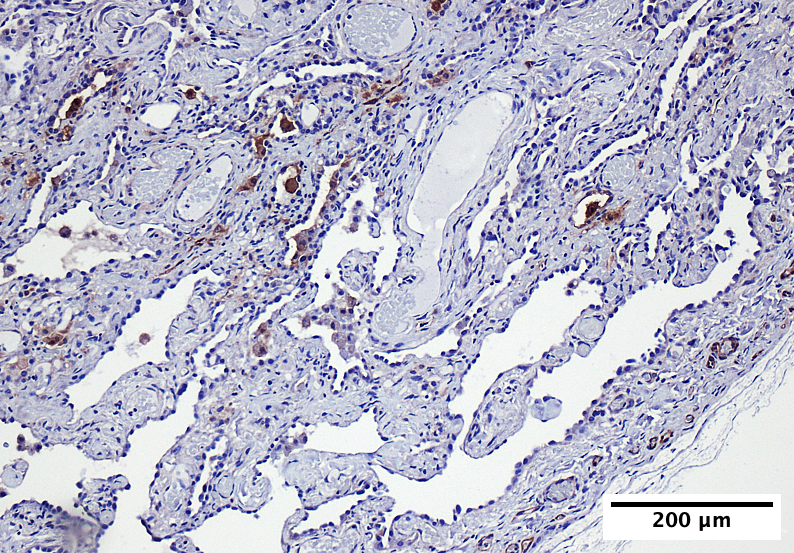

Supplement: Supplementary file 6 — Source data Fig. 1 [file 44321_2024_188_MOESM6_ESM.zip › Figure 1/1A/PATIENT 1_low magnification.png]

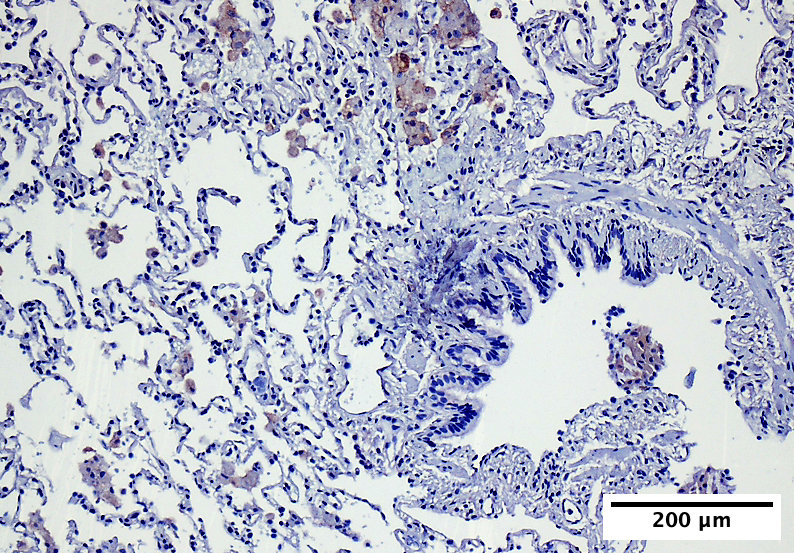

Supplement: Supplementary file 6 — Source data Fig. 1 [file 44321_2024_188_MOESM6_ESM.zip › Figure 1/1A/CONTROL_low magnification.png]

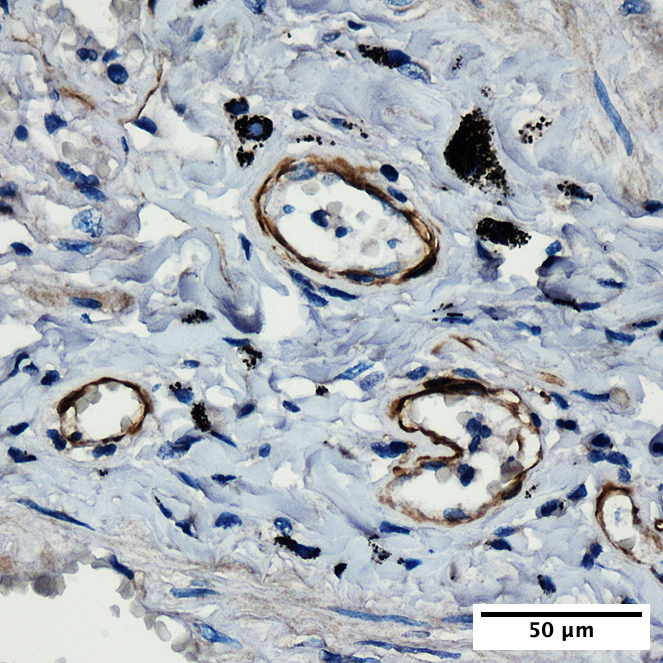

Supplement: Supplementary file 6 — Source data Fig. 1 [file 44321_2024_188_MOESM6_ESM.zip › Figure 1/1A/PATIENT 2_high magnification.png]

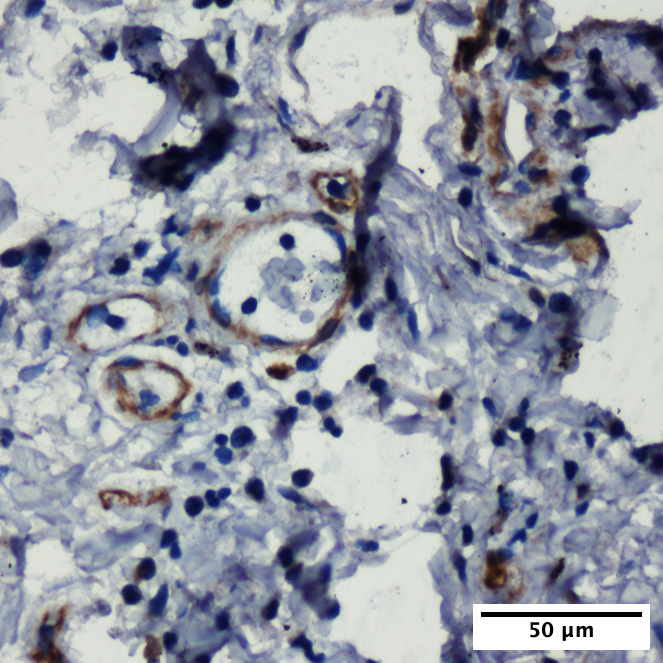

Supplement: Supplementary file 6 — Source data Fig. 1 [file 44321_2024_188_MOESM6_ESM.zip › Figure 1/1A/PATIENT 3_high magnification.png]

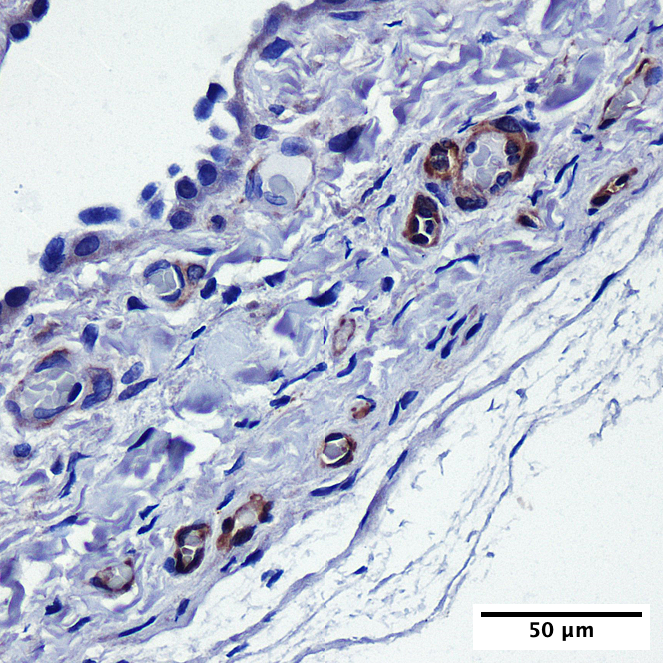

Supplement: Supplementary file 6 — Source data Fig. 1 [file 44321_2024_188_MOESM6_ESM.zip › Figure 1/1A/PATIENT 1_high magnification.png]

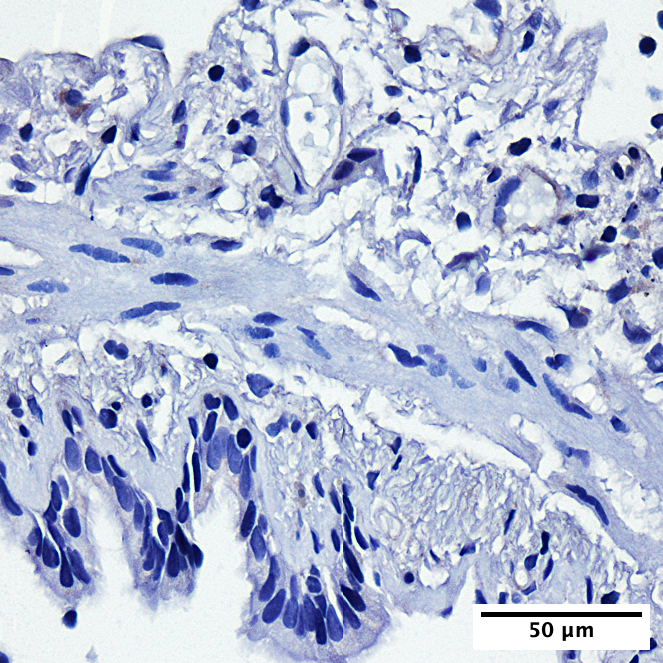

Supplement: Supplementary file 6 — Source data Fig. 1 [file 44321_2024_188_MOESM6_ESM.zip › Figure 1/1A/CONTROL_high magnification.png]

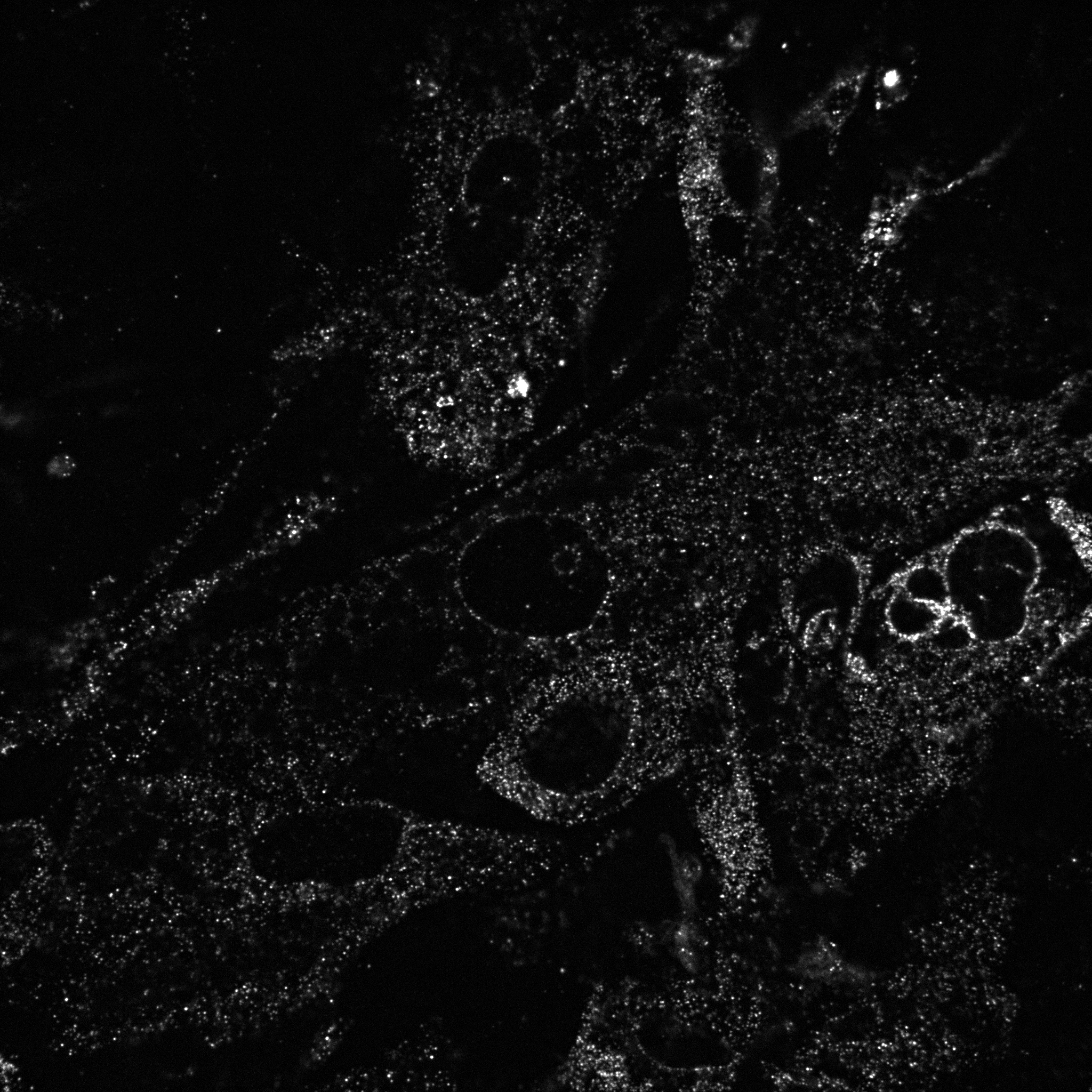

Supplement: Supplementary file 7 — Source data Fig. 2 [file 44321_2024_188_MOESM7_ESM.zip › Figure 2/2L-P/uninfected.tif]

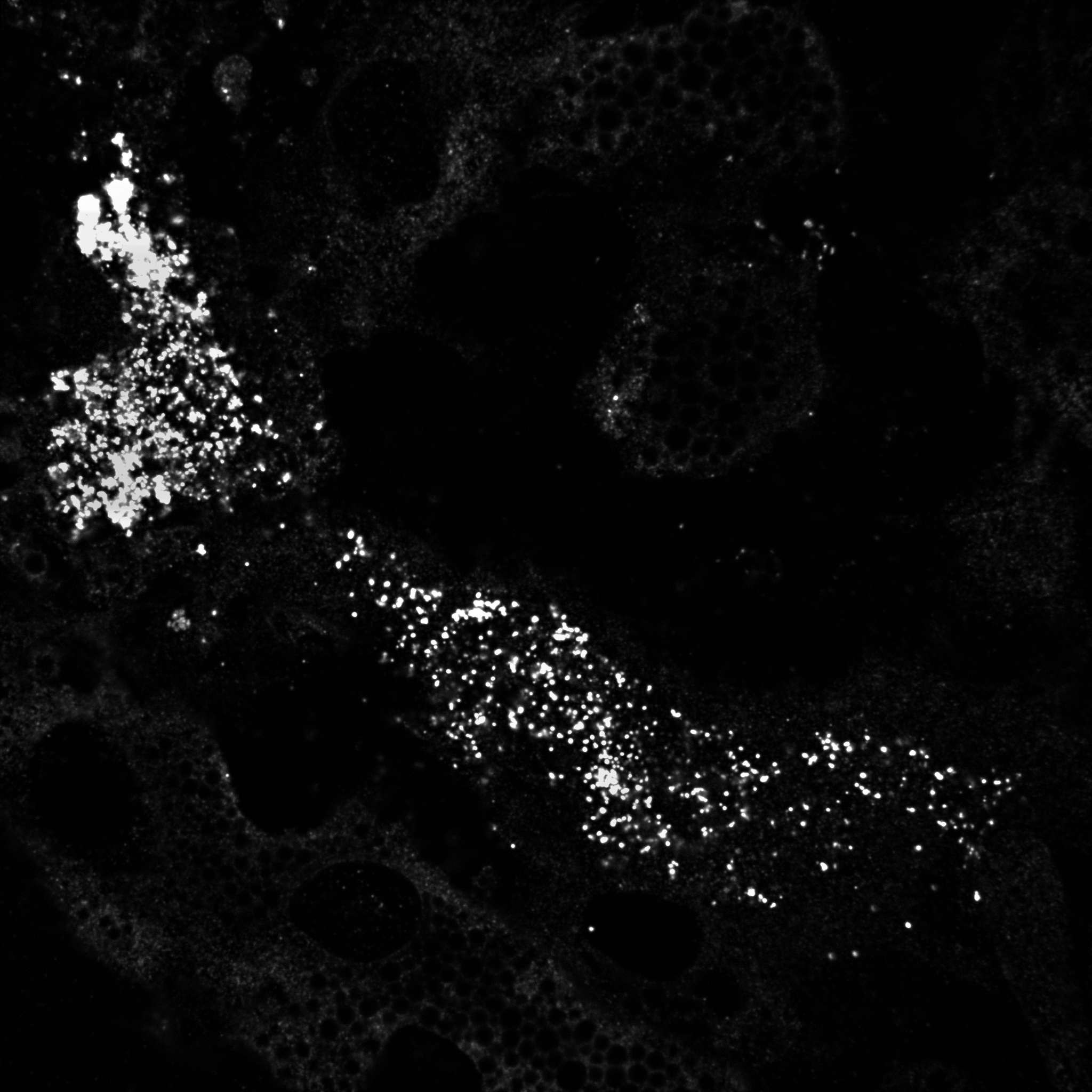

Supplement: Supplementary file 7 — Source data Fig. 2 [file 44321_2024_188_MOESM7_ESM.zip › Figure 2/2L-P/Infected_48hpi.tif]

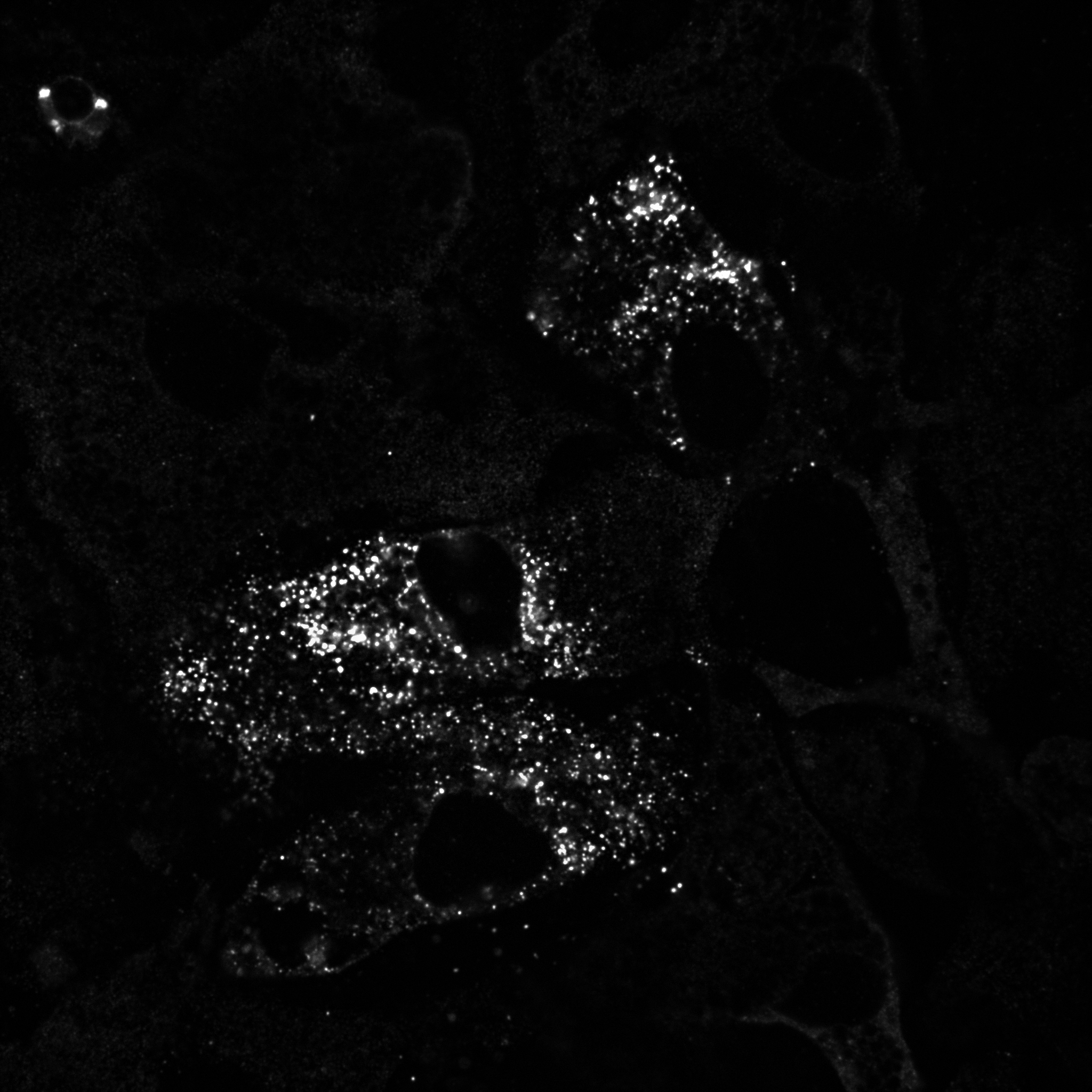

Supplement: Supplementary file 7 — Source data Fig. 2 [file 44321_2024_188_MOESM7_ESM.zip › Figure 2/2L-P/Infected_24hpi.tif]

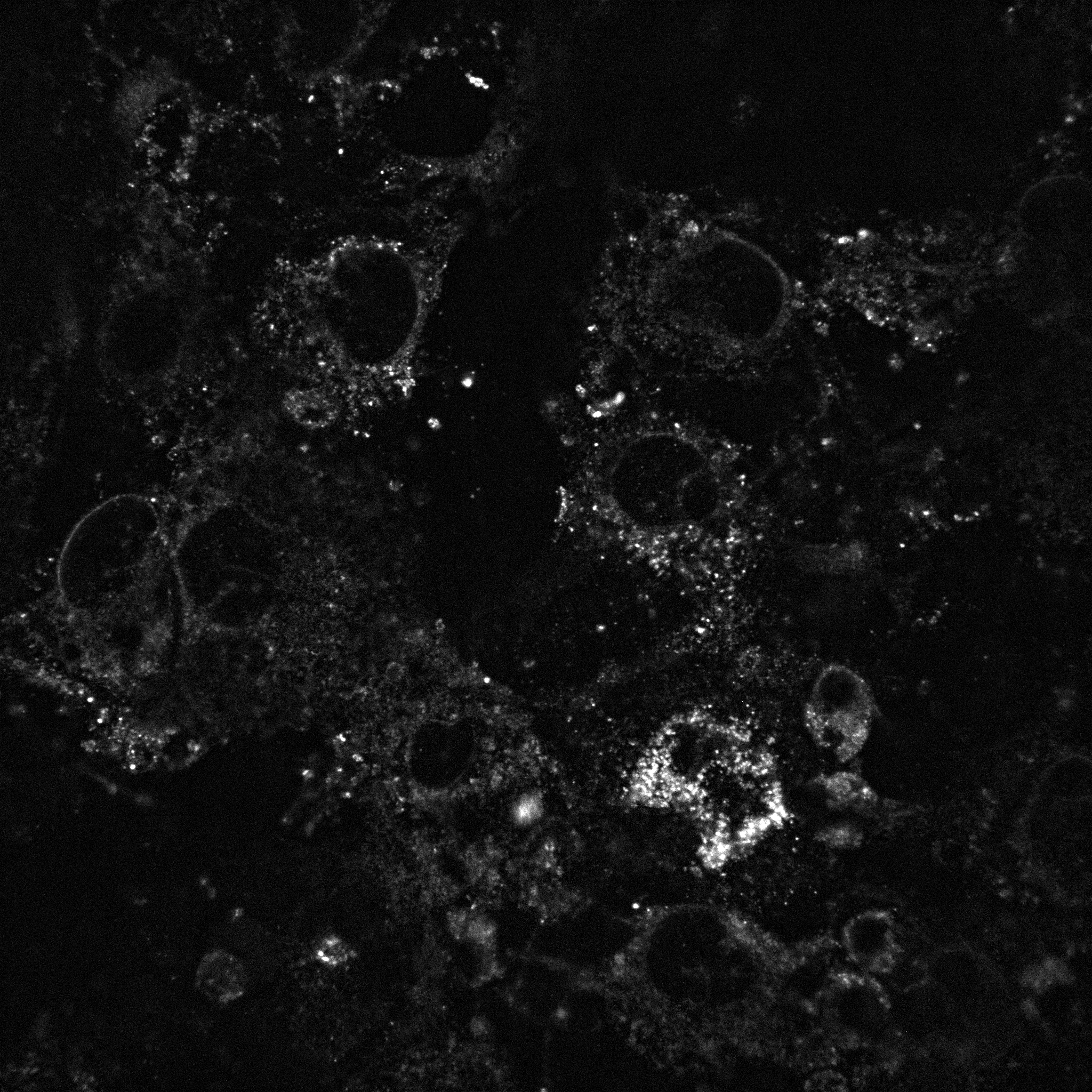

Supplement: Supplementary file 7 — Source data Fig. 2 [file 44321_2024_188_MOESM7_ESM.zip › Figure 2/2L-P/Infected_12hpi.tif]

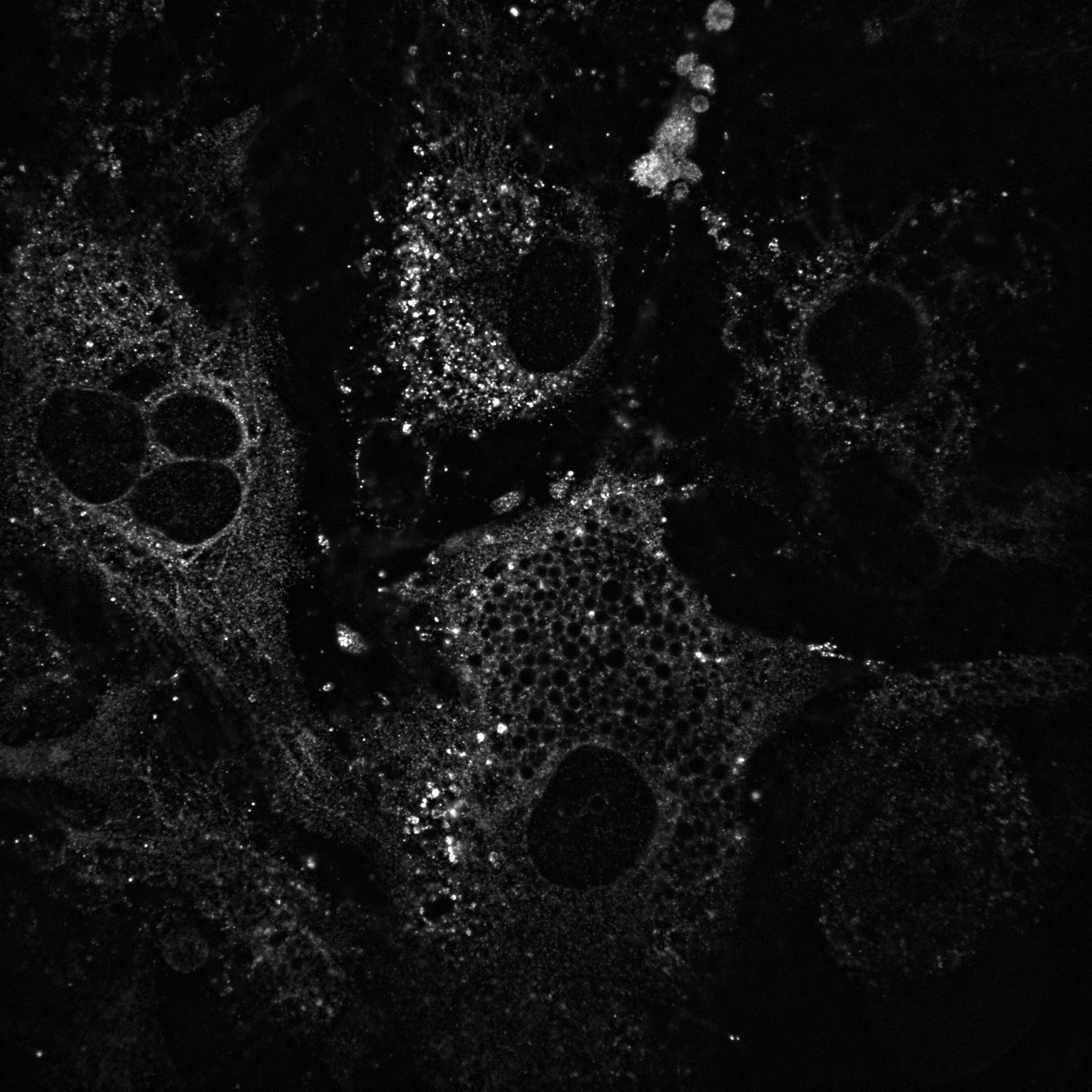

Supplement: Supplementary file 7 — Source data Fig. 2 [file 44321_2024_188_MOESM7_ESM.zip › Figure 2/2L-P/Infected_8hpi.tif]

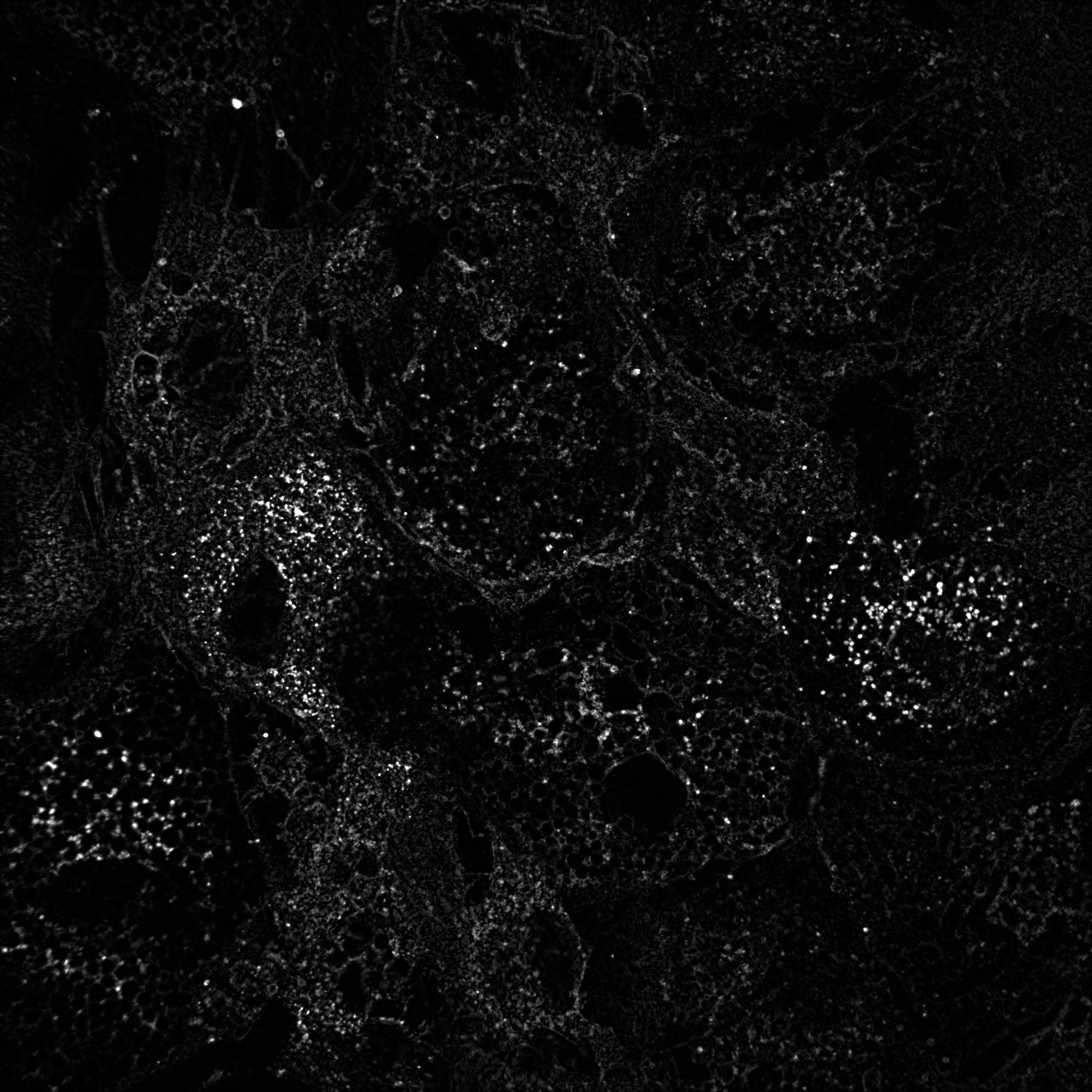

Supplement: Supplementary file 7 — Source data Fig. 2 [file 44321_2024_188_MOESM7_ESM.zip › Figure 2/2I-K/dsRNA.tif]

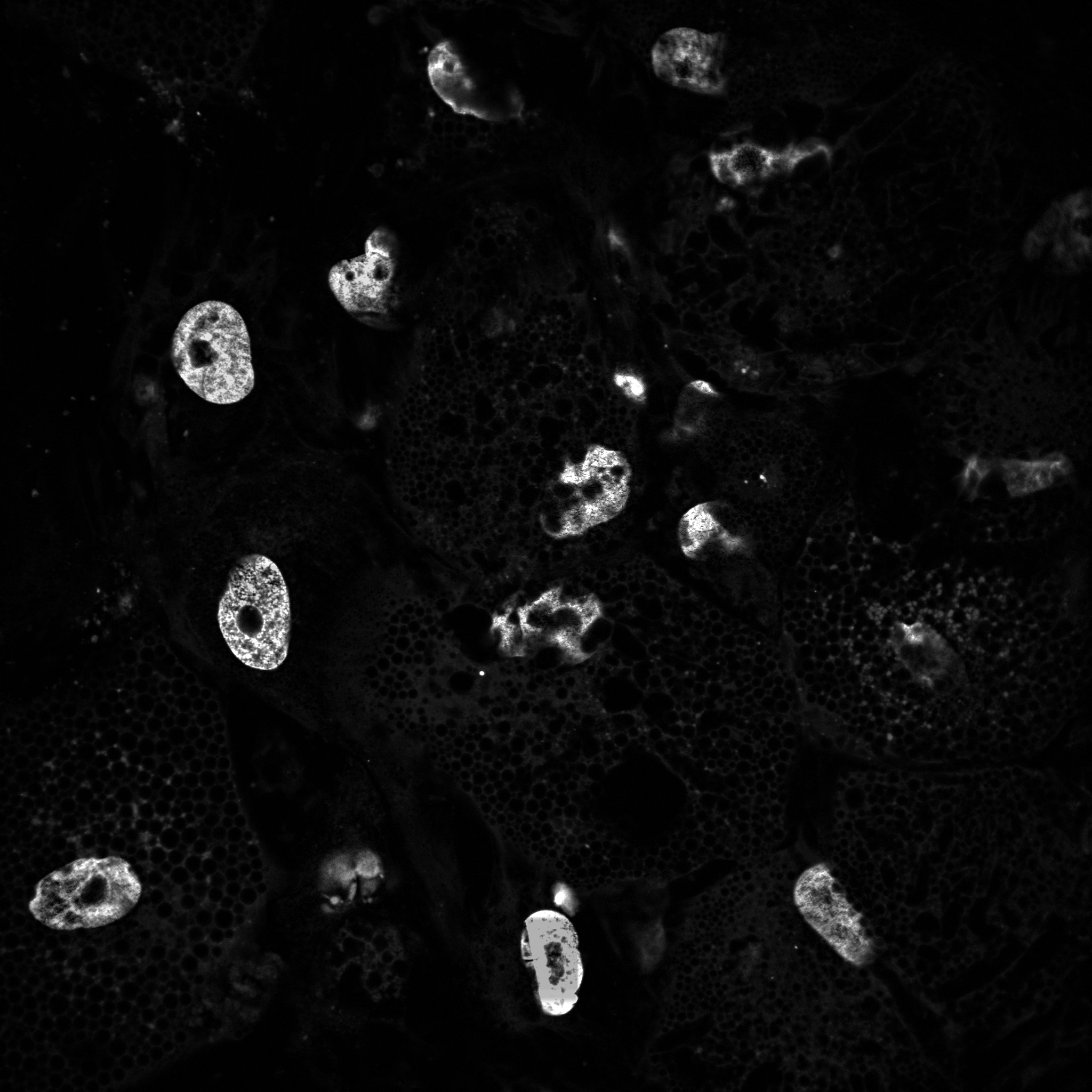

Supplement: Supplementary file 7 — Source data Fig. 2 [file 44321_2024_188_MOESM7_ESM.zip › Figure 2/2I-K/DAPI.tif]

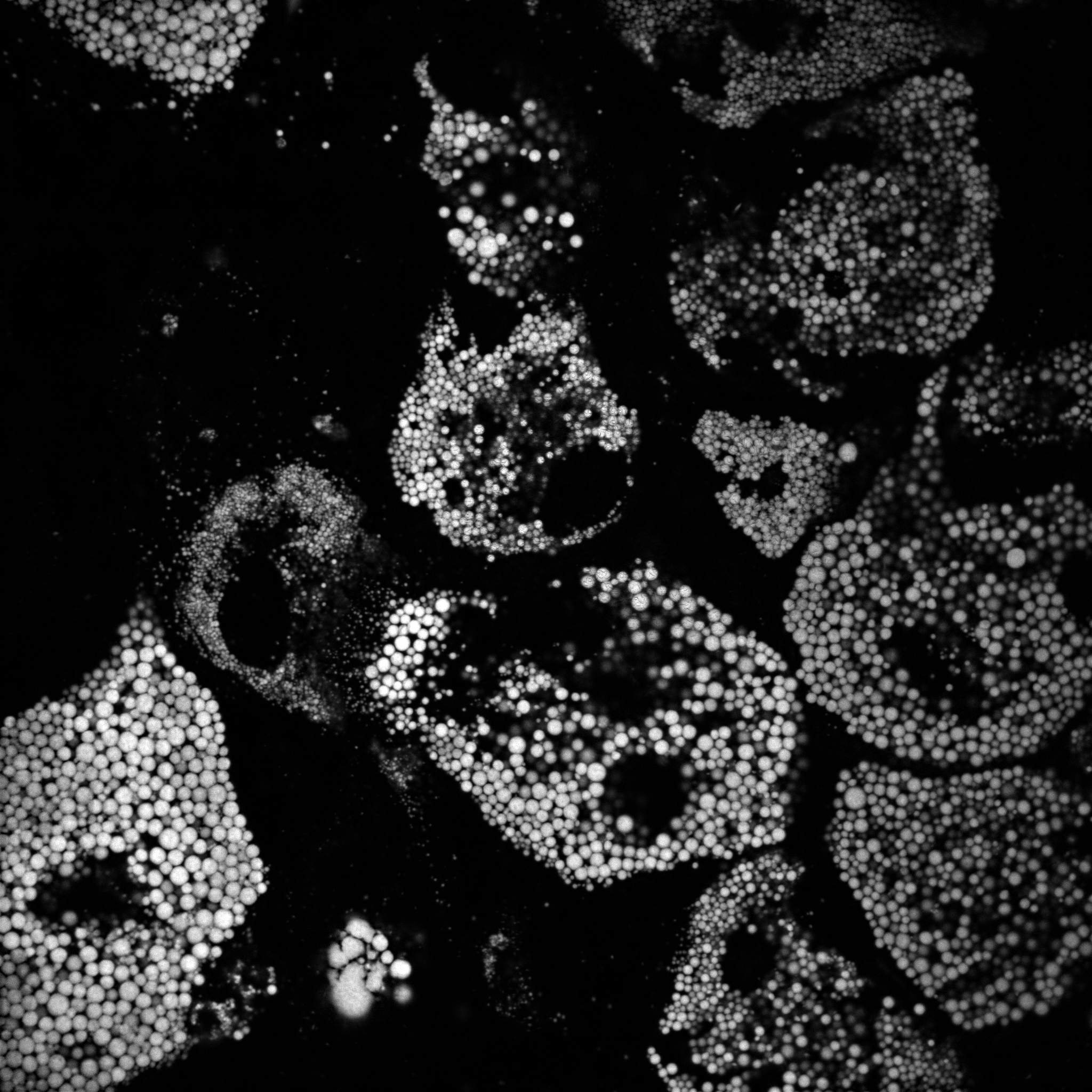

Supplement: Supplementary file 7 — Source data Fig. 2 [file 44321_2024_188_MOESM7_ESM.zip › Figure 2/2I-K/Lipid droplets.tif]

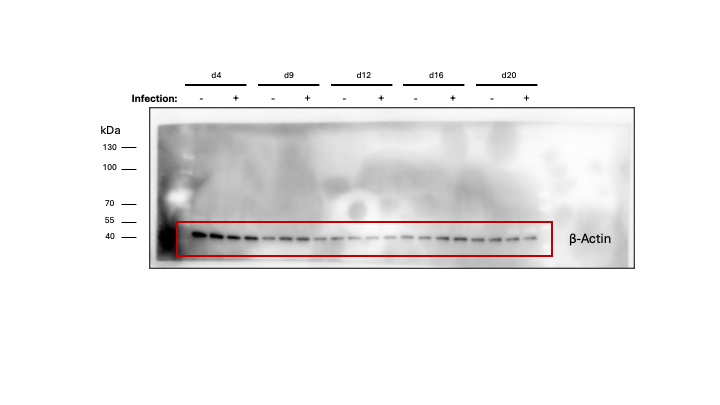

Supplement: Supplementary file 7 — Source data Fig. 2 [file 44321_2024_188_MOESM7_ESM.zip › Figure 2/2F-G/Actin_WB.tiff]

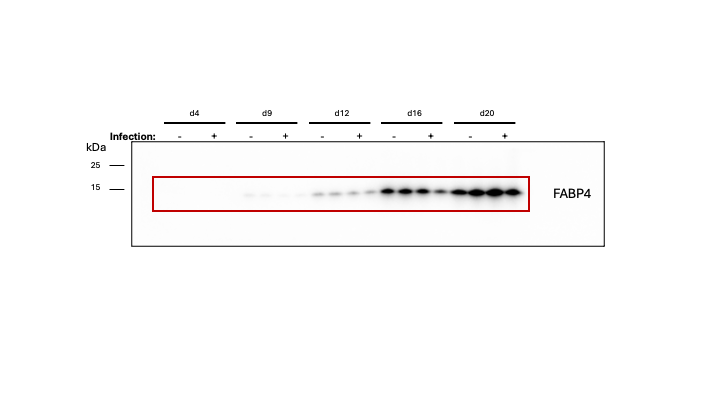

Supplement: Supplementary file 7 — Source data Fig. 2 [file 44321_2024_188_MOESM7_ESM.zip › Figure 2/2F-G/FABP4_WB.tiff]

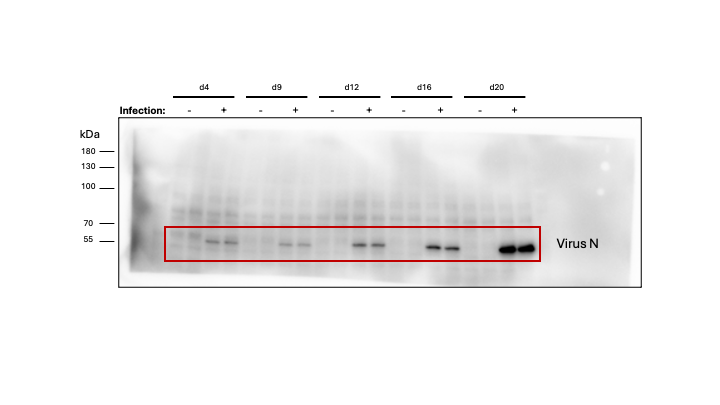

Supplement: Supplementary file 7 — Source data Fig. 2 [file 44321_2024_188_MOESM7_ESM.zip › Figure 2/2F-G/nucleocapsid_WB.tiff]

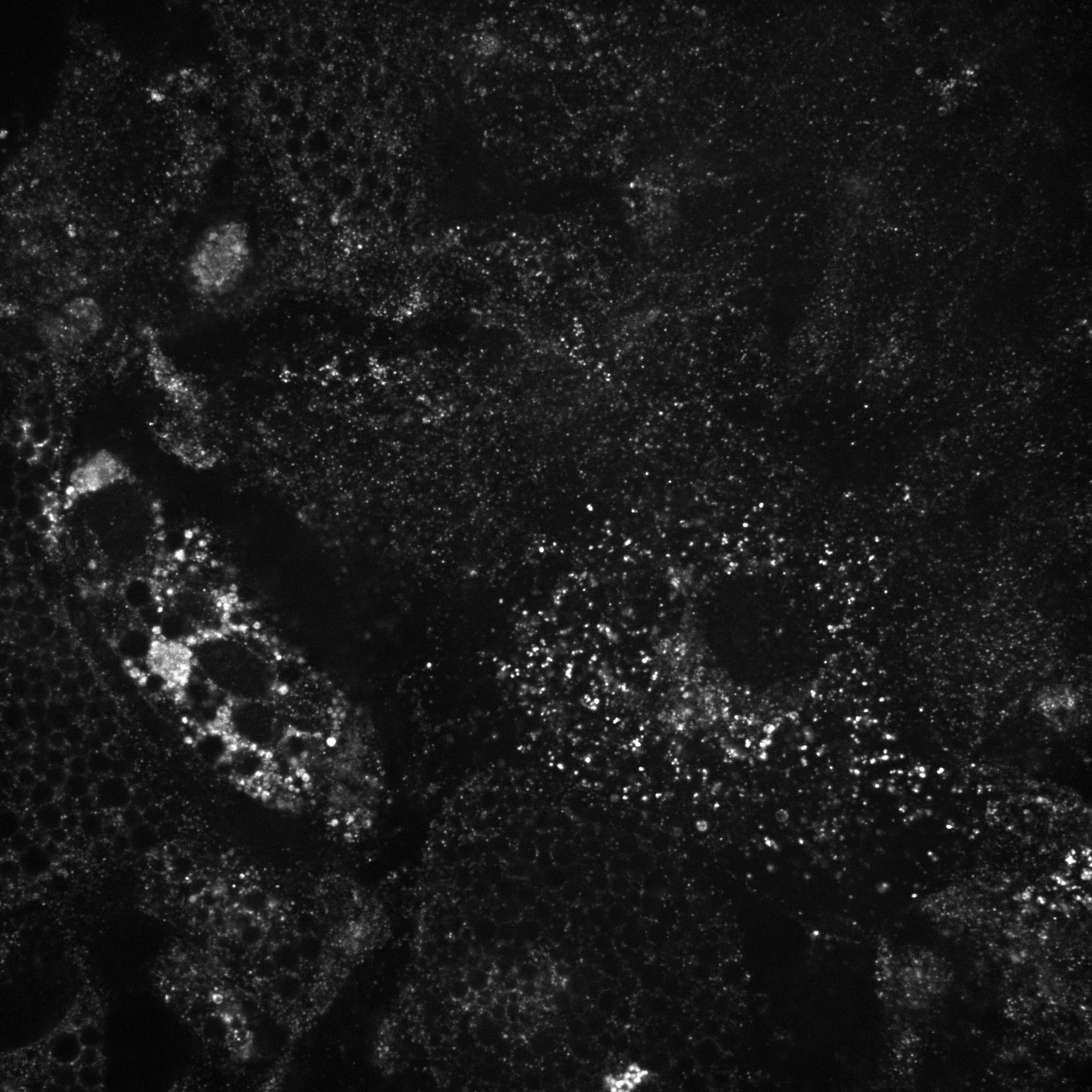

Supplement: Supplementary file 8 — Source data Fig. 3 [file 44321_2024_188_MOESM8_ESM.zip › Figure 3/3J-L/WT_calnexin.tif]

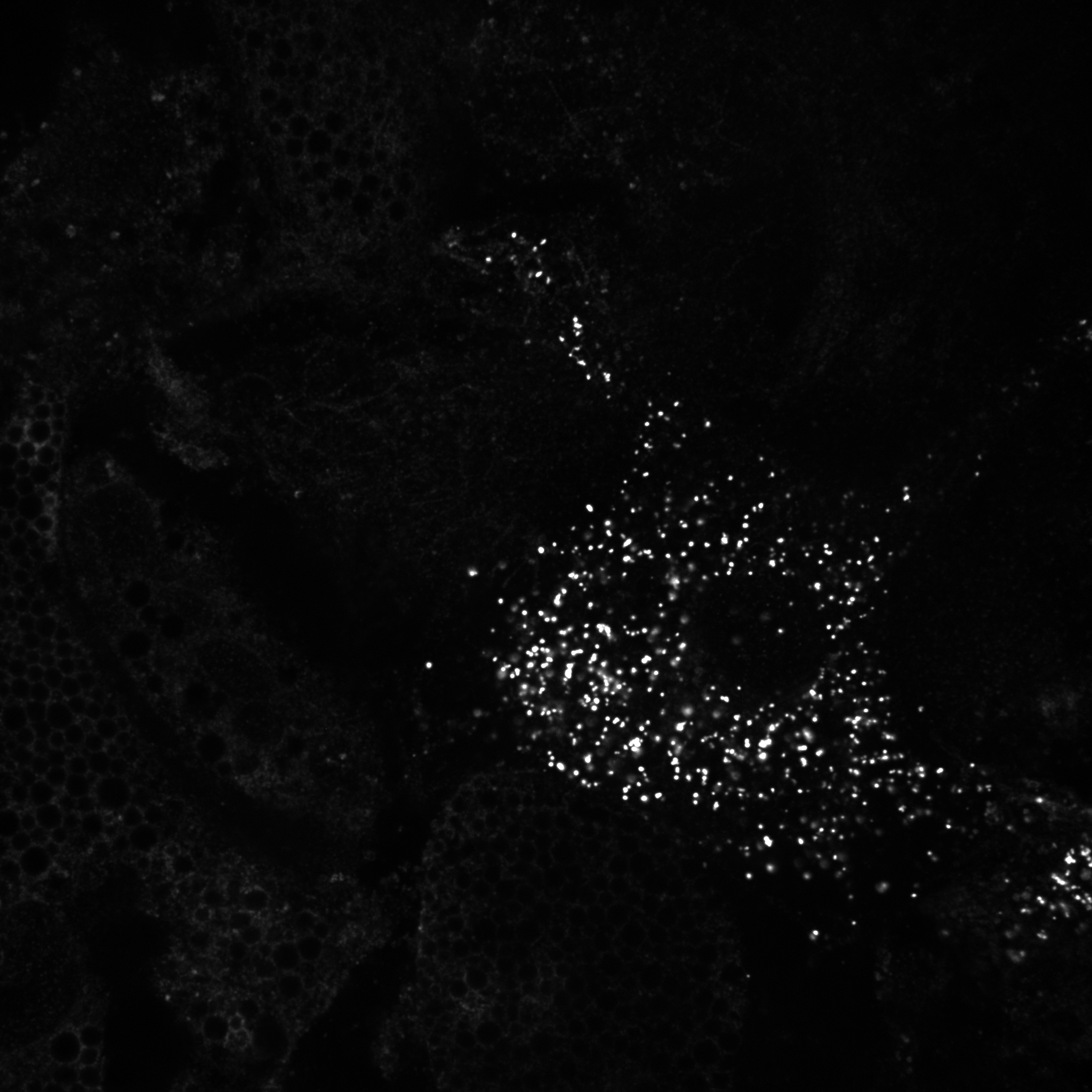

Supplement: Supplementary file 8 — Source data Fig. 3 [file 44321_2024_188_MOESM8_ESM.zip › Figure 3/3J-L/WT_dsRNA.tif]

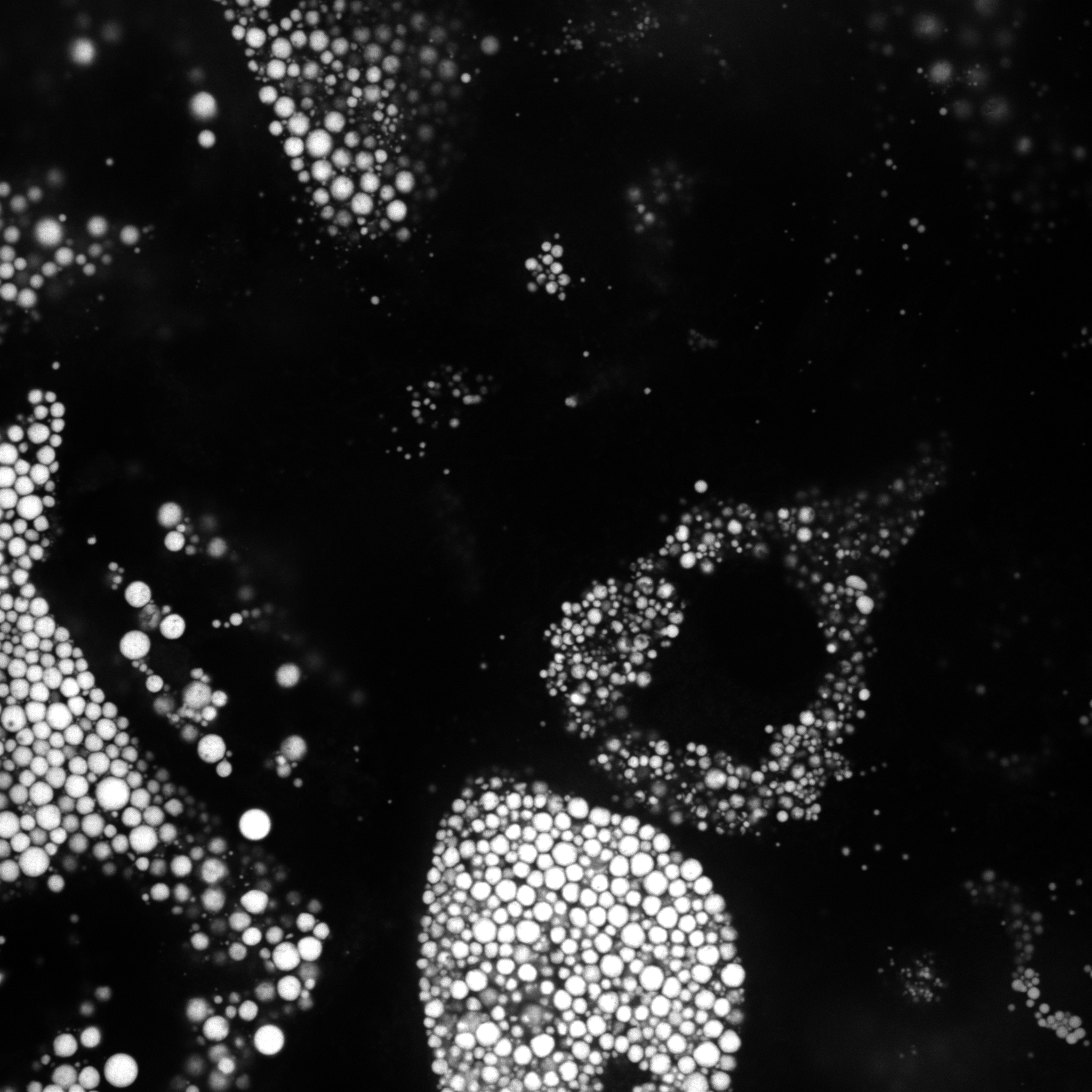

Supplement: Supplementary file 8 — Source data Fig. 3 [file 44321_2024_188_MOESM8_ESM.zip › Figure 3/3J-L/WT_LDs.tif]

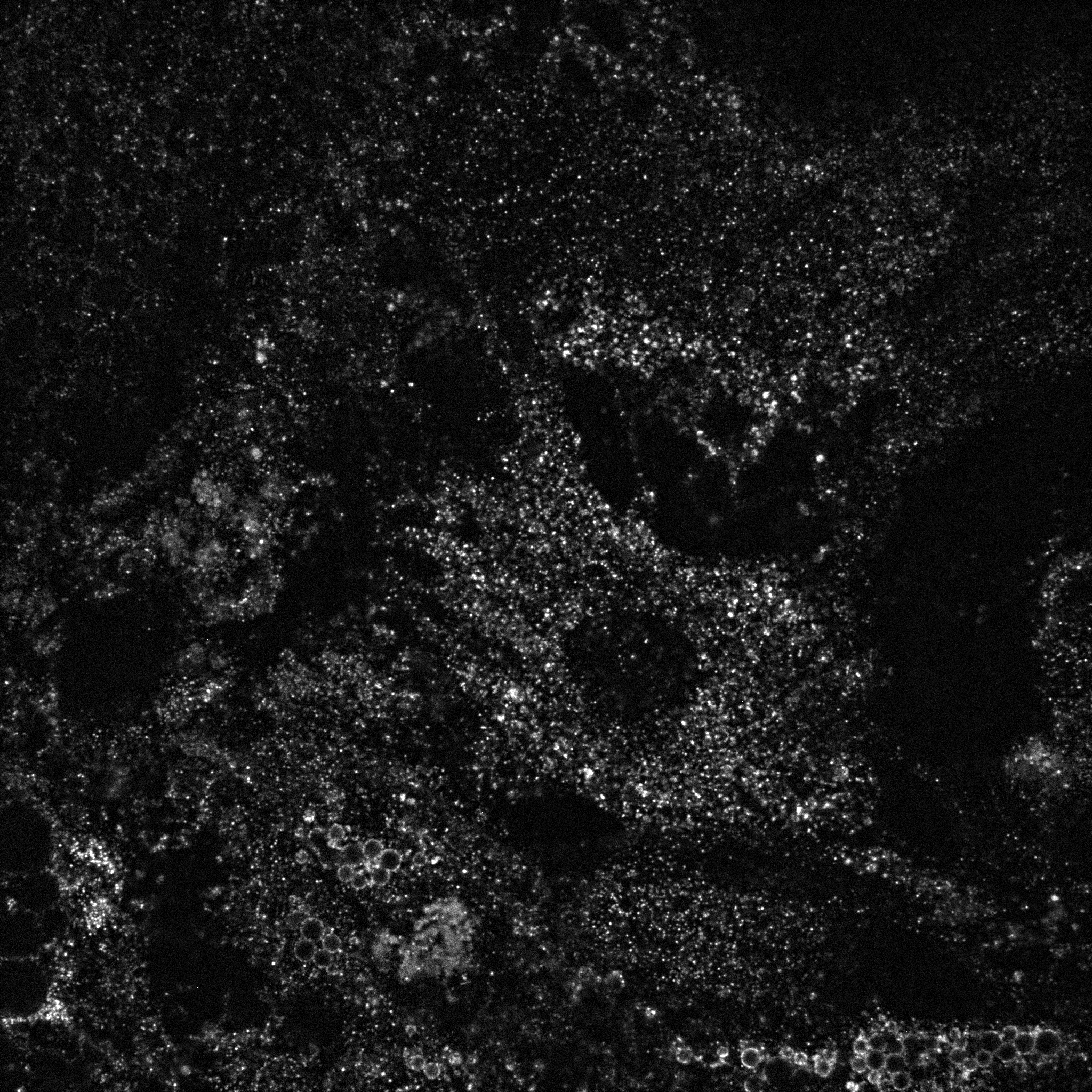

Supplement: Supplementary file 8 — Source data Fig. 3 [file 44321_2024_188_MOESM8_ESM.zip › Figure 3/3J-L/FABP4-KO_calnexin.tif]

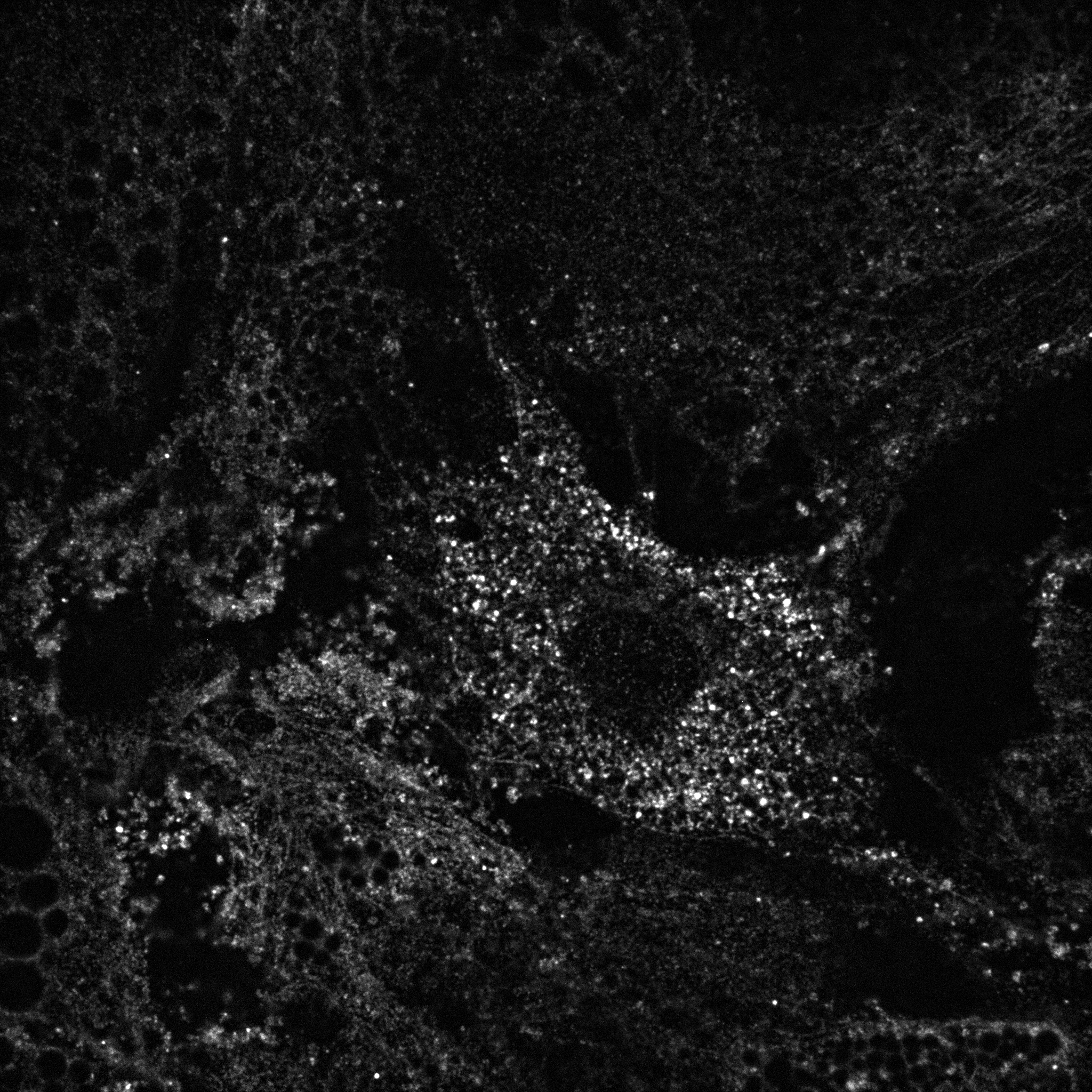

Supplement: Supplementary file 8 — Source data Fig. 3 [file 44321_2024_188_MOESM8_ESM.zip › Figure 3/3J-L/FABP4-KO_dsRNA.tif]

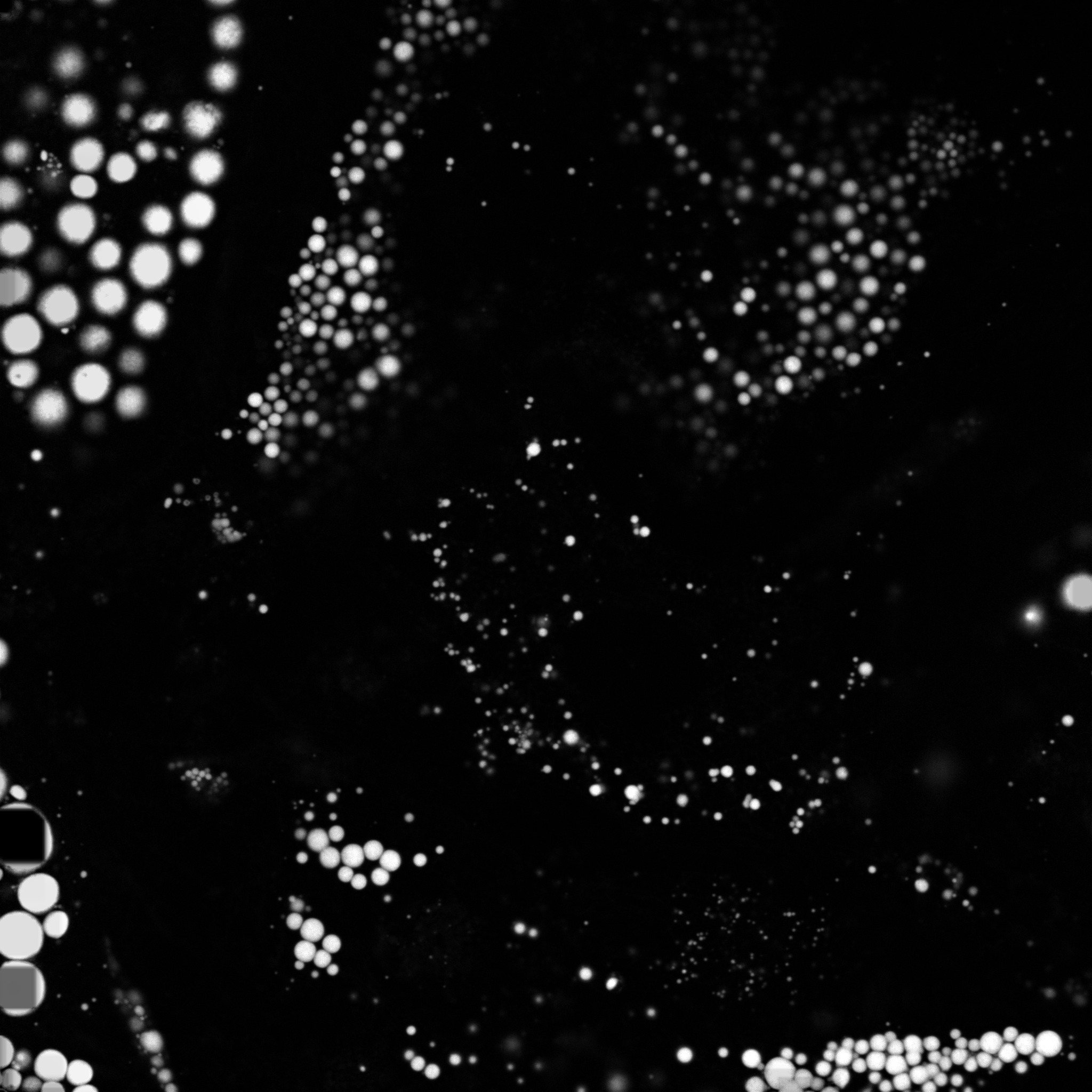

Supplement: Supplementary file 8 — Source data Fig. 3 [file 44321_2024_188_MOESM8_ESM.zip › Figure 3/3J-L/FABP4-KO_LDs.tif]

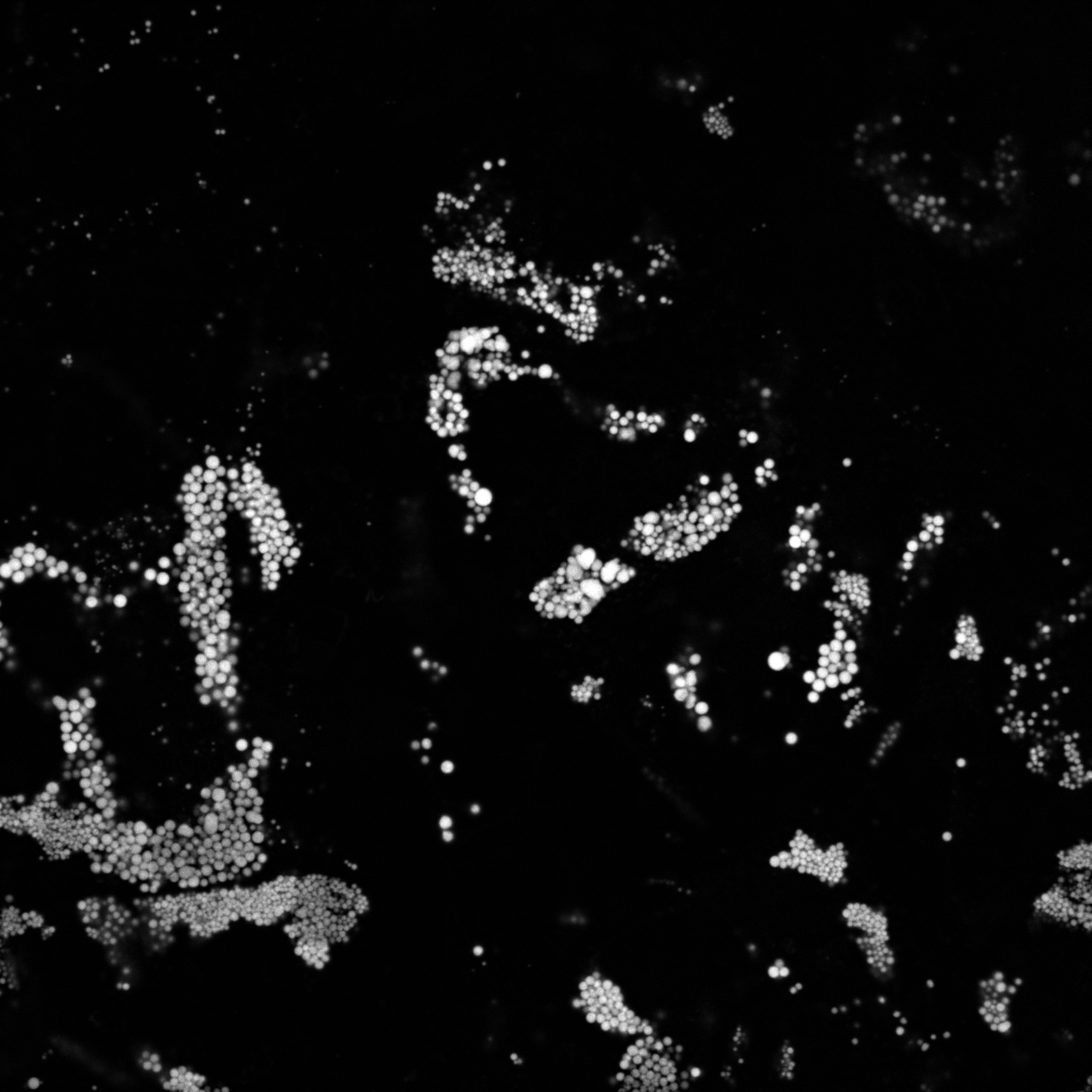

Supplement: Supplementary file 8 — Source data Fig. 3 [file 44321_2024_188_MOESM8_ESM.zip › Figure 3/3I-J/BMS.tif]

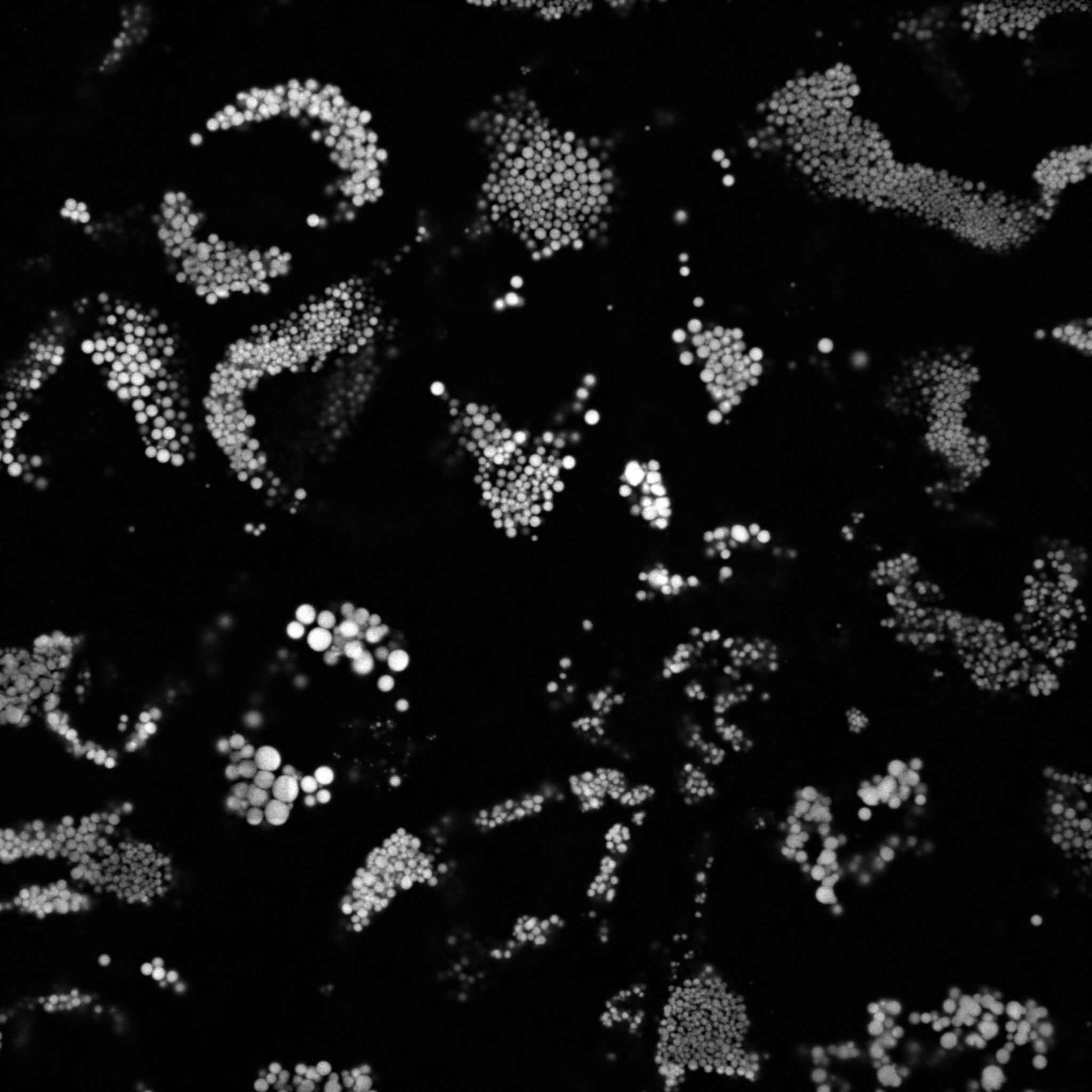

Supplement: Supplementary file 8 — Source data Fig. 3 [file 44321_2024_188_MOESM8_ESM.zip › Figure 3/3I-J/CRE-14.tif]

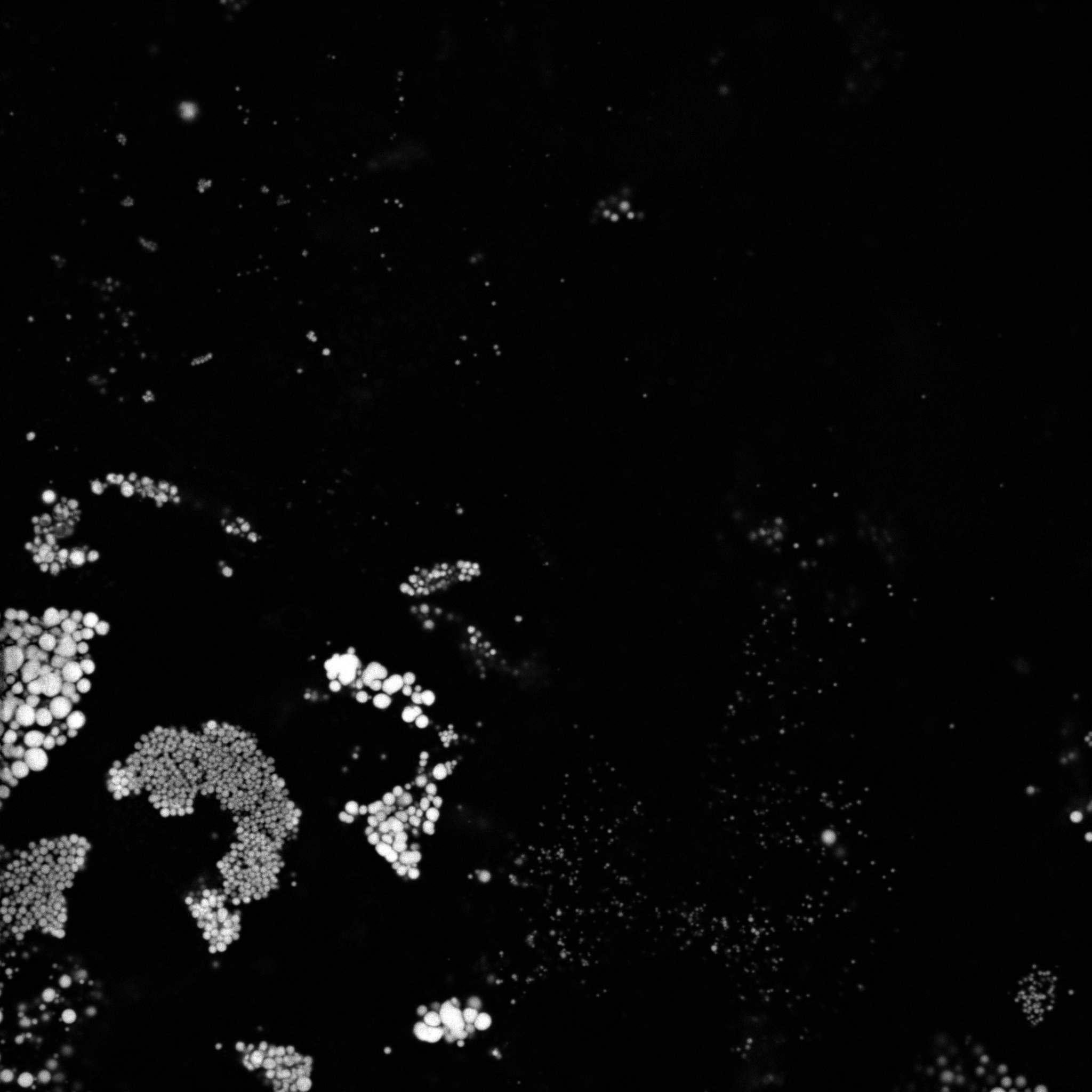

Supplement: Supplementary file 8 — Source data Fig. 3 [file 44321_2024_188_MOESM8_ESM.zip › Figure 3/3I-J/DMSO.tif]

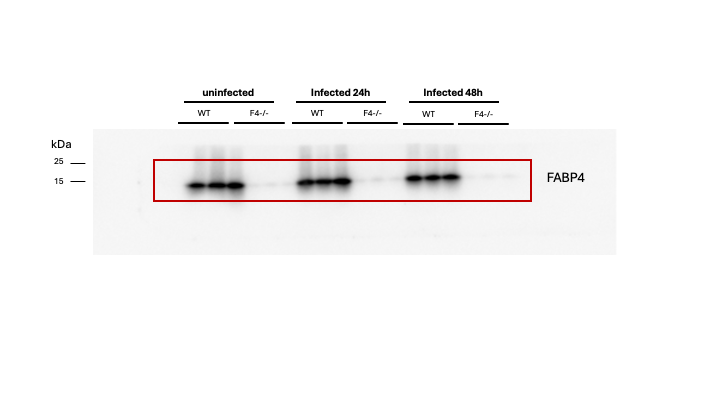

Supplement: Supplementary file 8 — Source data Fig. 3 [file 44321_2024_188_MOESM8_ESM.zip › Figure 3/3F-G/FABP4.tiff]

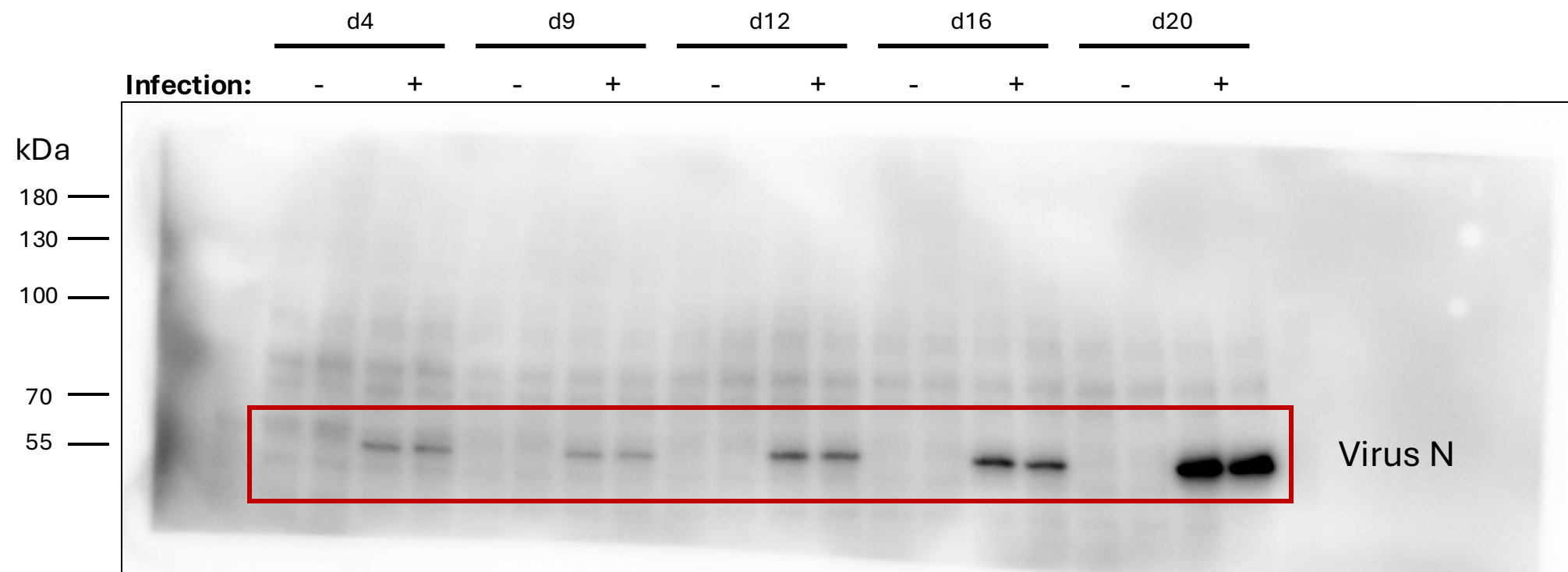

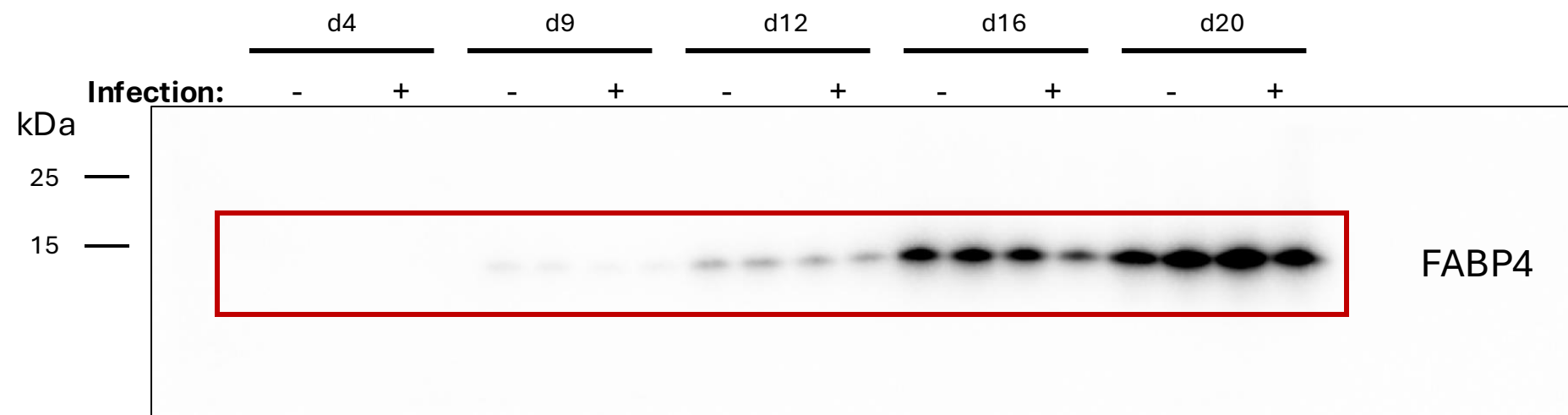

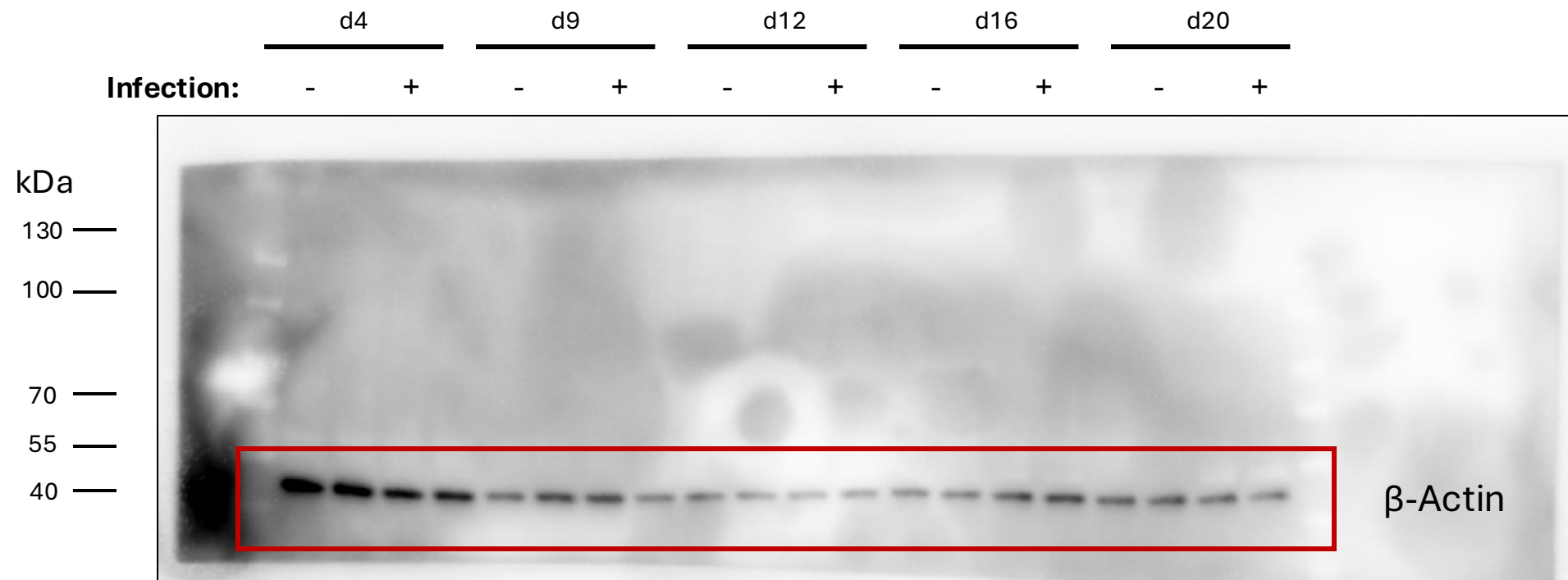

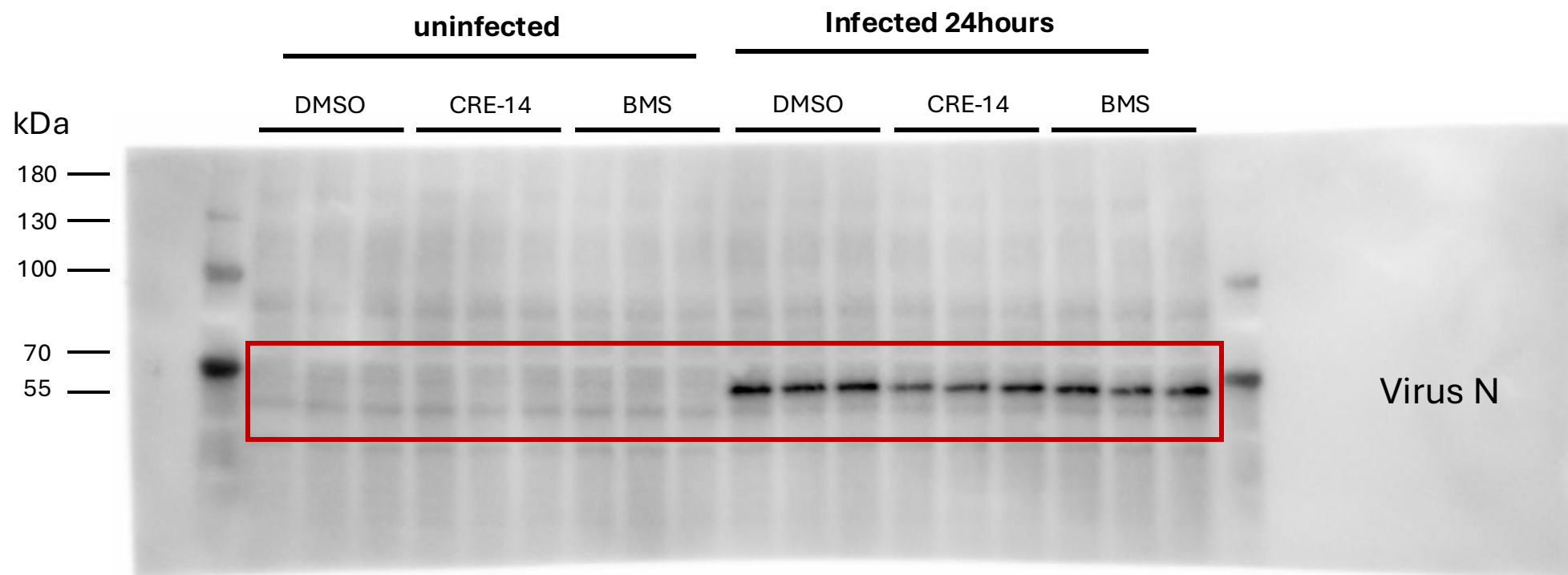

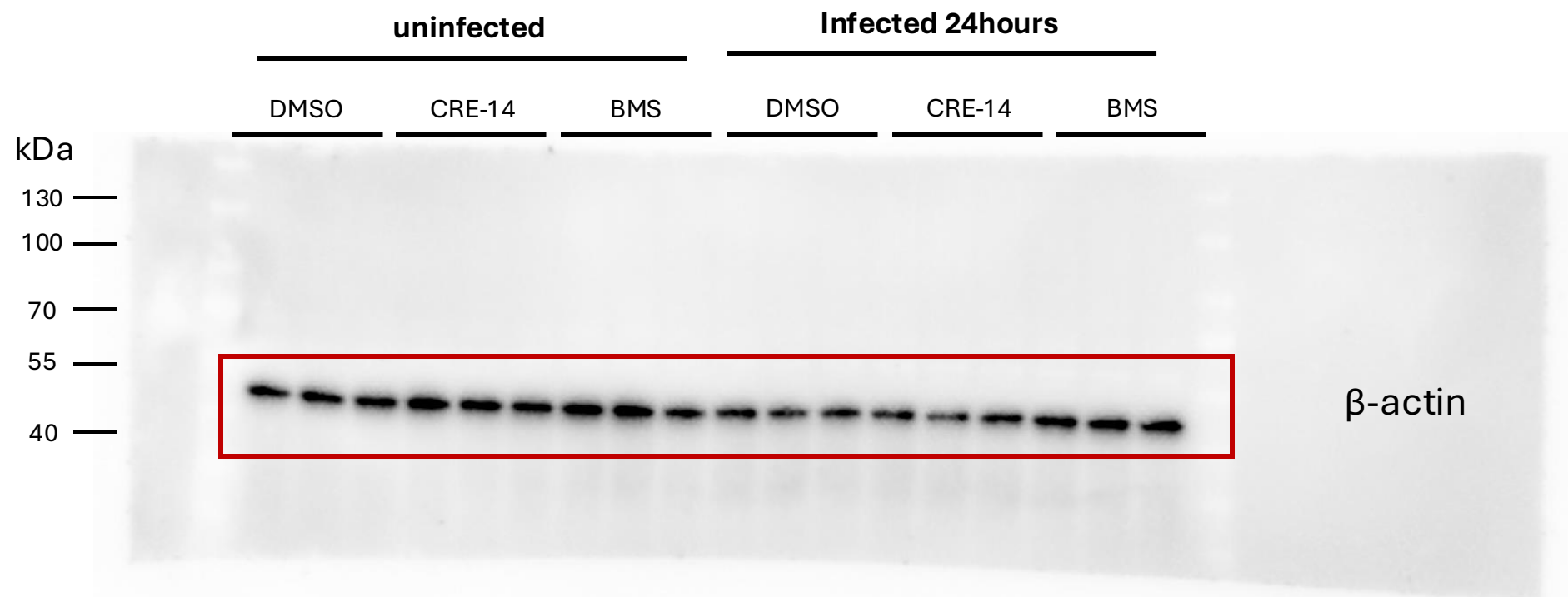

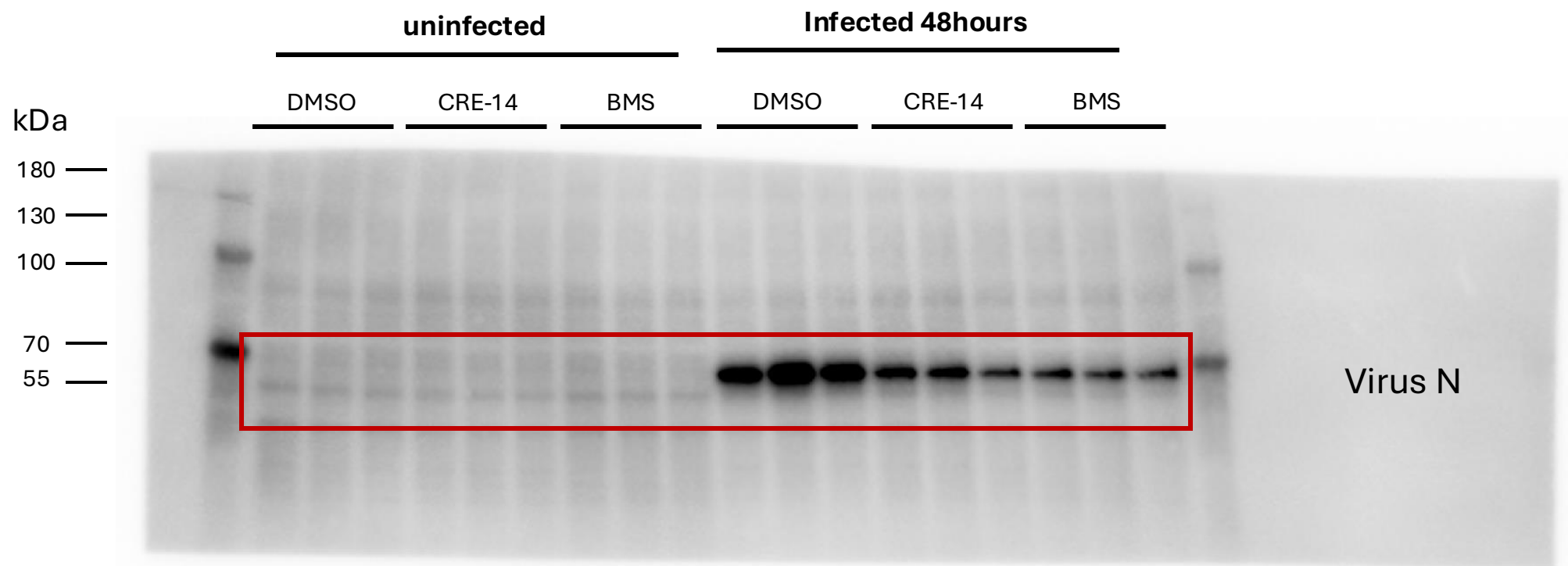

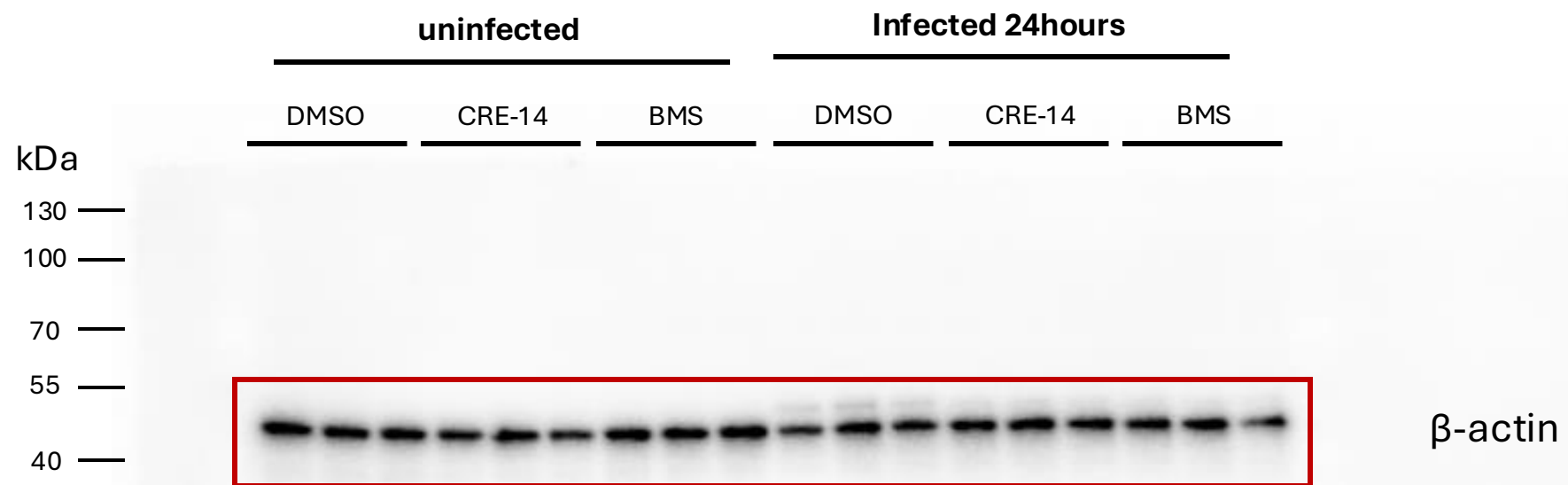

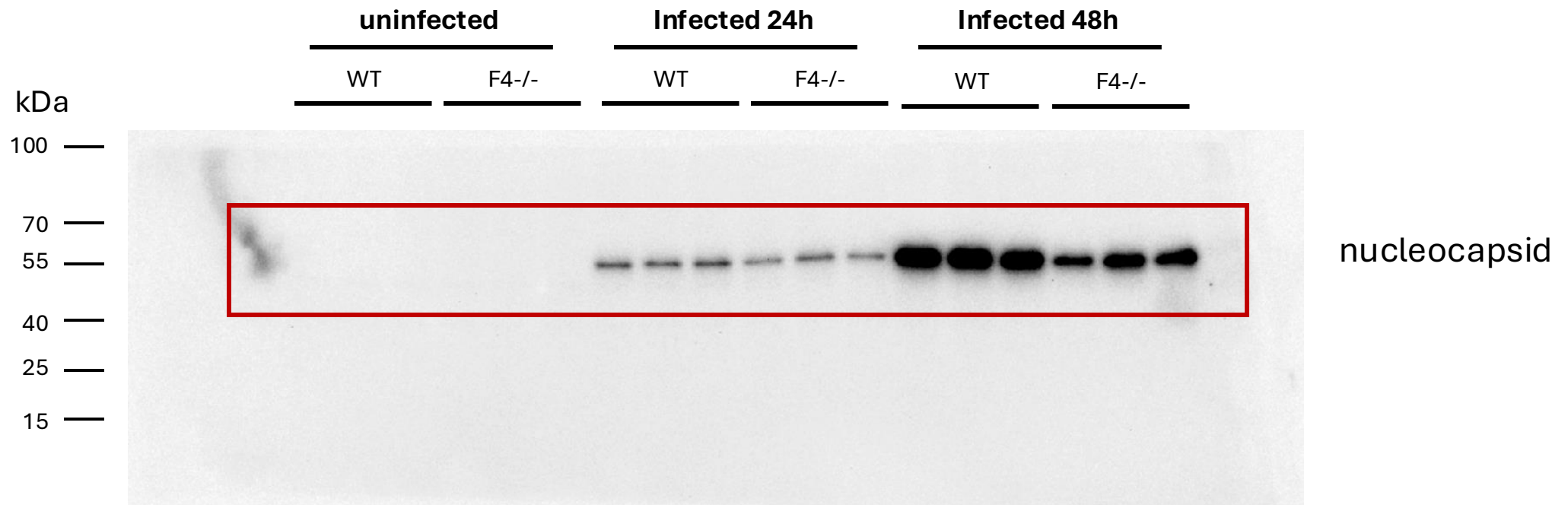

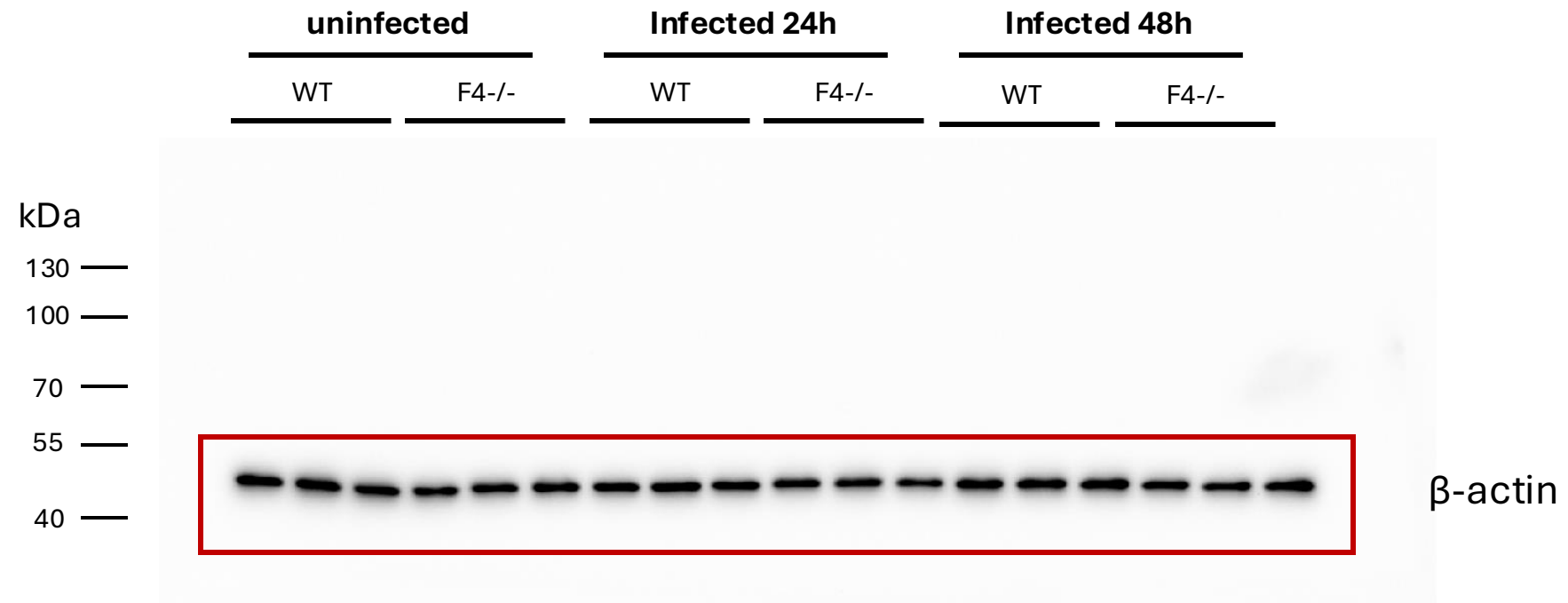

Supplement: Supplementary file 8 — Source data Fig. 3 [file 44321_2024_188_MOESM8_ESM.zip › Figure 3/3F-G/actin.pdf]

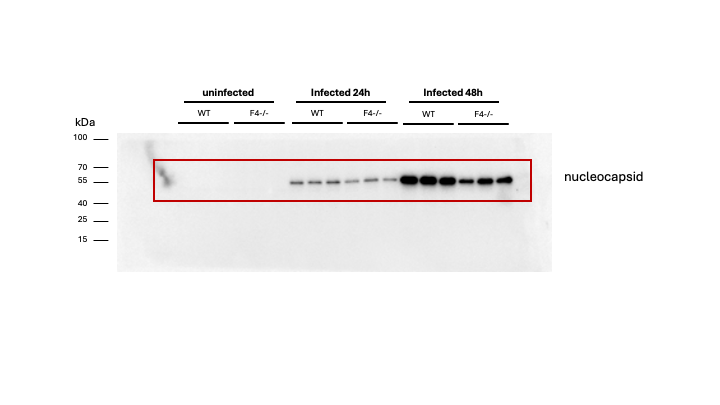

Supplement: Supplementary file 8 — Source data Fig. 3 [file 44321_2024_188_MOESM8_ESM.zip › Figure 3/3F-G/nucleocapsid.tiff]

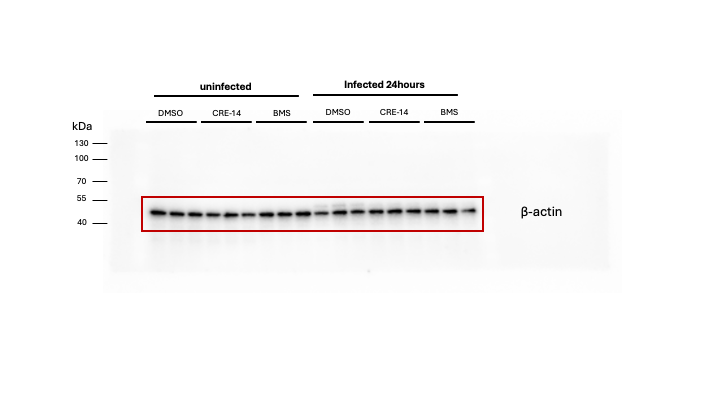

Supplement: Supplementary file 8 — Source data Fig. 3 [file 44321_2024_188_MOESM8_ESM.zip › Figure 3/3B-C/48h_actin.tiff]

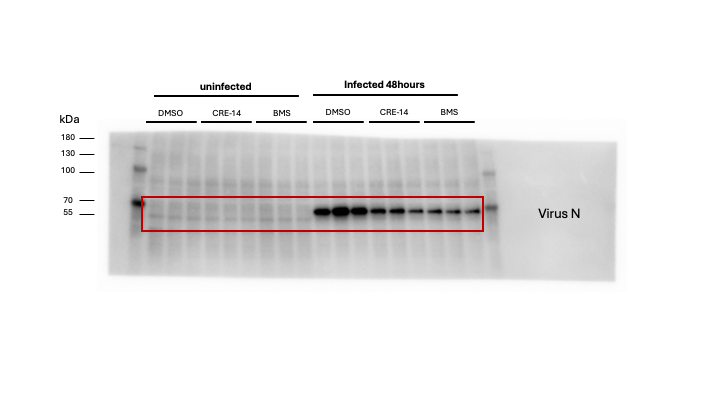

Supplement: Supplementary file 8 — Source data Fig. 3 [file 44321_2024_188_MOESM8_ESM.zip › Figure 3/3B-C/48h_nucleocapsid.tiff]

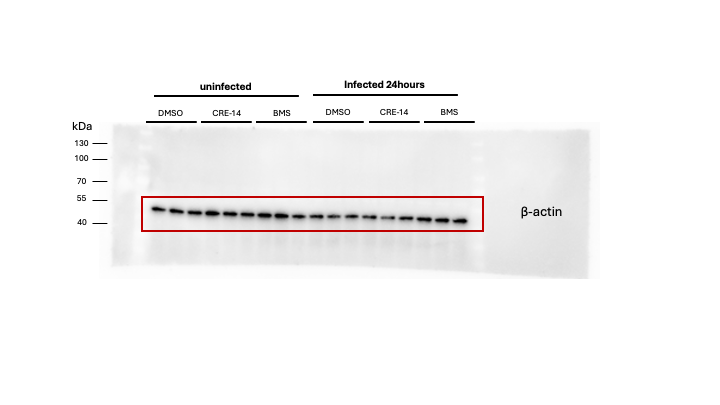

Supplement: Supplementary file 8 — Source data Fig. 3 [file 44321_2024_188_MOESM8_ESM.zip › Figure 3/3B-C/24h_actin.tiff]

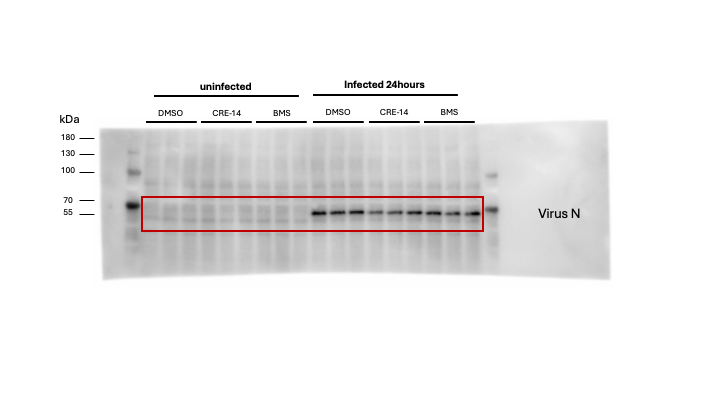

Supplement: Supplementary file 8 — Source data Fig. 3 [file 44321_2024_188_MOESM8_ESM.zip › Figure 3/3B-C/24h_nucleocapsid.tiff]

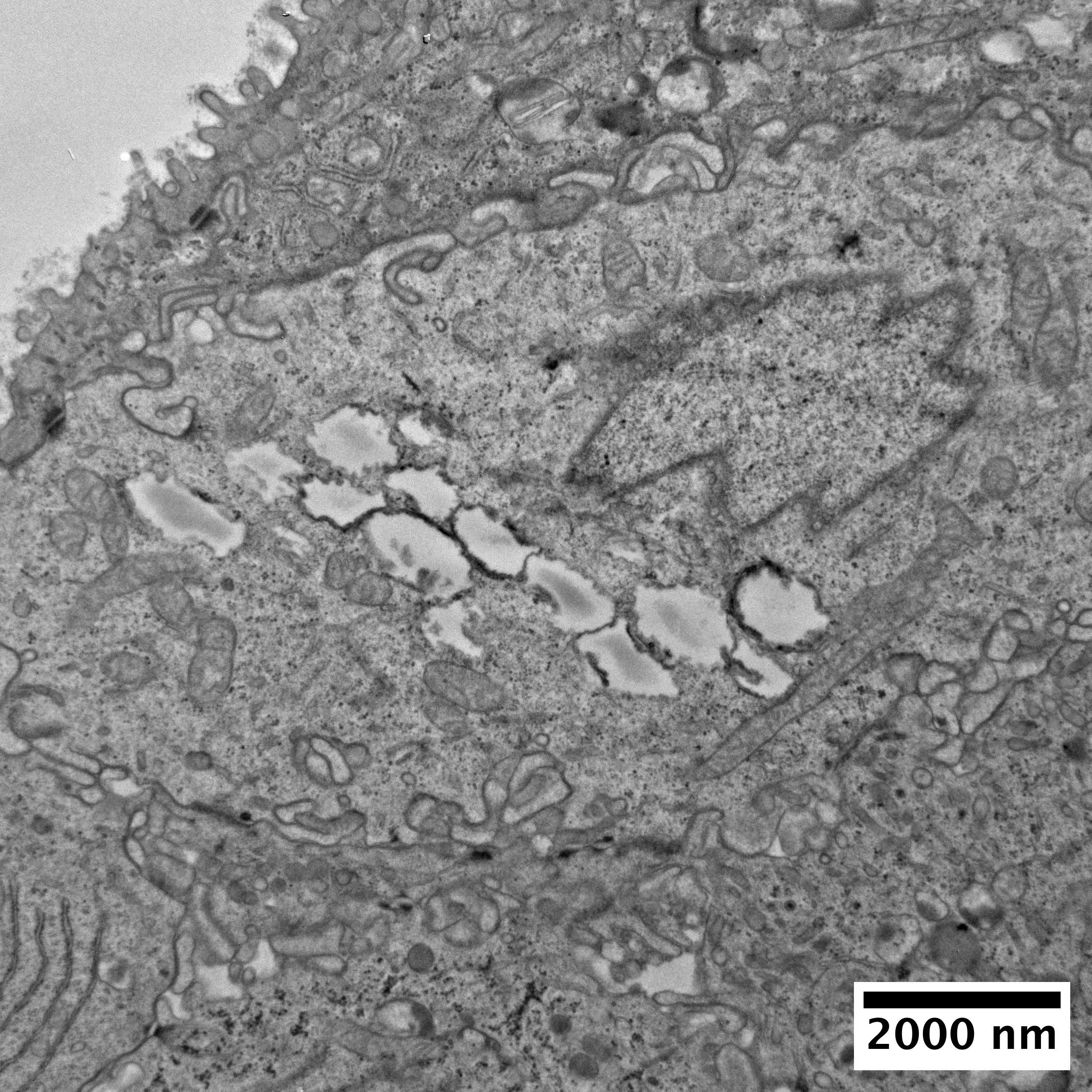

Supplement: Supplementary file 9 — Source data Fig. 4 [file 44321_2024_188_MOESM9_ESM.zip › Figure 4/4L/infected.CRE14_4k.png]

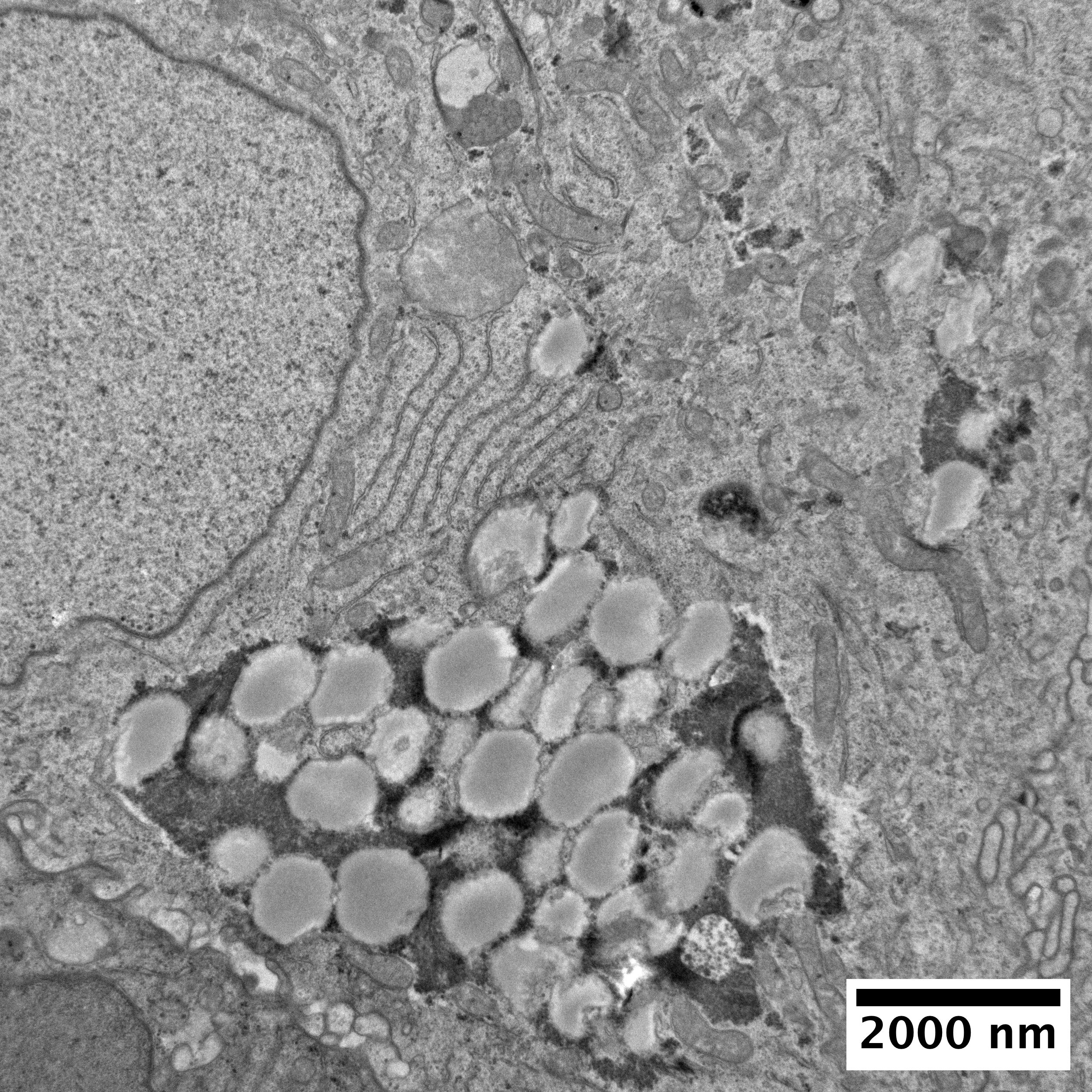

Supplement: Supplementary file 9 — Source data Fig. 4 [file 44321_2024_188_MOESM9_ESM.zip › Figure 4/4L/infected.DMSO_4k.png]

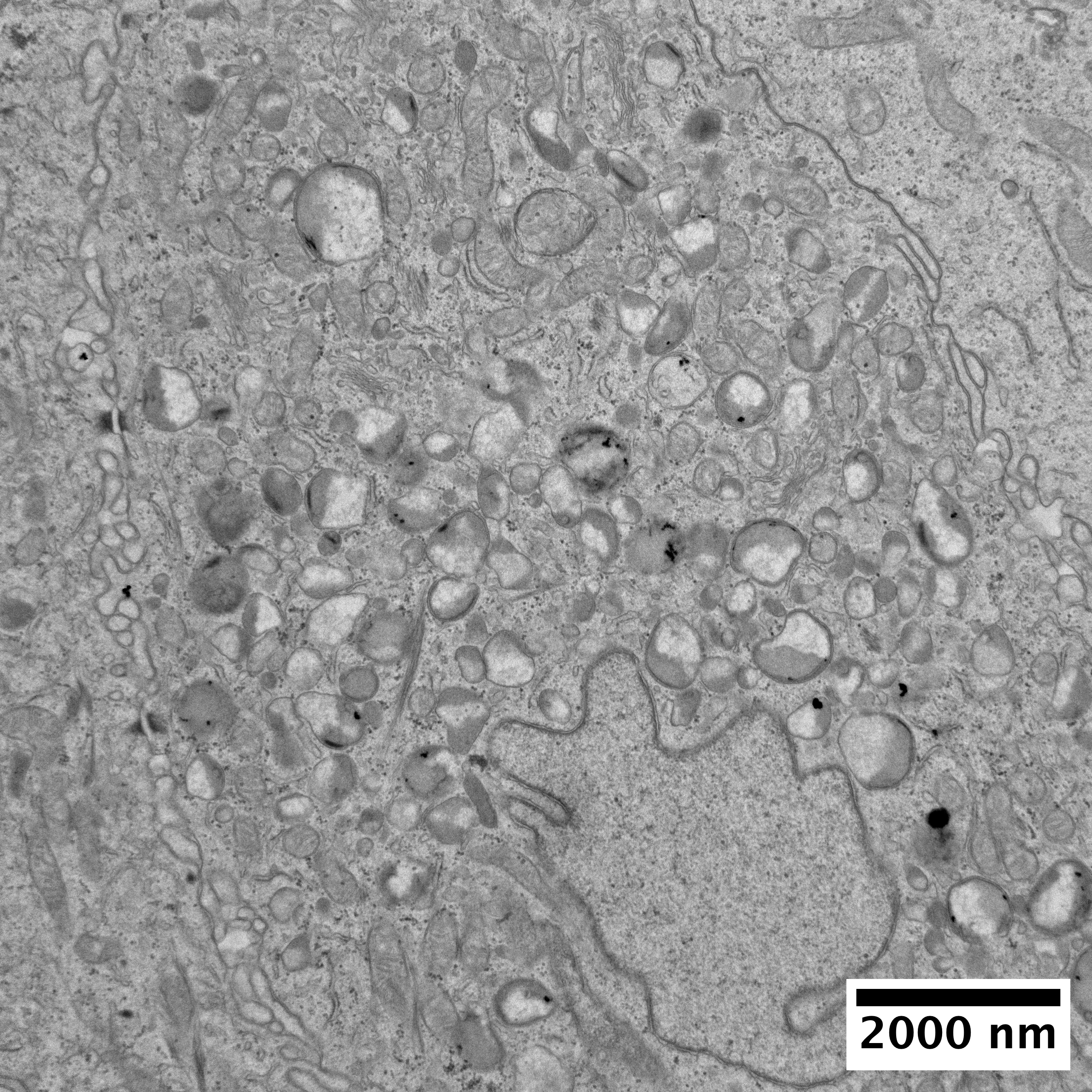

Supplement: Supplementary file 9 — Source data Fig. 4 [file 44321_2024_188_MOESM9_ESM.zip › Figure 4/4F-K/infected.DMSO_4k.png]

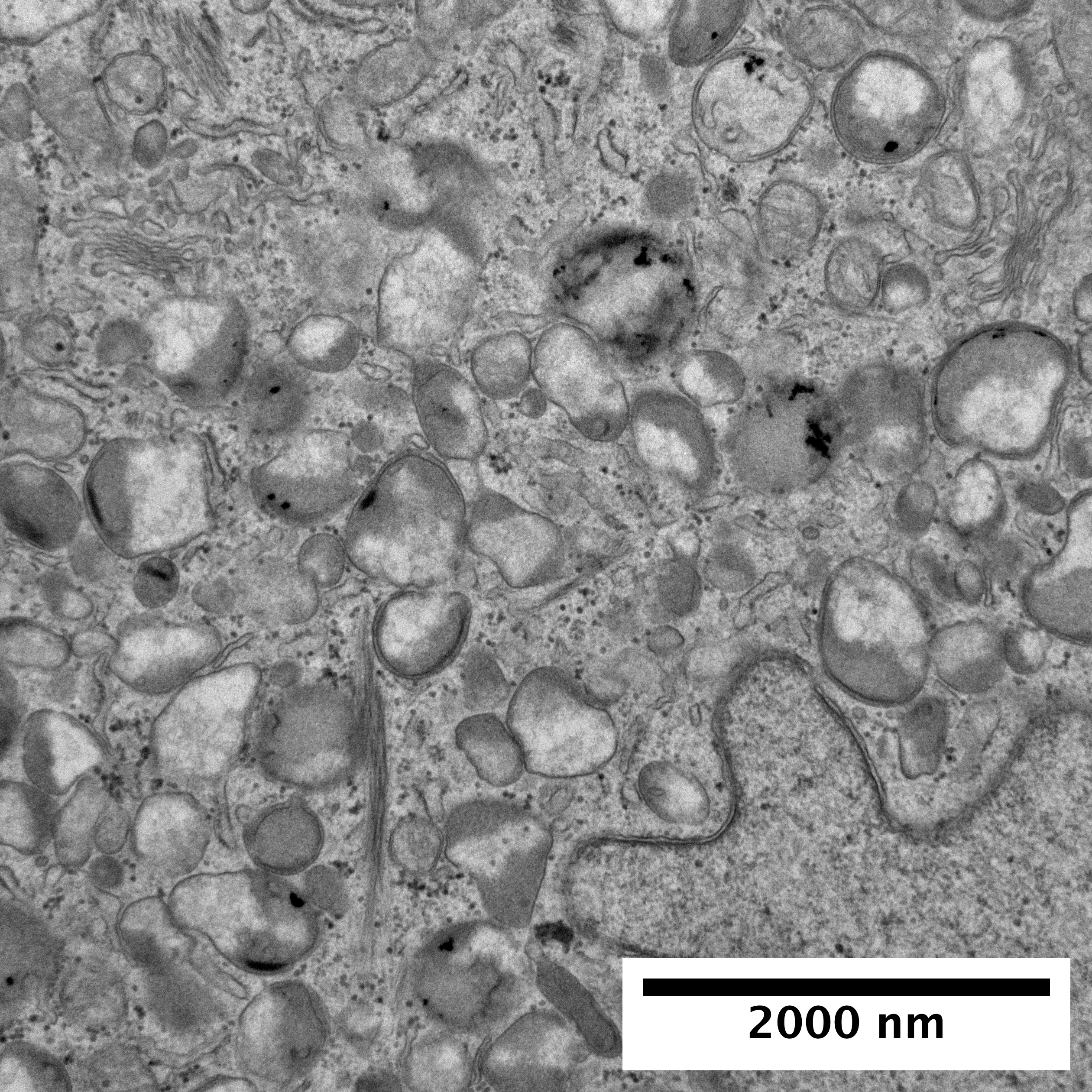

Supplement: Supplementary file 9 — Source data Fig. 4 [file 44321_2024_188_MOESM9_ESM.zip › Figure 4/4F-K/infected.DMSO_8k.png]

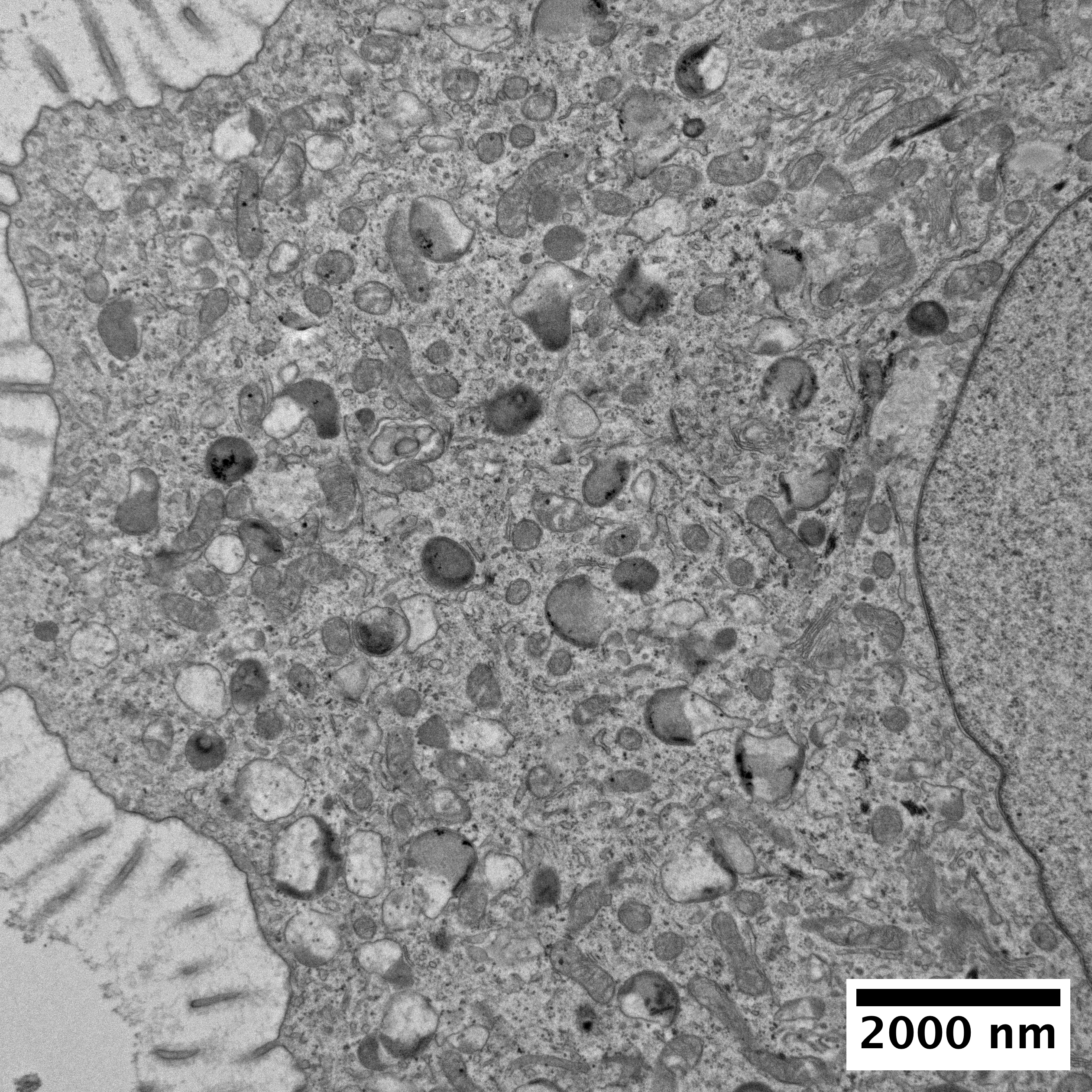

Supplement: Supplementary file 9 — Source data Fig. 4 [file 44321_2024_188_MOESM9_ESM.zip › Figure 4/4F-K/infected.CRE14_4k.png]

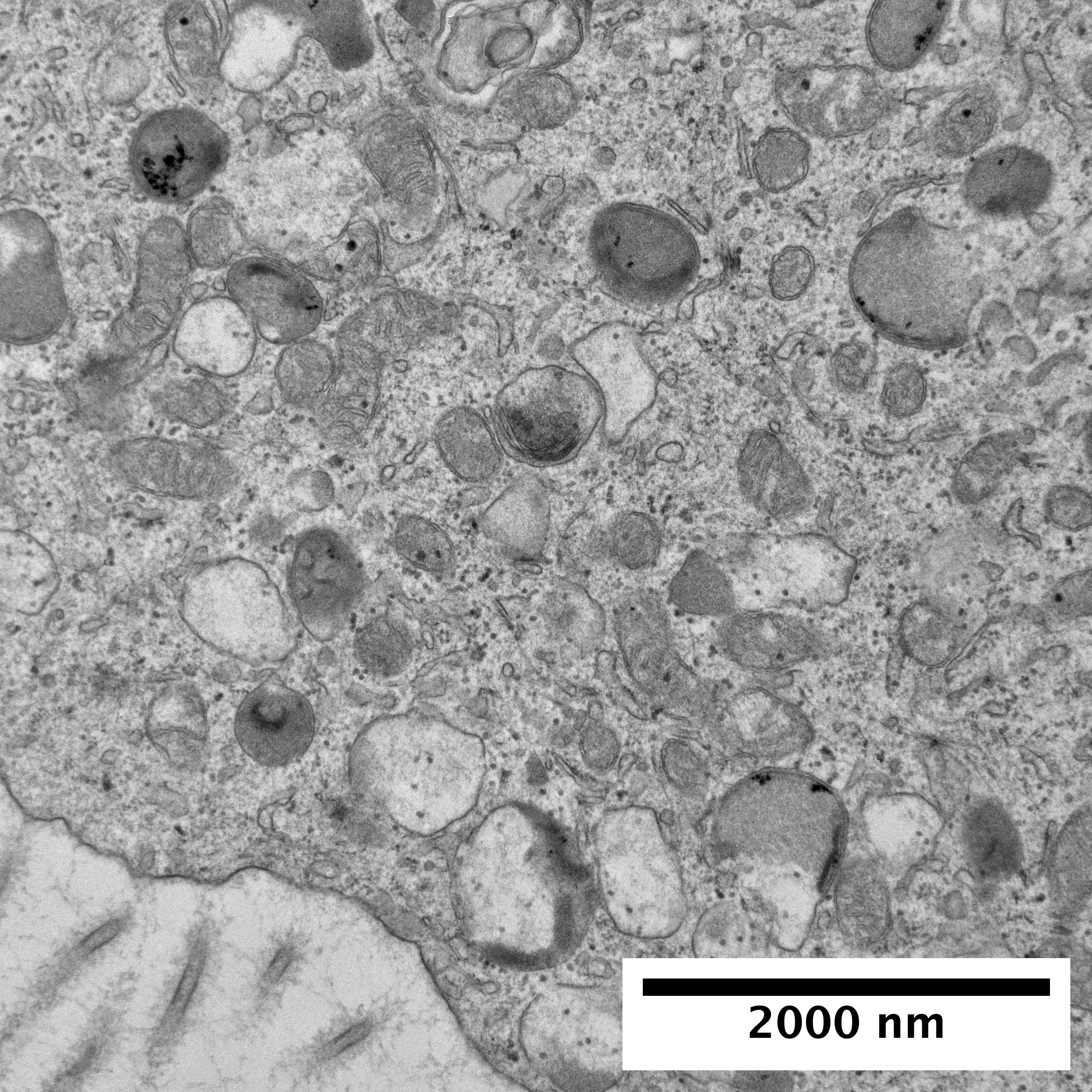

Supplement: Supplementary file 9 — Source data Fig. 4 [file 44321_2024_188_MOESM9_ESM.zip › Figure 4/4F-K/infected.CRE14_8k.png]

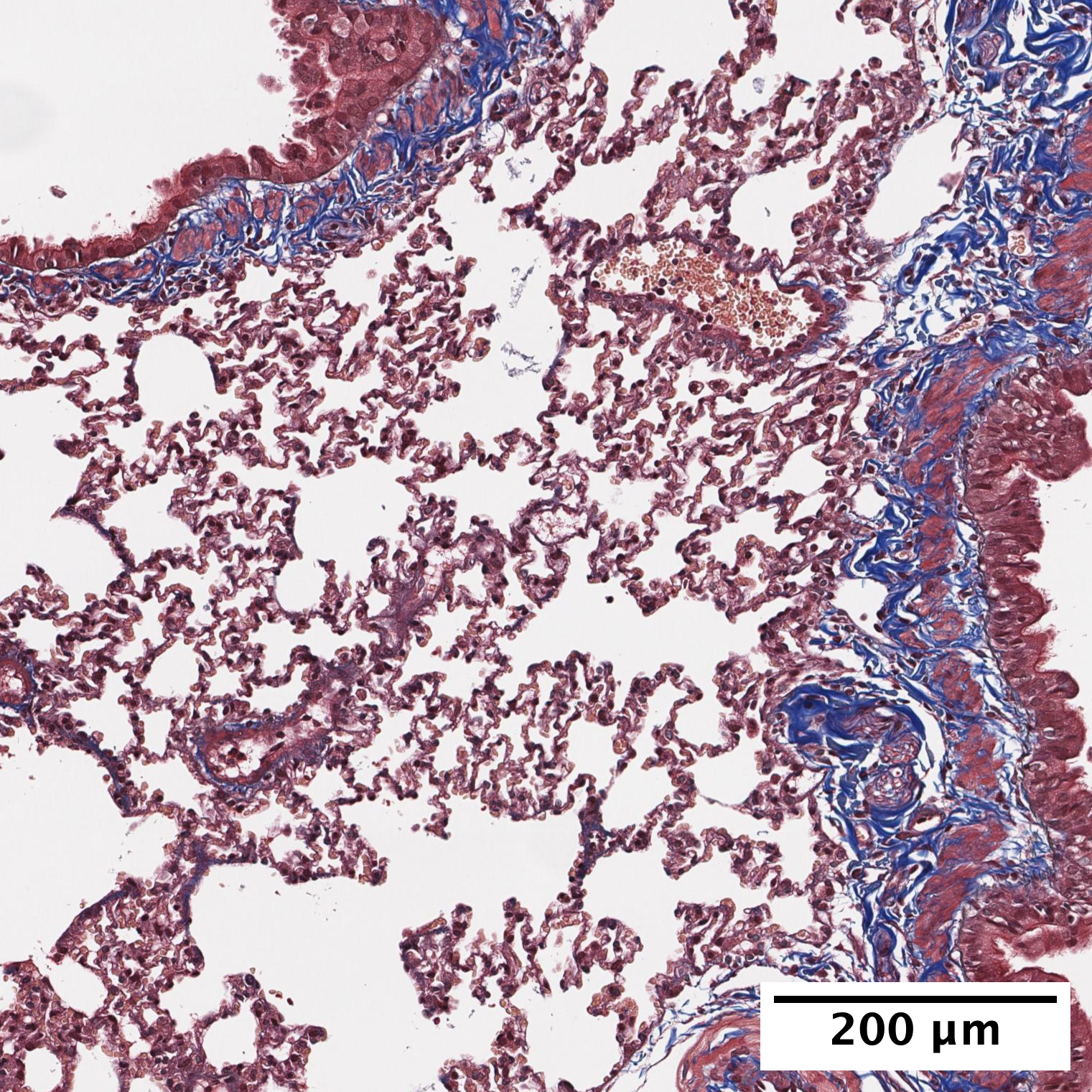

Supplement: Supplementary file 10 — Source data Fig. 5 [file 44321_2024_188_MOESM10_ESM.zip › Figure 5/5M/Trichrome_inf.CRE14_x10.png]

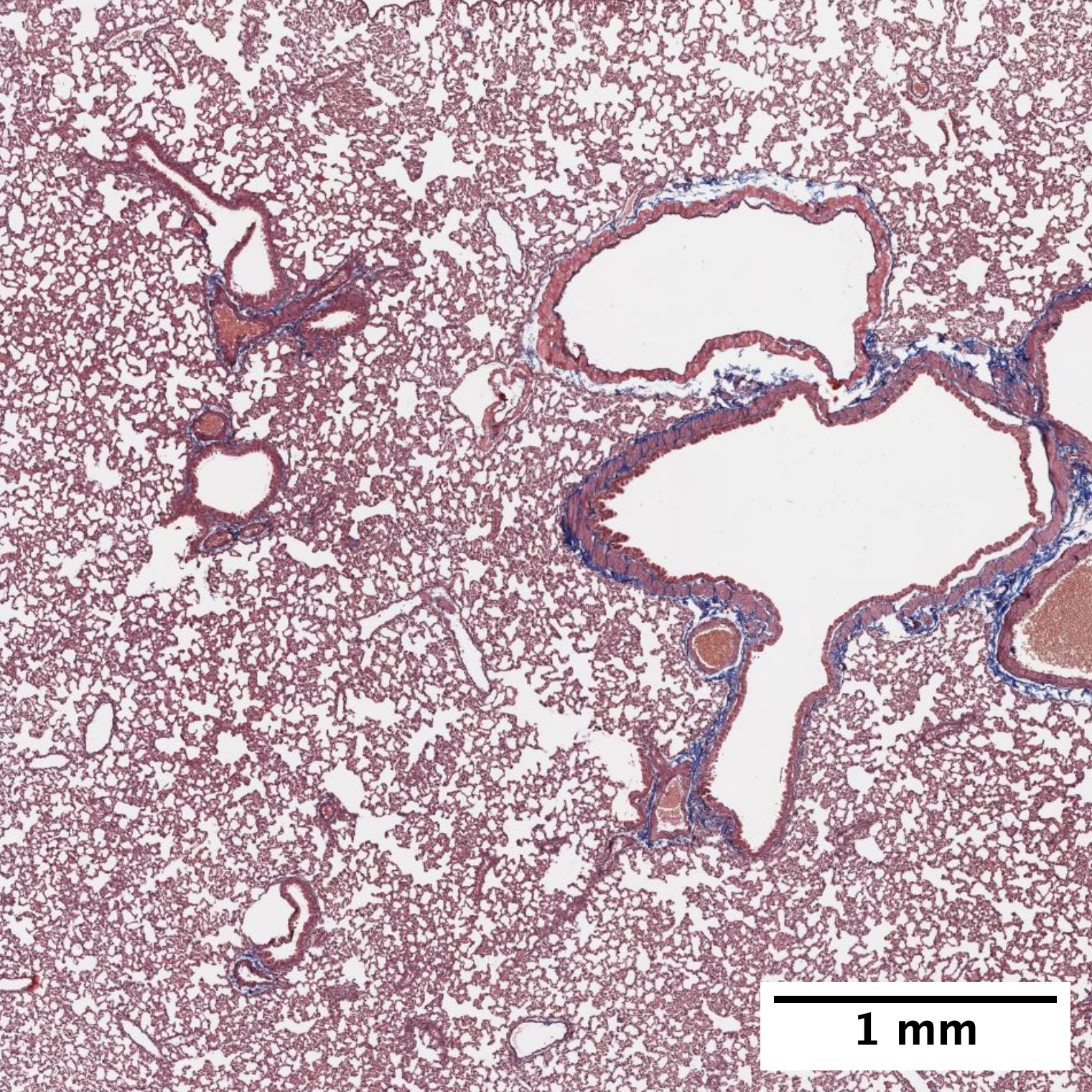

Supplement: Supplementary file 10 — Source data Fig. 5 [file 44321_2024_188_MOESM10_ESM.zip › Figure 5/5M/Trichrome_uninf.veh_x2.png]

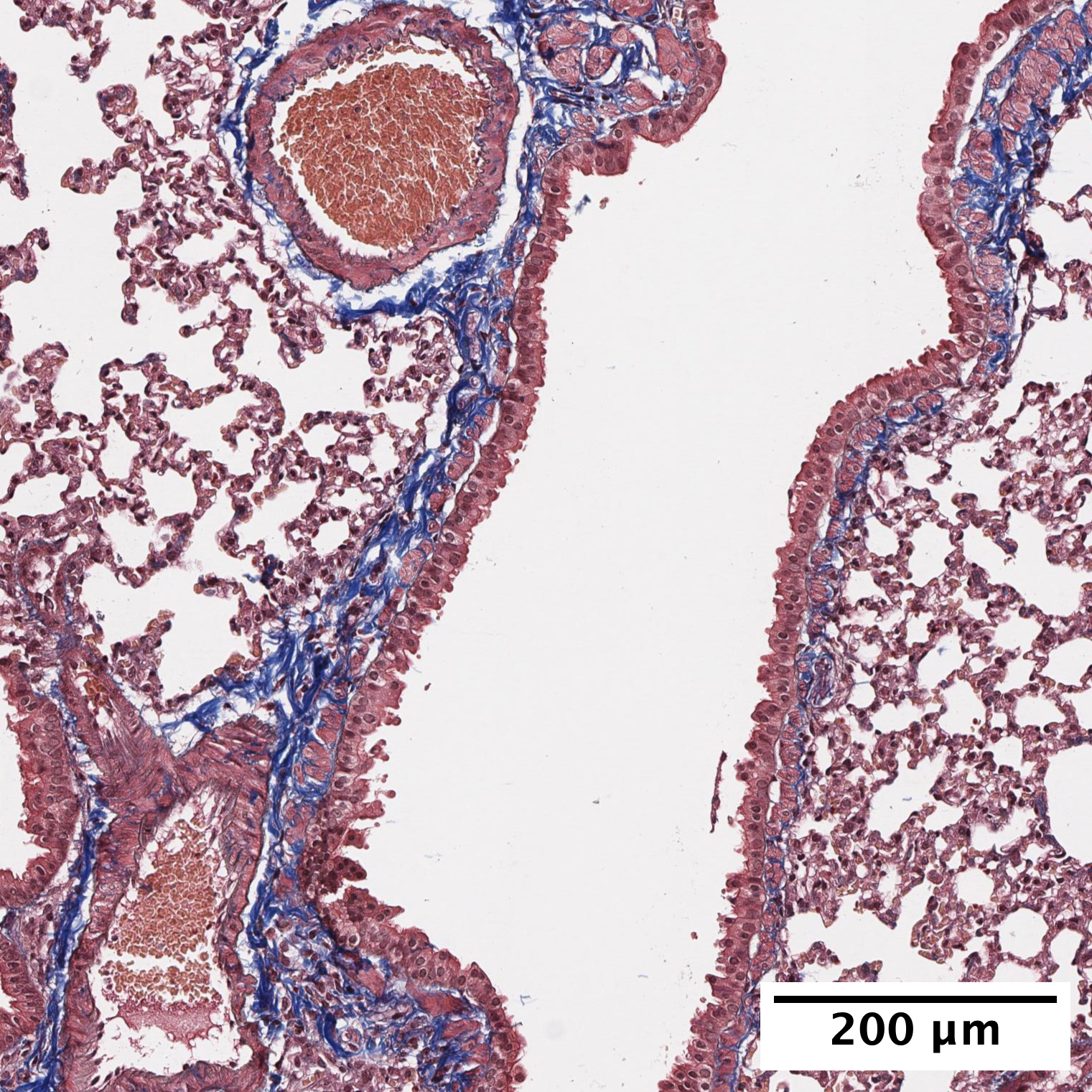

Supplement: Supplementary file 10 — Source data Fig. 5 [file 44321_2024_188_MOESM10_ESM.zip › Figure 5/5M/Trichrome_uninf.veh_x10.png]

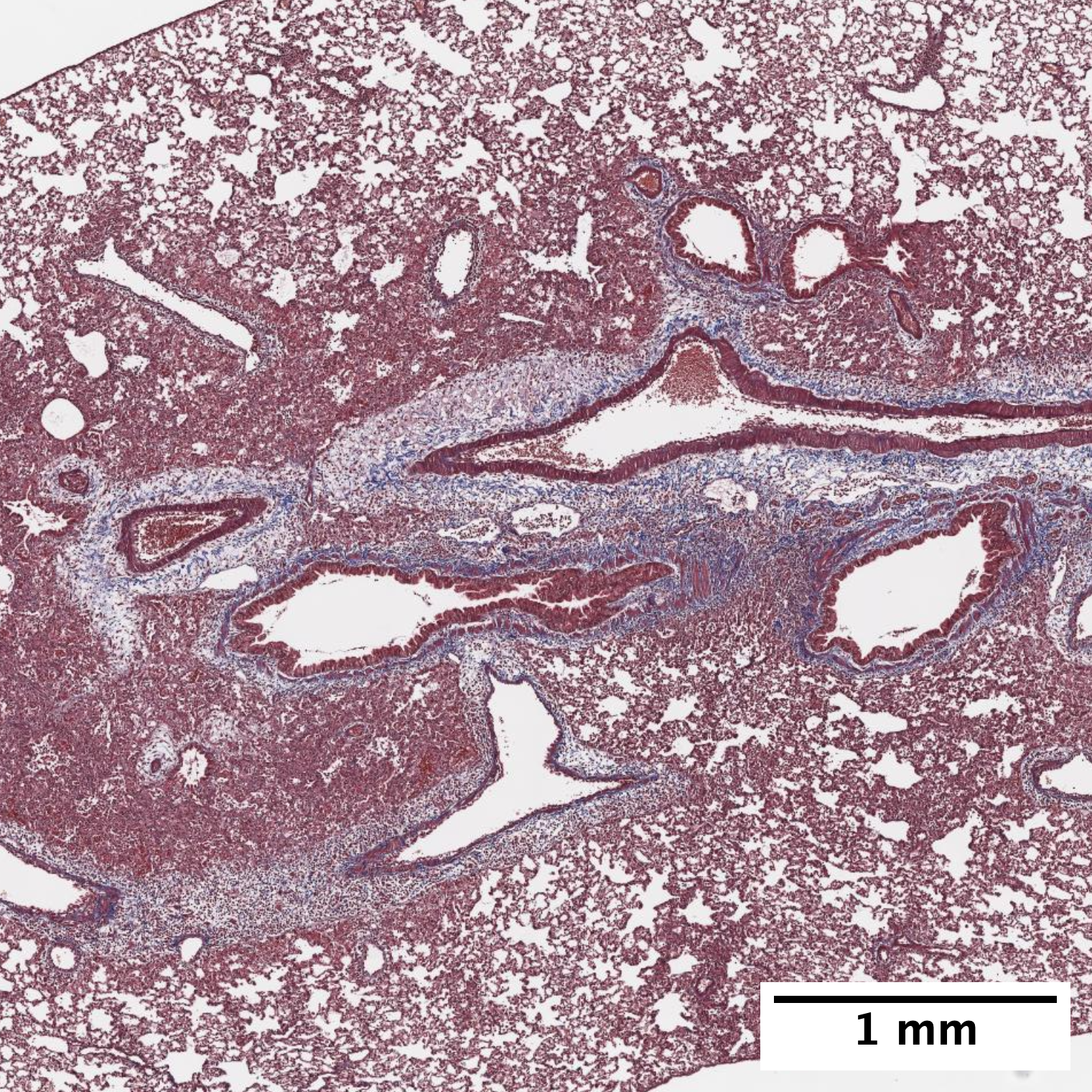

Supplement: Supplementary file 10 — Source data Fig. 5 [file 44321_2024_188_MOESM10_ESM.zip › Figure 5/5M/Trichrome_inf.veh_x2.png]

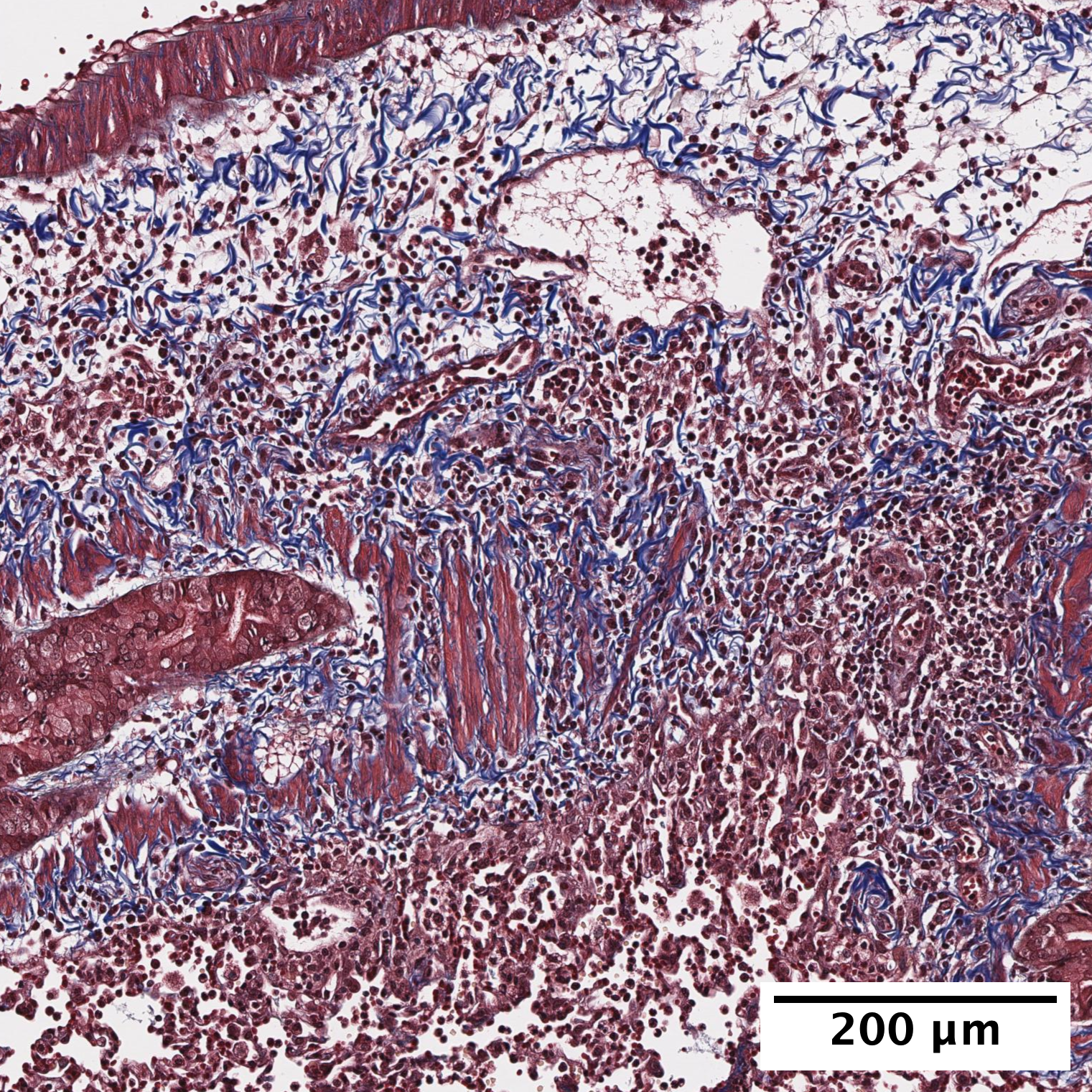

Supplement: Supplementary file 10 — Source data Fig. 5 [file 44321_2024_188_MOESM10_ESM.zip › Figure 5/5M/Trichrome_inf.veh_x10.png]

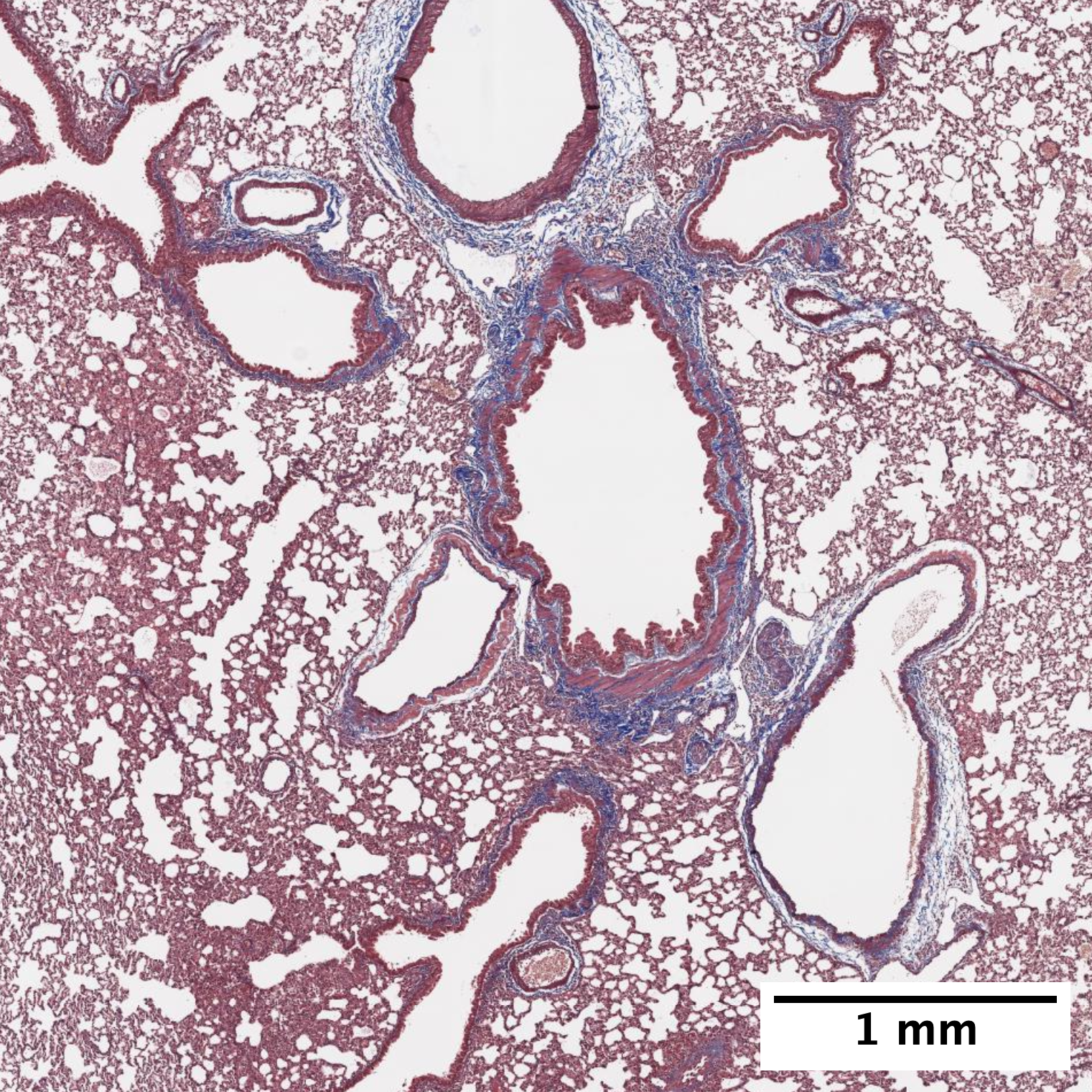

Supplement: Supplementary file 10 — Source data Fig. 5 [file 44321_2024_188_MOESM10_ESM.zip › Figure 5/5M/Trichrome_inf.CRE14_x2.png]

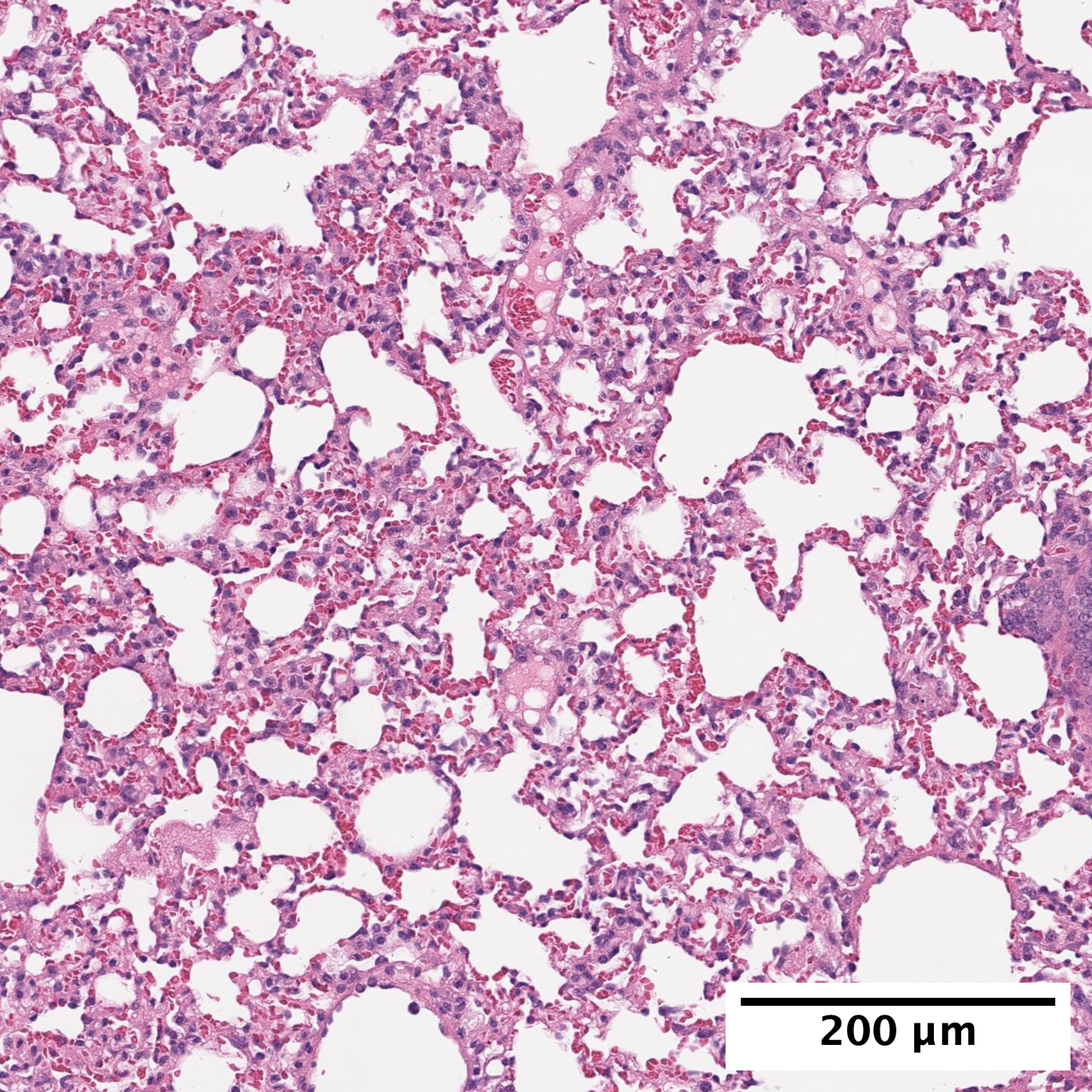

Supplement: Supplementary file 10 — Source data Fig. 5 [file 44321_2024_188_MOESM10_ESM.zip › Figure 5/5F-L/HE_infected.CRE-14_x10.png]

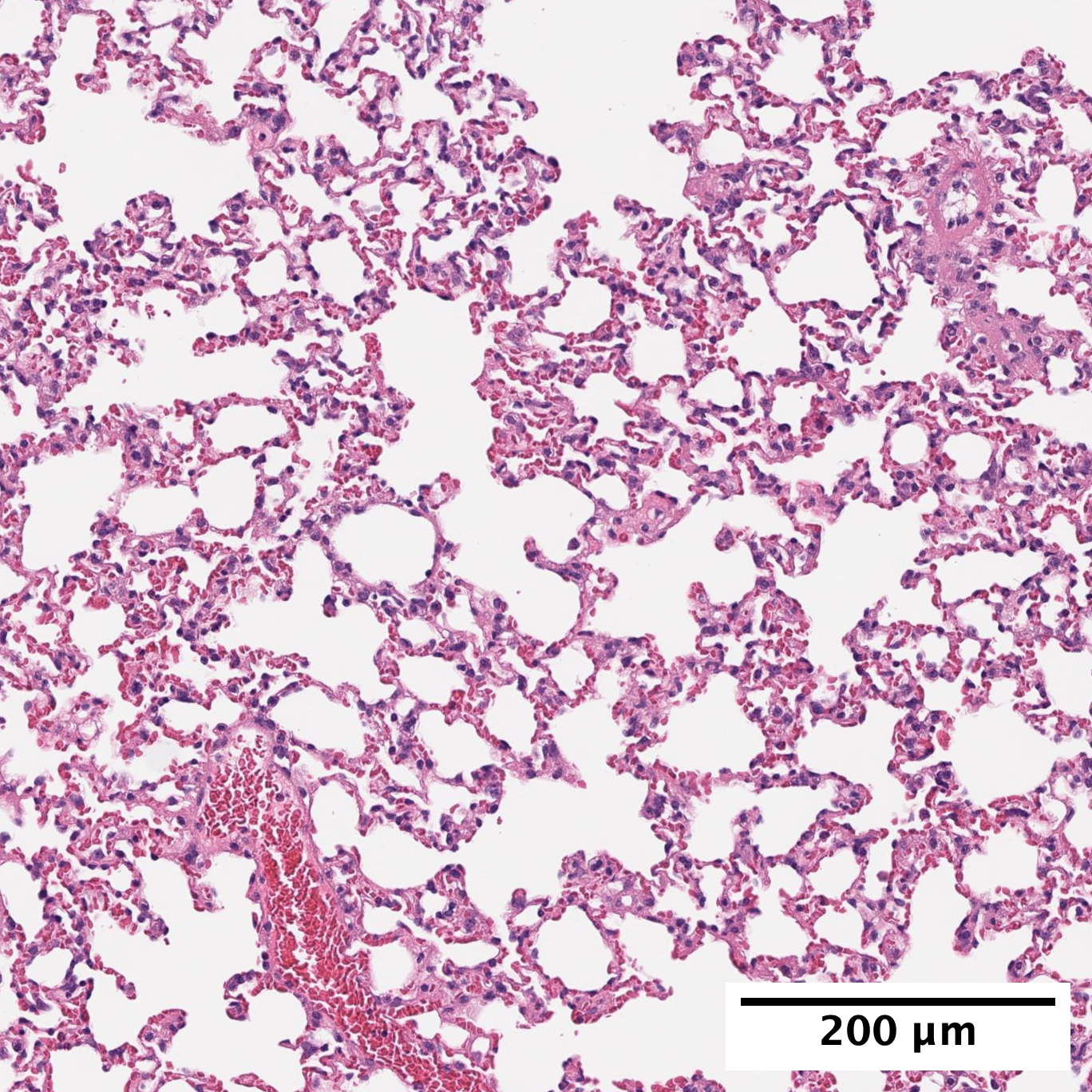

Supplement: Supplementary file 10 — Source data Fig. 5 [file 44321_2024_188_MOESM10_ESM.zip › Figure 5/5F-L/HE_Vehicle_x10.png]

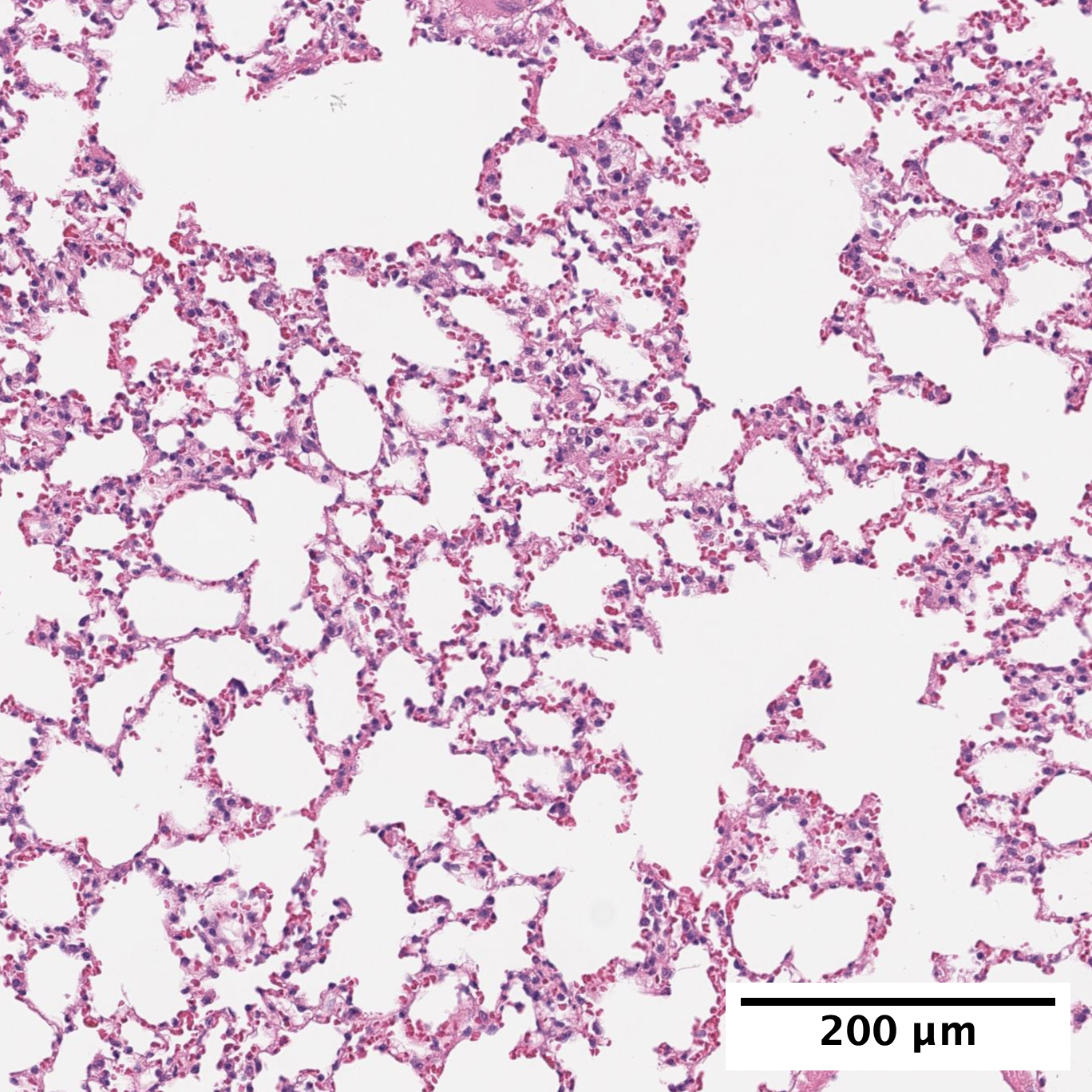

Supplement: Supplementary file 10 — Source data Fig. 5 [file 44321_2024_188_MOESM10_ESM.zip › Figure 5/5F-L/HE_CRE-14_x10.png]

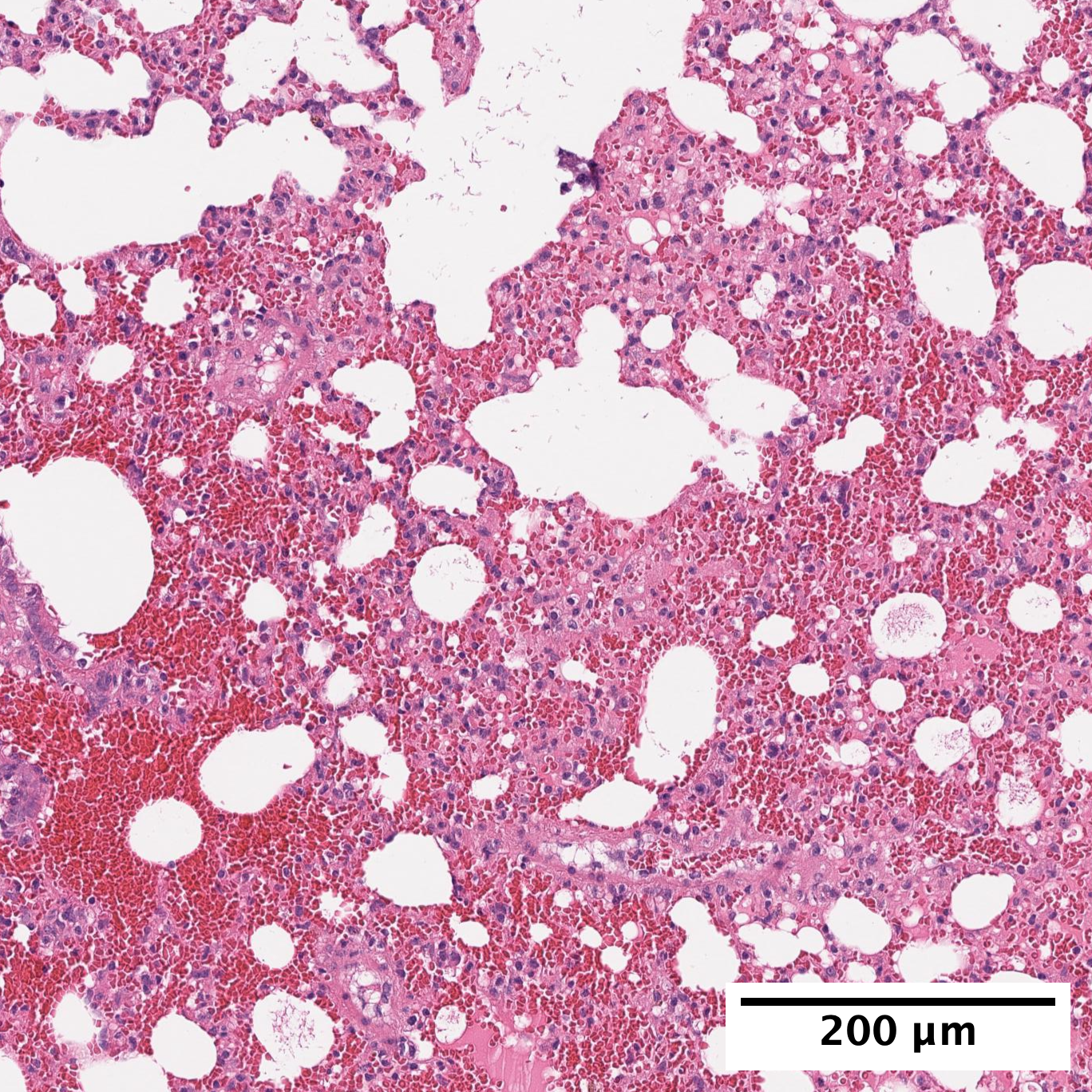

Supplement: Supplementary file 10 — Source data Fig. 5 [file 44321_2024_188_MOESM10_ESM.zip › Figure 5/5F-L/HE_infected.vehicle_x10.png]

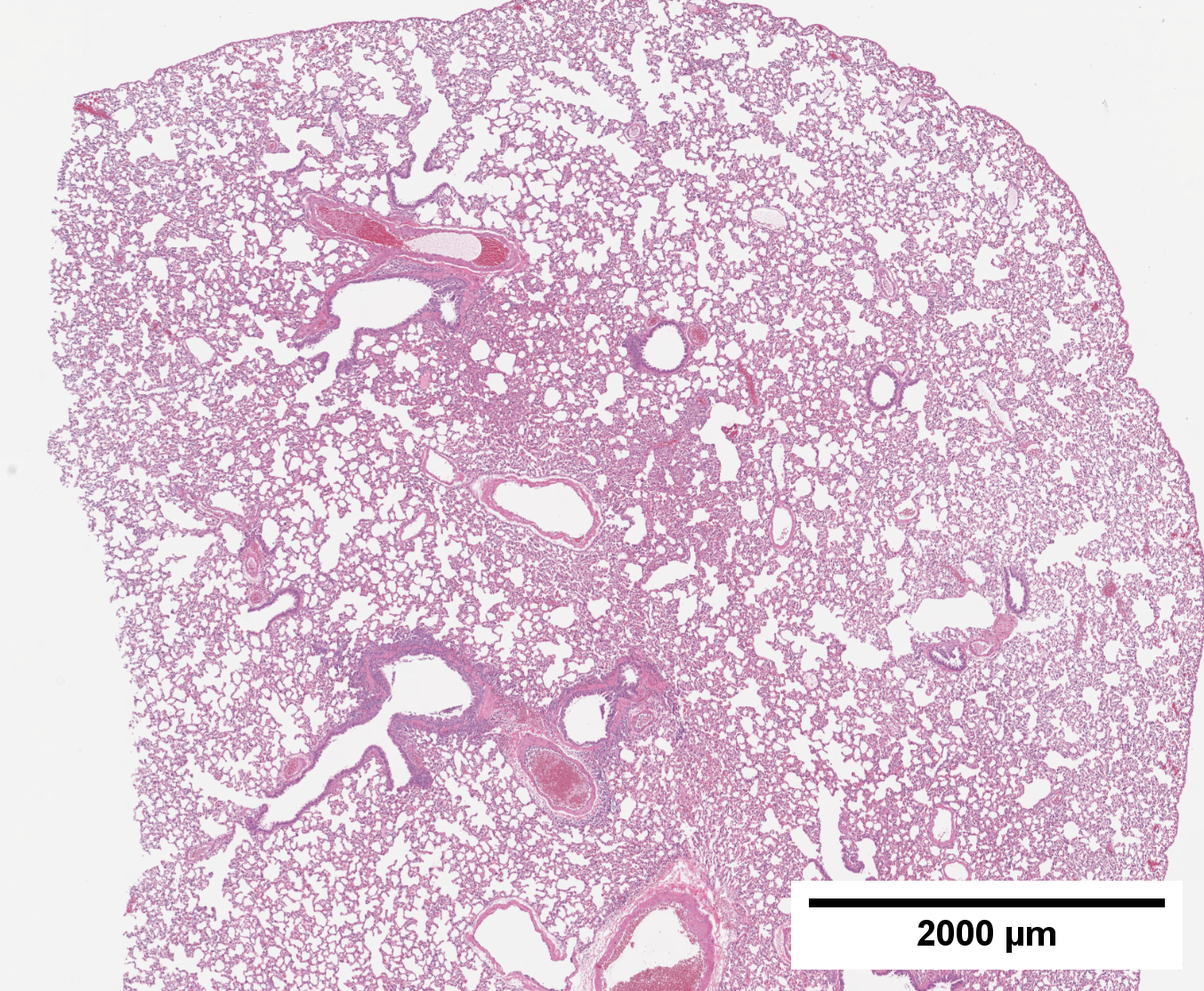

Supplement: Supplementary file 10 — Source data Fig. 5 [file 44321_2024_188_MOESM10_ESM.zip › Figure 5/5F-L/HE_infected.CRE-14_cropped.png]

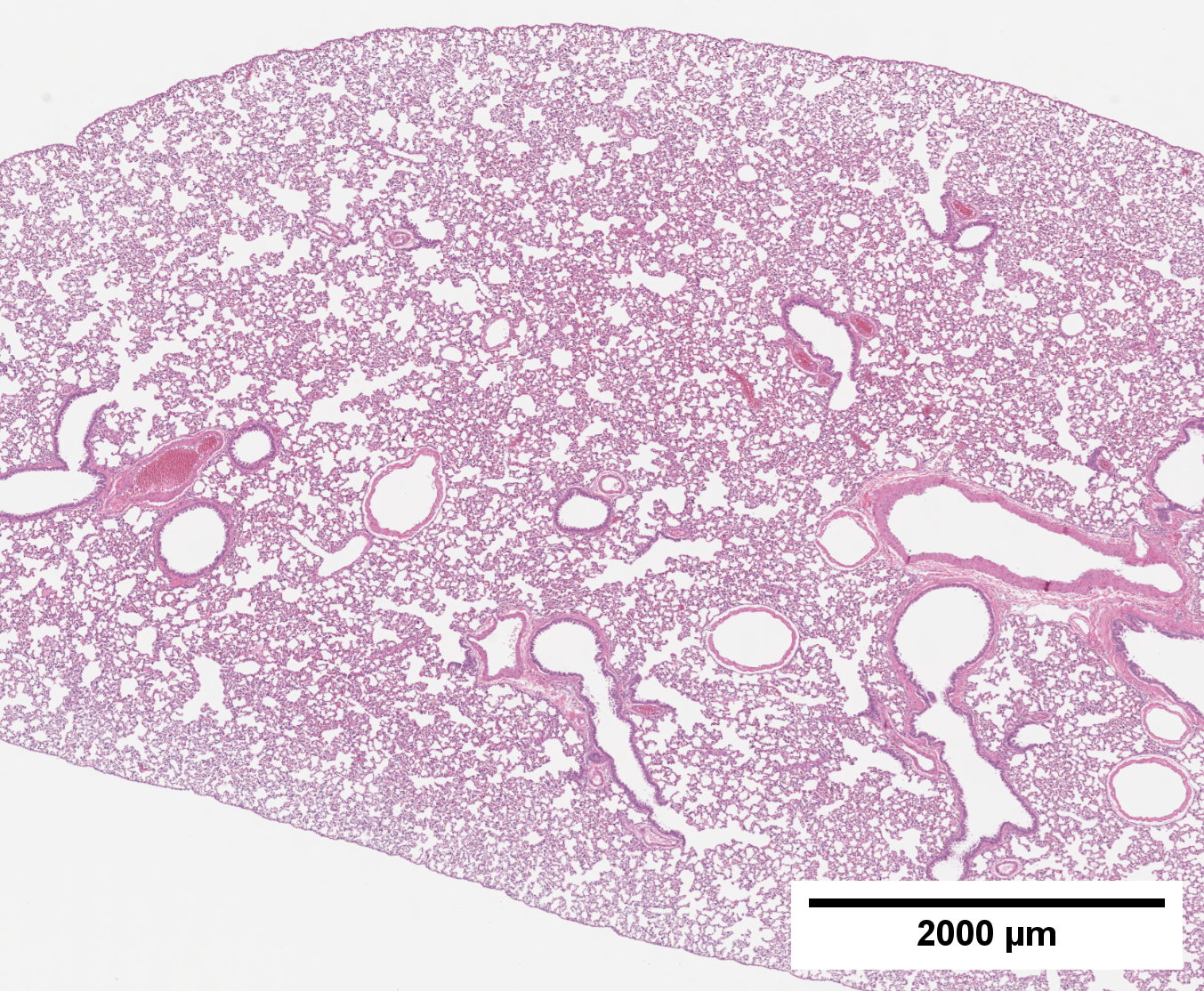

Supplement: Supplementary file 10 — Source data Fig. 5 [file 44321_2024_188_MOESM10_ESM.zip › Figure 5/5F-L/HE_vehicle_cropped.png]

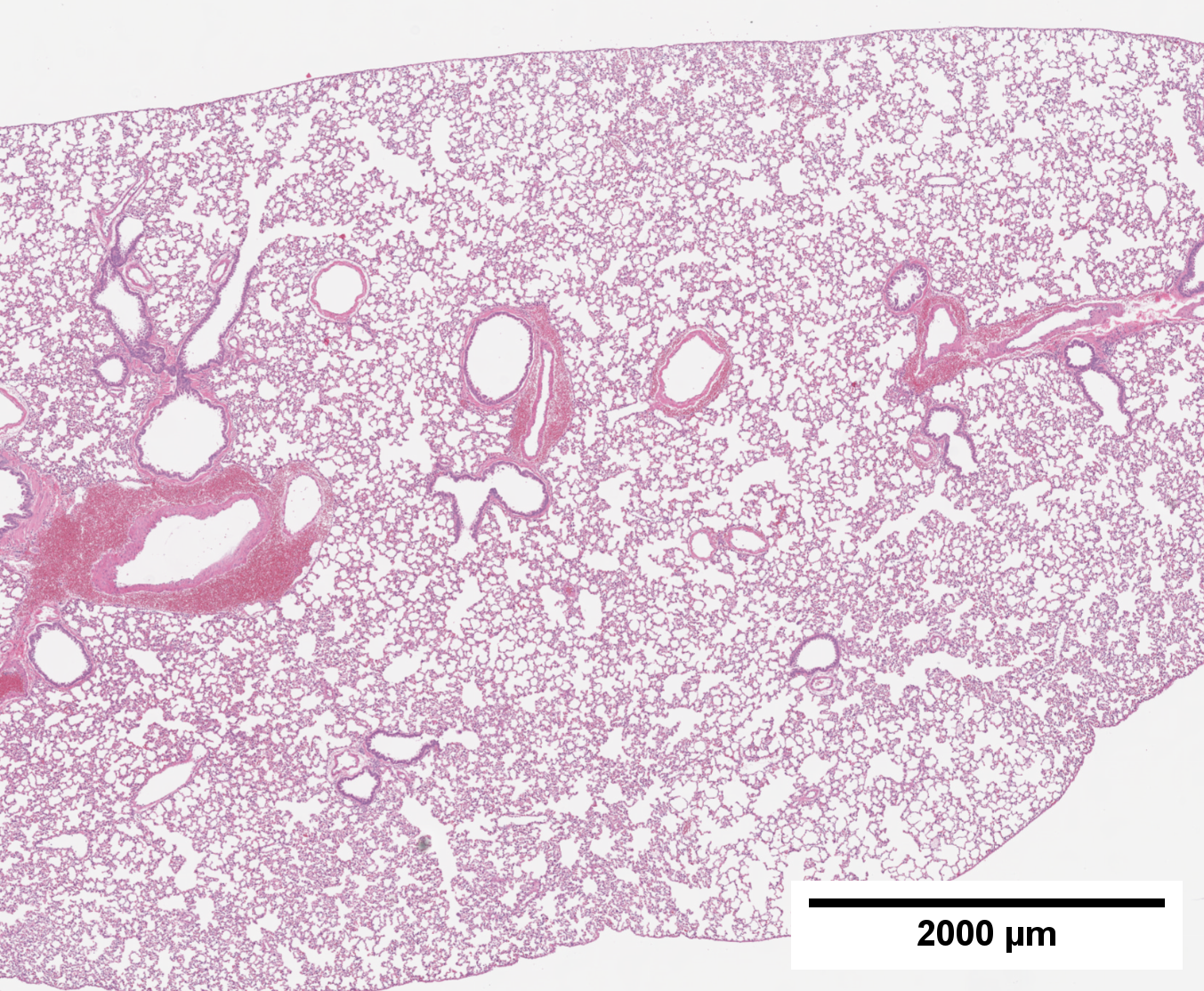

Supplement: Supplementary file 10 — Source data Fig. 5 [file 44321_2024_188_MOESM10_ESM.zip › Figure 5/5F-L/HE_CRE-14_cropped.png]

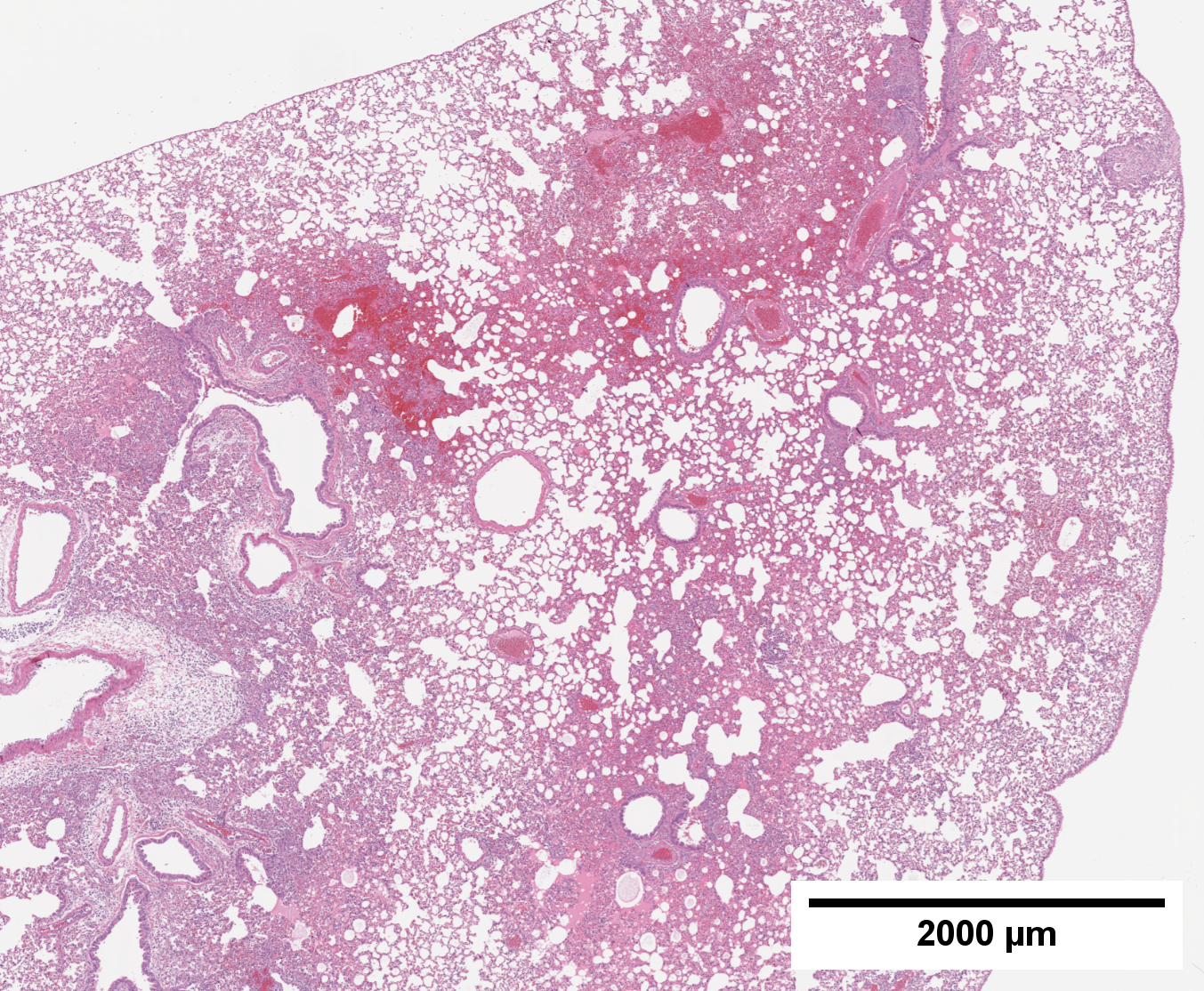

Supplement: Supplementary file 10 — Source data Fig. 5 [file 44321_2024_188_MOESM10_ESM.zip › Figure 5/5F-L/HE_infected.vehicle_cropped.png]

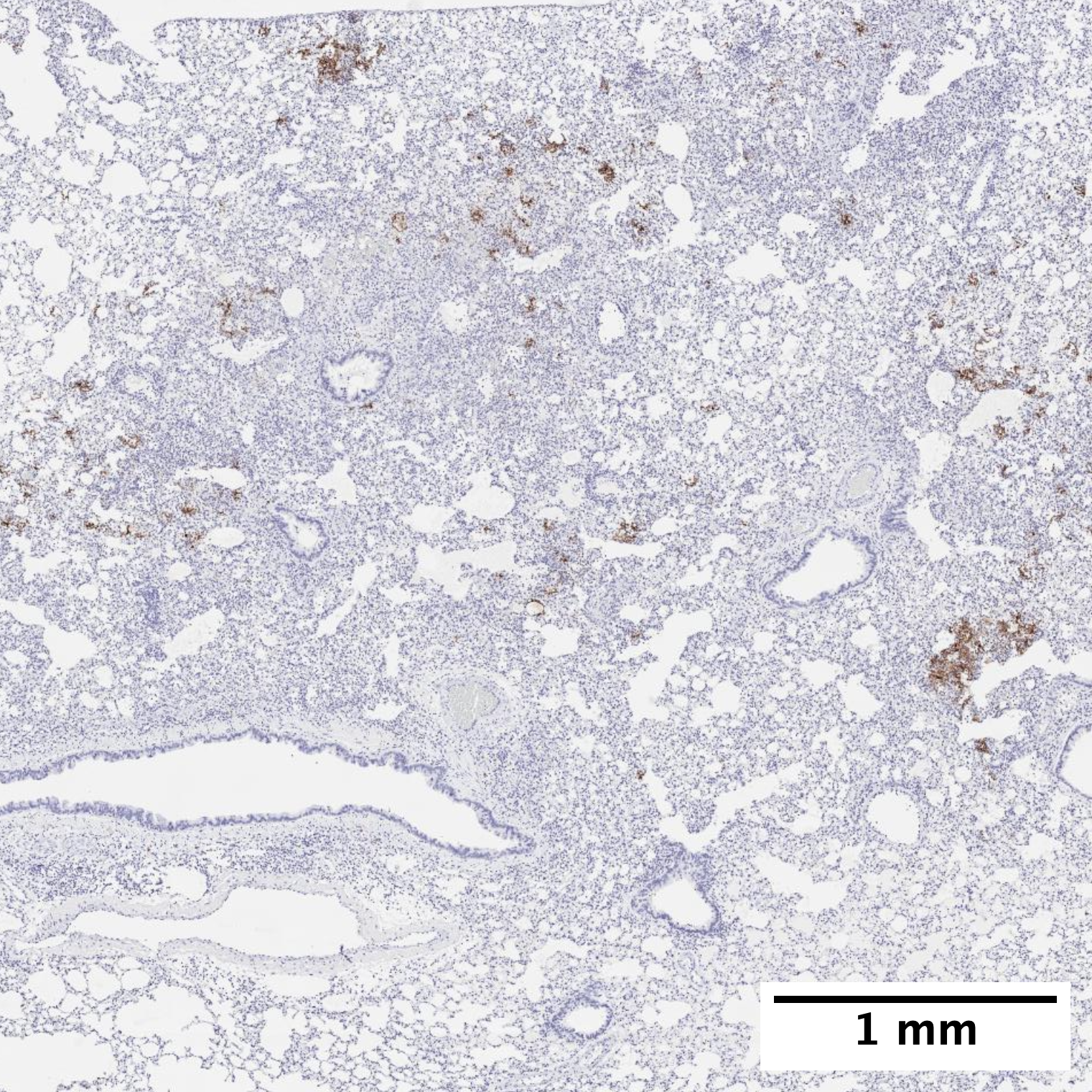

Supplement: Supplementary file 10 — Source data Fig. 5 [file 44321_2024_188_MOESM10_ESM.zip › Figure 5/5E/inf_veh_x2.png]

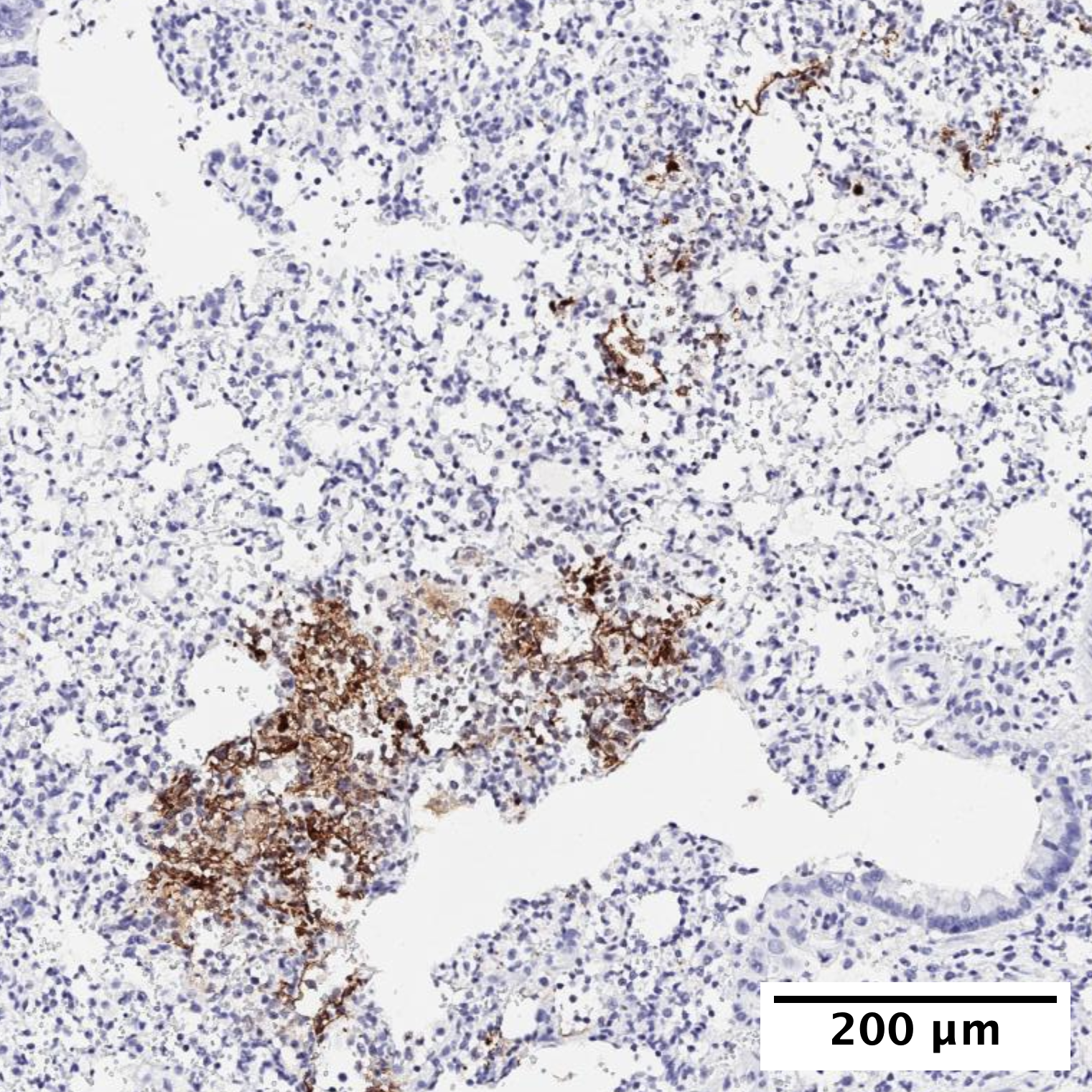

Supplement: Supplementary file 10 — Source data Fig. 5 [file 44321_2024_188_MOESM10_ESM.zip › Figure 5/5E/inf_veh_x10.png]

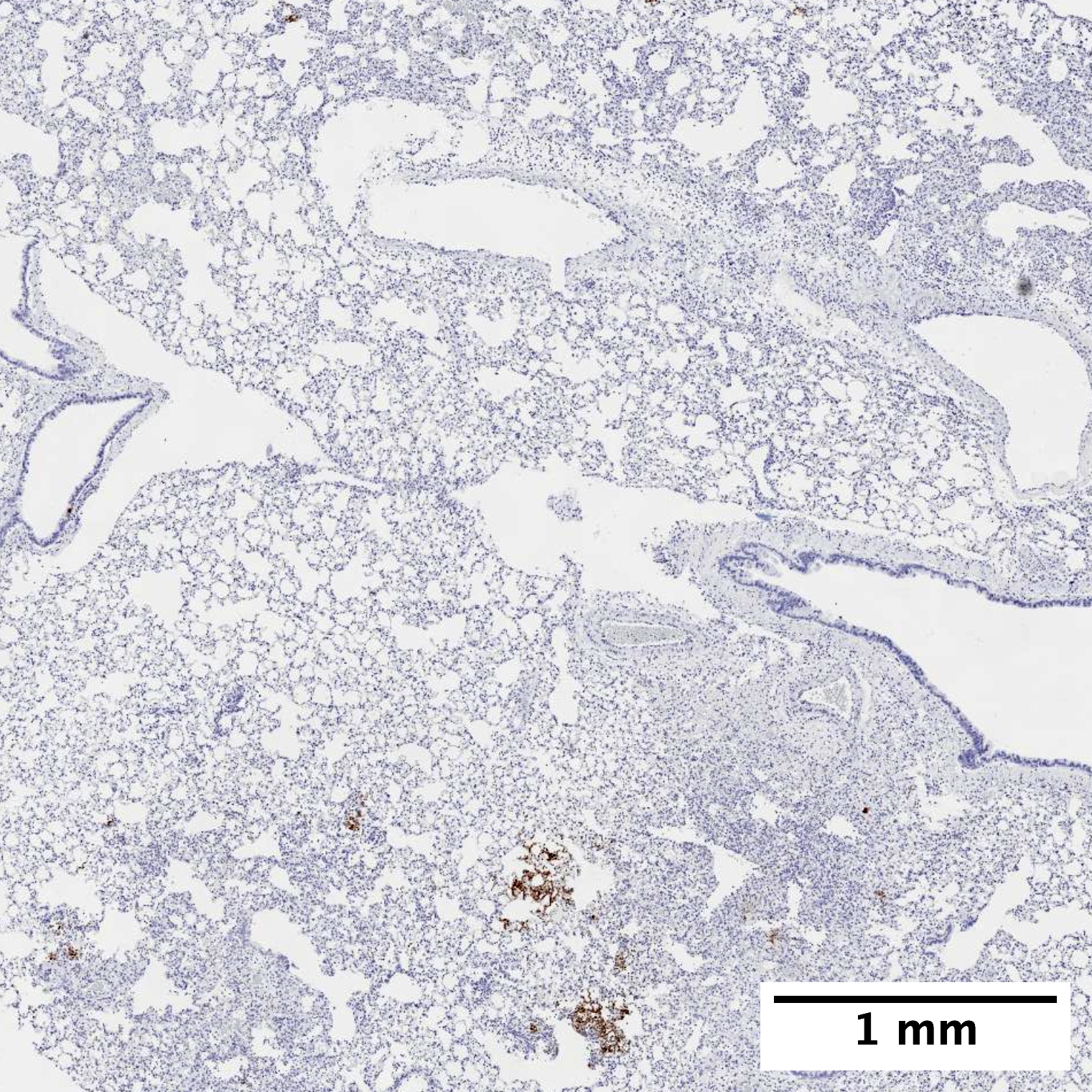

Supplement: Supplementary file 10 — Source data Fig. 5 [file 44321_2024_188_MOESM10_ESM.zip › Figure 5/5E/inf_CRE14_x2.png]

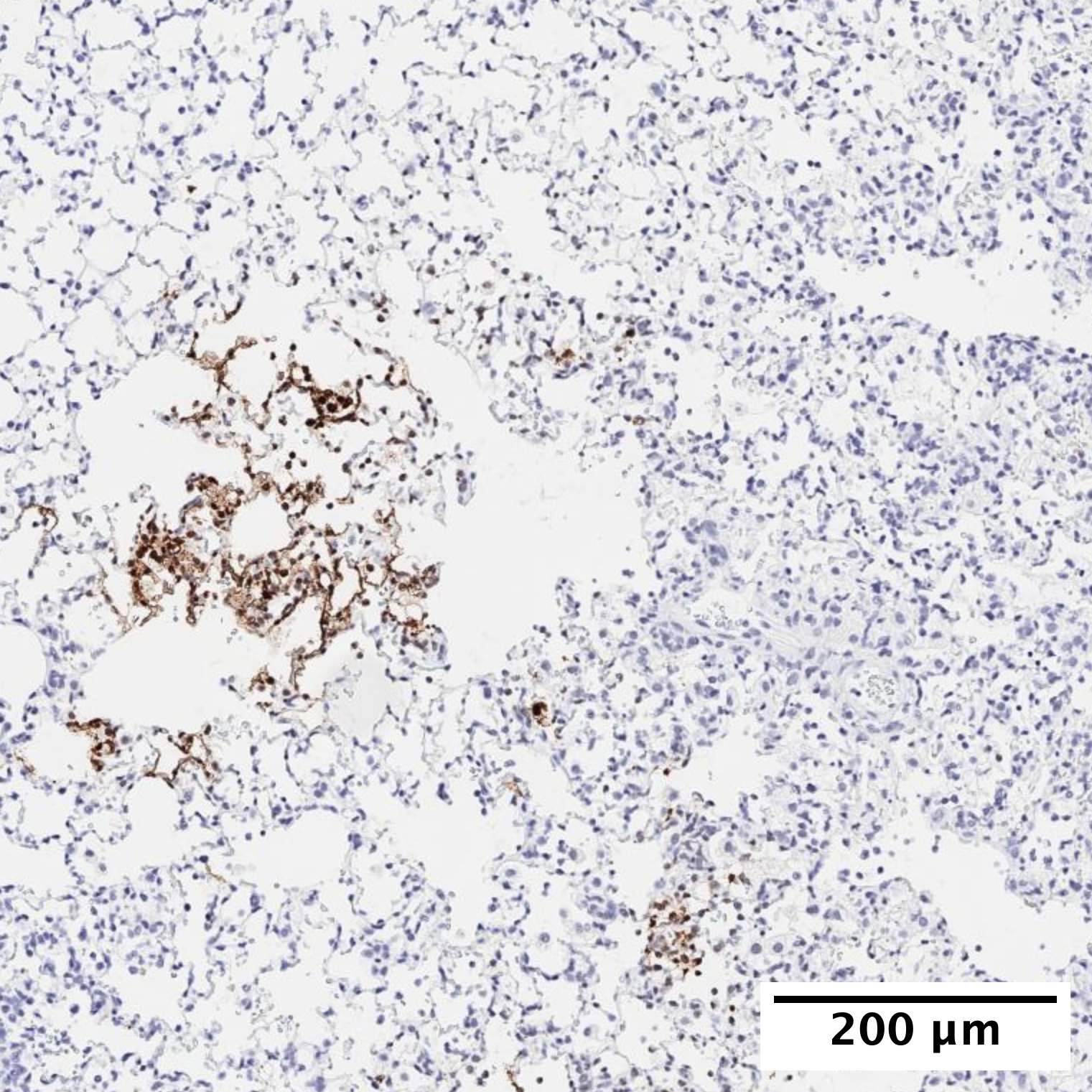

Supplement: Supplementary file 10 — Source data Fig. 5 [file 44321_2024_188_MOESM10_ESM.zip › Figure 5/5E/inf_CRE14_x10.png]

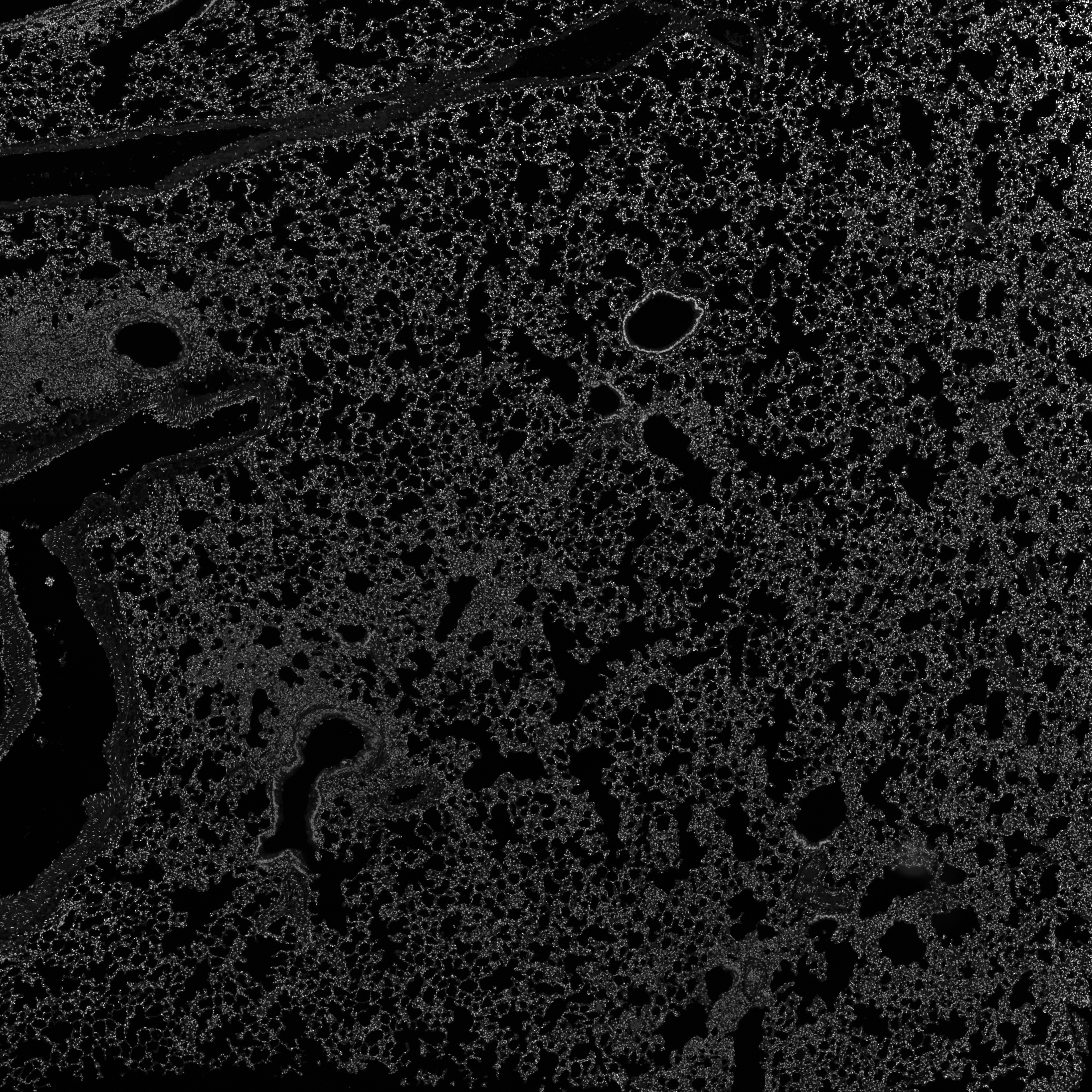

Supplement: Supplementary file 10 — Source data Fig. 5 [file 44321_2024_188_MOESM10_ESM.zip › Figure 5/5C-D/nucleocapsid_IF_CRE-14.tif]

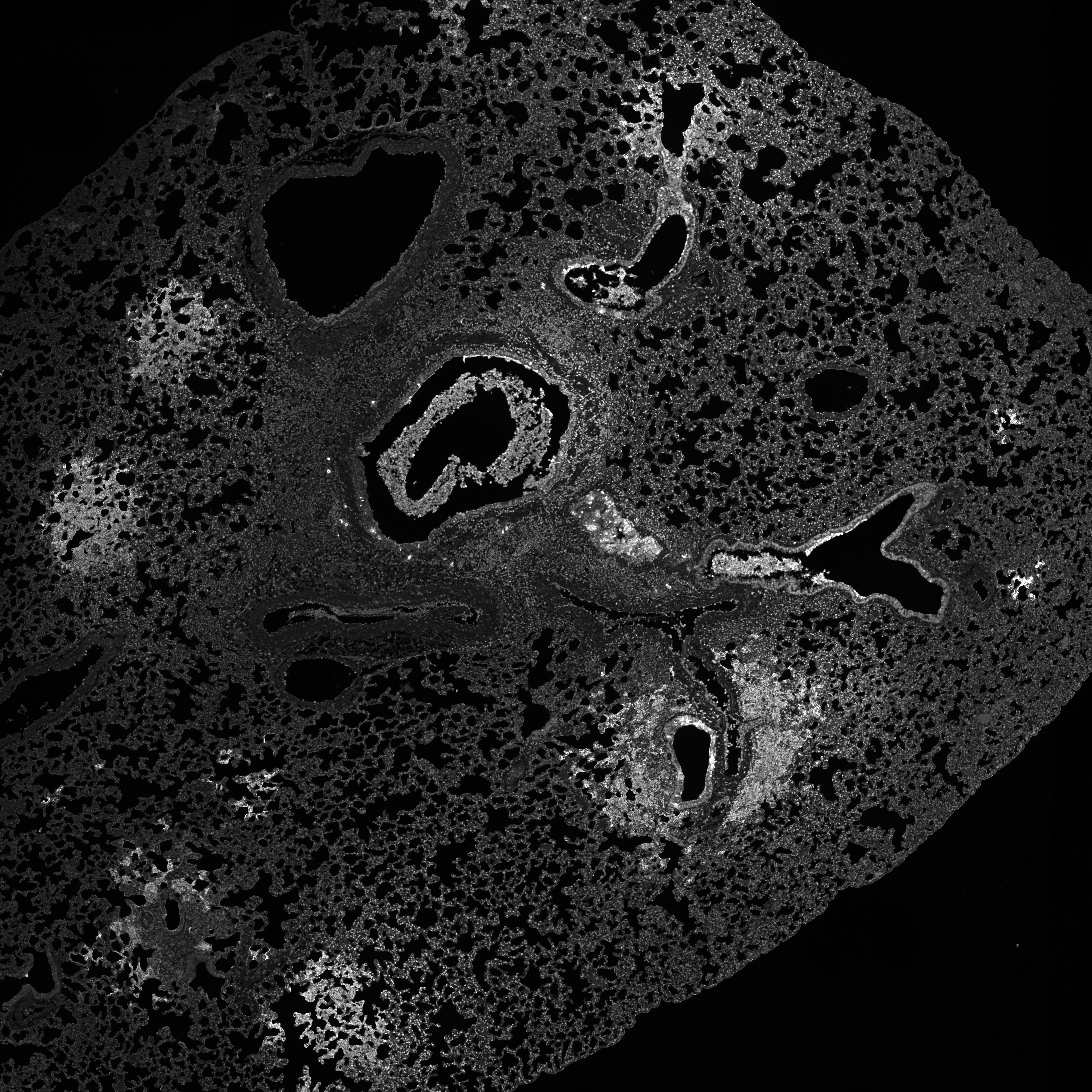

Supplement: Supplementary file 10 — Source data Fig. 5 [file 44321_2024_188_MOESM10_ESM.zip › Figure 5/5C-D/nucleocapsid_IF_Vehicle.tif]

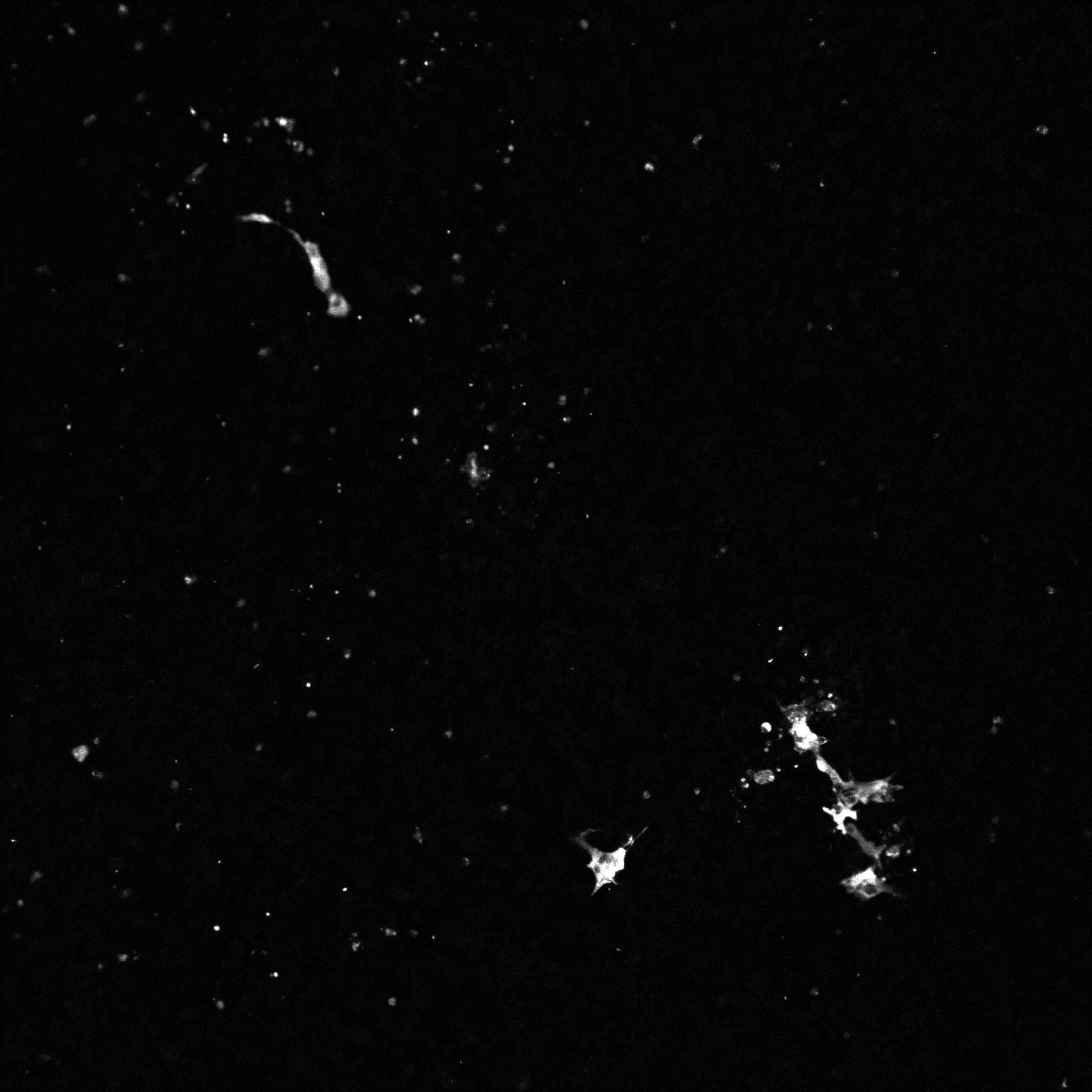

Supplement: Supplementary file 11 — Figure EV1-3 Source Data [file 44321_2024_188_MOESM11_ESM.zip › Expanded View 3/EV.3G-H/dsRNA_BMS_infected.tif]

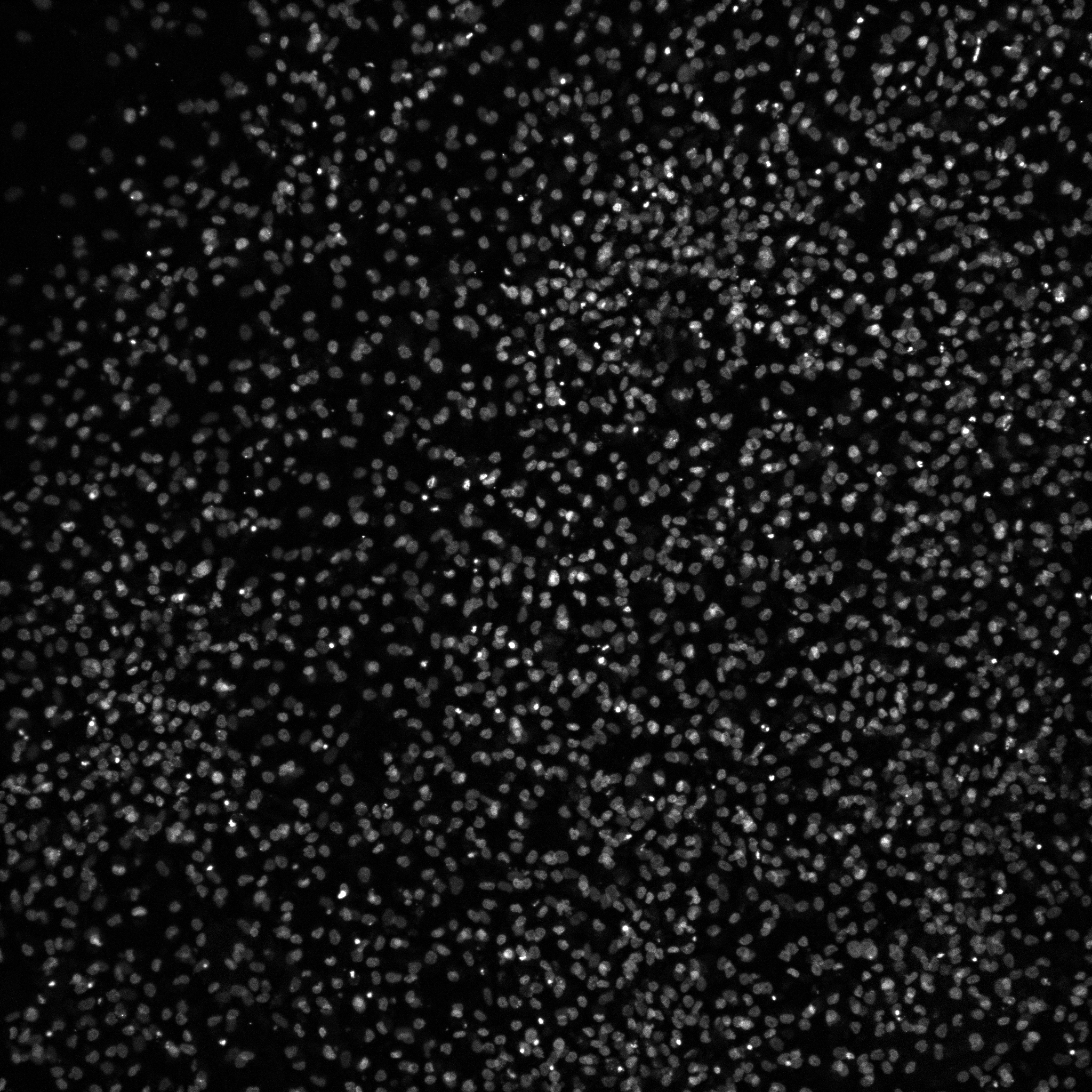

Supplement: Supplementary file 11 — Figure EV1-3 Source Data [file 44321_2024_188_MOESM11_ESM.zip › Expanded View 3/EV.3G-H/DAPI_BMS_infected.tif]

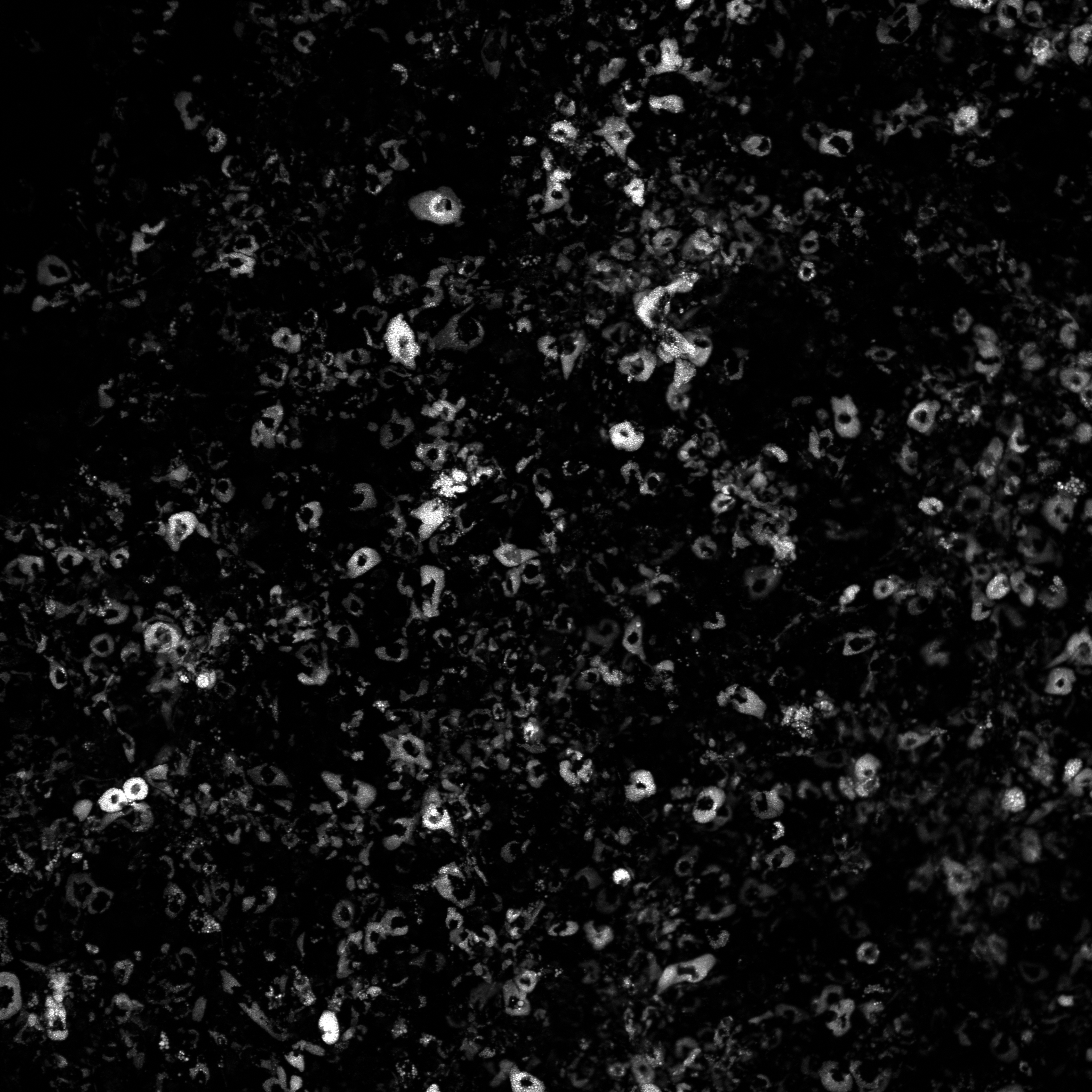

Supplement: Supplementary file 11 — Figure EV1-3 Source Data [file 44321_2024_188_MOESM11_ESM.zip › Expanded View 3/EV.3G-H/LDs_BMS_infected.tif]

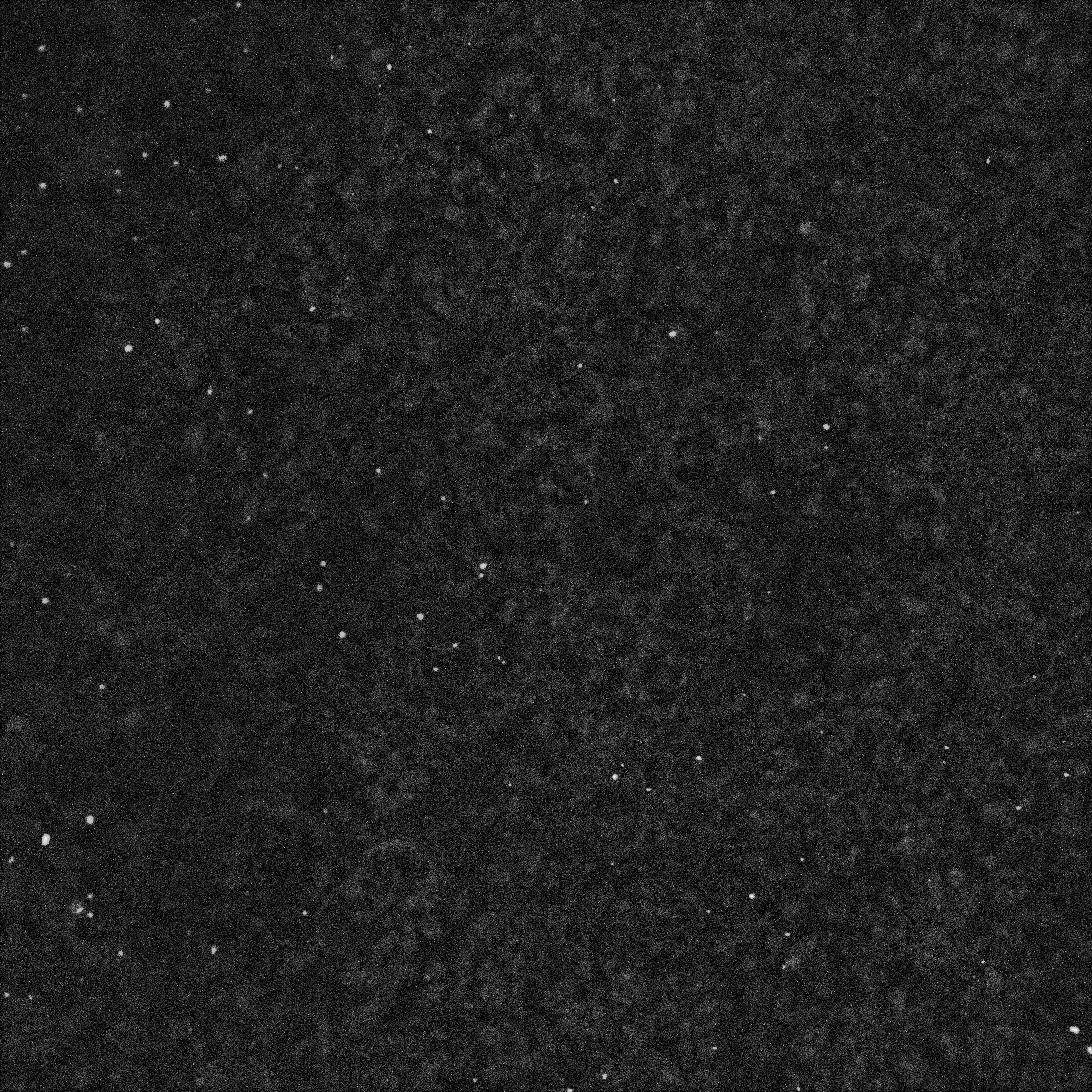

Supplement: Supplementary file 11 — Figure EV1-3 Source Data [file 44321_2024_188_MOESM11_ESM.zip › Expanded View 3/EV.3G-H/dsRNA_BMS_uninfected.tif]

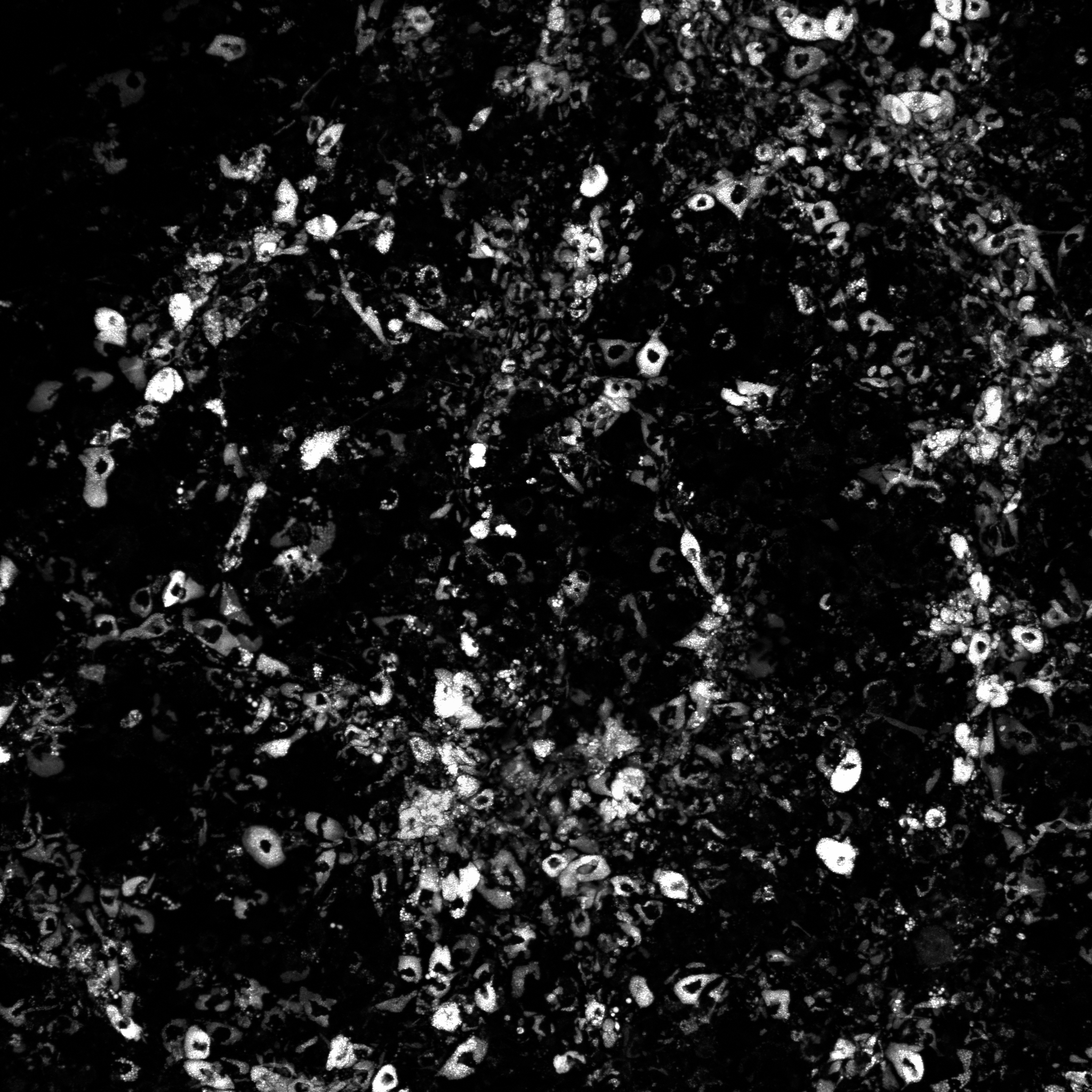

Supplement: Supplementary file 11 — Figure EV1-3 Source Data [file 44321_2024_188_MOESM11_ESM.zip › Expanded View 3/EV.3G-H/LDs_BMS_uninfected.tif]

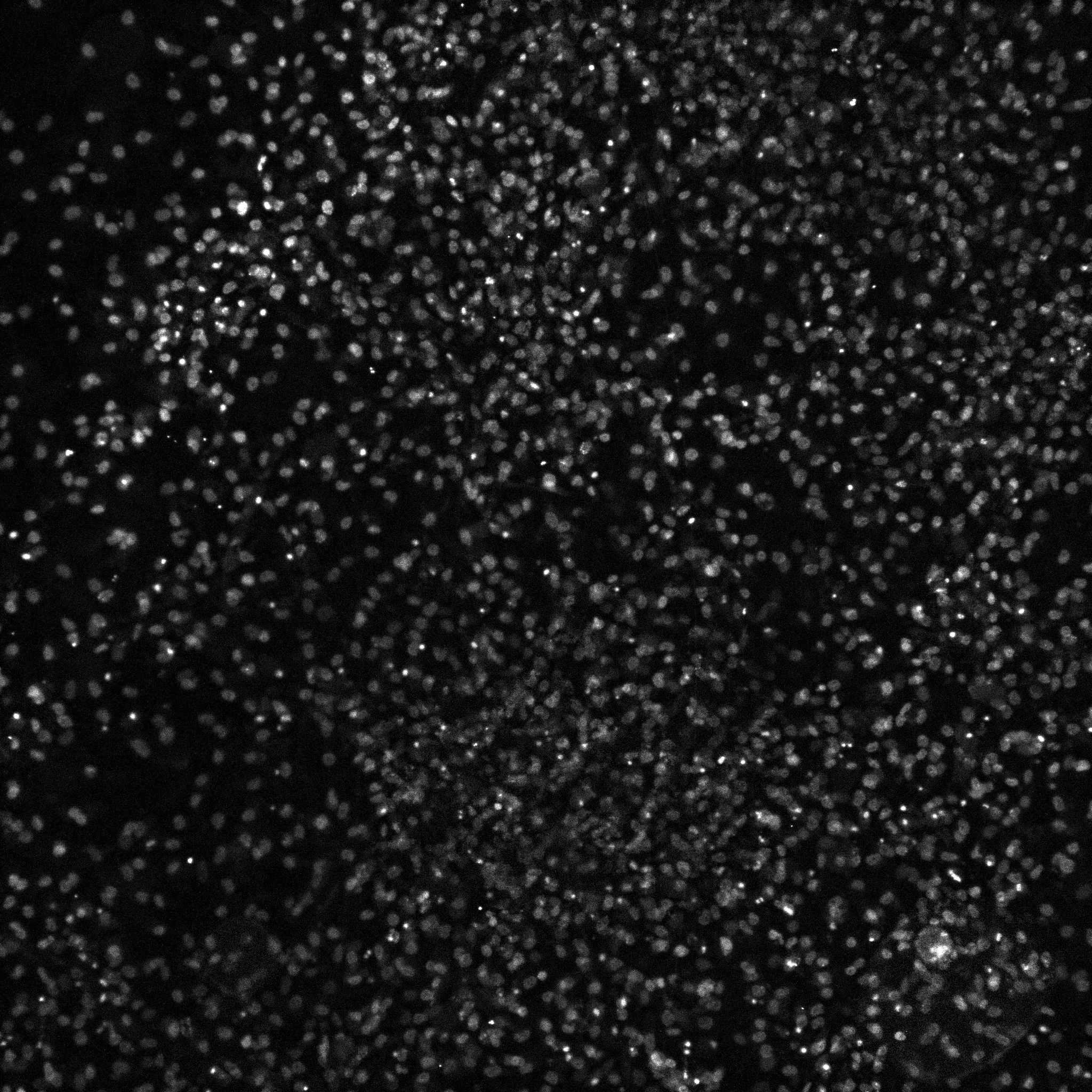

Supplement: Supplementary file 11 — Figure EV1-3 Source Data [file 44321_2024_188_MOESM11_ESM.zip › Expanded View 3/EV.3G-H/DAPI_BMS_uninfected.tif]

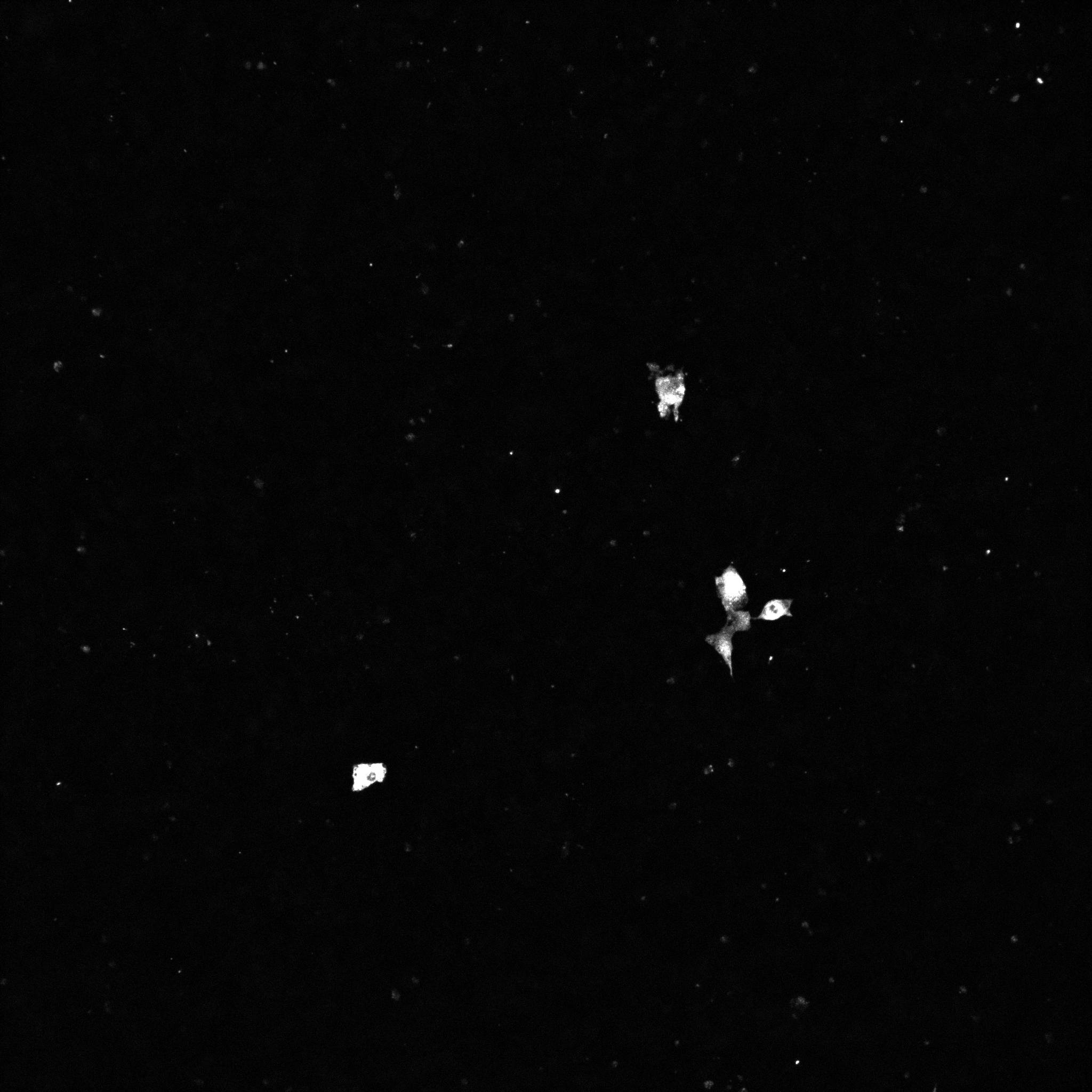

Supplement: Supplementary file 11 — Figure EV1-3 Source Data [file 44321_2024_188_MOESM11_ESM.zip › Expanded View 3/EV.3G-H/dsRNA_CRE_infected.tif]

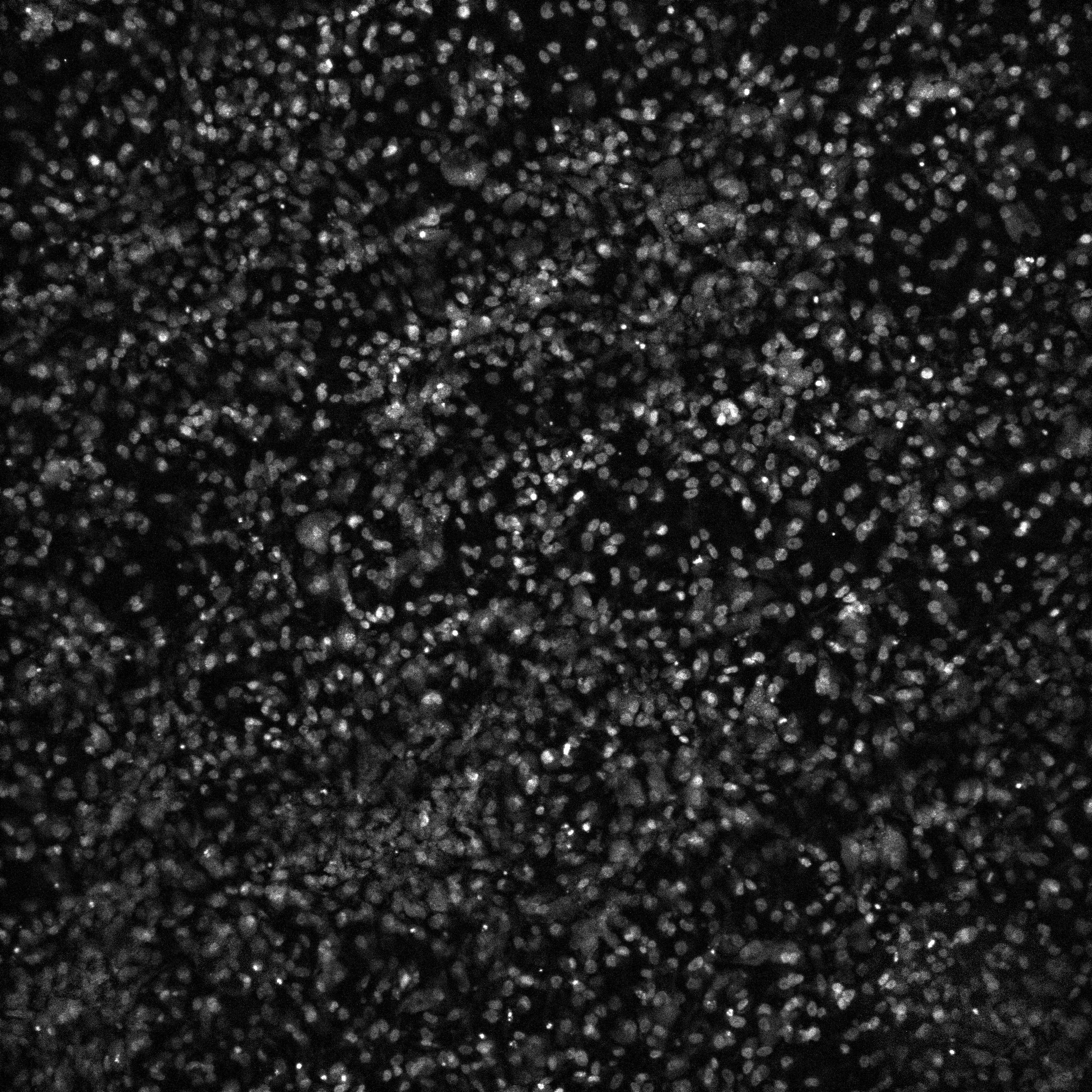

Supplement: Supplementary file 11 — Figure EV1-3 Source Data [file 44321_2024_188_MOESM11_ESM.zip › Expanded View 3/EV.3G-H/DAPI_CRE_infected.tif]

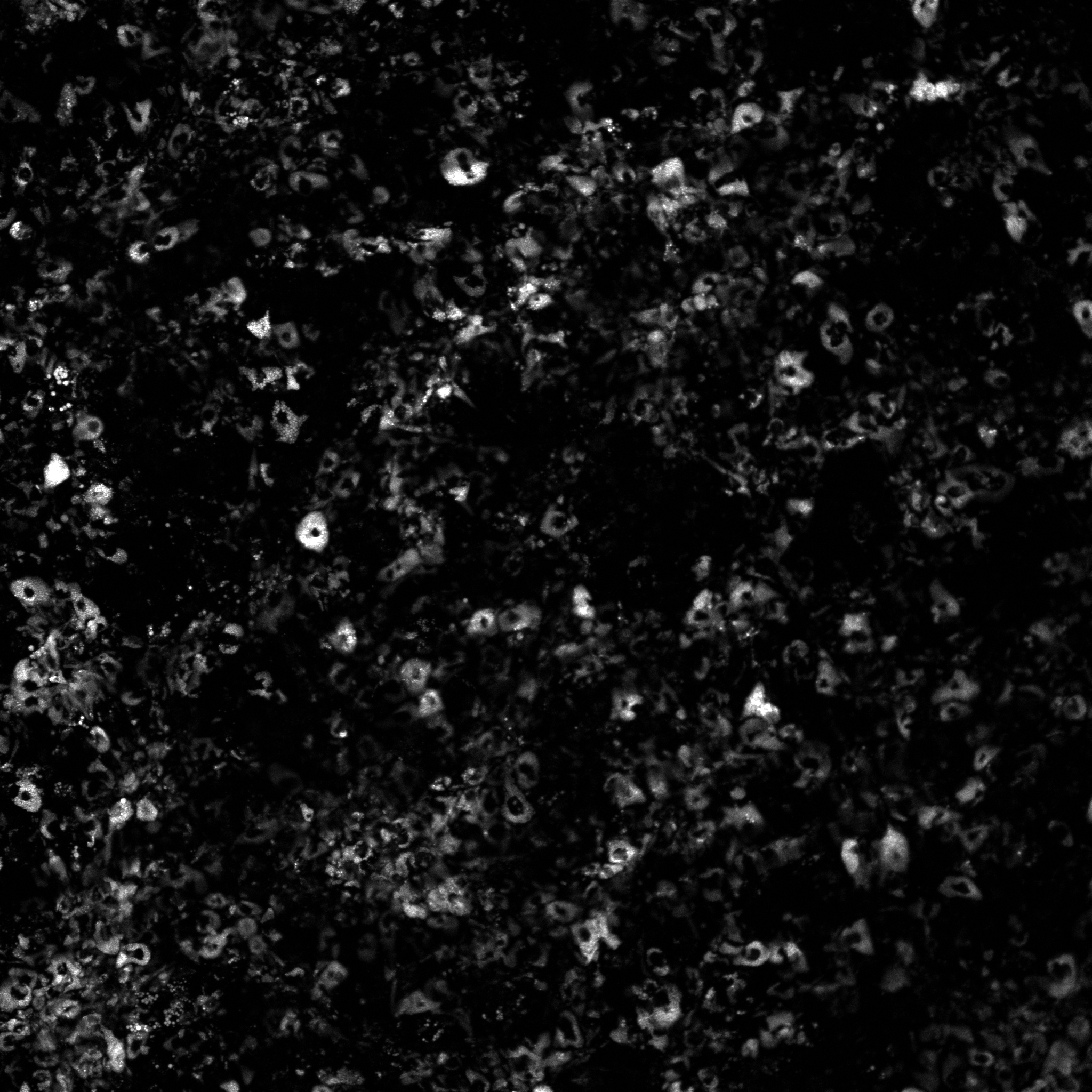

Supplement: Supplementary file 11 — Figure EV1-3 Source Data [file 44321_2024_188_MOESM11_ESM.zip › Expanded View 3/EV.3G-H/LDs_CRE_infected.tif]

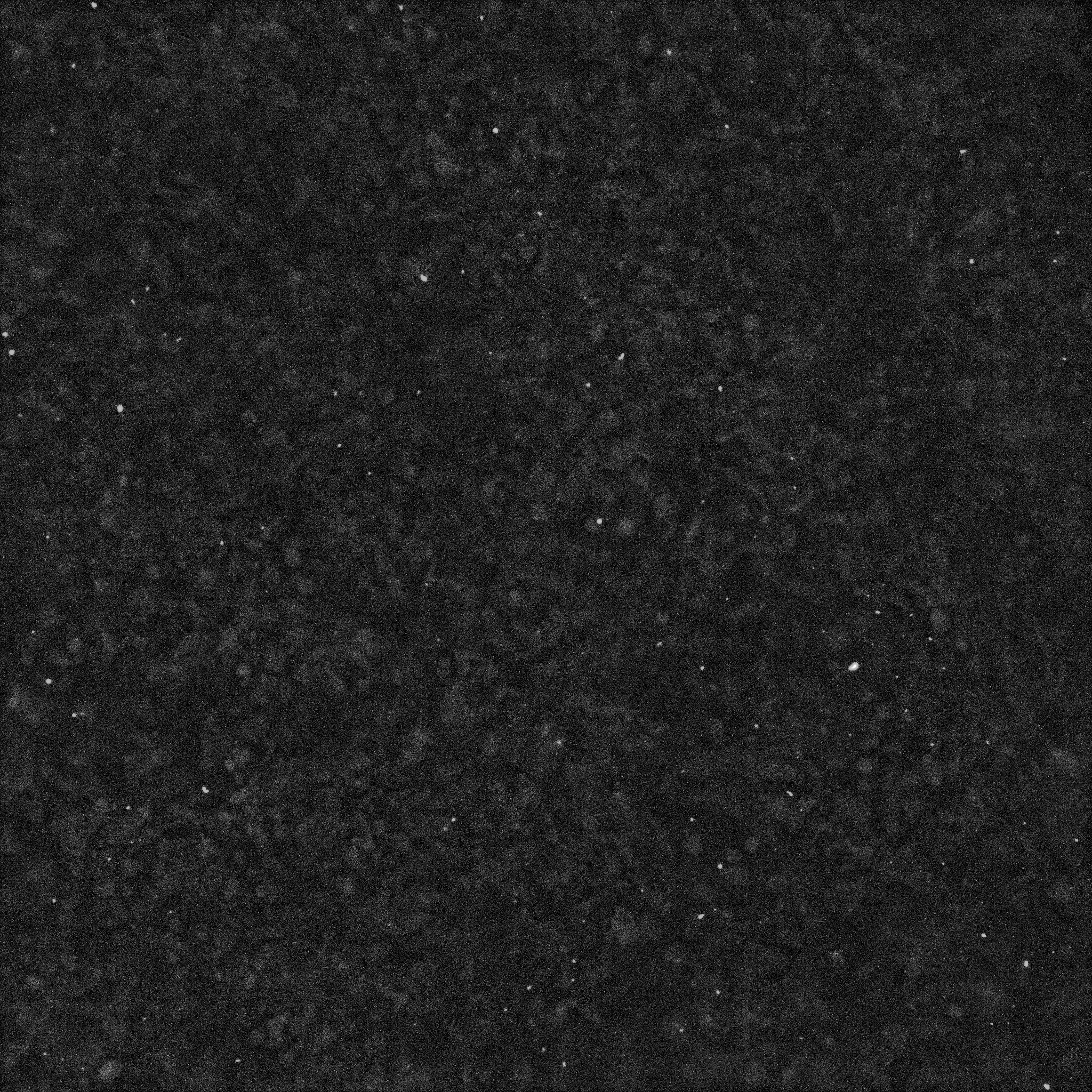

Supplement: Supplementary file 11 — Figure EV1-3 Source Data [file 44321_2024_188_MOESM11_ESM.zip › Expanded View 3/EV.3G-H/dsRNA_CRE_uninfected.tif]

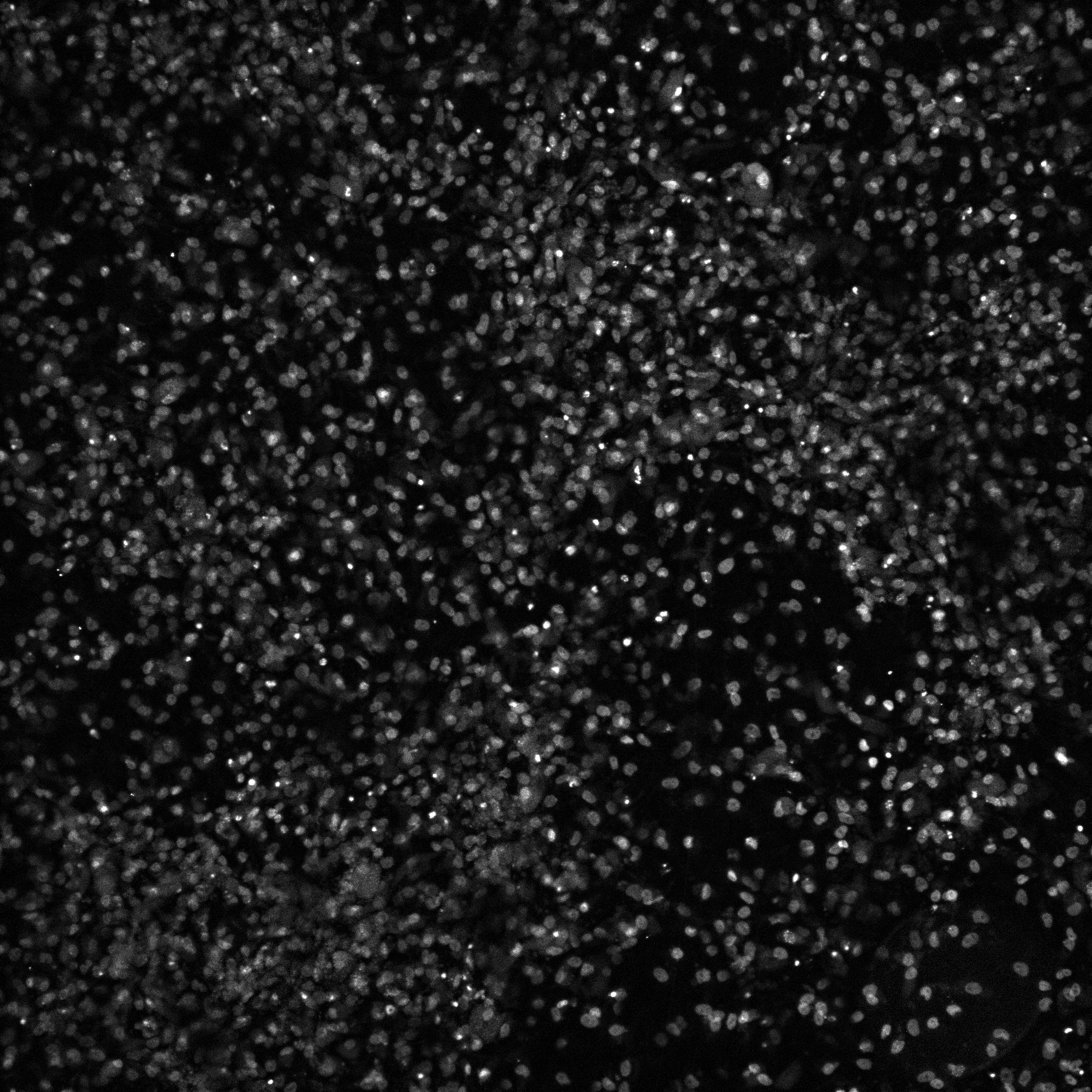

Supplement: Supplementary file 11 — Figure EV1-3 Source Data [file 44321_2024_188_MOESM11_ESM.zip › Expanded View 3/EV.3G-H/DAPI_CRE_uninfected.tif]

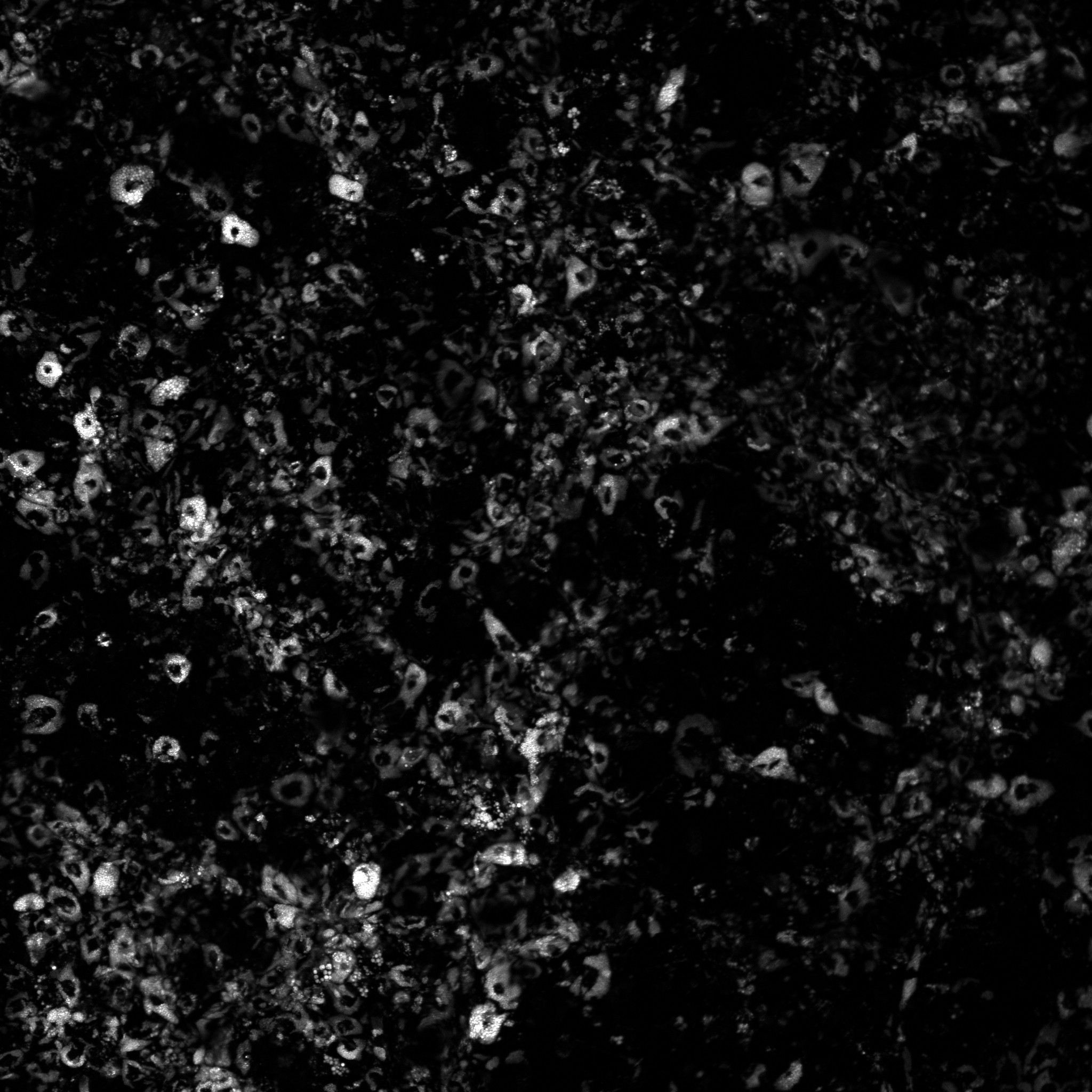

Supplement: Supplementary file 11 — Figure EV1-3 Source Data [file 44321_2024_188_MOESM11_ESM.zip › Expanded View 3/EV.3G-H/LDs_CRE_uninfected.tif]

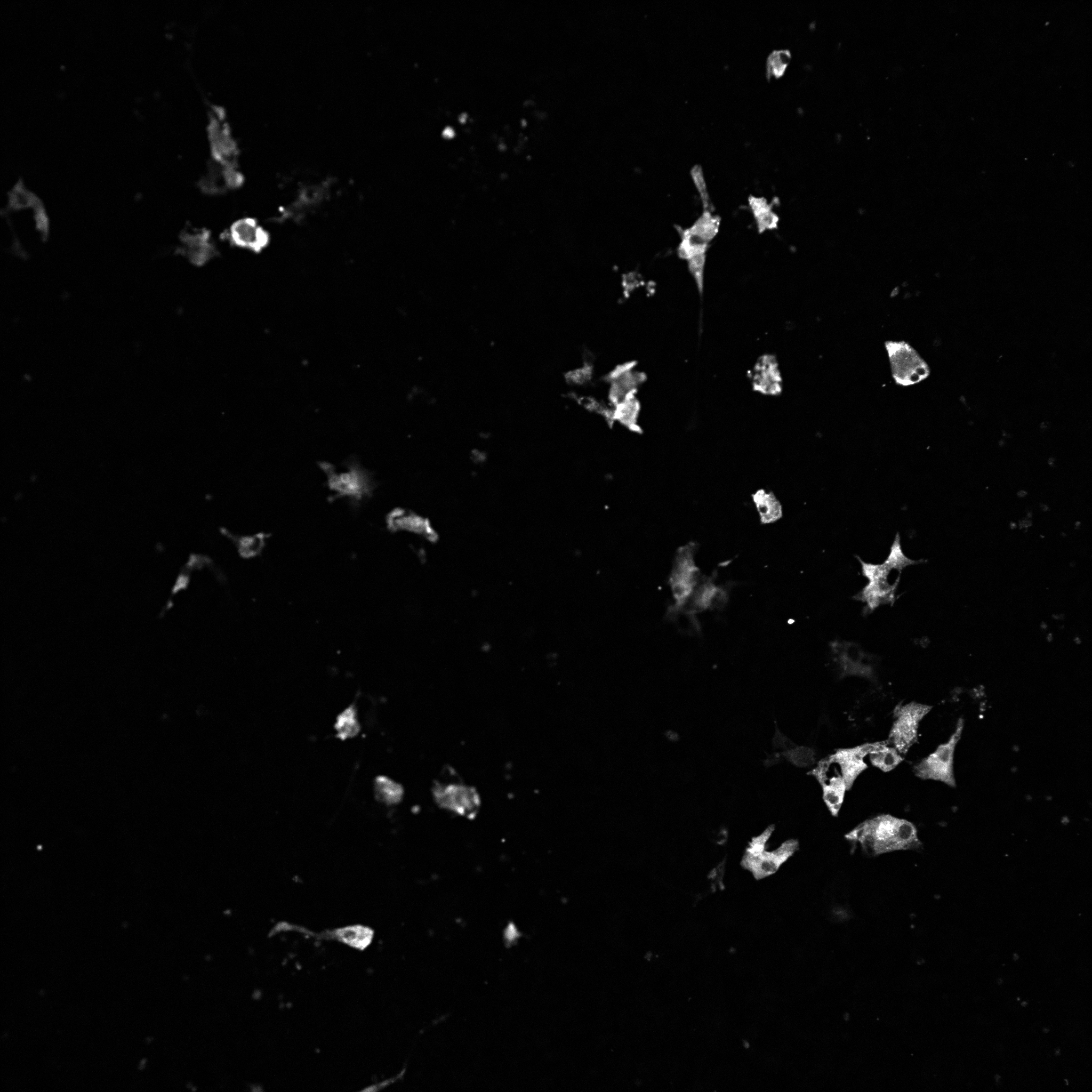

Supplement: Supplementary file 11 — Figure EV1-3 Source Data [file 44321_2024_188_MOESM11_ESM.zip › Expanded View 3/EV.3G-H/dsRNA_DMSO_infected.tif]

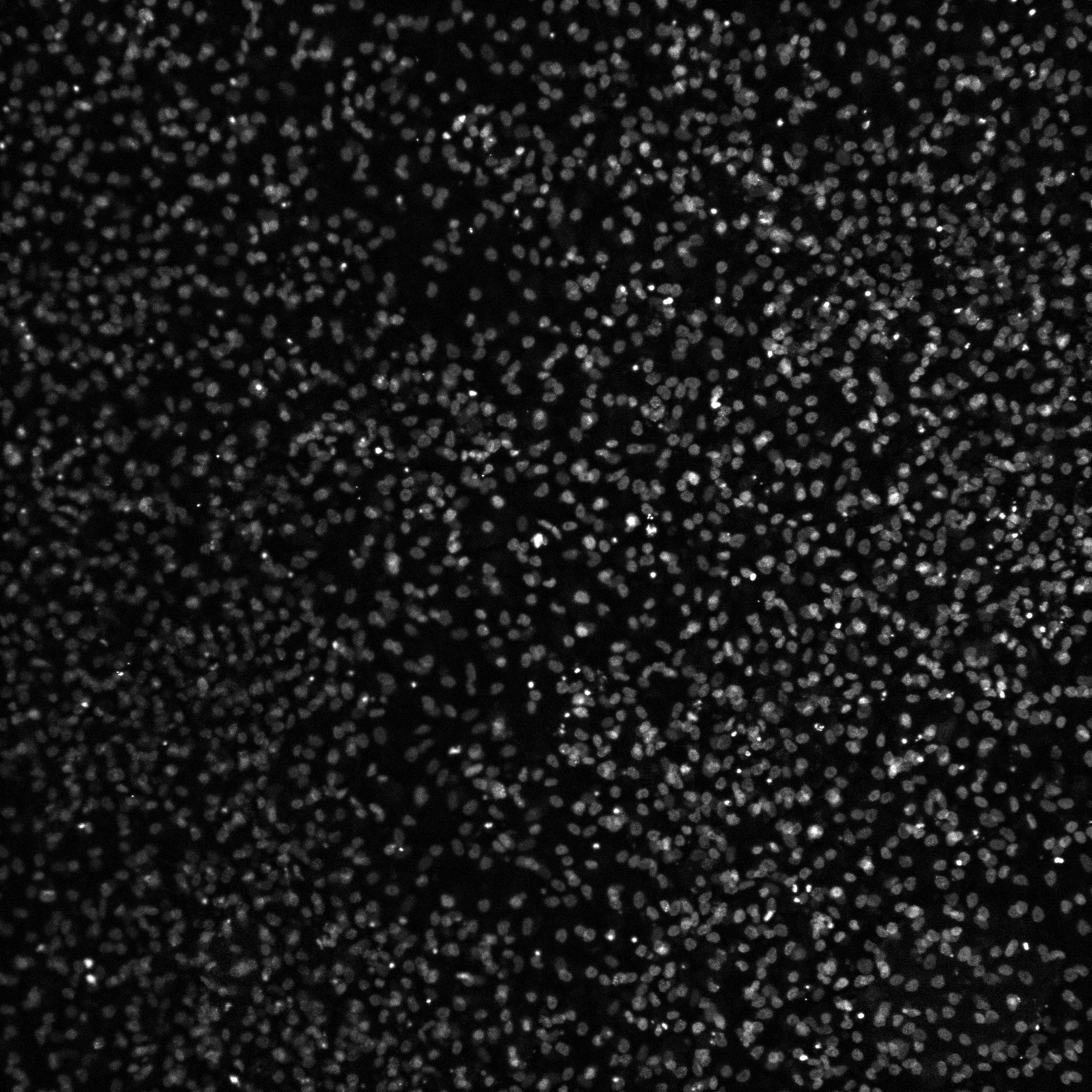

Supplement: Supplementary file 11 — Figure EV1-3 Source Data [file 44321_2024_188_MOESM11_ESM.zip › Expanded View 3/EV.3G-H/DAPI_DMSO_infected.tif]

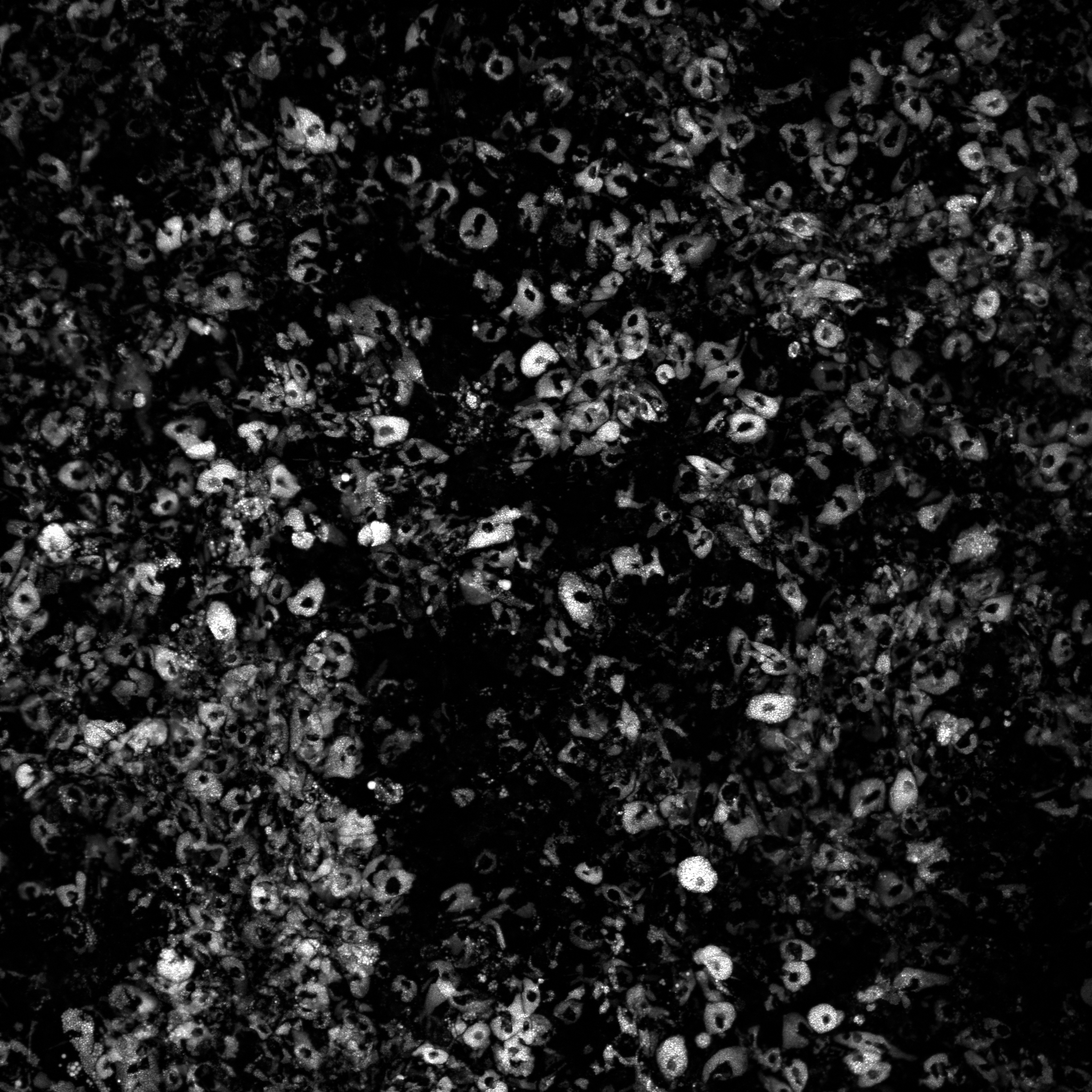

Supplement: Supplementary file 11 — Figure EV1-3 Source Data [file 44321_2024_188_MOESM11_ESM.zip › Expanded View 3/EV.3G-H/LDs_DMSO_infected.tif]

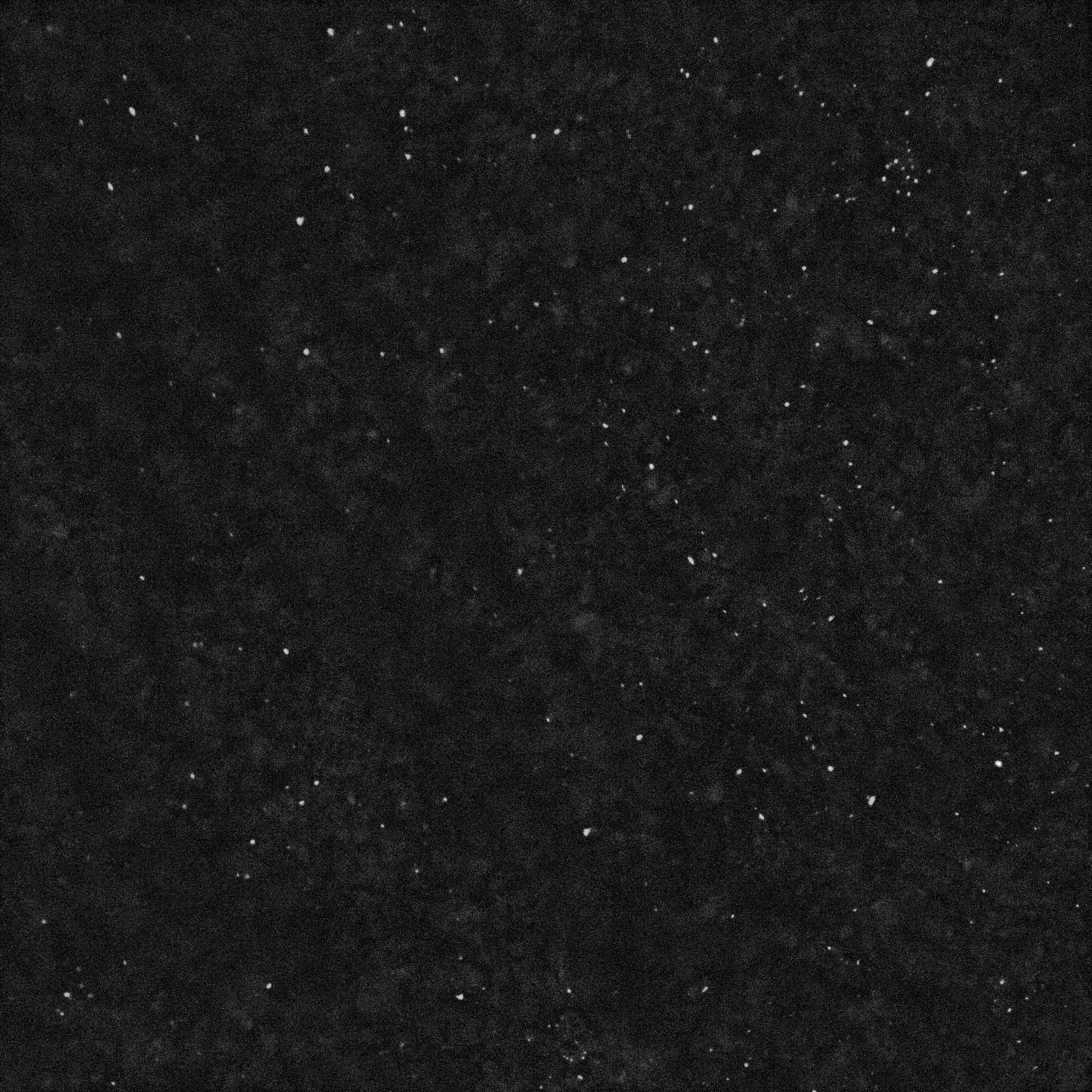

Supplement: Supplementary file 11 — Figure EV1-3 Source Data [file 44321_2024_188_MOESM11_ESM.zip › Expanded View 3/EV.3G-H/dsRNA_DMSO_uninfected.tif]

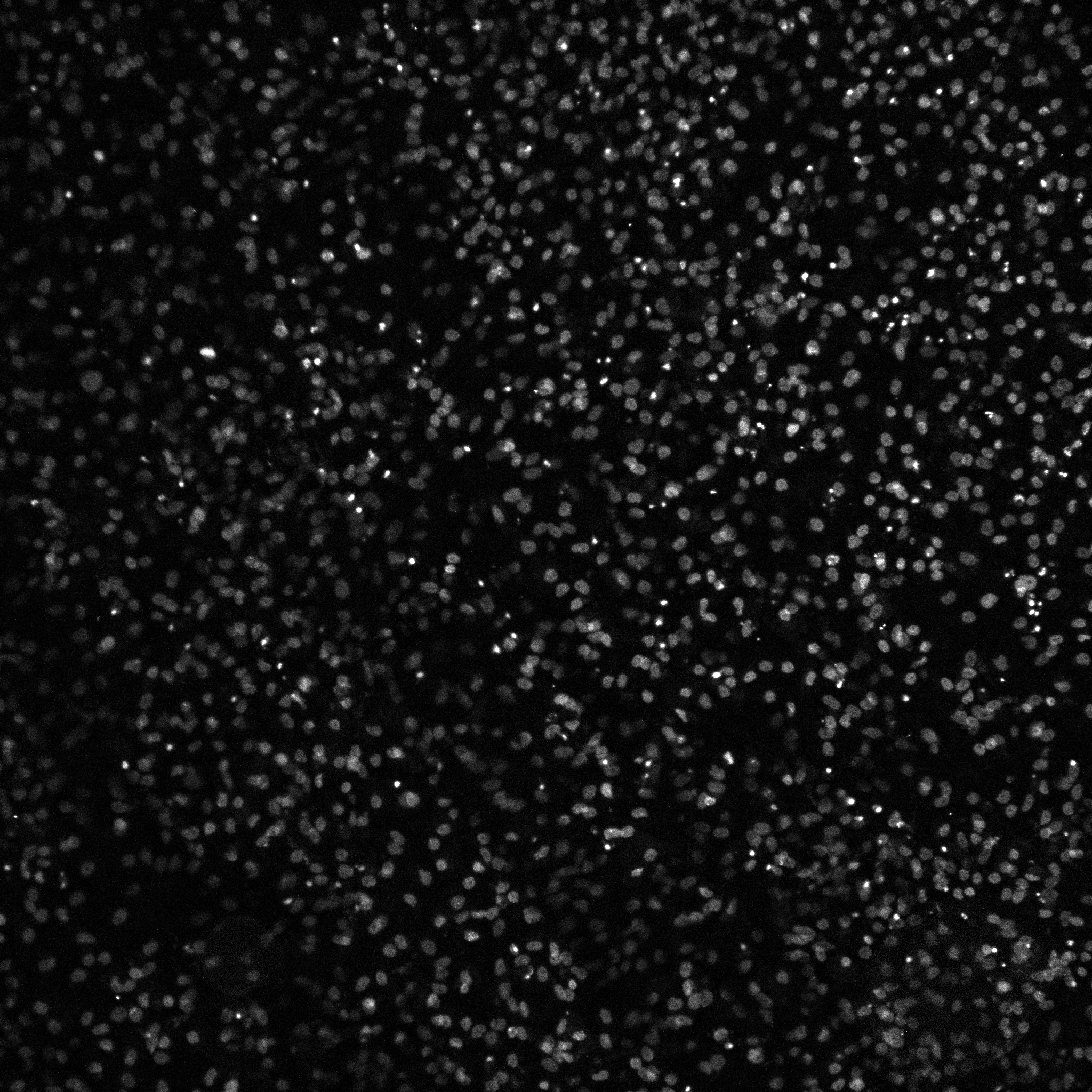

Supplement: Supplementary file 11 — Figure EV1-3 Source Data [file 44321_2024_188_MOESM11_ESM.zip › Expanded View 3/EV.3G-H/DAPI_DMSO_uninfected.tif]

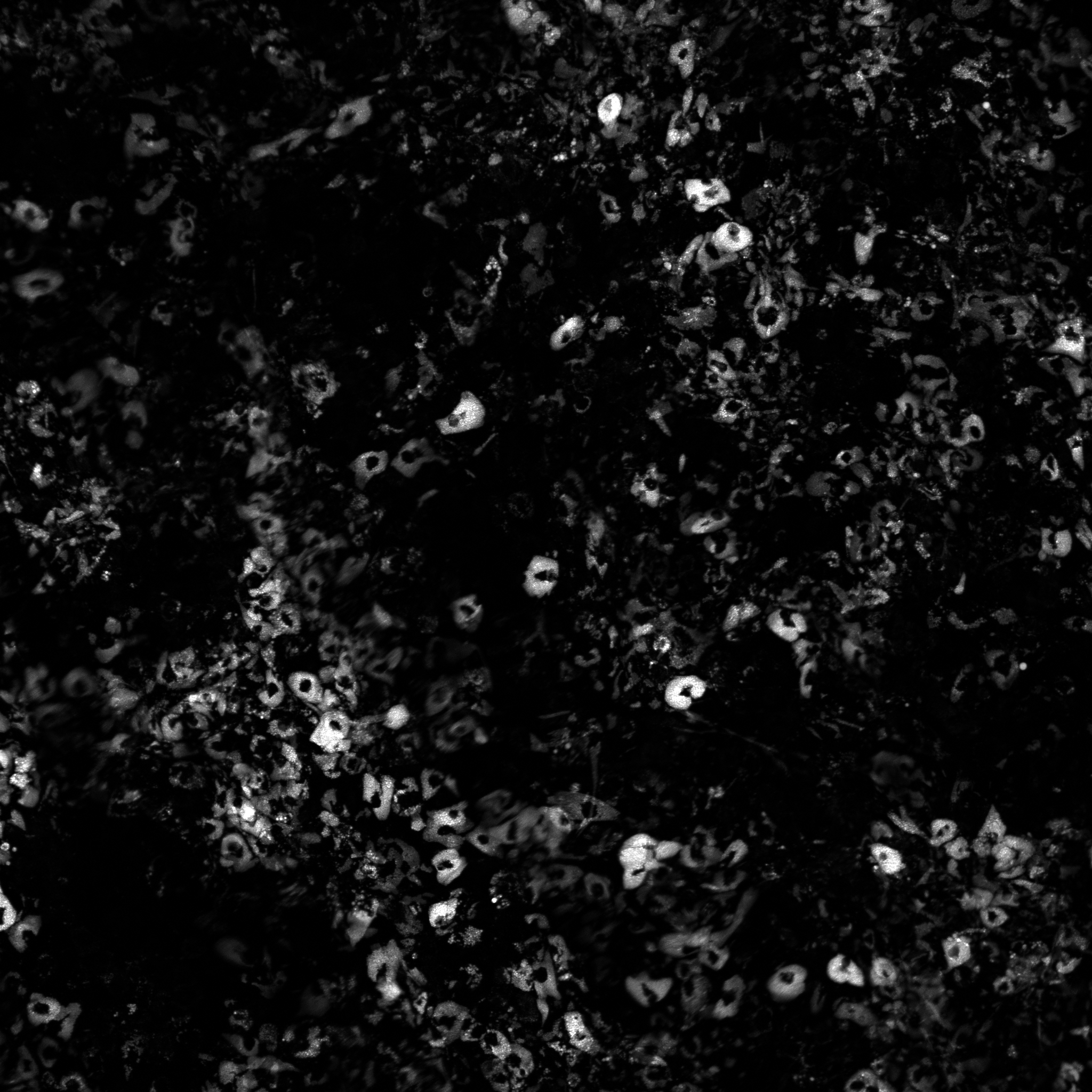

Supplement: Supplementary file 11 — Figure EV1-3 Source Data [file 44321_2024_188_MOESM11_ESM.zip › Expanded View 3/EV.3G-H/LDs_DMSO_uninfected.tif]

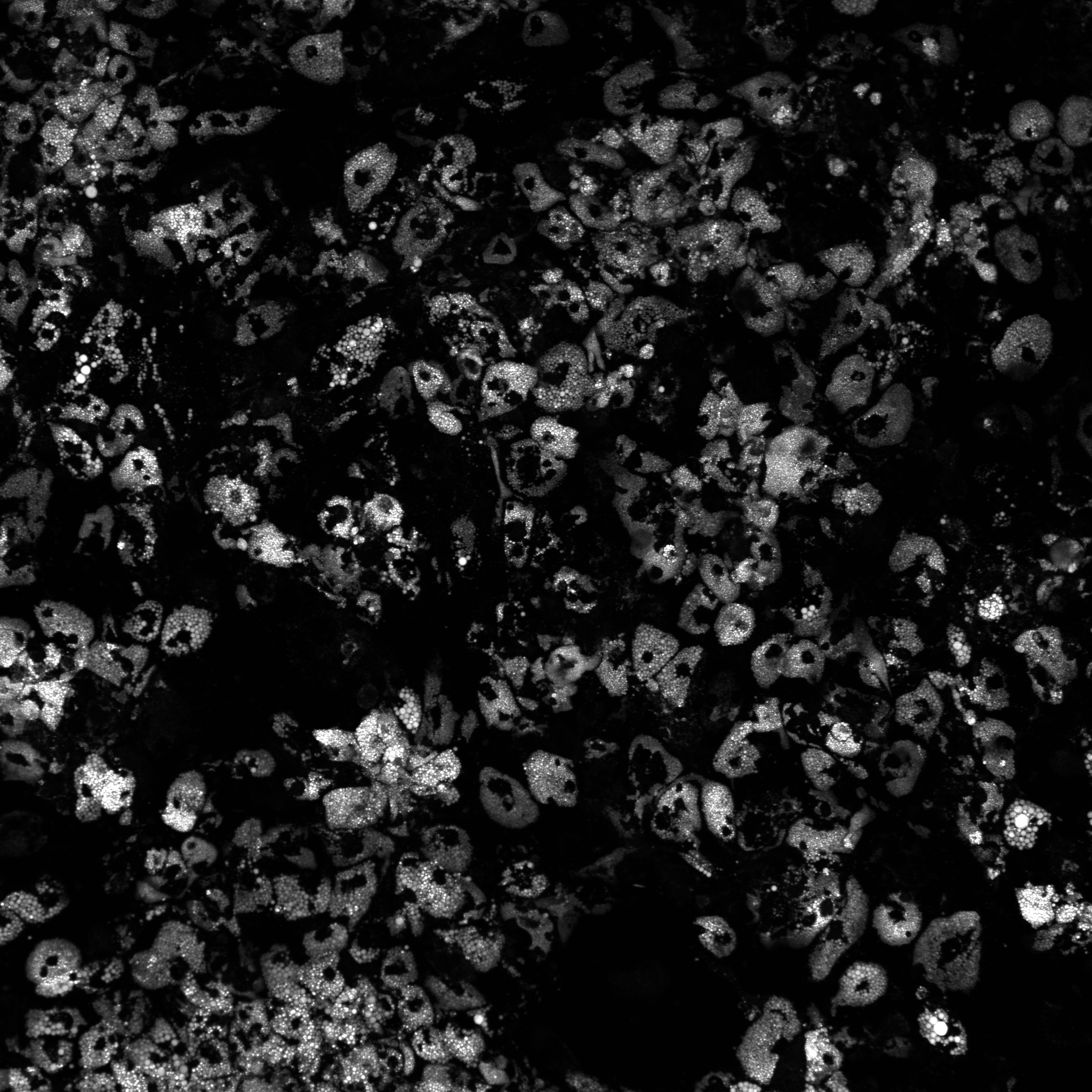

Supplement: Supplementary file 11 — Figure EV1-3 Source Data [file 44321_2024_188_MOESM11_ESM.zip › Expanded View 3/EV.3I/LDs_FABP4-KO.tif]

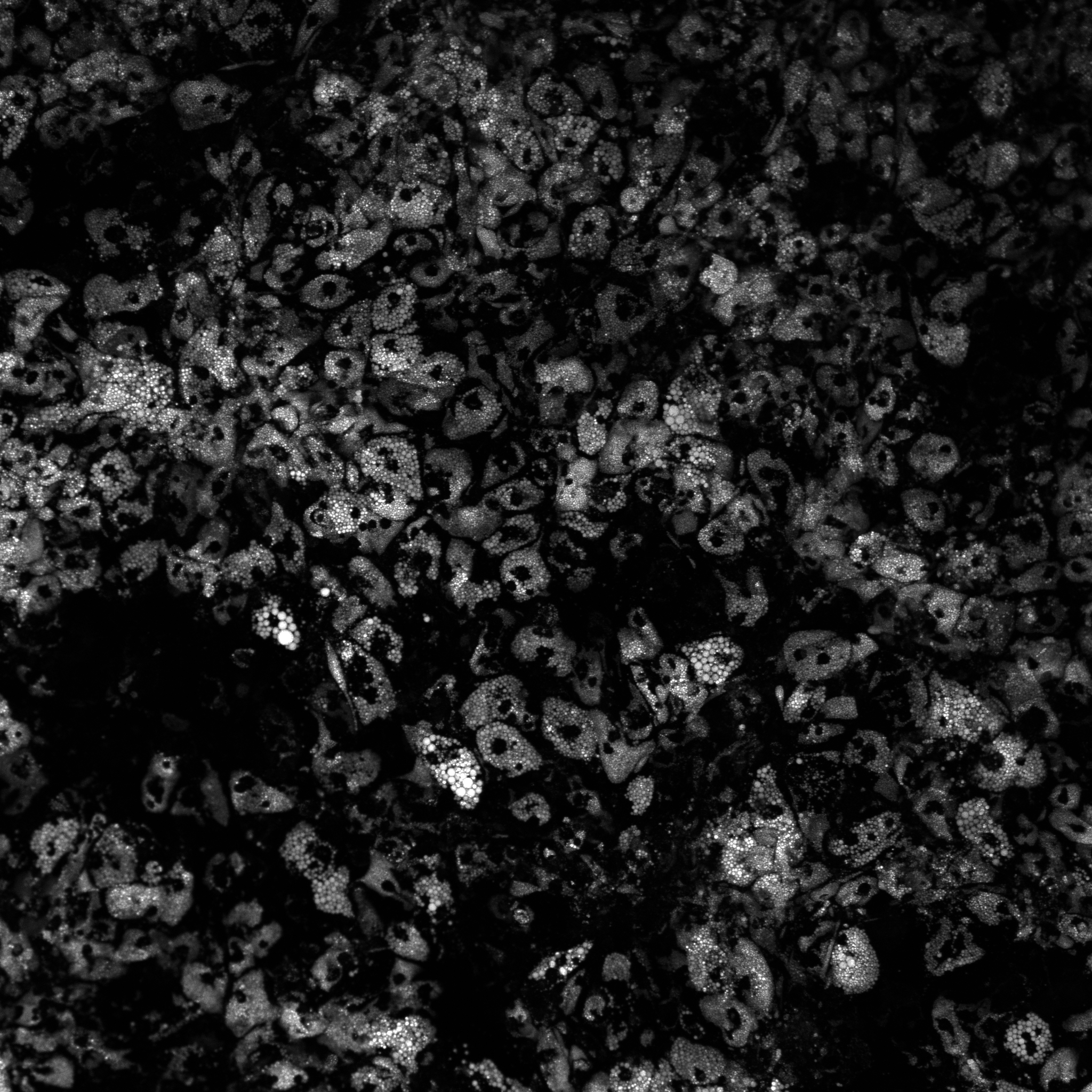

Supplement: Supplementary file 11 — Figure EV1-3 Source Data [file 44321_2024_188_MOESM11_ESM.zip › Expanded View 3/EV.3I/LDs_WT.tif]

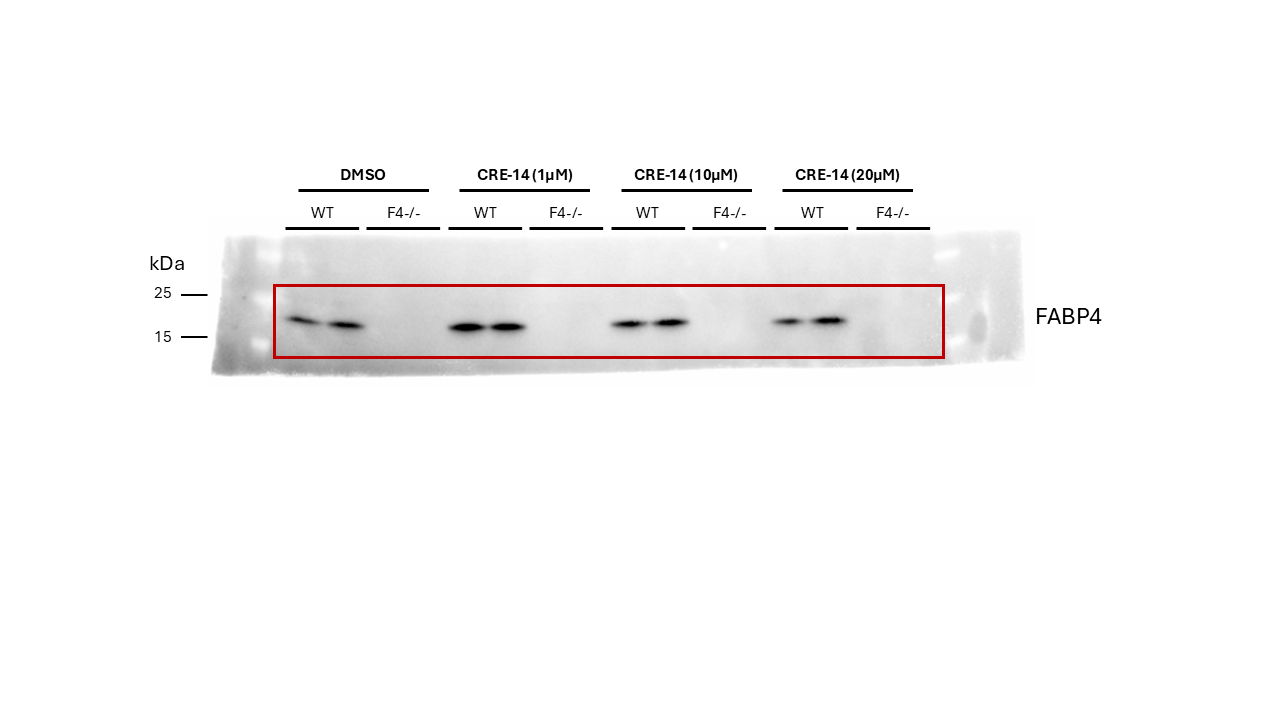

Supplement: Supplementary file 11 — Figure EV1-3 Source Data [file 44321_2024_188_MOESM11_ESM.zip › Expanded View 3/EV.3K-M/CRE-14_FABP4.tif]

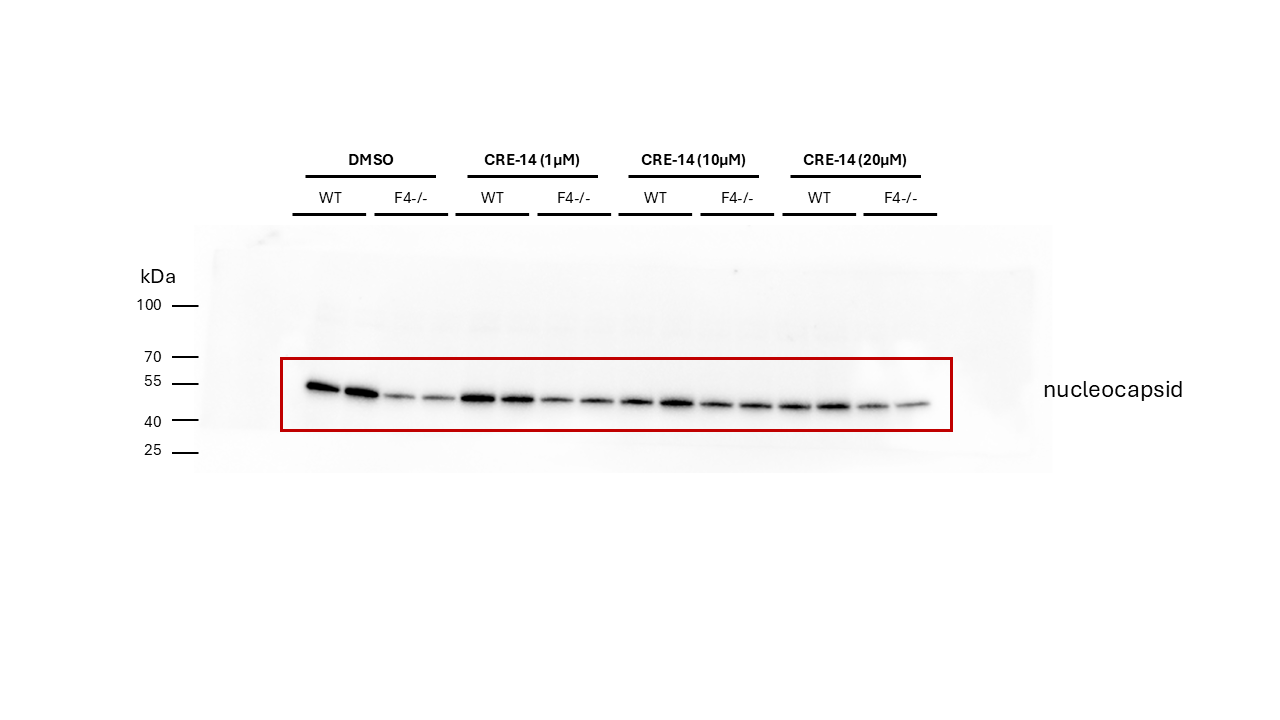

Supplement: Supplementary file 11 — Figure EV1-3 Source Data [file 44321_2024_188_MOESM11_ESM.zip › Expanded View 3/EV.3K-M/CRE-14_nucleocapsid.tif]

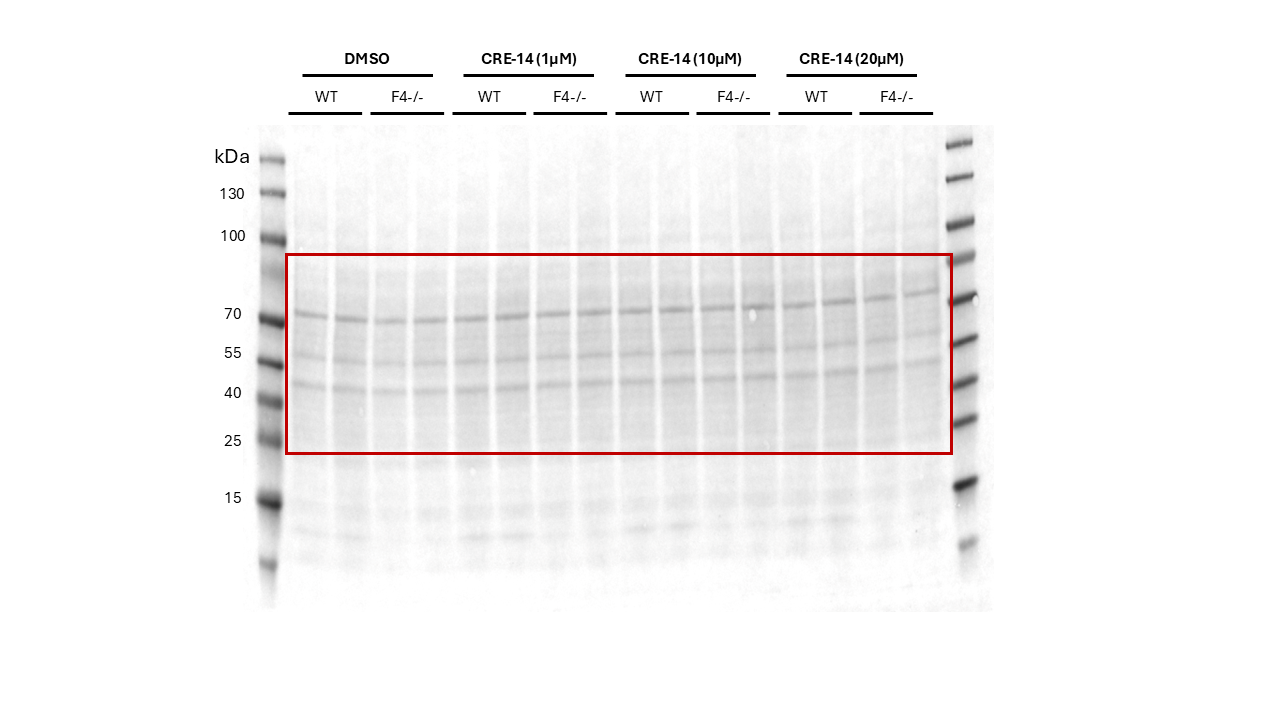

Supplement: Supplementary file 11 — Figure EV1-3 Source Data [file 44321_2024_188_MOESM11_ESM.zip › Expanded View 3/EV.3K-M/CRE-14_ponceau.tif]

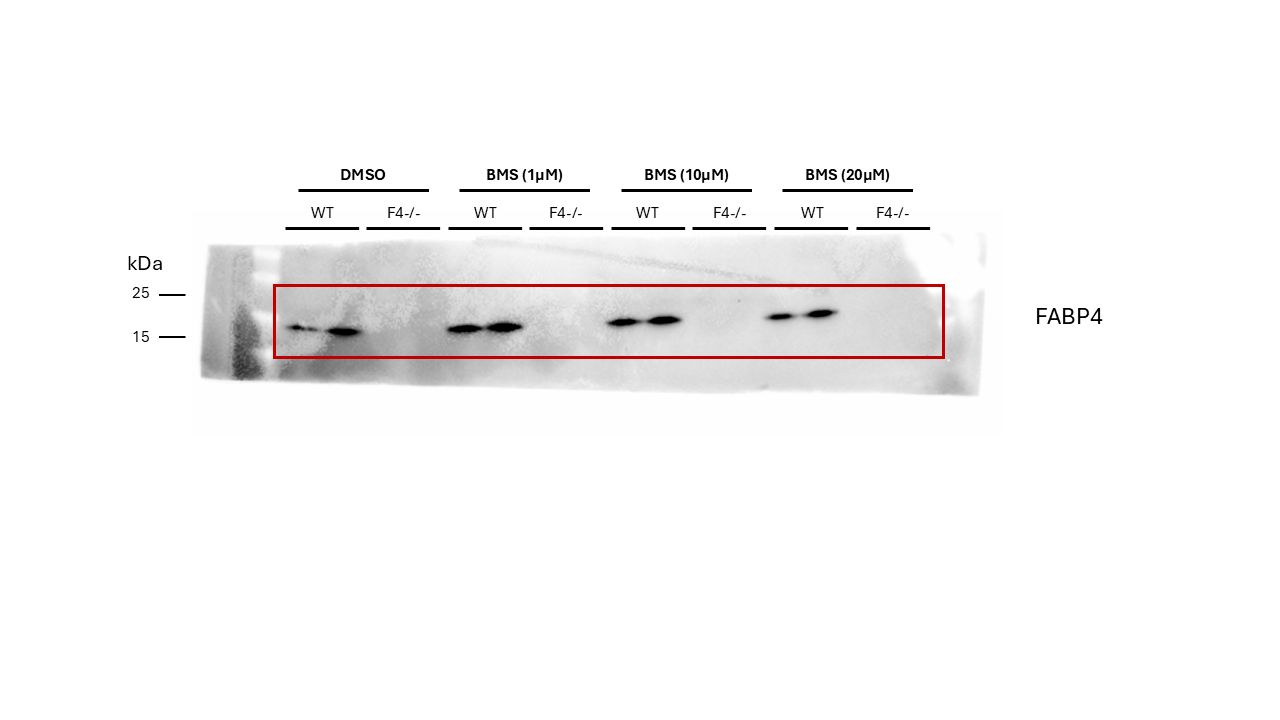

Supplement: Supplementary file 11 — Figure EV1-3 Source Data [file 44321_2024_188_MOESM11_ESM.zip › Expanded View 3/EV.3K-M/BMS_FABP4.tif]

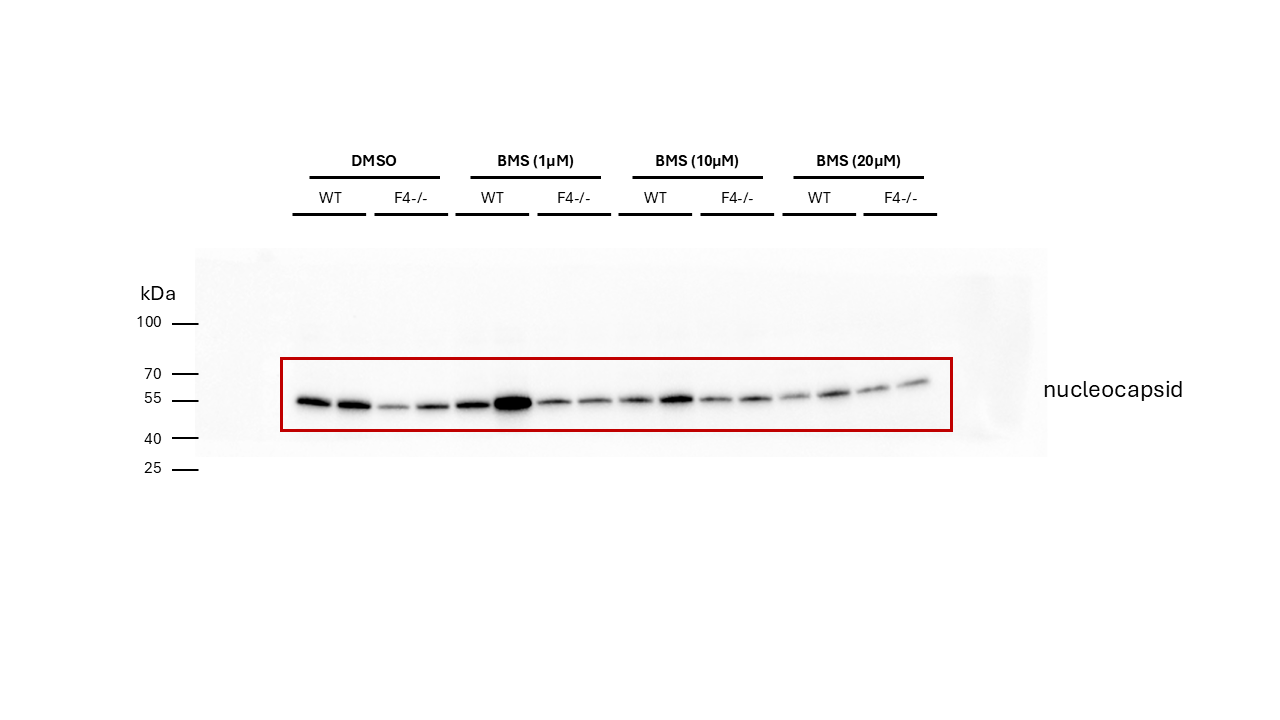

Supplement: Supplementary file 11 — Figure EV1-3 Source Data [file 44321_2024_188_MOESM11_ESM.zip › Expanded View 3/EV.3K-M/BMS_nucleocapsid.tif]

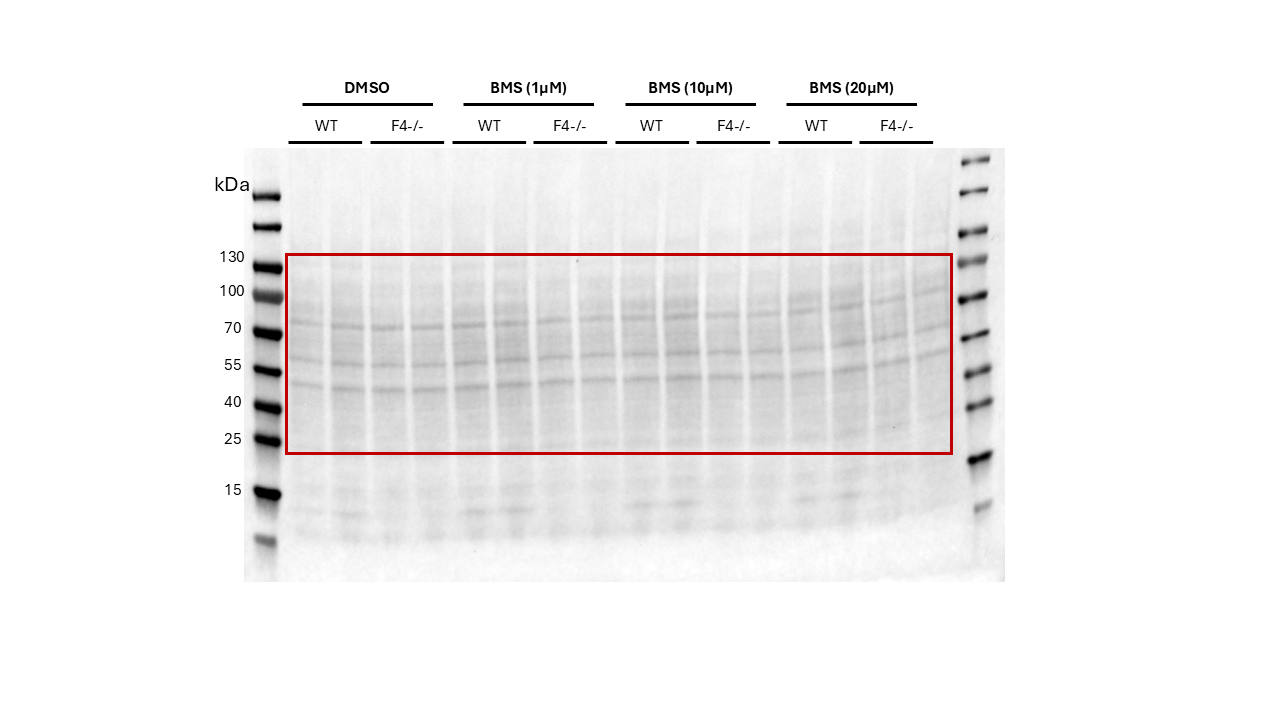

Supplement: Supplementary file 11 — Figure EV1-3 Source Data [file 44321_2024_188_MOESM11_ESM.zip › Expanded View 3/EV.3K-M/BMS_ponceau.tif]

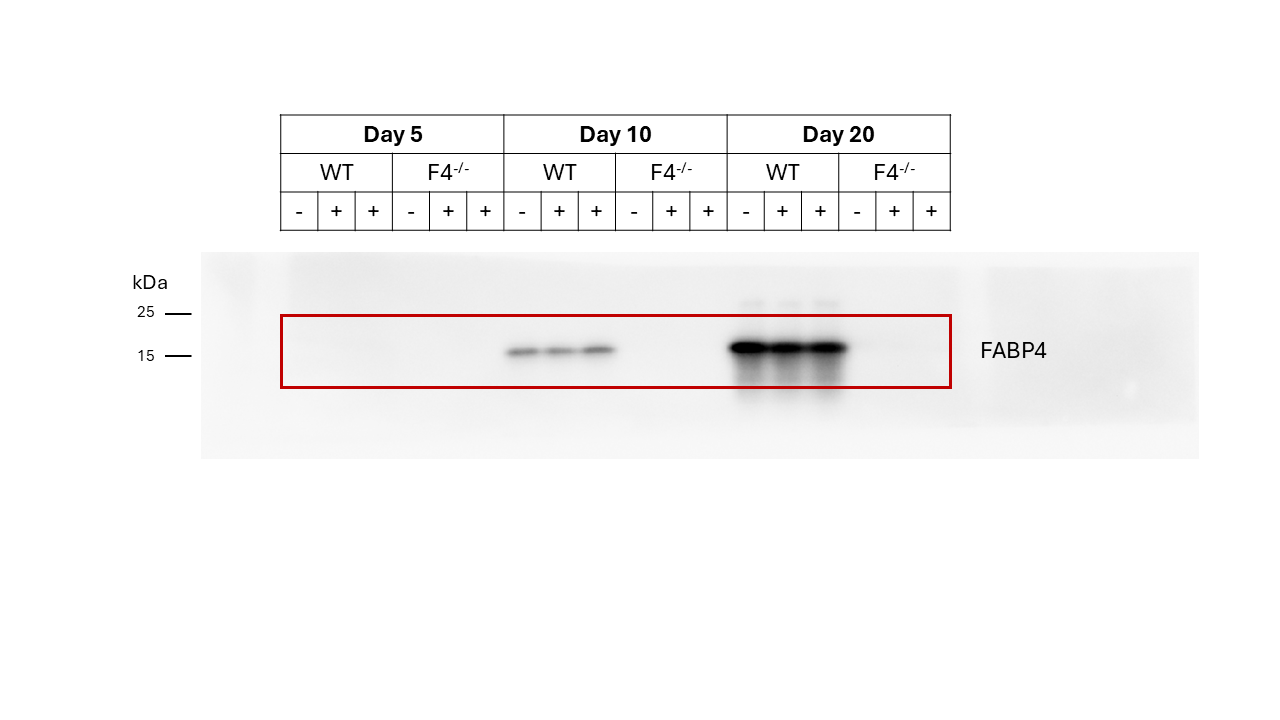

Supplement: Supplementary file 11 — Figure EV1-3 Source Data [file 44321_2024_188_MOESM11_ESM.zip › Expanded View 3/EV.3O-P/FABP4.tif]

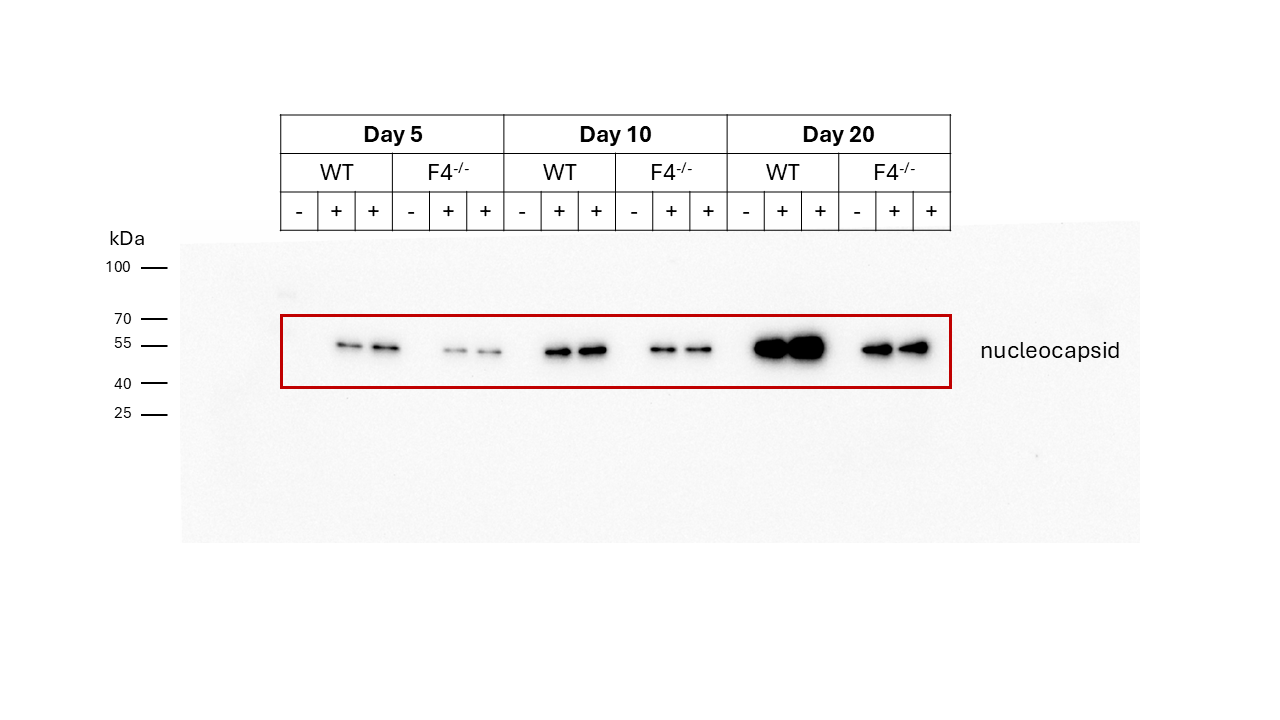

Supplement: Supplementary file 11 — Figure EV1-3 Source Data [file 44321_2024_188_MOESM11_ESM.zip › Expanded View 3/EV.3O-P/nucleocapsid.tif]

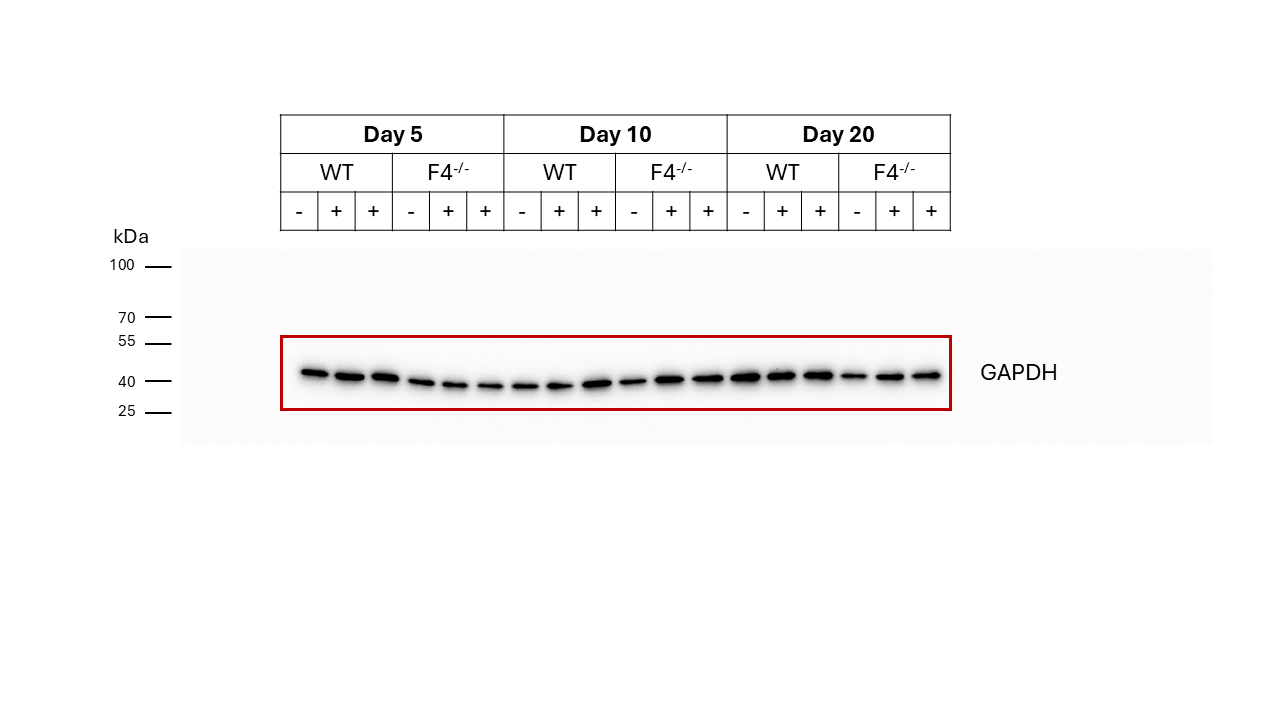

Supplement: Supplementary file 11 — Figure EV1-3 Source Data [file 44321_2024_188_MOESM11_ESM.zip › Expanded View 3/EV.3O-P/GAPDH.tif]

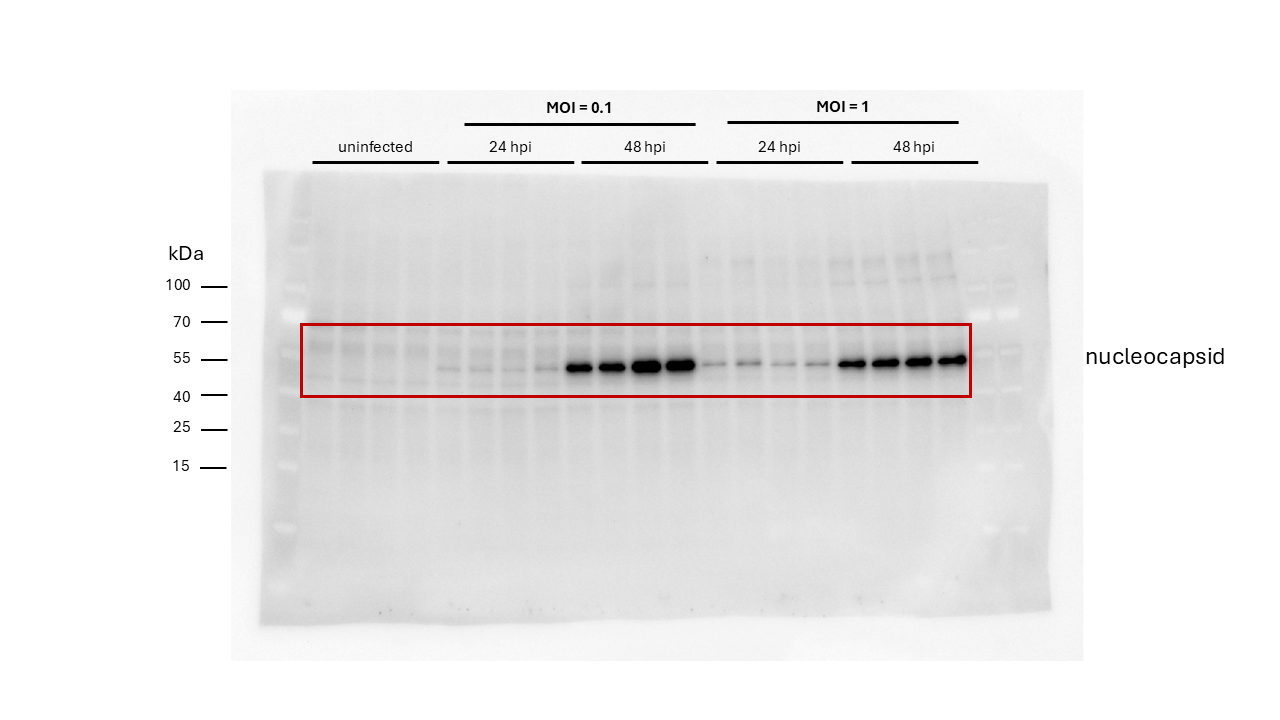

Supplement: Supplementary file 11 — Figure EV1-3 Source Data [file 44321_2024_188_MOESM11_ESM.zip › Expanded View 2/EV.2E-F/WB_nucleocapsid.tif]

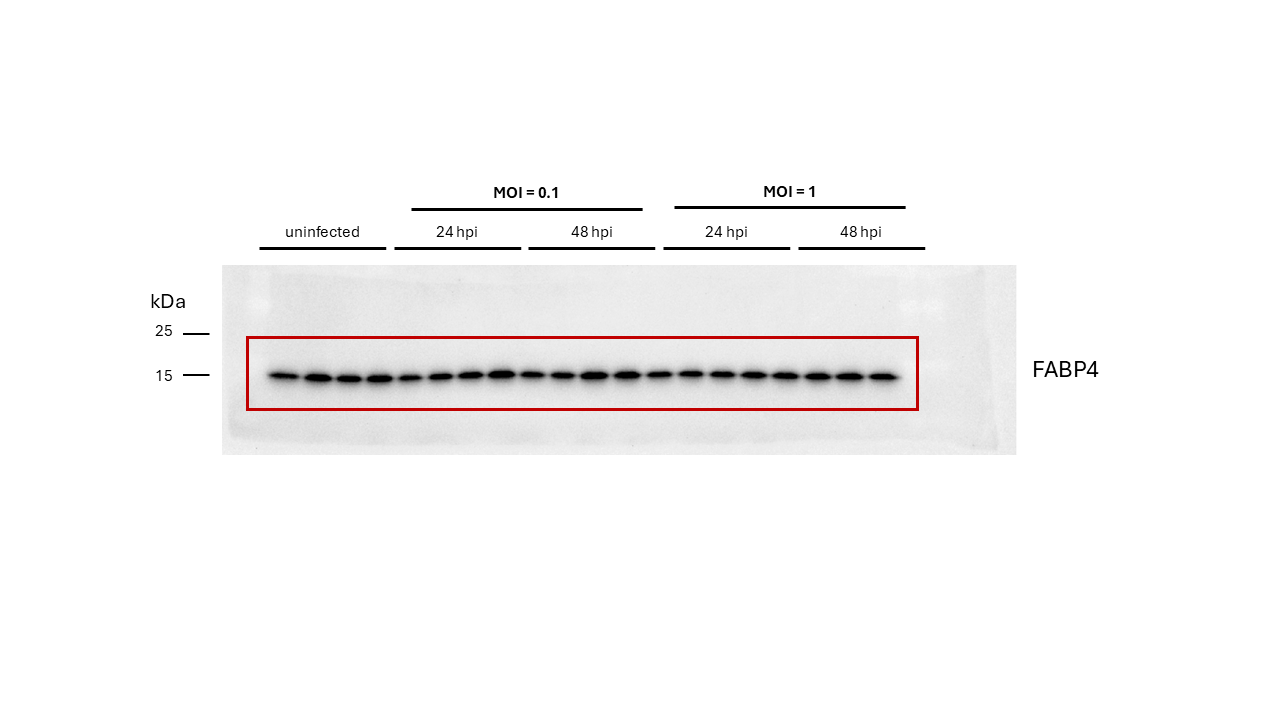

Supplement: Supplementary file 11 — Figure EV1-3 Source Data [file 44321_2024_188_MOESM11_ESM.zip › Expanded View 2/EV.2E-F/WB_FABP4.tif]

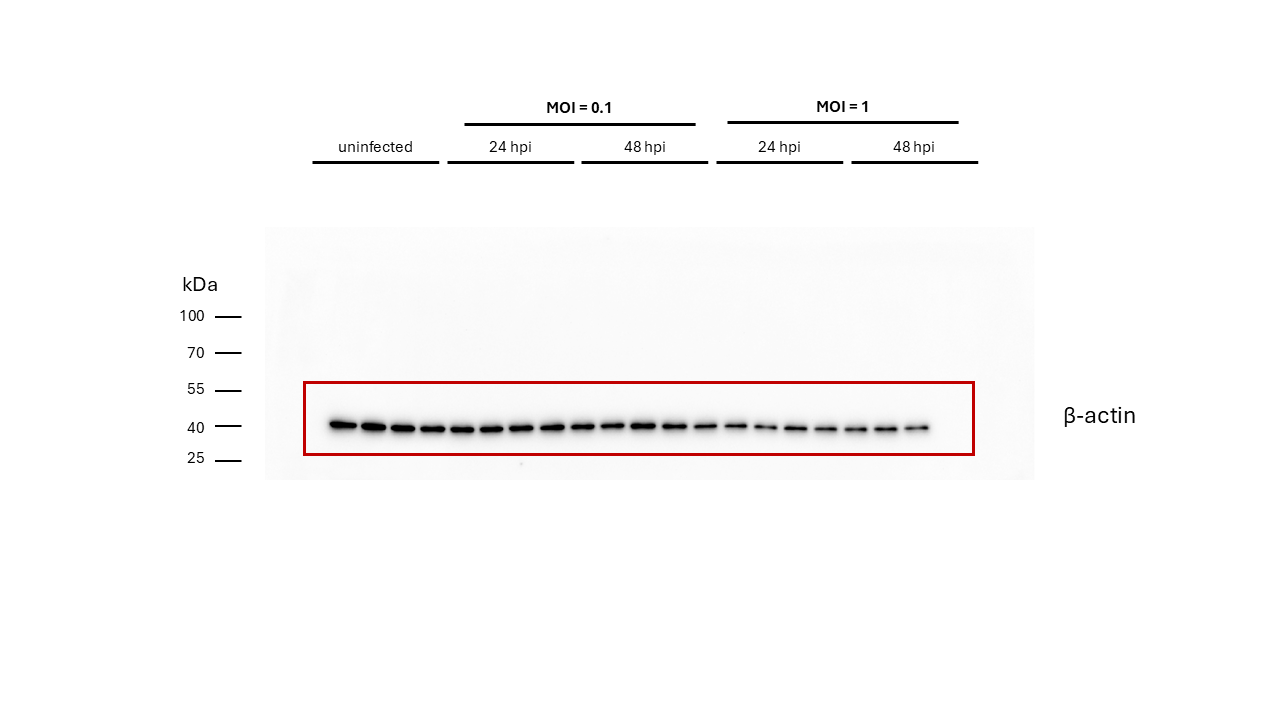

Supplement: Supplementary file 11 — Figure EV1-3 Source Data [file 44321_2024_188_MOESM11_ESM.zip › Expanded View 2/EV.2E-F/WB_actin.tif]

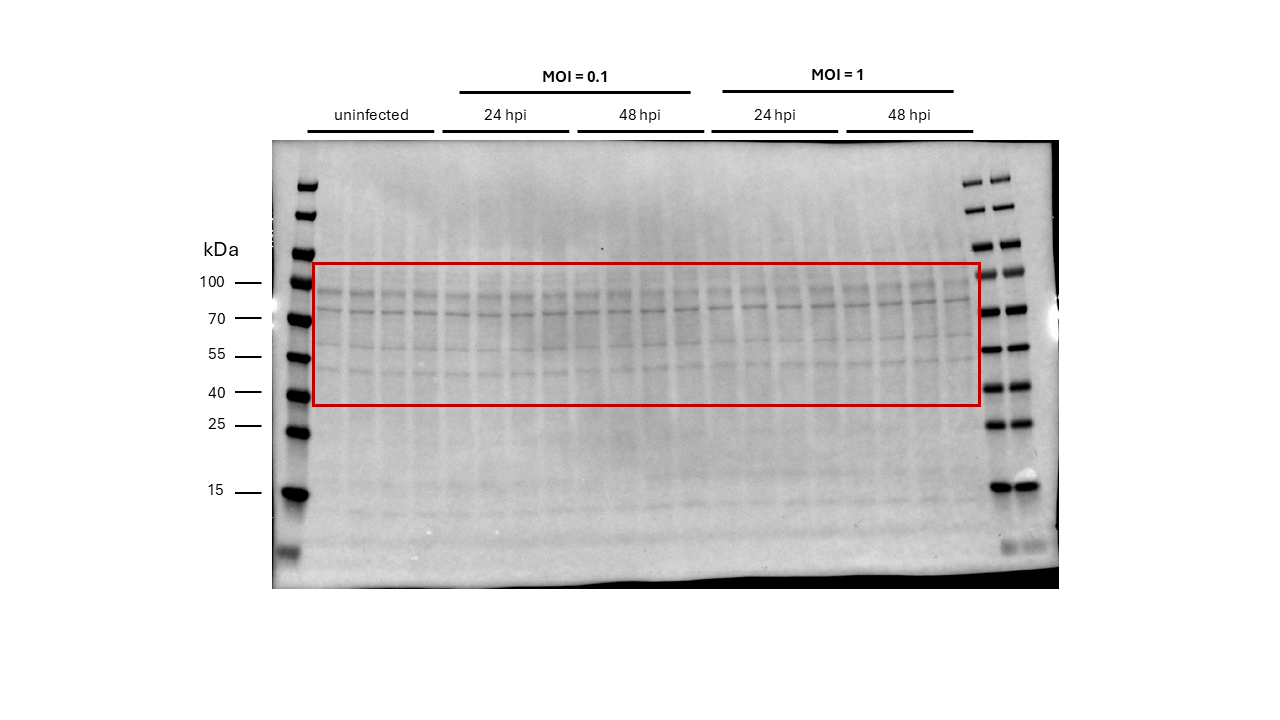

Supplement: Supplementary file 11 — Figure EV1-3 Source Data [file 44321_2024_188_MOESM11_ESM.zip › Expanded View 2/EV.2E-F/WB_ponceau.tif]

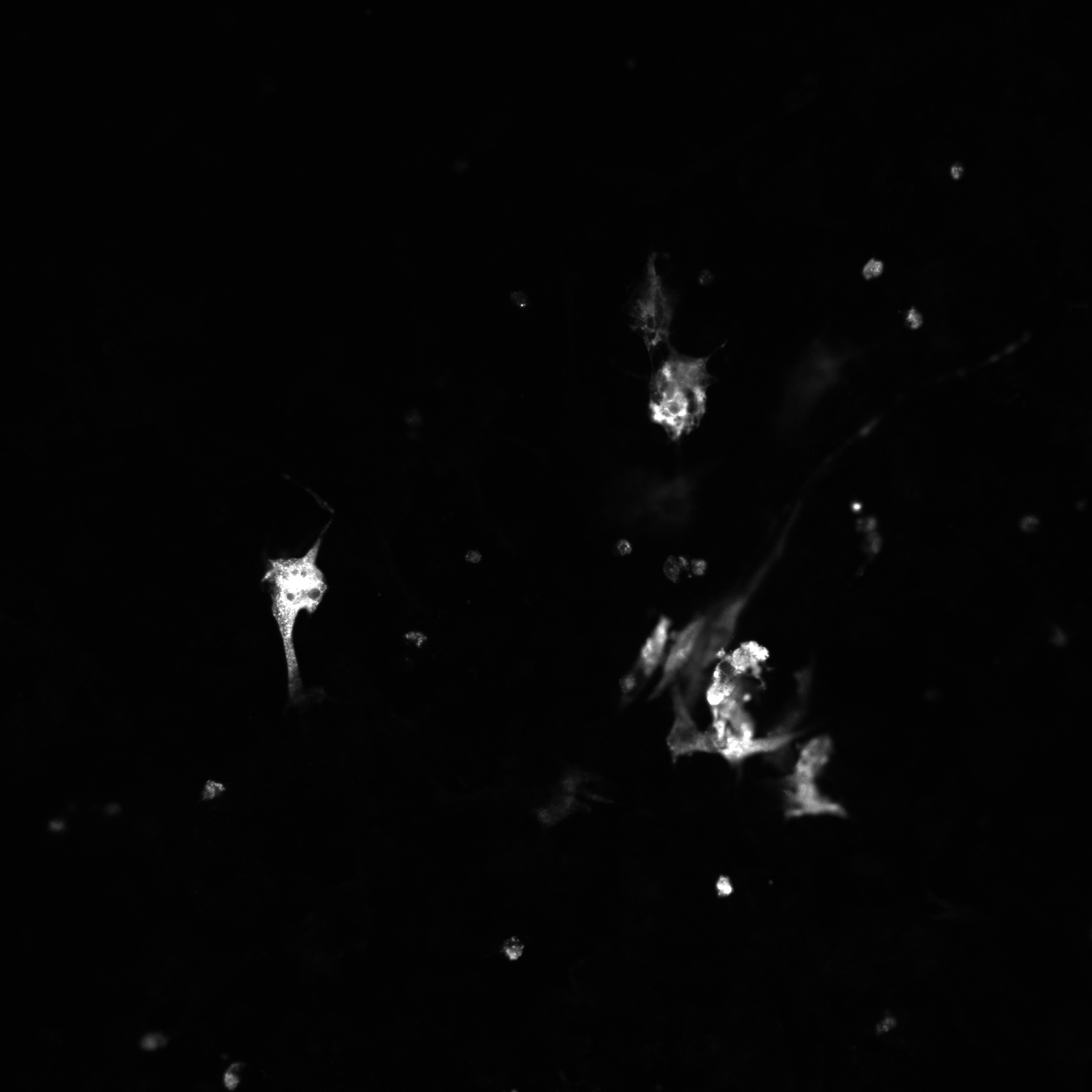

Supplement: Supplementary file 11 — Figure EV1-3 Source Data [file 44321_2024_188_MOESM11_ESM.zip › Expanded View 2/EV.2I/nucleocapsid_Infected_24hr_20X.tif]

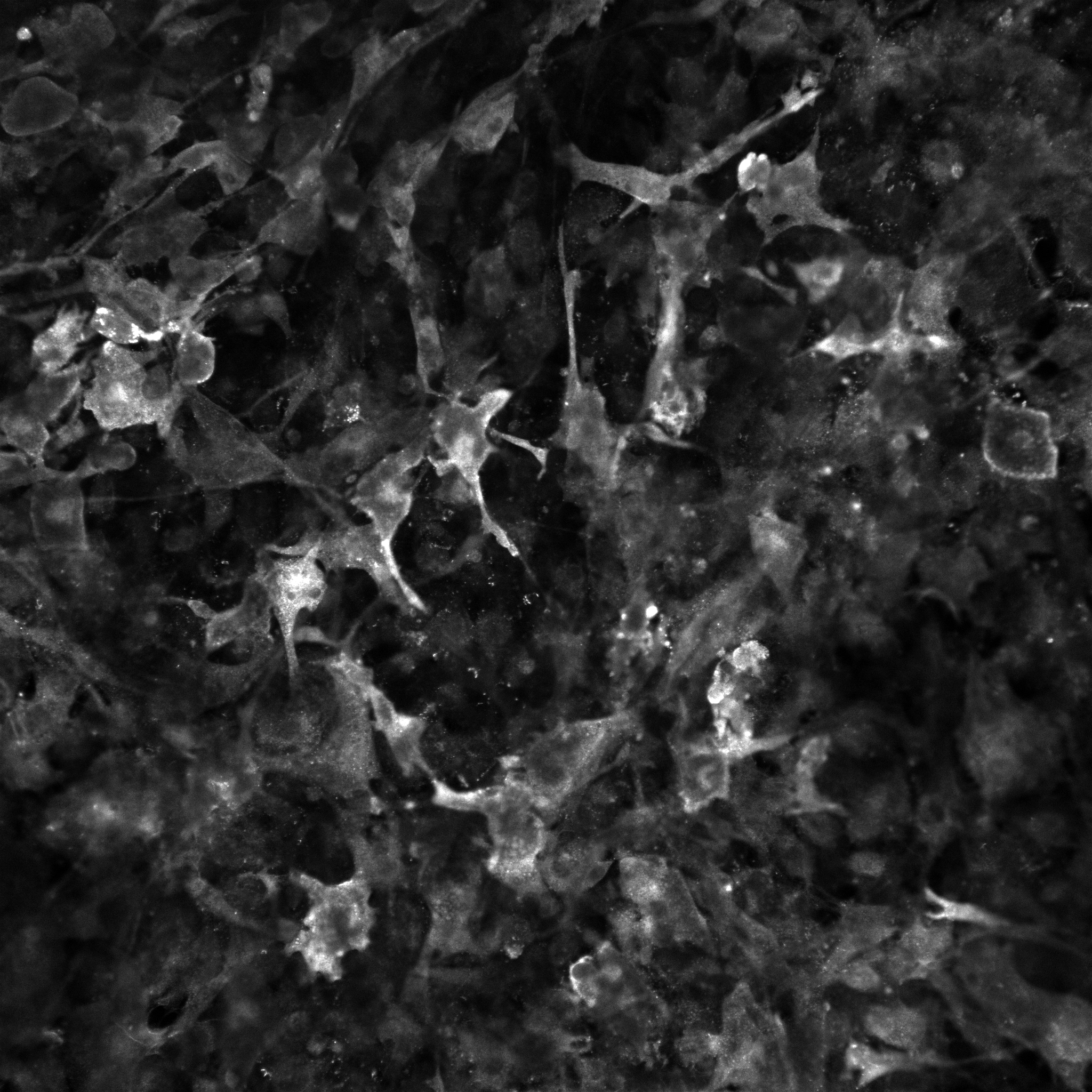

Supplement: Supplementary file 11 — Figure EV1-3 Source Data [file 44321_2024_188_MOESM11_ESM.zip › Expanded View 2/EV.2I/FABP4_Infected_24hr_20X.tif]

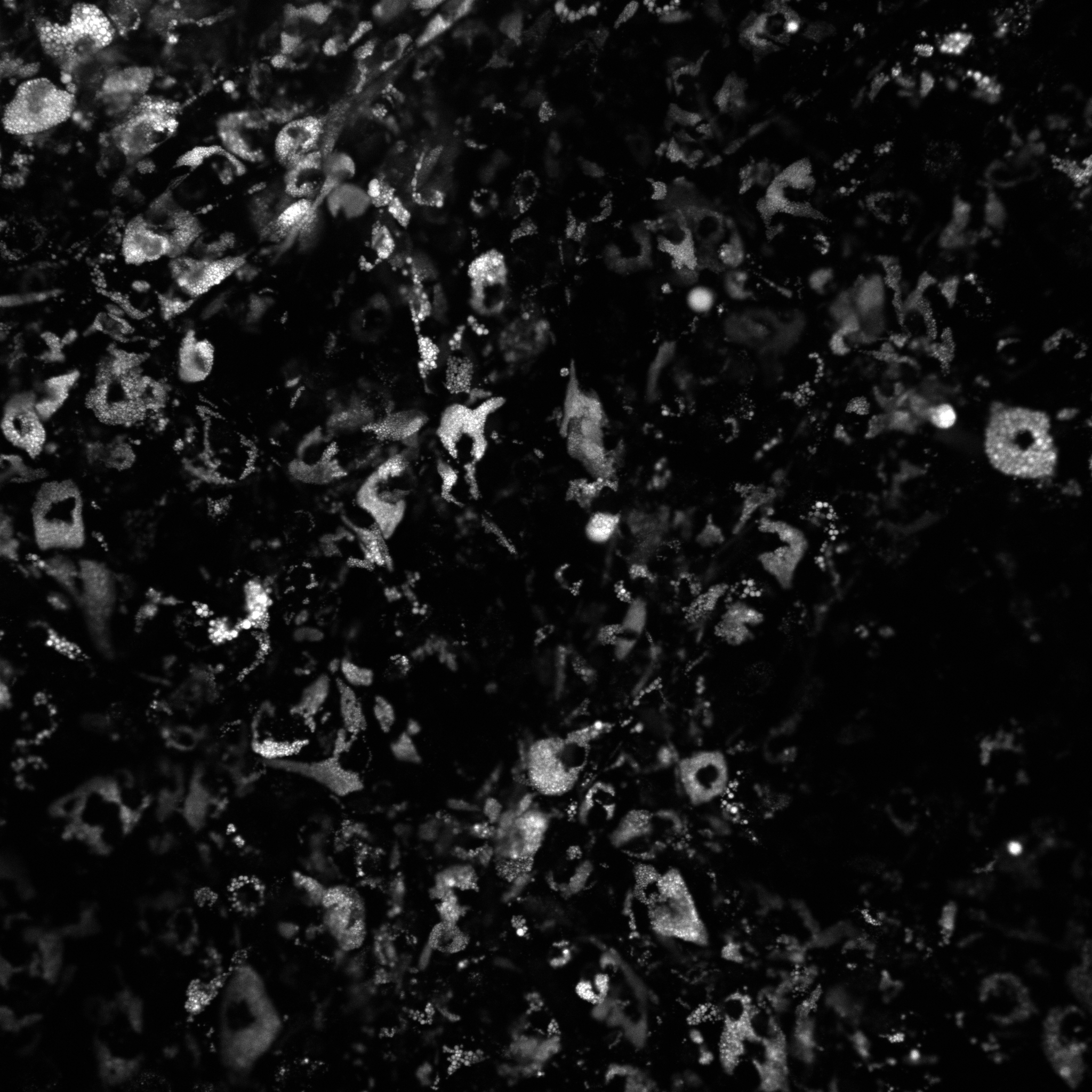

Supplement: Supplementary file 11 — Figure EV1-3 Source Data [file 44321_2024_188_MOESM11_ESM.zip › Expanded View 2/EV.2I/LDs_Infected_24hr_20X.tif]

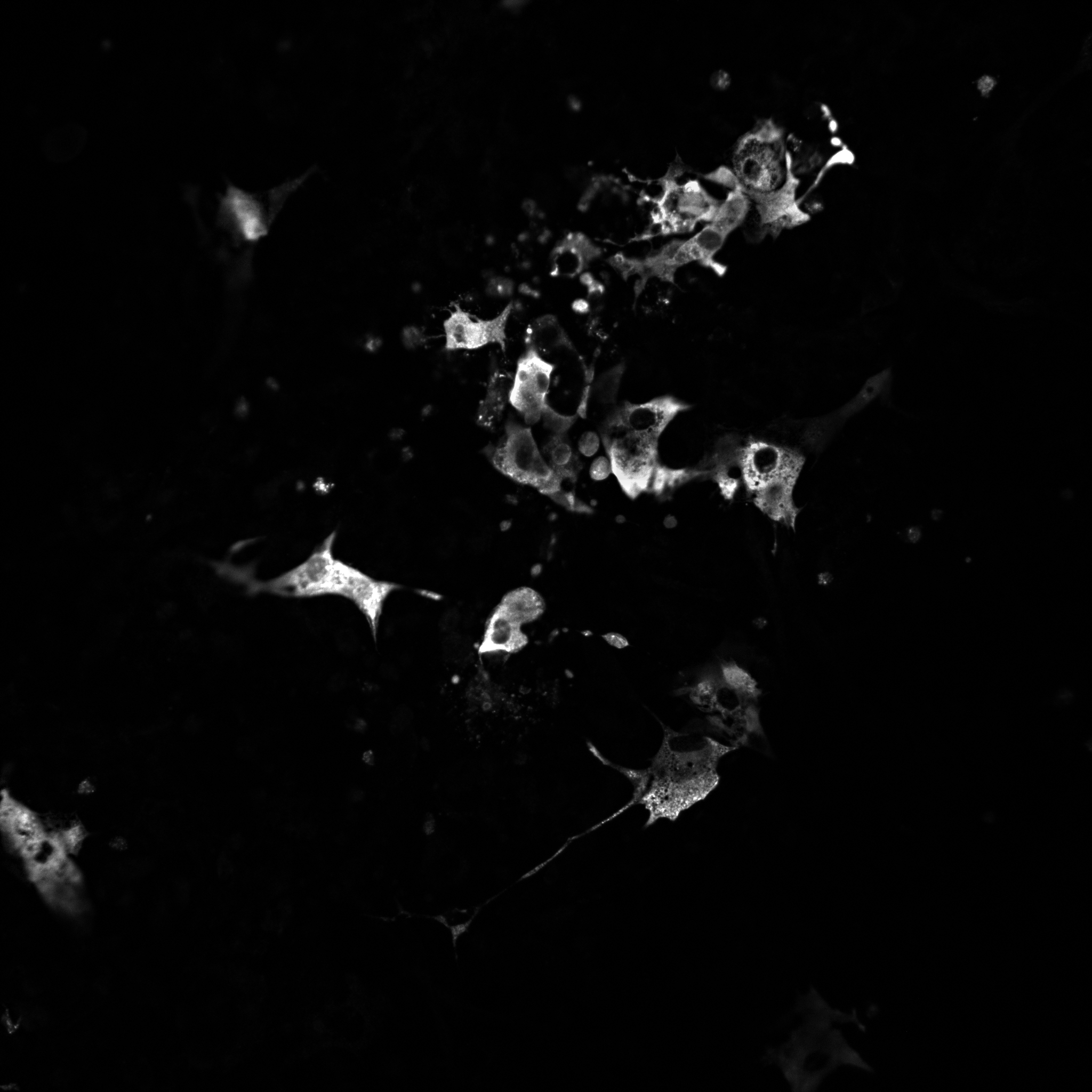

Supplement: Supplementary file 11 — Figure EV1-3 Source Data [file 44321_2024_188_MOESM11_ESM.zip › Expanded View 2/EV.2I/nucleocapsid_Infected_48hr_20X.tif]

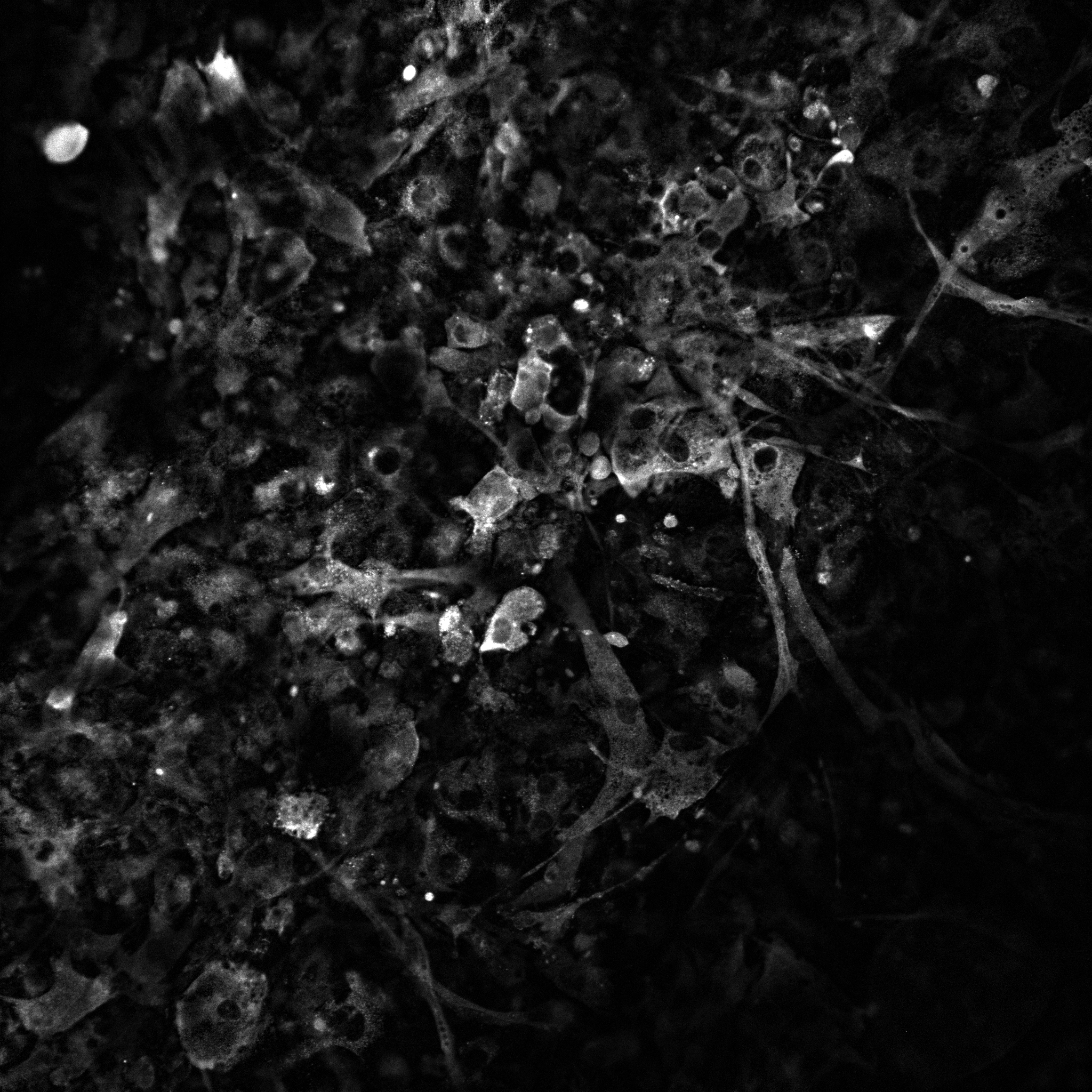

Supplement: Supplementary file 11 — Figure EV1-3 Source Data [file 44321_2024_188_MOESM11_ESM.zip › Expanded View 2/EV.2I/FABP4_Infected_48hr_20X.tif]

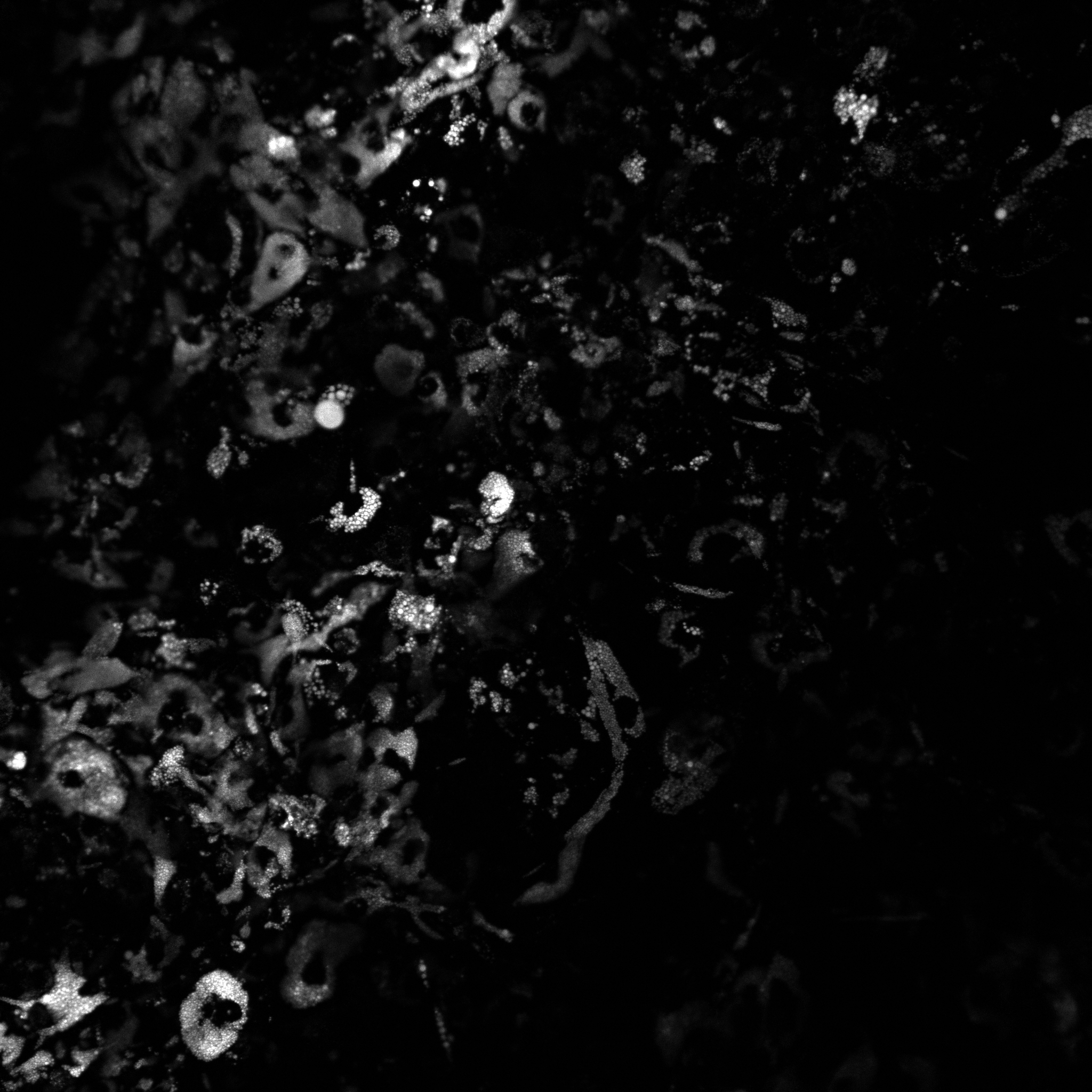

Supplement: Supplementary file 11 — Figure EV1-3 Source Data [file 44321_2024_188_MOESM11_ESM.zip › Expanded View 2/EV.2I/LDs_Infected_48hr_20X.tif]
