# Supplementary material for: FABP4 as a therapeutic host target controlling SARS-CoV-2 infection
Source: EMBO Mol Med. 2025 Jan 22;17(3):414–40. doi: 10.1038/s44321-024-00188-x (PMC11904229; doi:10.1038/s44321-024-00188-x)
Supplement: Supplementary file 11 — Figure EV1-3 Source Data [file 44321_2024_188_MOESM11_ESM.zip › Expanded View 3/EV.3B-C/MST Report FABP4.pdf]

2bind
molecular interactions

Dr. Thomas Schubert, 2021/09/06

Experimental Setup

Assay Type Fluorescent MST binding assay

Target (fluorescent molecule)

Name:	FABP4 NT-650-NHS-2nd Gen
Concentration (constant):	10 nM
Vol. in final reaction mix:	5 µl

Ligand (non-fluorescent molecule)

Name:	CRE-2229
Max. concentration:	20.0 µM
Min. concentration:	610 pM
Vol. in final reaction mix:	5 µl

Experimental setup

Assay buffer:	1 x PBS pH 7.4, 1 mM TCEP, 0.05% Pluronic F-127, 2% DMSO
Capillary type:	Premium Coated
MST Instrument:	Monolith NT.115 Pico (red-pico)
Laser Power:	40%
LED Power:	5%
Temperature:	25°C
Analysis Method:	MST Signal
Type of Repeats:	technical

Result overview Binding

	K_D	Amplitude	S/N
**Run 1**	758 nM	11.6	7.8
**Run 2**	1.15 µM	10.2	7.5

Protocol

A serial dilution of the ligand was prepared in a way to match the final buffer conditions in the reaction mix (assay buffer). The highest concentration of ligand was 40.0 µM and the lowest 1.22 nM. 5 µl of each dilution step were mixed with 5 µl of the fluorescent molecule. The final reaction mixture, which was filled in capillaries, contained a respective amount of ligand (max. conc. 20.0 µM, min. conc. 610 pM) and constant 10 nM fluorescent molecule.
The samples were analyzed on a Monolith NT.115 Pico (red-pico) at 25°C, with 5% LED power and 40% Laser power.

Statement of Quality

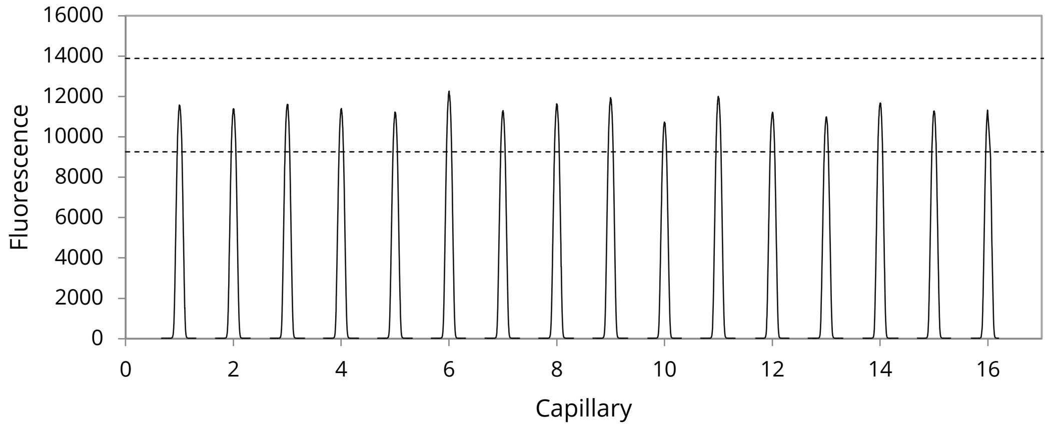

Capillary Scan

No sticking of the target to the capillary walls was observed in the capillary scan.

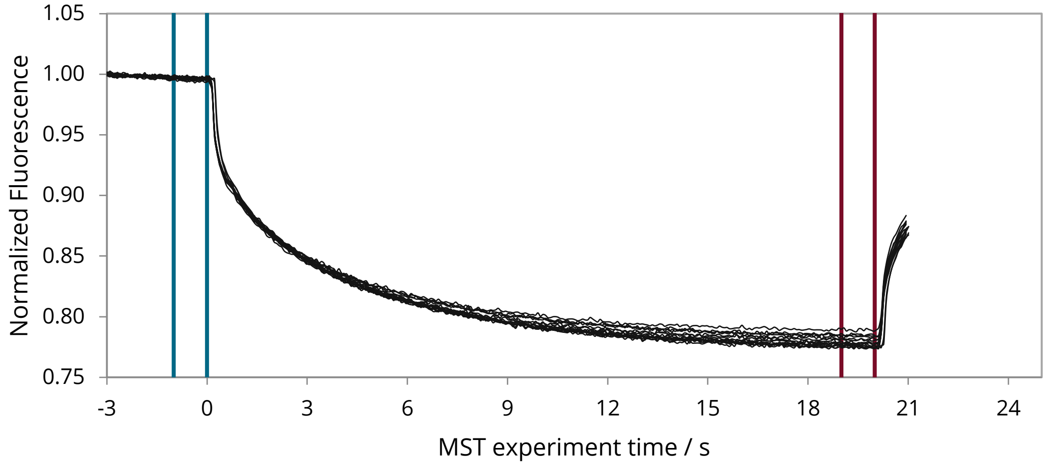

MST Traces

No sample aggregation or precipitation effects were observed in the normalized fluorescence.

MST Data

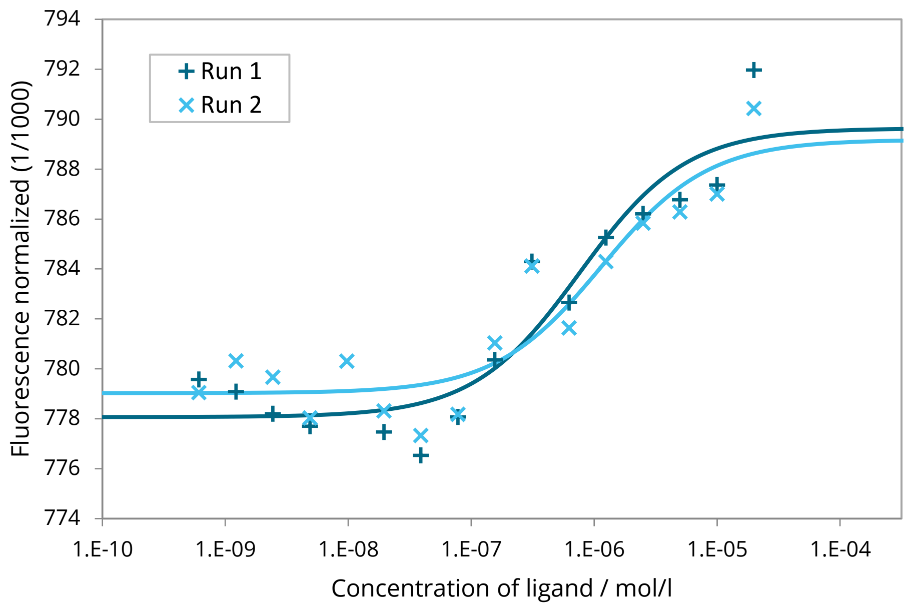

	K_D	Amplitude	S/N
**Run 1**	758 nM	11.6	7.8
**Run 2**	1.15 µM	10.2	7.5

Comment

Binding was detected in both technical runs. The Fnorm signal change was large enough for reliable data analysis. The signal/noise ratios of the data fits were sufficient (>5.0).

Raw Data

ligand conc. / nM	Run 1	Run 2
20000	792.0	790.4
10000	787.4	787.0
5000	786.8	786.3
2500	786.2	785.8
1250	785.3	784.3
625	782.7	781.6
312	784.3	784.1
156	780.4	781.0
78.1	778.1	778.2
39.1	776.5	777.3
19.5	777.5	778.3
9.77	792.6**	780.3
4.88	777.7	778.0
2.44	778.2	779.7
1.22	779.1	780.3

0.610	779.6	779.1

*outlier **excluded

Curve fit formula

Curve fit formula for 1:1 binding model:

$$A + T \Leftrightarrow AT$$

$$F(c_T) = F_u + (F_b - F_u) * \frac{c_{AT}}{c_A}$$

$$\frac{c_{AT}}{c_A} = fraction\ bound = \frac{1}{2c_A} * \Big(c_T + c_A + K_D - \sqrt{(c_T + c_A +}$$

$\mathbf{F_u}$	fluorescence in unbound state
$\mathbf{F_b}$	fluorescence in bound state
$\mathbf{K_D}$	dissociation constant, to be determined
$\mathbf{c_{AT}}$	concentration of formed complex
$\mathbf{c_A}$	constant concentration of molecule A (fluorescent),
$\mathbf{c_T}$	concentration of molecule T in serial dilution

2bind
molecular interactions

2bind molecular interactions

$$\overline{\cdot\, K_D)^2 - 4c_T c_A}\Big)$$

known

2bind molecular interactions

How to read this report

Experimental setup

Gives general information about the experimental setup, such as assay type, target (the fluorescently labelled molecule), ligand (the non-labelled molecule), buffer conditions, and dilution steps. Also gives information on the technical setup and a summary of the parameters of the molecular interaction.

Statement of quality

A great advantage of MST is the direct information about the quality of the technical process. Aggregation or precipitation of molecules as well as sticking effects in the capillaries can be detected in real-time during the measurements. The statement of quality summarizes this important information and gives information on the technical quality of the measurement.

MST data

Shows the normalized fluorescence graph for the repeat measurements and the raw data for each ligand concentration. In case the data points cannot be fitted to obtain a binding curve, the statement “no interaction detectable” is given. When the progression of the binding curve is clearly visible, but no proper curve is possible (for example due to a lack of data points in the plateau regions), the statement “binding indicated” is given. If the data points allow for a high quality curve-fit by using either the Hill equation or the K_D fit derived from the law of mass action according to the binding model, the affinity is calculated and stated as EC50 or K_D values. In addition, the amplitude of the binding curve is given. Please note that due to intrinsic thermophoresis properties, different target and ligand pairs can result positive or negative amplitudes. S/N is signal-to-noise ratio which is amplitude devided by noise. Noise is defined as standard deviation of difference between experimental data and fitted data.

Curve Fit

In the case an interaction has been detected, this page gives the mathematical formula used for fitting the normalized fluorescence data. Either the K_D fit derived from the law of mass action or the Hill equation are used for curve fitting.

FAQ

Aggregation

Sample aggregation can be identified from bumpy, uneven MST traces. Large aggregates move into the measurement focus by a convective flow induced by the local heating of the capillary. We classify MST traces into three categories: no aggregation, minor aggregation, and heavy aggregation, based on the extent of the aggregation signals in the traces. In general, we state minor aggregation if some aggregation signals are present in more than one trace and heavy aggregation if almost all traces display heavy aggregation signals. Note that in the merged display of all MST traces, minor aggregations present in individual traces can be covered by the other traces. In the case of minor aggregation, evaluation of the MST traces is possible most of the time, because the “cold” and “hot” regions can be set to parts of the traces that are not disturbed by aggregation signals. In the case of heavy aggregation, data analysis is not possible most of the time.

Amplitude

The amplitude is the absolute difference between the F_{norm} value of the “unbound” plateau at low ligand concentrations and the “bound” plateau at high ligand concentrations. The amplitude can be either positive or negative depending on the direction of the binding curve (positive or negative slope in the transition part).

Binding curve

The normalized fluorescence (F_{norm}) values for each MST trace are calculated from the ratio of F_0/F_1. Each Fnorm value is then plotted in a semi-logarithmic manner against the ligand concentration to yield a dose-response curve. This curve can be fitted with the formula given in this report in order to determine the steady-state affinity of the interaction.

Capillary scan

The capillary scan graph shows the transverse fluorescence profile of the capillaries. The individual capillaries provide information on possible unspecific adsorption of the samples (sticking) to the capillary inner walls. Under optimal circumstances, no sticking occurs and the peaks have a single, well-defined maximum. When sticking occurs, the peaks are broadened and feature more than one maximum point. Moreover, the capillary scan allows to check for equal concentrations of fluorescent target, which is a prerequisite for reliable MST analysis later on. If all capillaries contain the same concentration of fluorescent molecule, the peak heights should not differ by more than 20%, which is indicated by the dashed lines in the graph.

"Cold" and "Hot" areas

The normalized fluorescence values for each MST trace are calculated from the ratio of F_0/F_1.
F_0 is the average fluorescence when the laser is not activated (“cold” area between the blue lines in the MST trace). F_1 is the average fluorescence after the laser is turned on at a certain point of the MST trace (“hot” area between the red lines in the MST trace). Please note, that the “cold” and “hot” areas are set according to the following definitions: Minimal noise (standard deviation of difference between responses of experimental data and fitted data) and maximal binding amplitude (difference between unbound and bound state). Thus, the positions of the lines may vary between different experiments.

FAQ

Evaluation strategies

MST Signal: In general, the MST signal is composed of a TRIC (temperature-related intensity change) component and a thermophoretic component. The TRIC component is dependent on the fluorescence properties of the dye-label and the temperature gradient. The thermophoretic component is dependent on ligand-induced changes in size, charge, and hydration shell of the target. Both the TRIC and thermophoretic component are influenced by interactions between ligand and target. For evaluation of the MST signal, the "cold" and "hot" regions are set according to the principles described under the FAQ point "Cold and hot areas".

Initial Fluorescence Signal: In case of a significant fluorescence enhancement or fluorescence quenching effect (see below for explanations of these phenomenons) the raw, initial fluorescence values are evaluated instead of the normalized fluorescence signal.

Fluorescence enhancement

Fluorescence enhancement describes the observation that high ligand concentrations result in higher than average raw fluorescence readouts. In MST capillary scans this is visible from higher capillary peaks at high ligand concentrations. Possible reasons for fluorescence enhancement are, for example, either direct or indirect interaction of the ligand with the fluorescent dye and concomitant changes in the fluorescent properties of the dye.

Fluorescence quenching

Fluorescence quenching describes the observation that high ligand concentrations result in lower than average raw fluorescence readouts. In MST capillary scans this is visible from lower capillary peaks at high ligand concentrations. Possible reasons for fluorescence quenching are, for example, either direct or indirect interaction of the ligand with the fluorescent dye and concomitant changes in the fluorescent properties of the dye.

Independent repeat

Independent repeats are replicated measurements of a target-ligand pair where the samples for the individual replicates are prepared independently. Every repeat is subsequently measured in several technical runs.

Technical repeat

Technical repeats are replicated measurements of a sample series within the same capillaries. They serve for verification of the technical reproducibility of the measurements with respect to the used MST device. The time interval between the individual replicates also enables check of equilibrium conditions as well as identification of time-dependent, unspecific sticking and aggregation effects.

2bind
molecular interactions

Disclaimer

This experiment was performed based on the general principles of Good Scientific Practice. The applied methods and employed devices comply with the state of technology at the time of experiment conduction. No data pertinent to this experiment have been added, deleted, or modified after completion of this experiment. Evaluation of the data reflect the actual state of scientific knowledge at the time of experiment conduction. It is advised to validate the data with at least one other biophysical method.
