# Supplementary figures and images for: FABP4 as a therapeutic host target controlling SARS-CoV-2 infection (part 2 of 2)
Source: EMBO Mol Med. 2025 Jan 22;17(3):414–40. doi: 10.1038/s44321-024-00188-x (PMC11904229; doi:10.1038/s44321-024-00188-x)

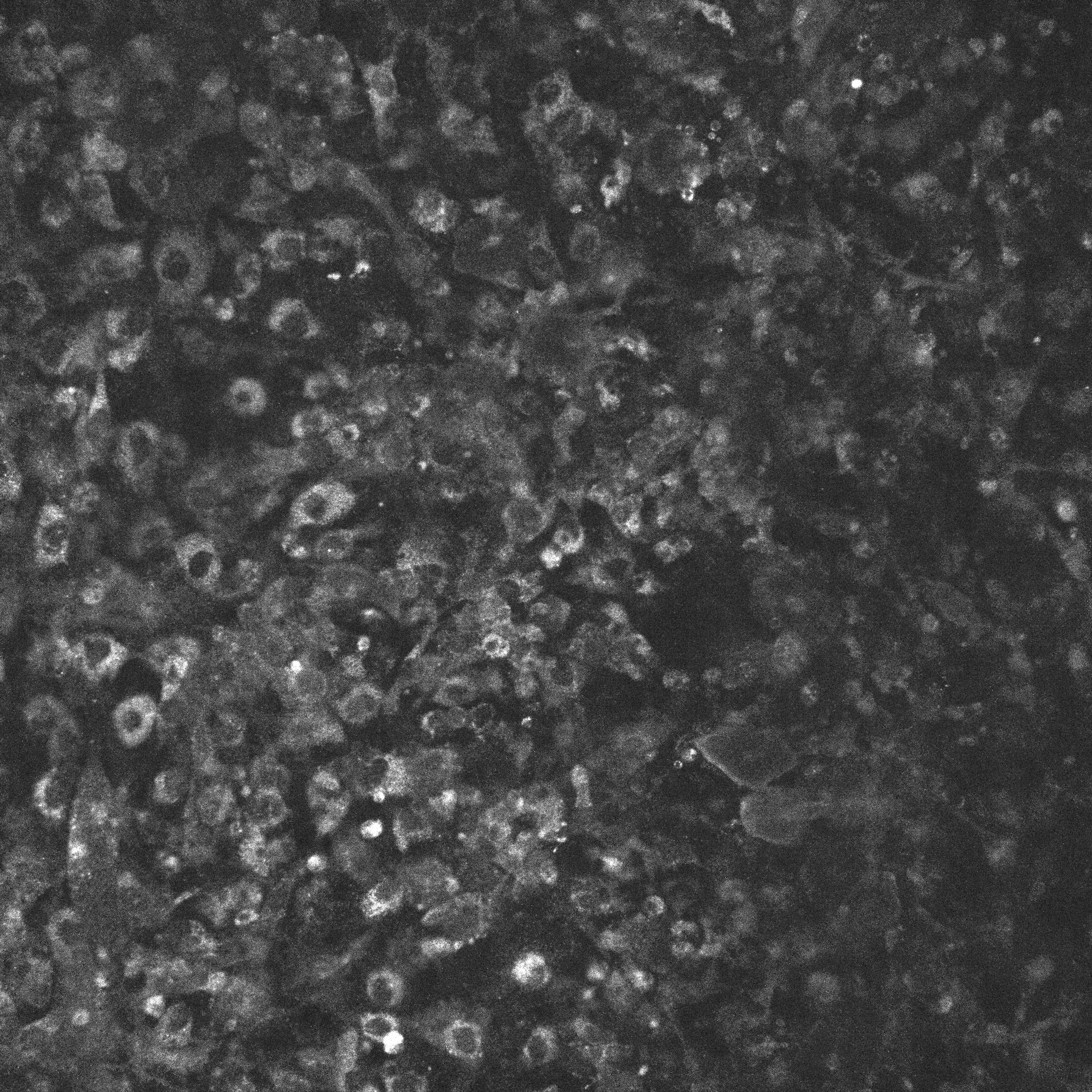

Supplement: Supplementary file 11 — Figure EV1-3 Source Data [file 44321_2024_188_MOESM11_ESM.zip › Expanded View 2/EV.2I/nucleocapsid_uninfected_20X.tif]

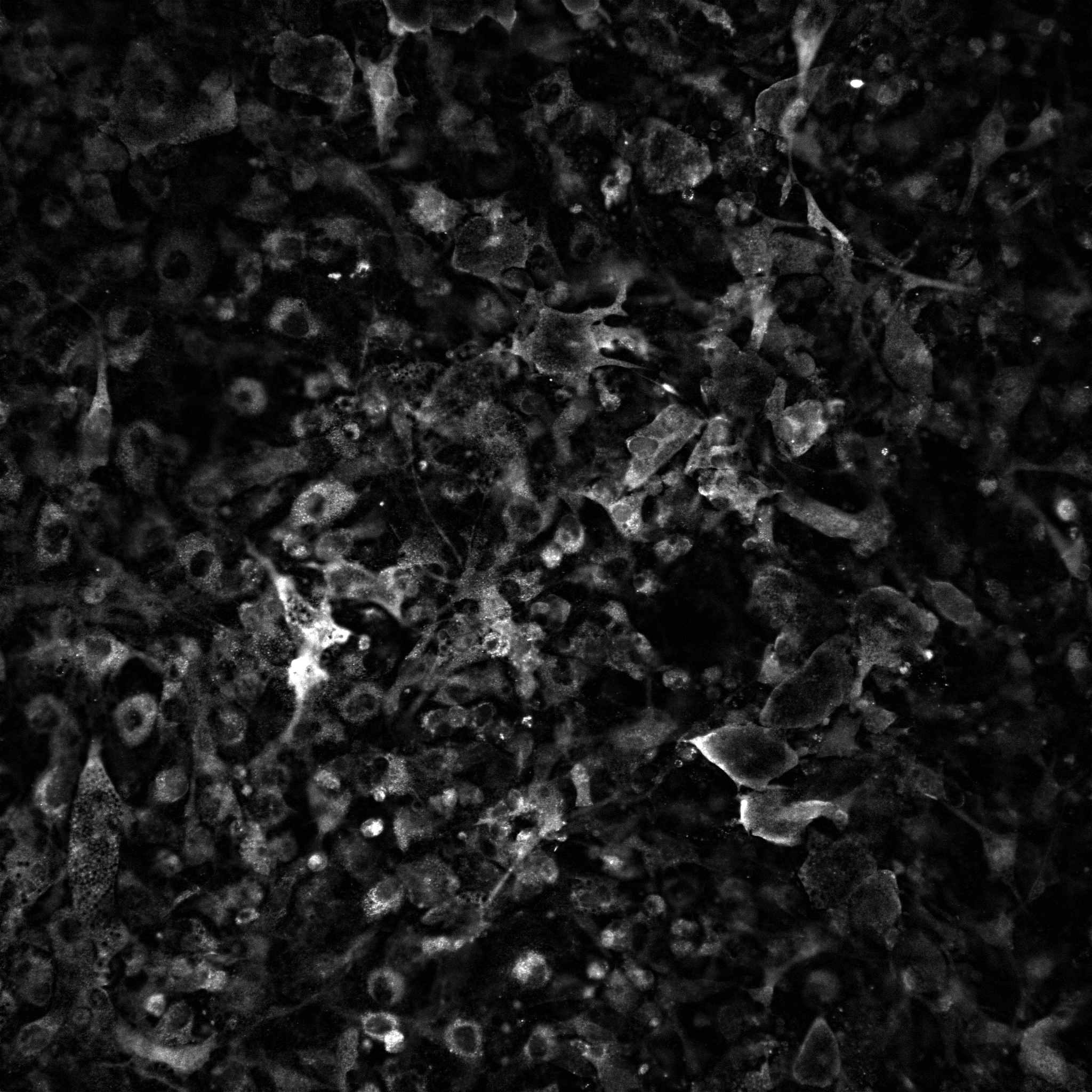

Supplement: Supplementary file 11 — Figure EV1-3 Source Data [file 44321_2024_188_MOESM11_ESM.zip › Expanded View 2/EV.2I/FABP4_uninfected_20X.tif]

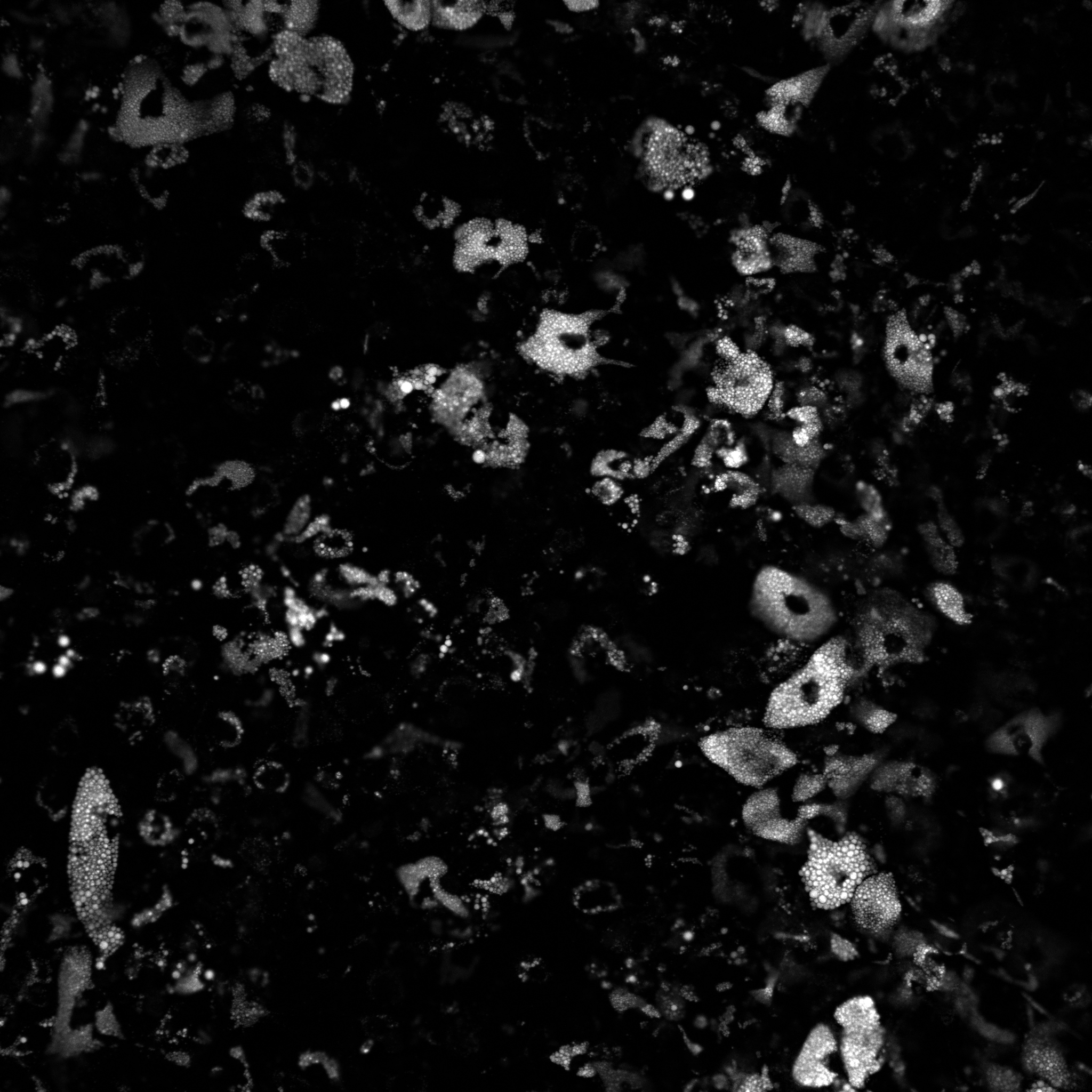

Supplement: Supplementary file 11 — Figure EV1-3 Source Data [file 44321_2024_188_MOESM11_ESM.zip › Expanded View 2/EV.2I/LDs_uninfected_20X.tif]

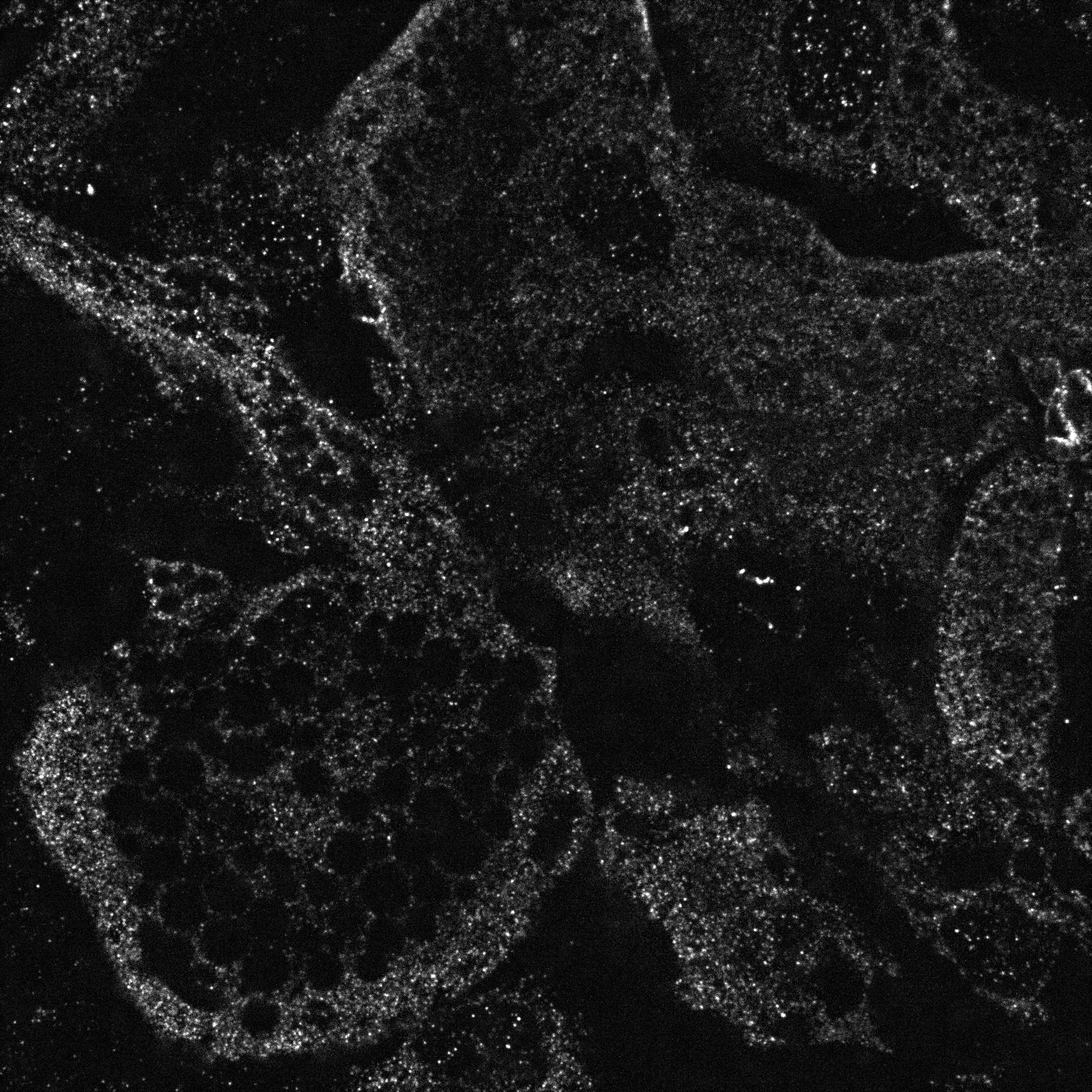

Supplement: Supplementary file 11 — Figure EV1-3 Source Data [file 44321_2024_188_MOESM11_ESM.zip › Expanded View 2/EV.2J/nucleocapsid_uninfected_100X.tif]

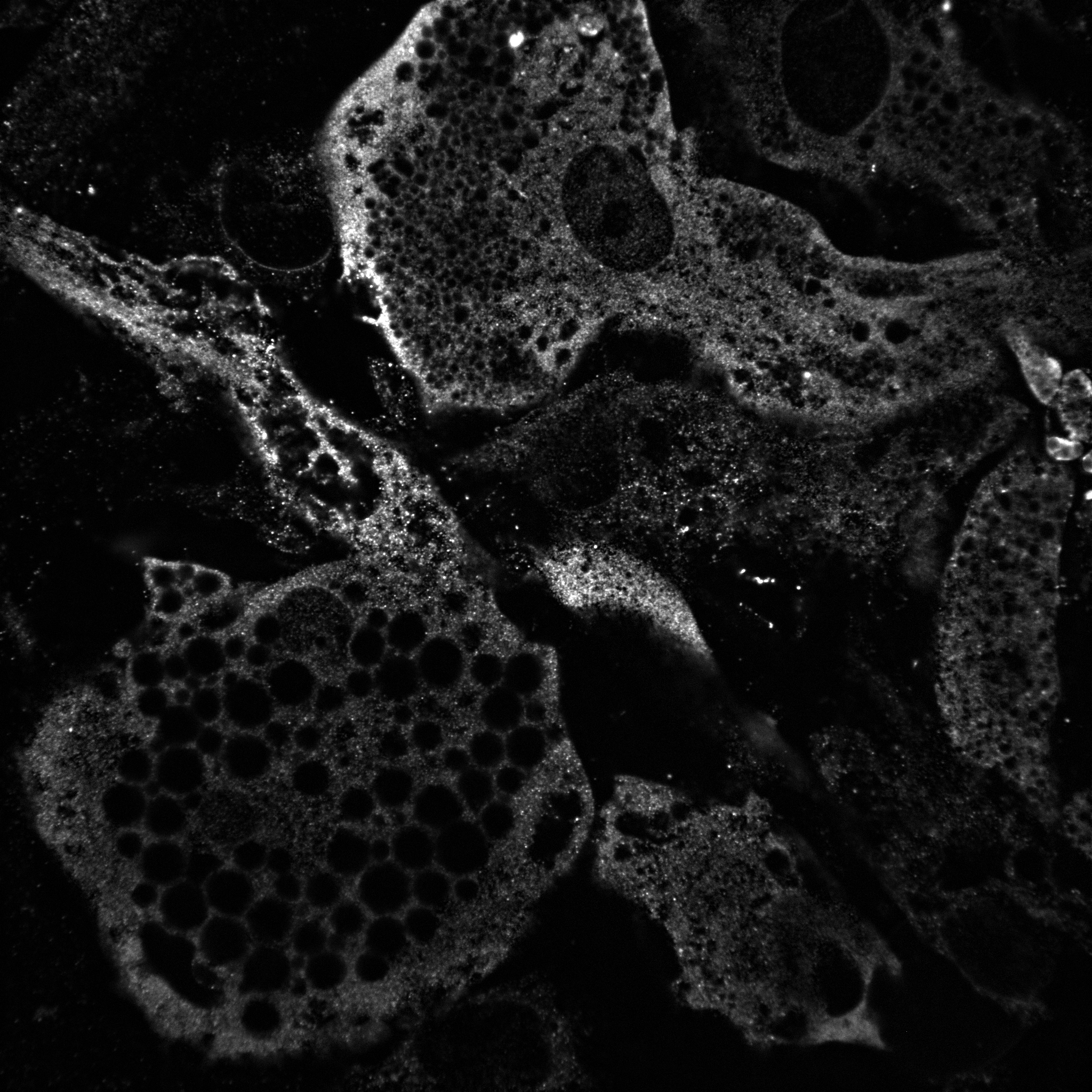

Supplement: Supplementary file 11 — Figure EV1-3 Source Data [file 44321_2024_188_MOESM11_ESM.zip › Expanded View 2/EV.2J/FABP4_uninfected_100X.tif]

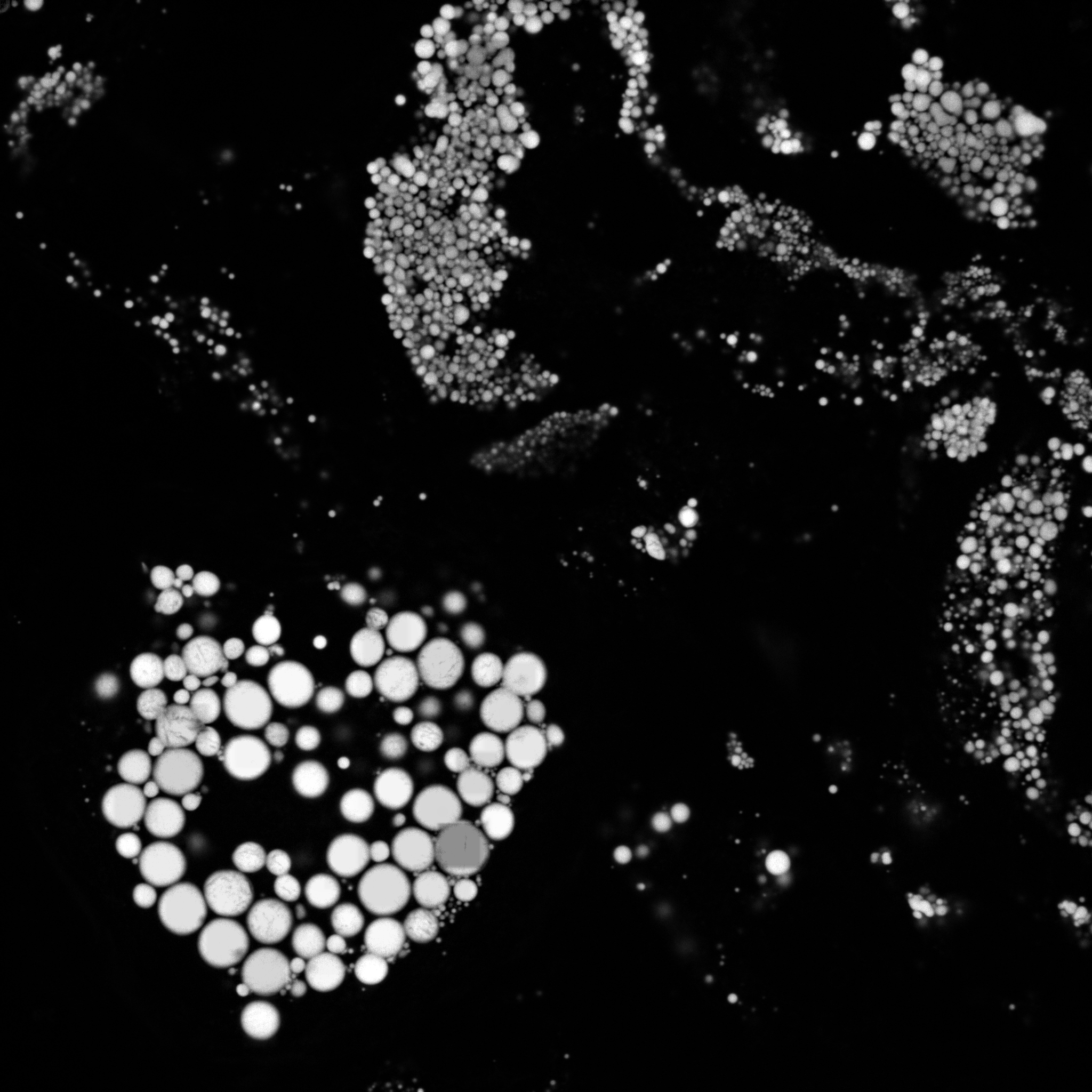

Supplement: Supplementary file 11 — Figure EV1-3 Source Data [file 44321_2024_188_MOESM11_ESM.zip › Expanded View 2/EV.2J/LDs_uninfected_100X.tif]

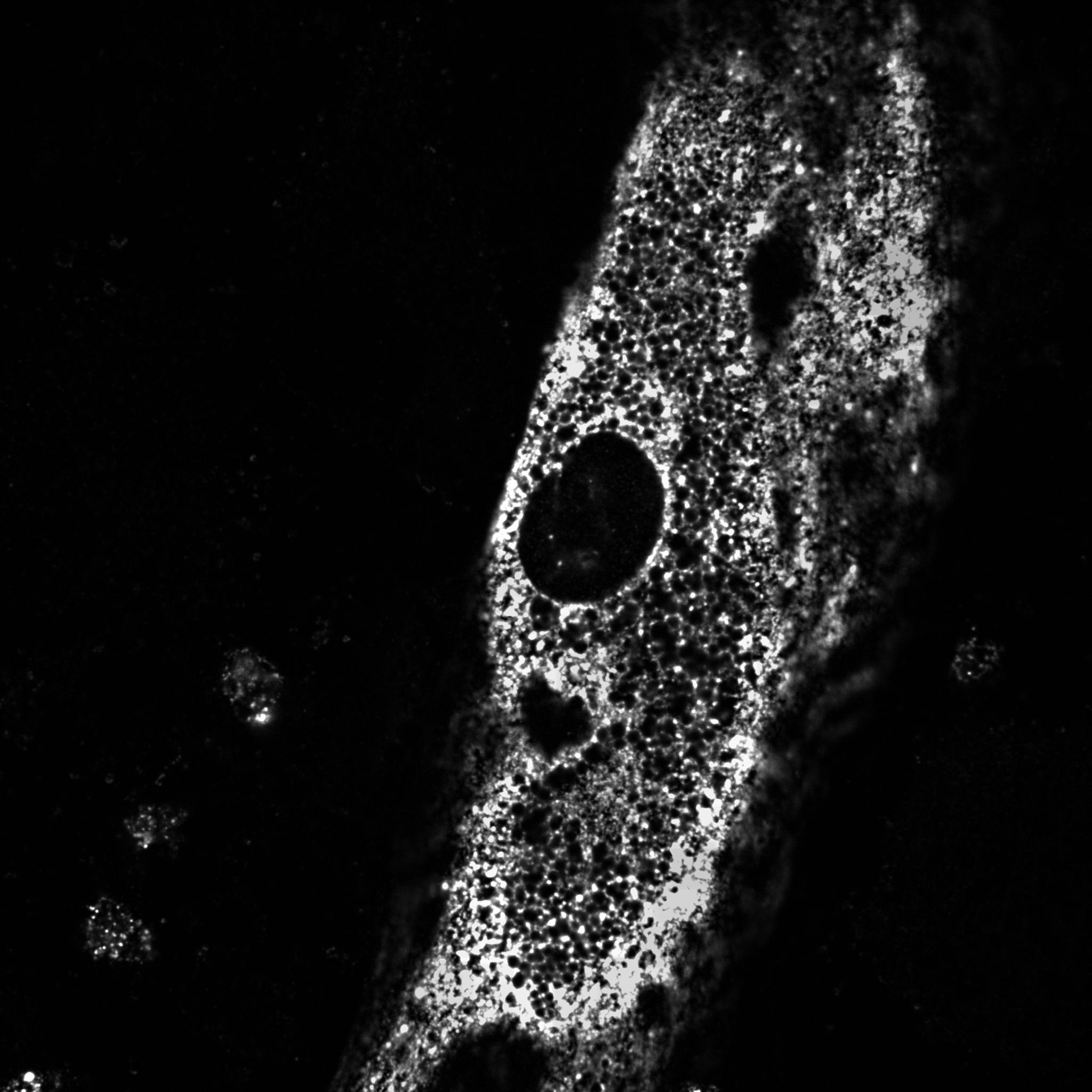

Supplement: Supplementary file 11 — Figure EV1-3 Source Data [file 44321_2024_188_MOESM11_ESM.zip › Expanded View 2/EV.2J/nucleocapsid_Infected_24hr_100X.tif]

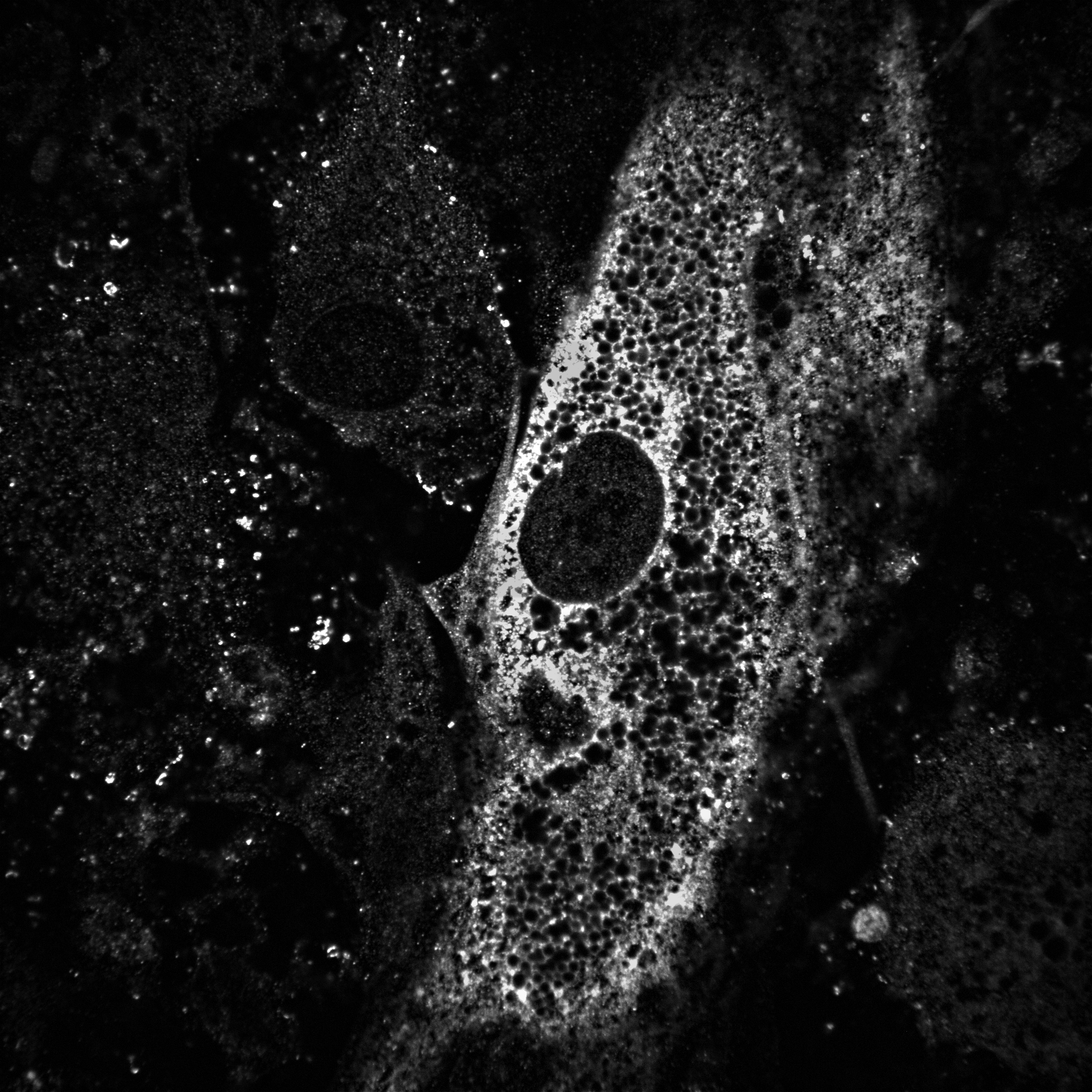

Supplement: Supplementary file 11 — Figure EV1-3 Source Data [file 44321_2024_188_MOESM11_ESM.zip › Expanded View 2/EV.2J/FABP4_Infected_24hr_100X.tif]

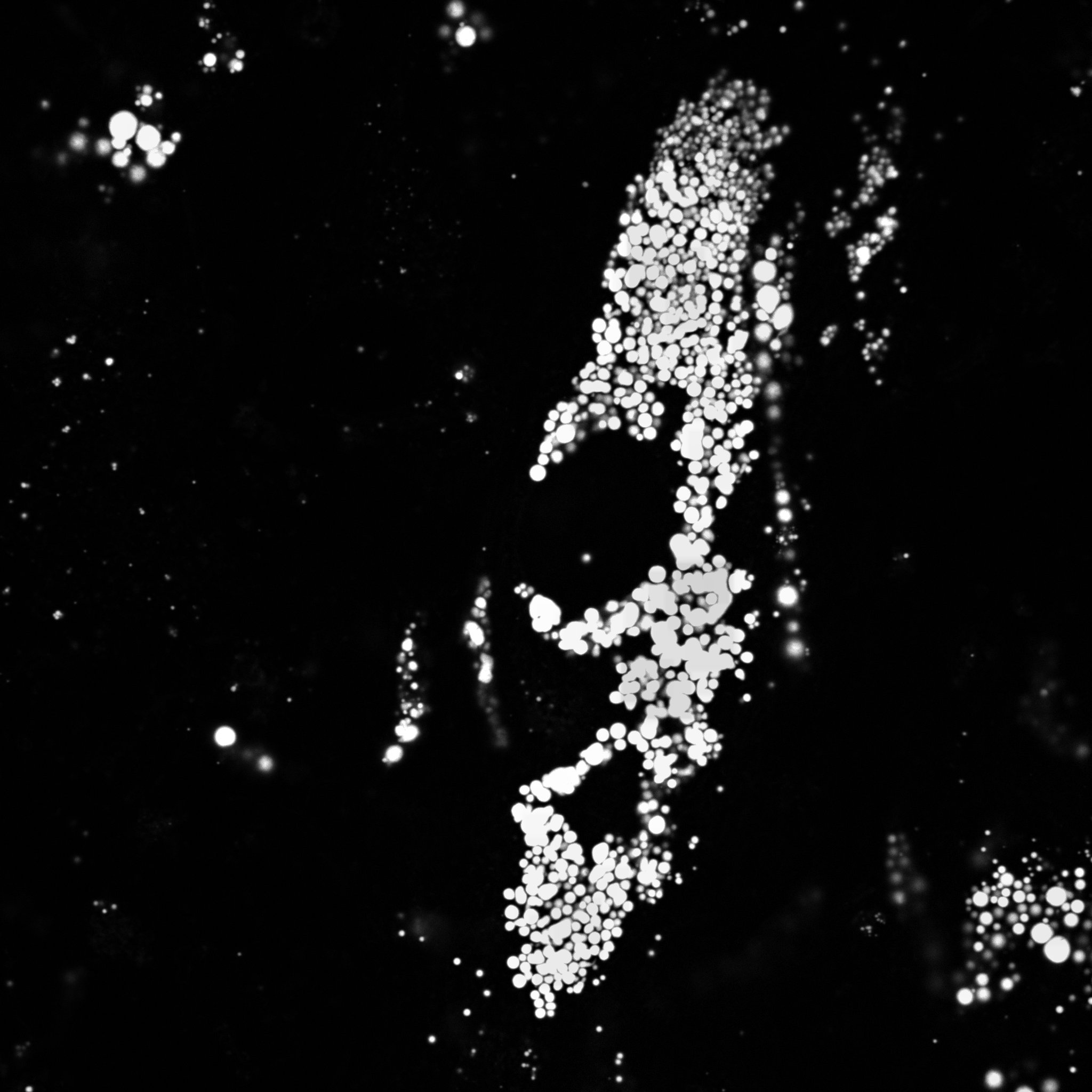

Supplement: Supplementary file 11 — Figure EV1-3 Source Data [file 44321_2024_188_MOESM11_ESM.zip › Expanded View 2/EV.2J/LDs_Infected_24hr_100X.tif]

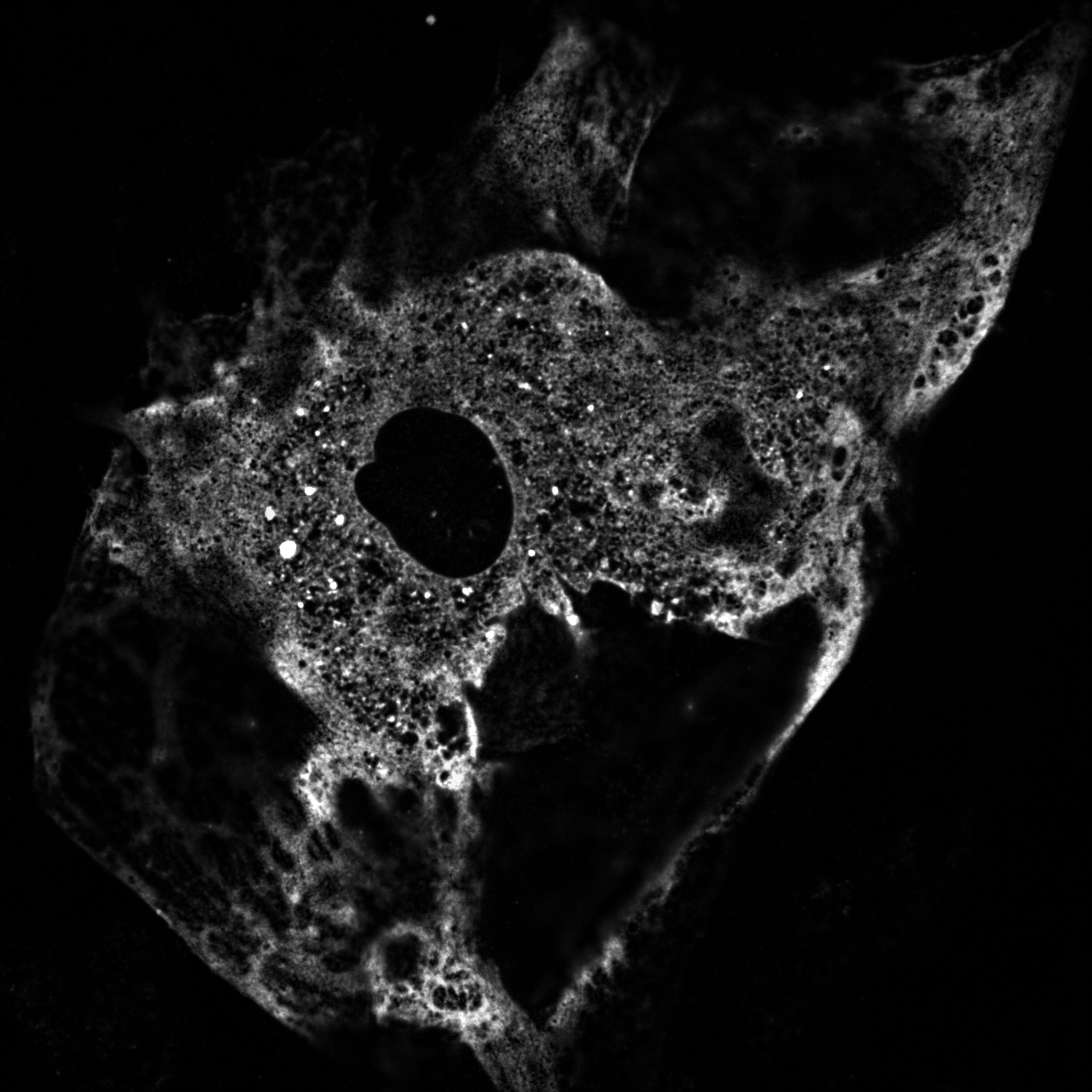

Supplement: Supplementary file 11 — Figure EV1-3 Source Data [file 44321_2024_188_MOESM11_ESM.zip › Expanded View 2/EV.2J/nucleocapsid_Infected_48hr_100X.tif]

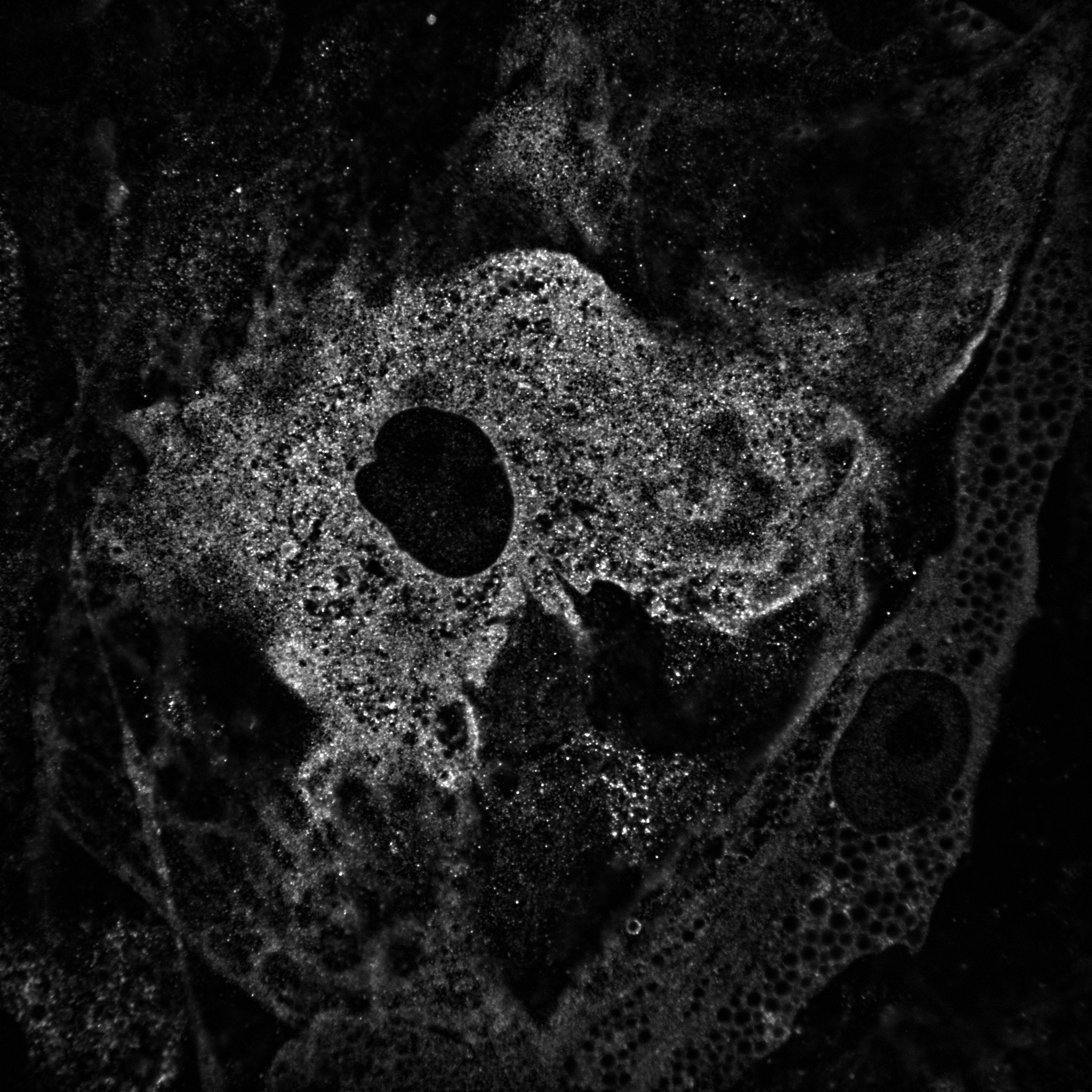

Supplement: Supplementary file 11 — Figure EV1-3 Source Data [file 44321_2024_188_MOESM11_ESM.zip › Expanded View 2/EV.2J/FABP4_Infected_48hr_100X.tif]

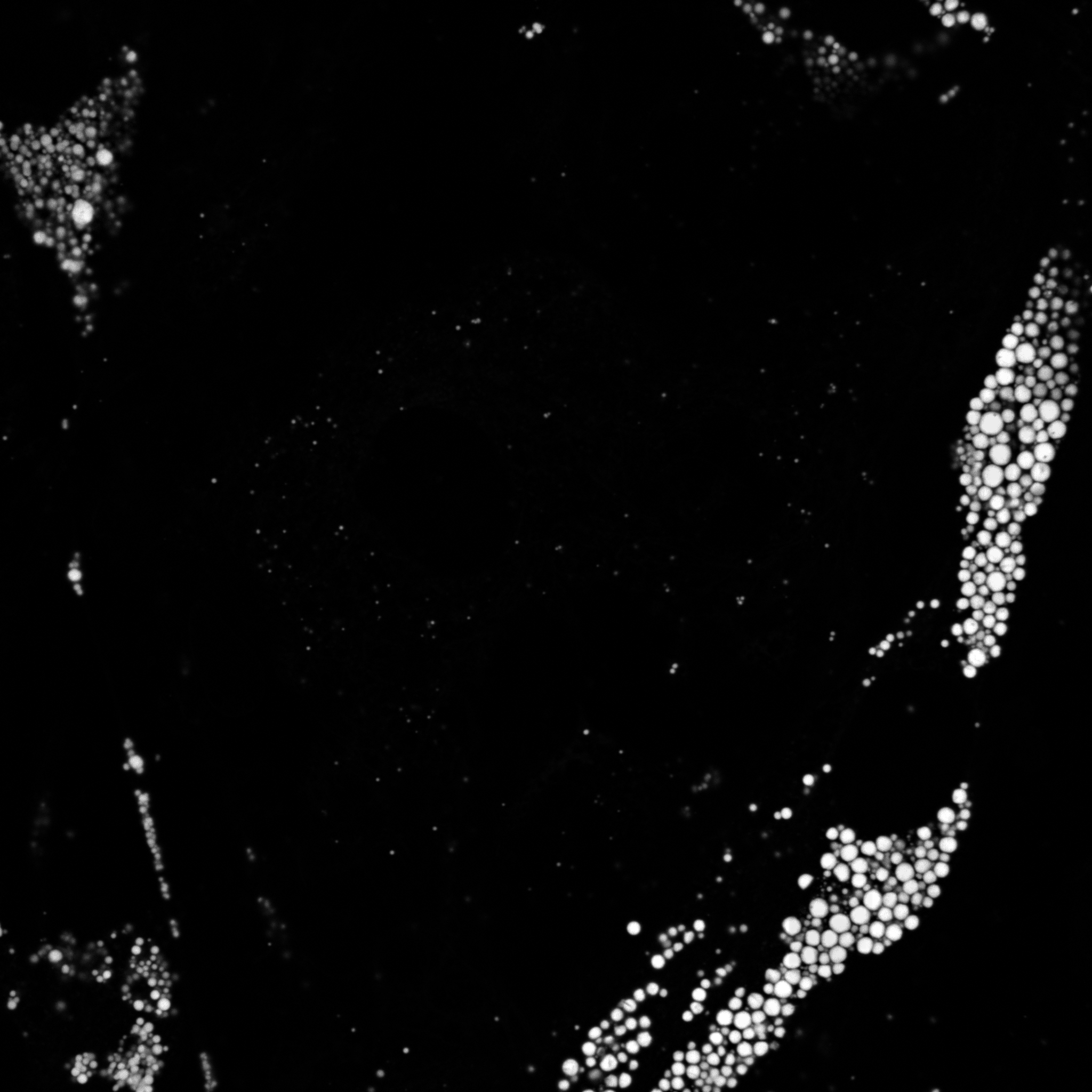

Supplement: Supplementary file 11 — Figure EV1-3 Source Data [file 44321_2024_188_MOESM11_ESM.zip › Expanded View 2/EV.2J/LDs_Infected_48hr_100X.tif]

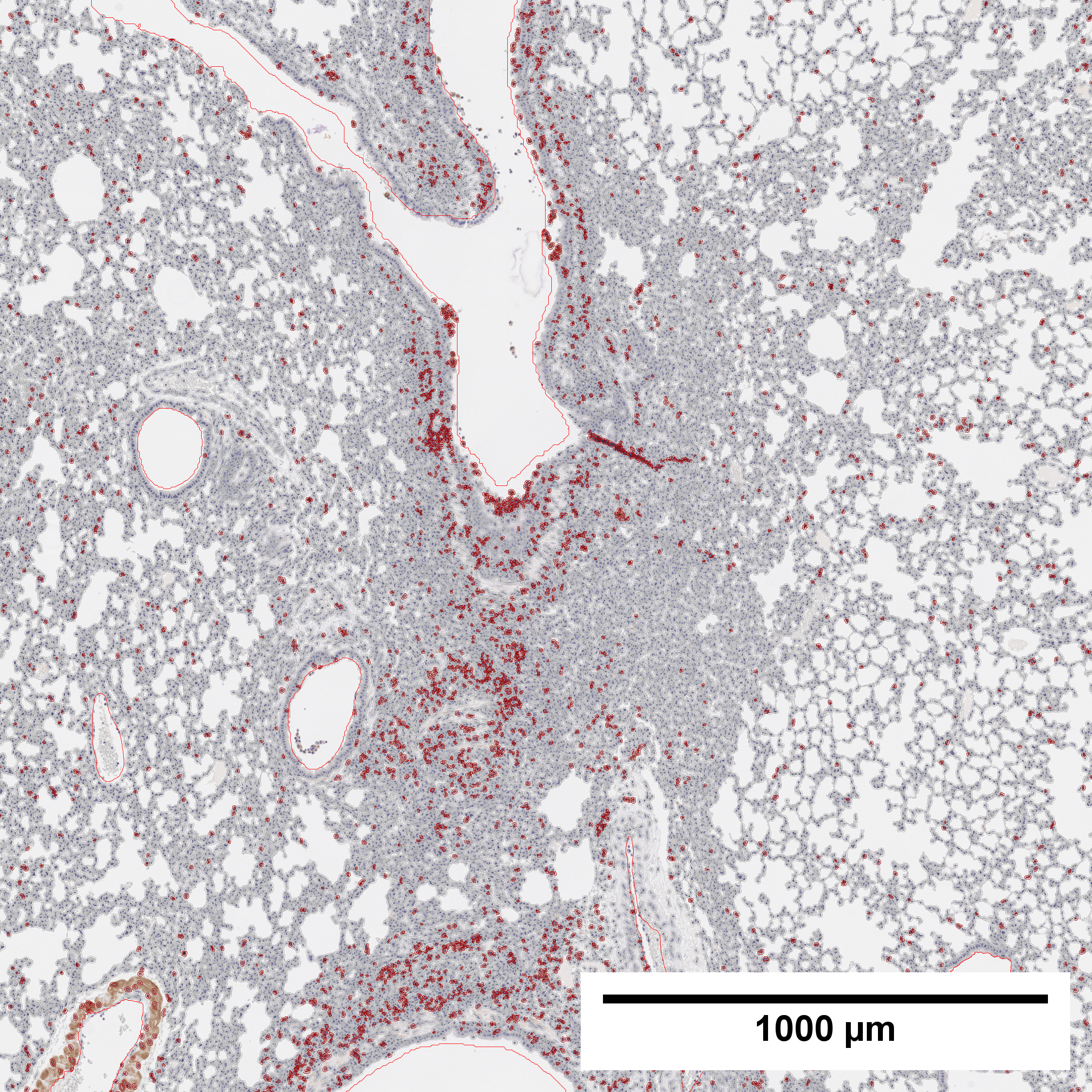

Supplement: Supplementary file 12 — Figure EV4-5 Source Data [file 44321_2024_188_MOESM12_ESM.zip › Expanded View 5/EV.5H-J/EV.5H_CD68_inf.CRE_3.png]

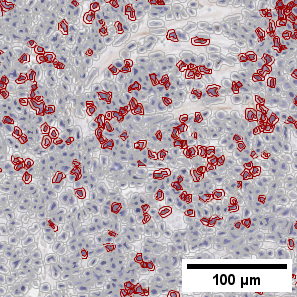

Supplement: Supplementary file 12 — Figure EV4-5 Source Data [file 44321_2024_188_MOESM12_ESM.zip › Expanded View 5/EV.5H-J/EV.5H_CD68_inf.CRE_3_overlay.png]

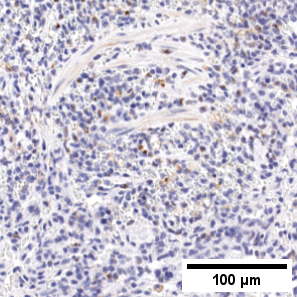

Supplement: Supplementary file 12 — Figure EV4-5 Source Data [file 44321_2024_188_MOESM12_ESM.zip › Expanded View 5/EV.5H-J/EV.5H_CD68_inf.CRE_3_crop.png]

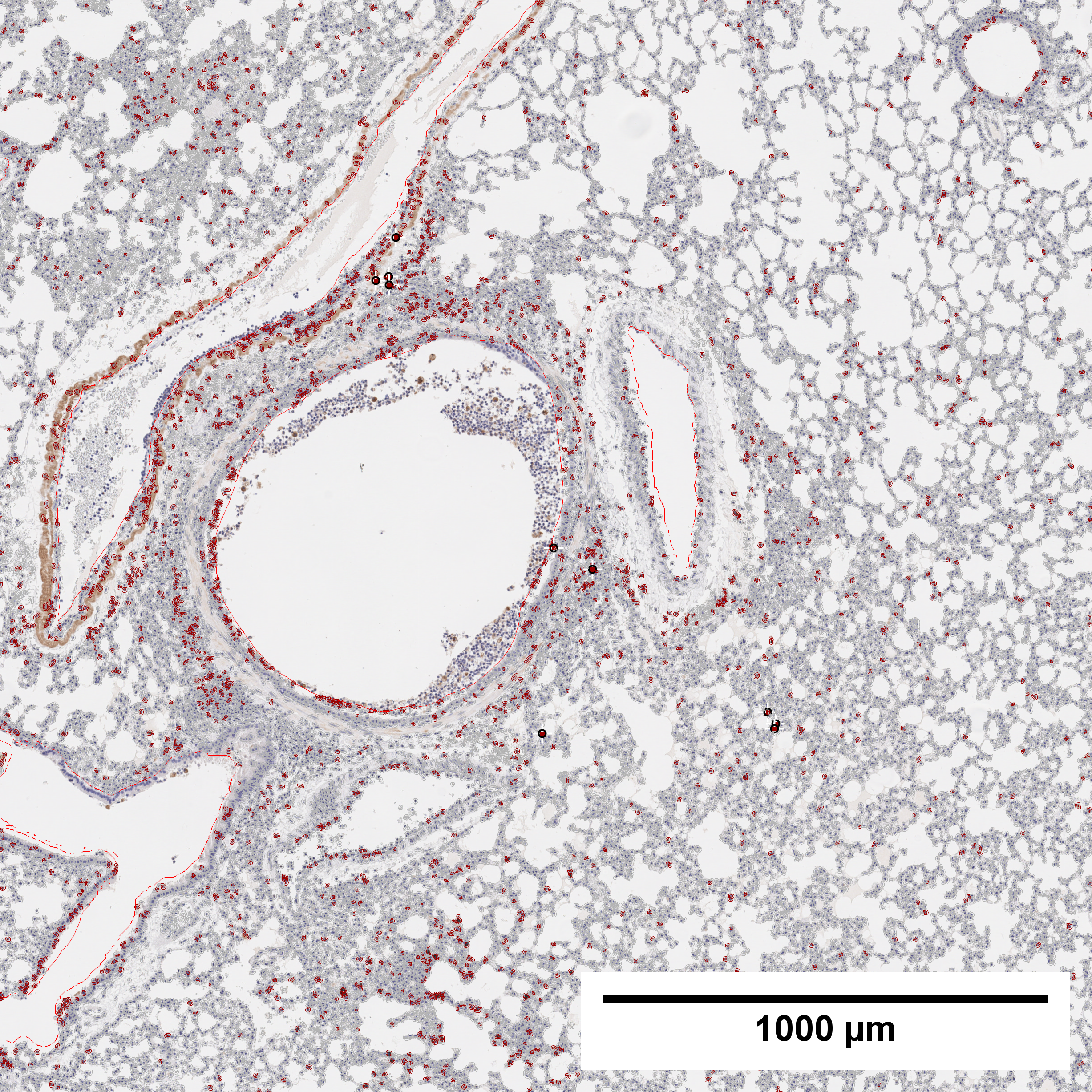

Supplement: Supplementary file 12 — Figure EV4-5 Source Data [file 44321_2024_188_MOESM12_ESM.zip › Expanded View 5/EV.5H-J/EV.5H_CD68_inf.Veh_3.png]

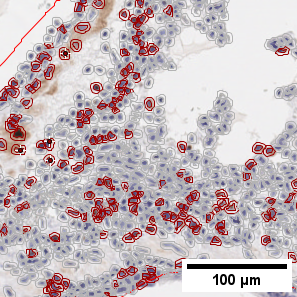

Supplement: Supplementary file 12 — Figure EV4-5 Source Data [file 44321_2024_188_MOESM12_ESM.zip › Expanded View 5/EV.5H-J/EV.5H_CD68_inf.Veh_3_overlay.png]

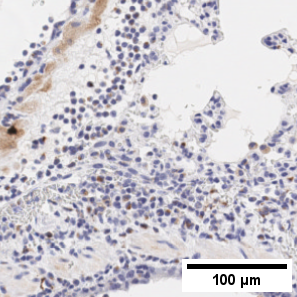

Supplement: Supplementary file 12 — Figure EV4-5 Source Data [file 44321_2024_188_MOESM12_ESM.zip › Expanded View 5/EV.5H-J/EV.5H_CD68_inf.Veh_3_crop.png]

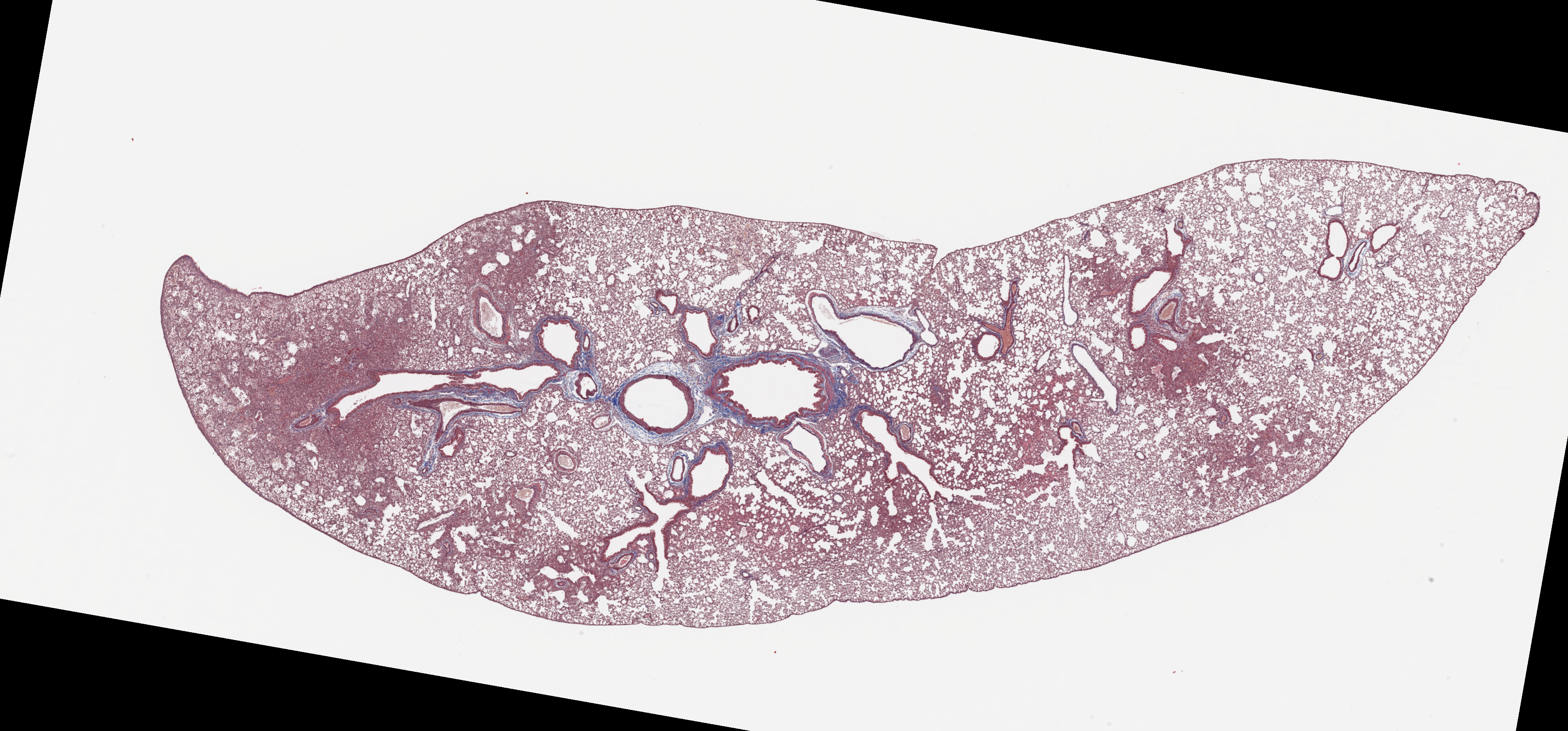

Supplement: Supplementary file 12 — Figure EV4-5 Source Data [file 44321_2024_188_MOESM12_ESM.zip › Expanded View 5/EV.5G/EV.5G_infected_CRE-14.png]

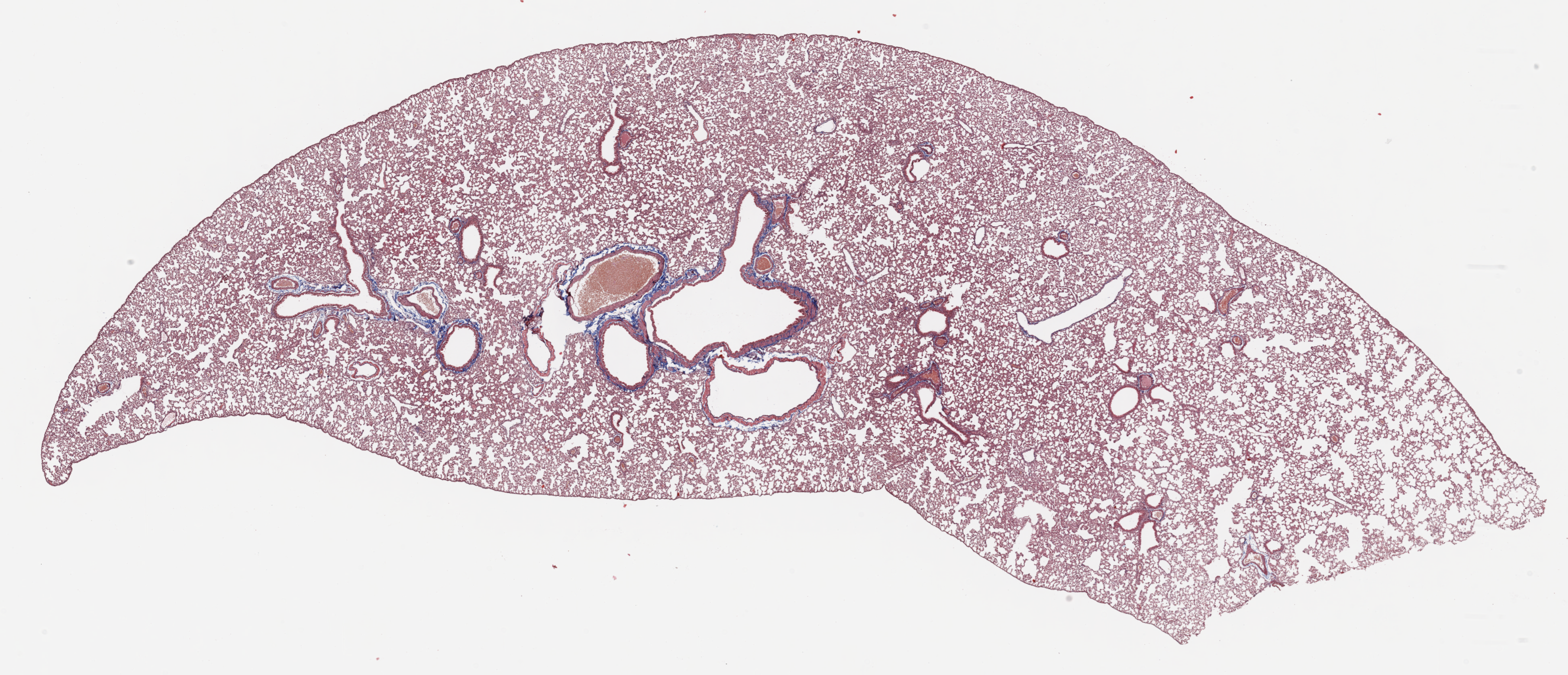

Supplement: Supplementary file 12 — Figure EV4-5 Source Data [file 44321_2024_188_MOESM12_ESM.zip › Expanded View 5/EV.5G/EV.5G_uninfected_vehicle.png]

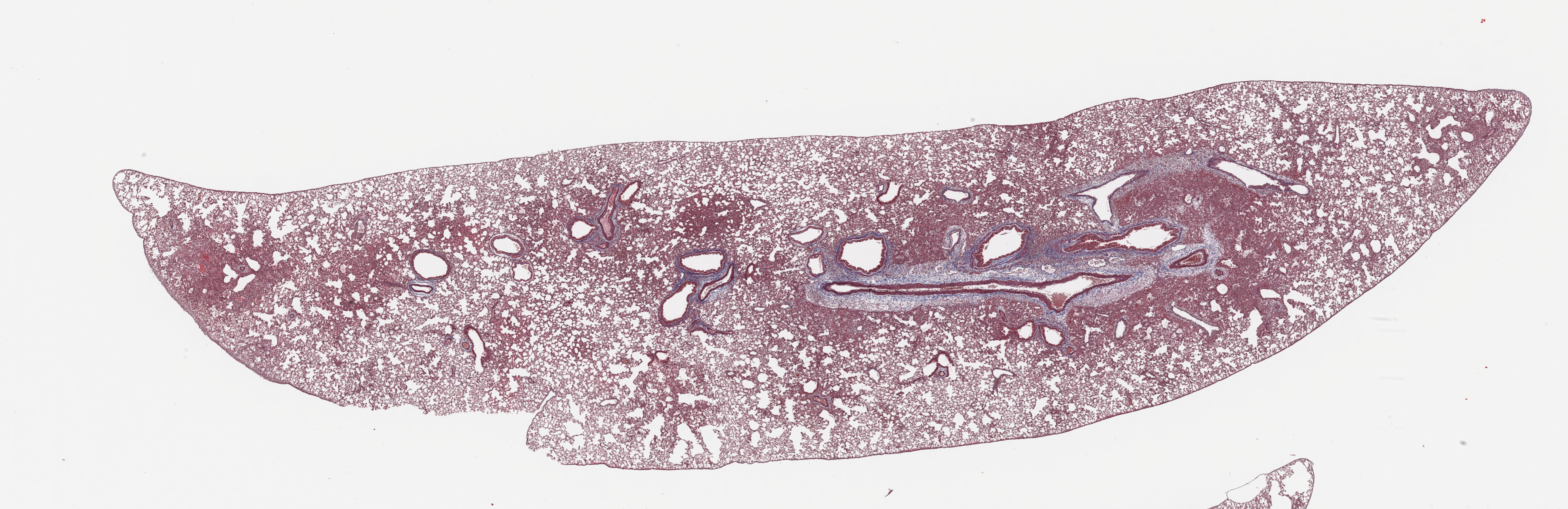

Supplement: Supplementary file 12 — Figure EV4-5 Source Data [file 44321_2024_188_MOESM12_ESM.zip › Expanded View 5/EV.5G/EV.5G_infected_vehicle.png]

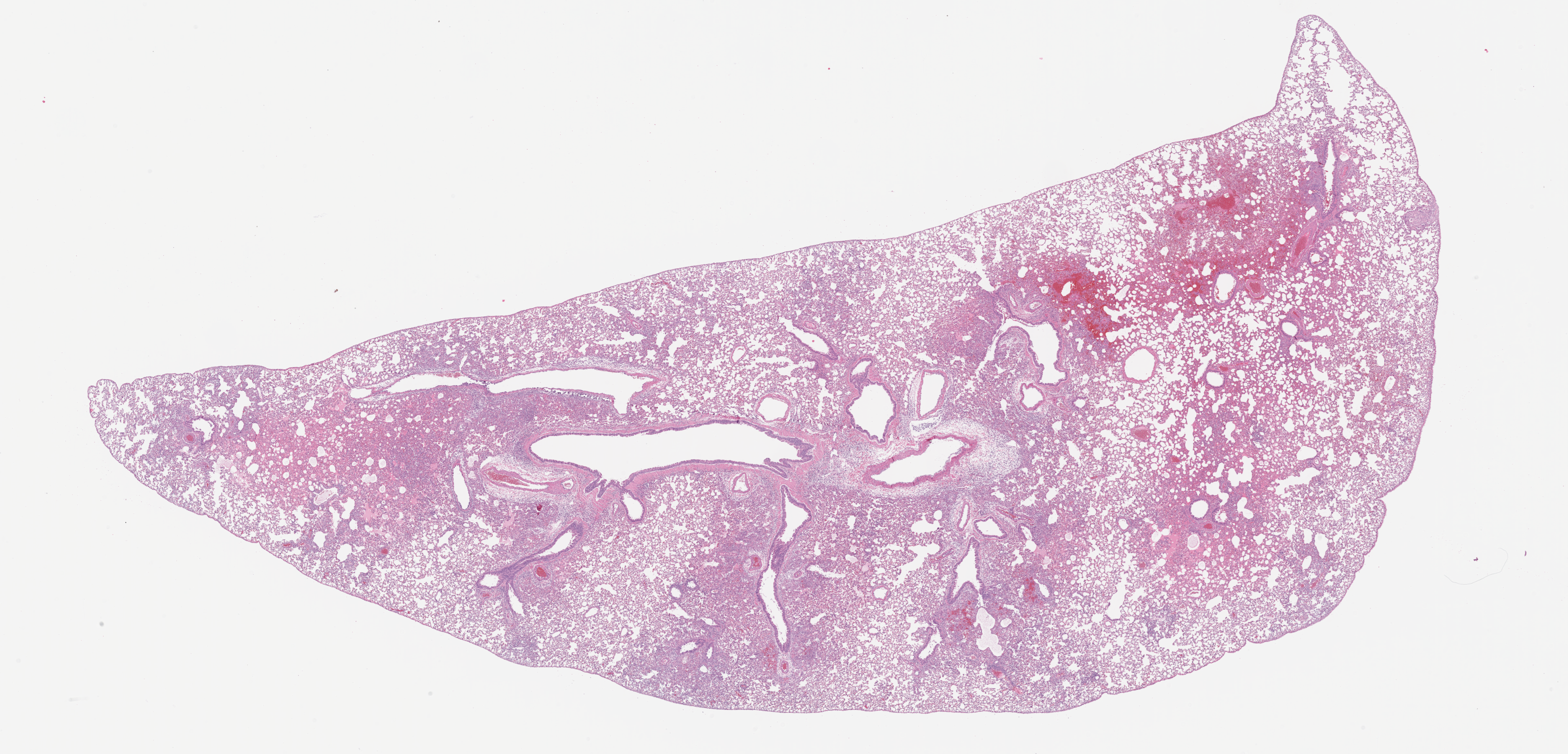

Supplement: Supplementary file 12 — Figure EV4-5 Source Data [file 44321_2024_188_MOESM12_ESM.zip › Expanded View 5/EV.5F/EV.5F_infected.vehicle.tif]

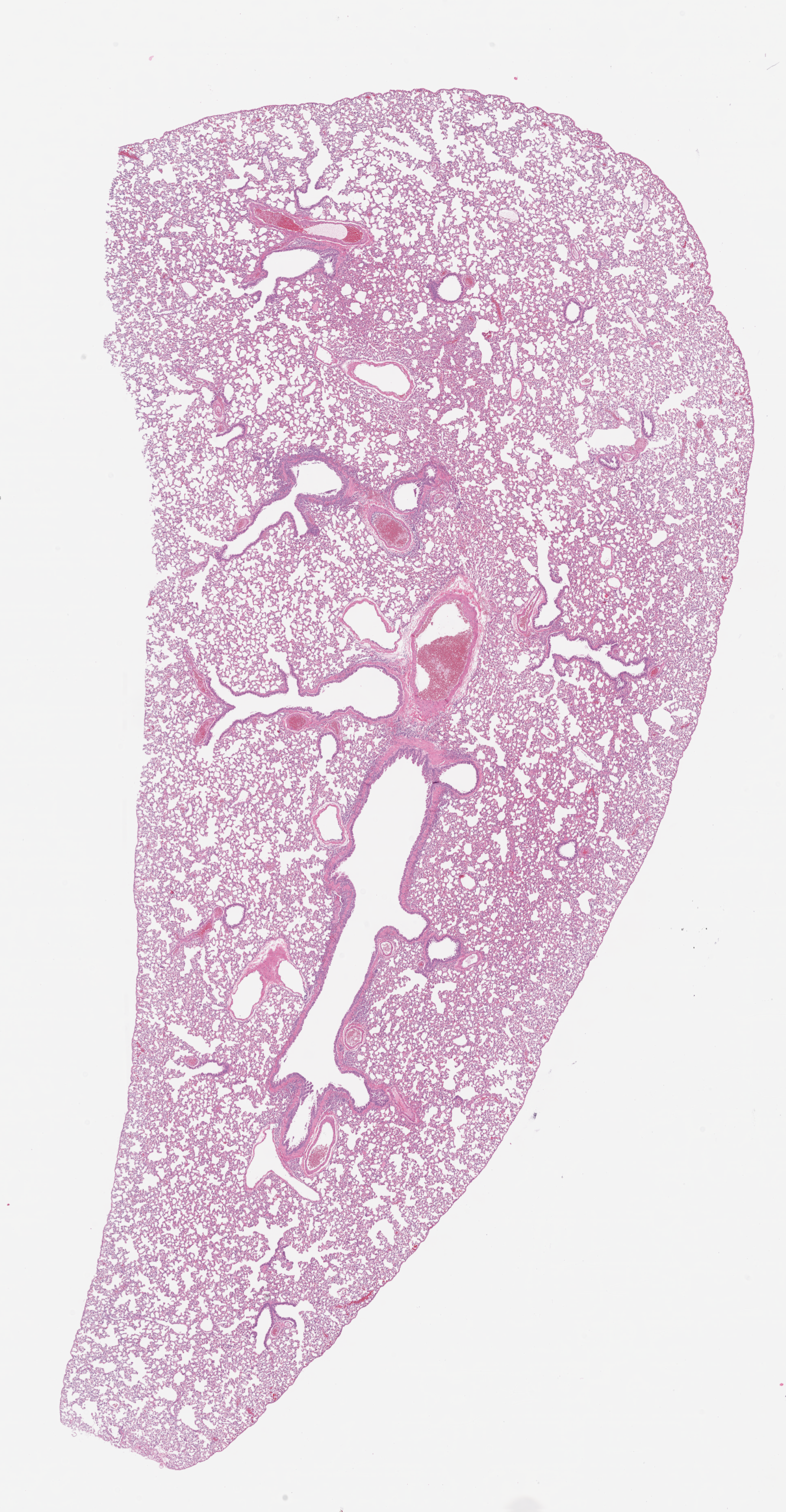

Supplement: Supplementary file 12 — Figure EV4-5 Source Data [file 44321_2024_188_MOESM12_ESM.zip › Expanded View 5/EV.5F/EV.5F_infected.CRE-14.tif]

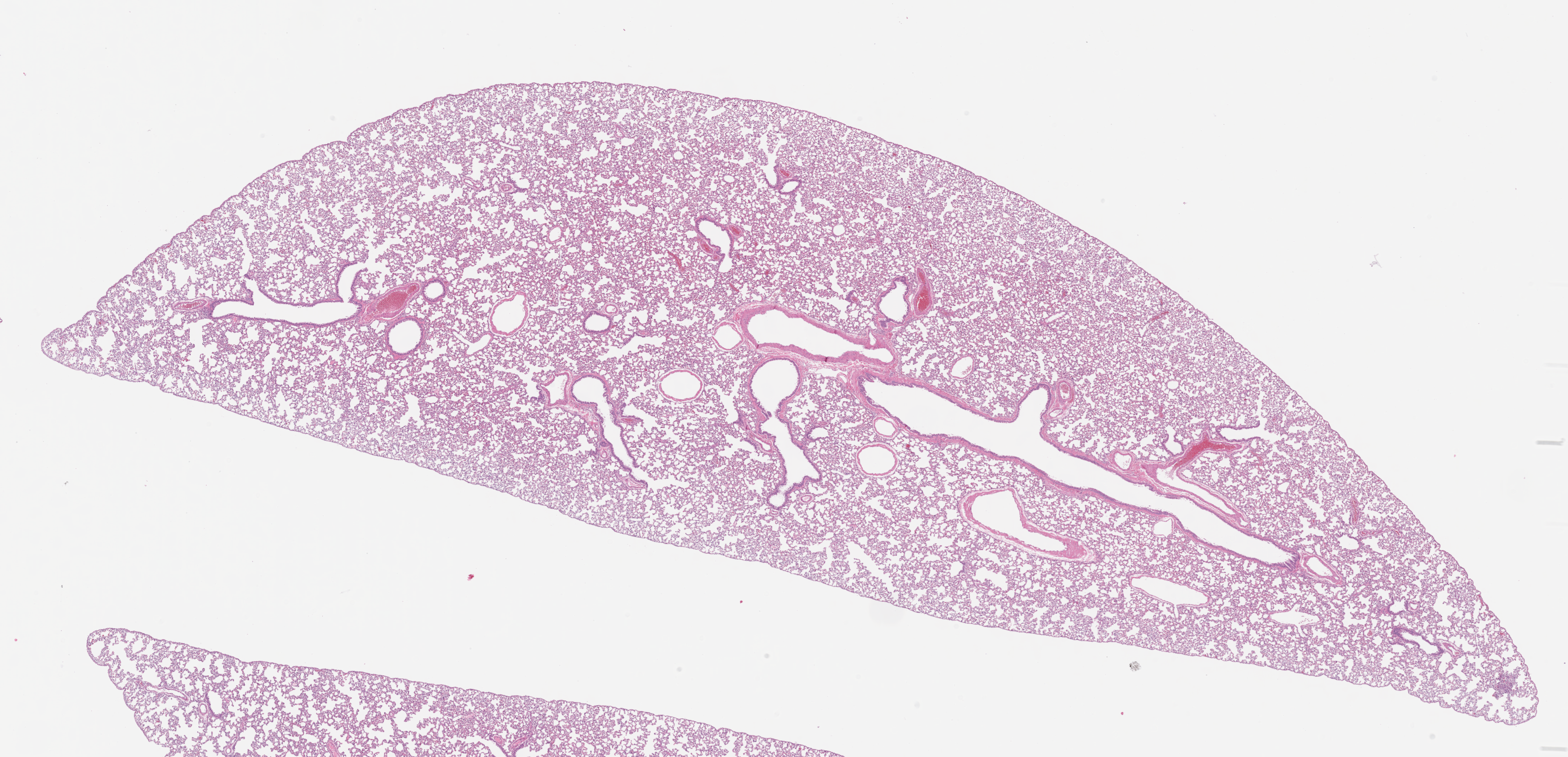

Supplement: Supplementary file 12 — Figure EV4-5 Source Data [file 44321_2024_188_MOESM12_ESM.zip › Expanded View 5/EV.5F/EV.5F_vehicle.tif]

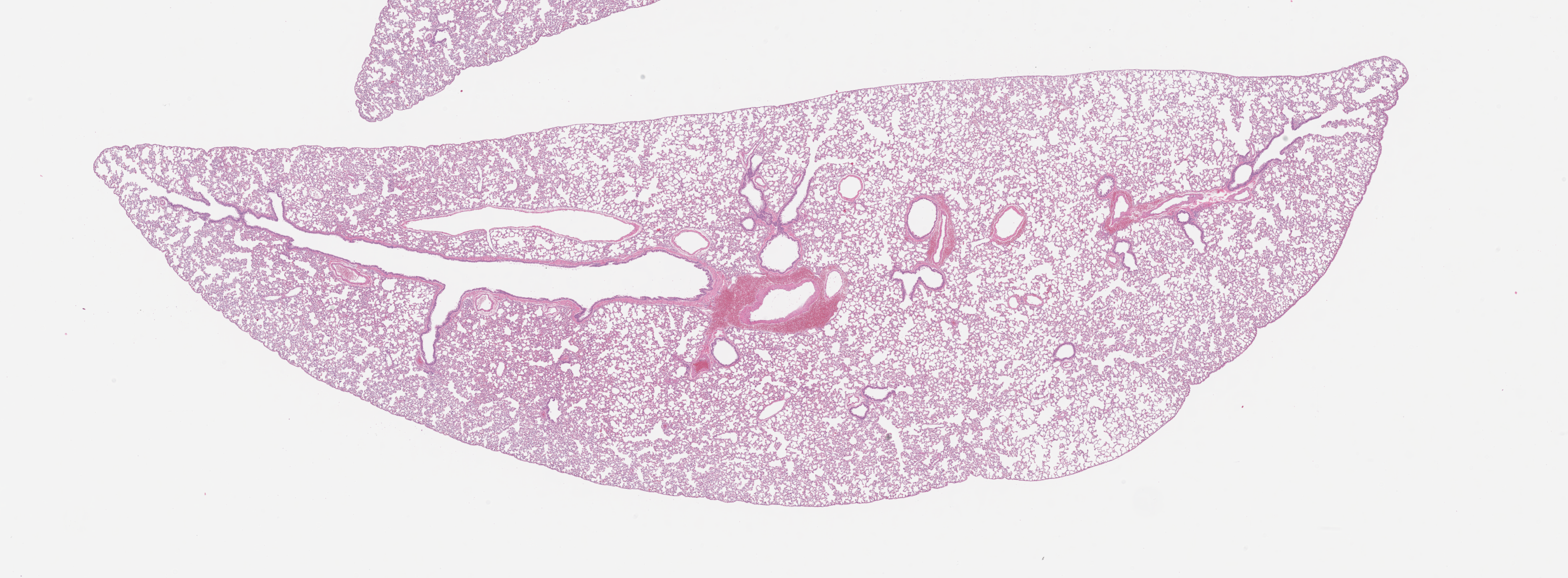

Supplement: Supplementary file 12 — Figure EV4-5 Source Data [file 44321_2024_188_MOESM12_ESM.zip › Expanded View 5/EV.5F/EV.5F_CRE-14.tif]

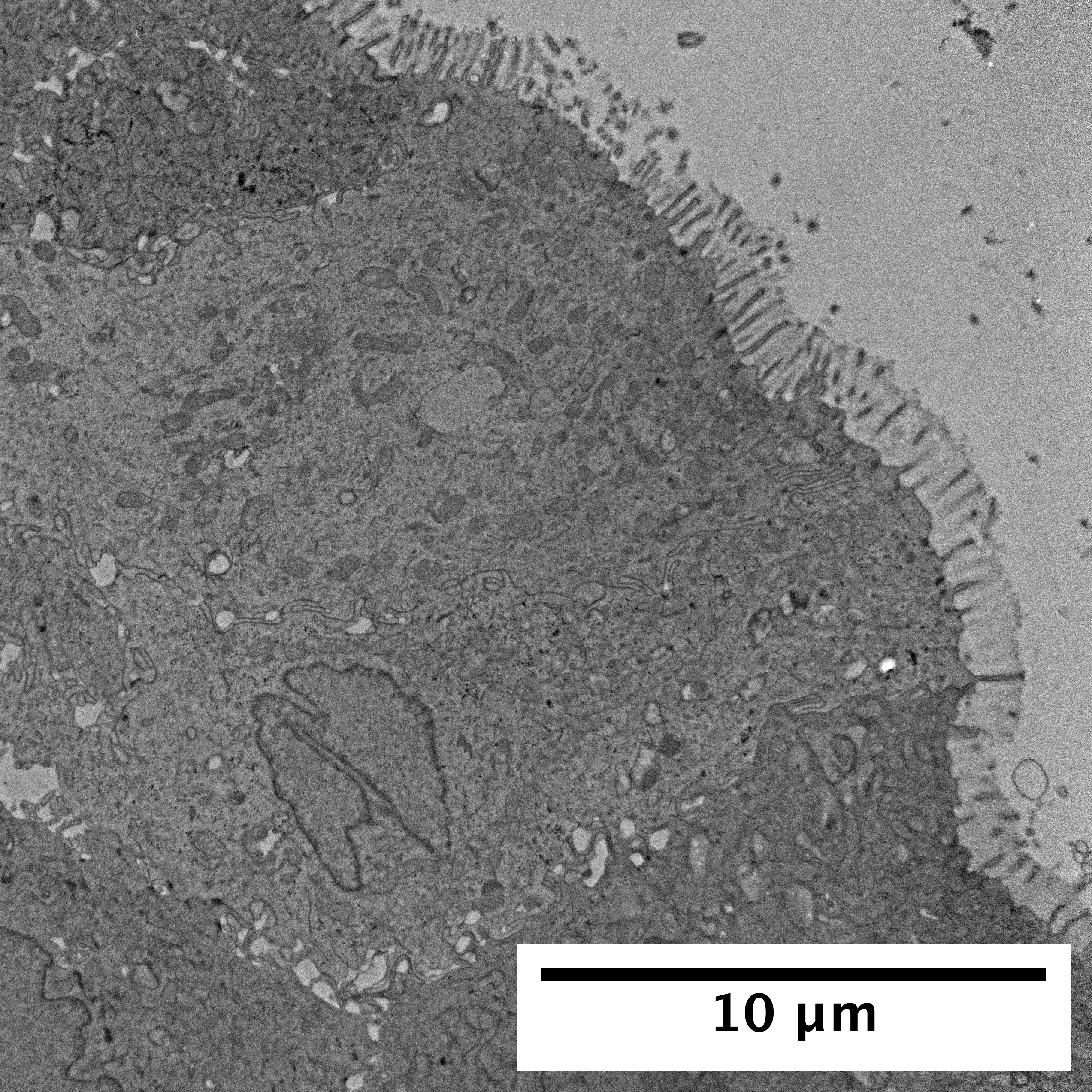

Supplement: Supplementary file 12 — Figure EV4-5 Source Data [file 44321_2024_188_MOESM12_ESM.zip › Expanded View 4/EV.4B/EV.4B_uninf_CRE14_2k.png]

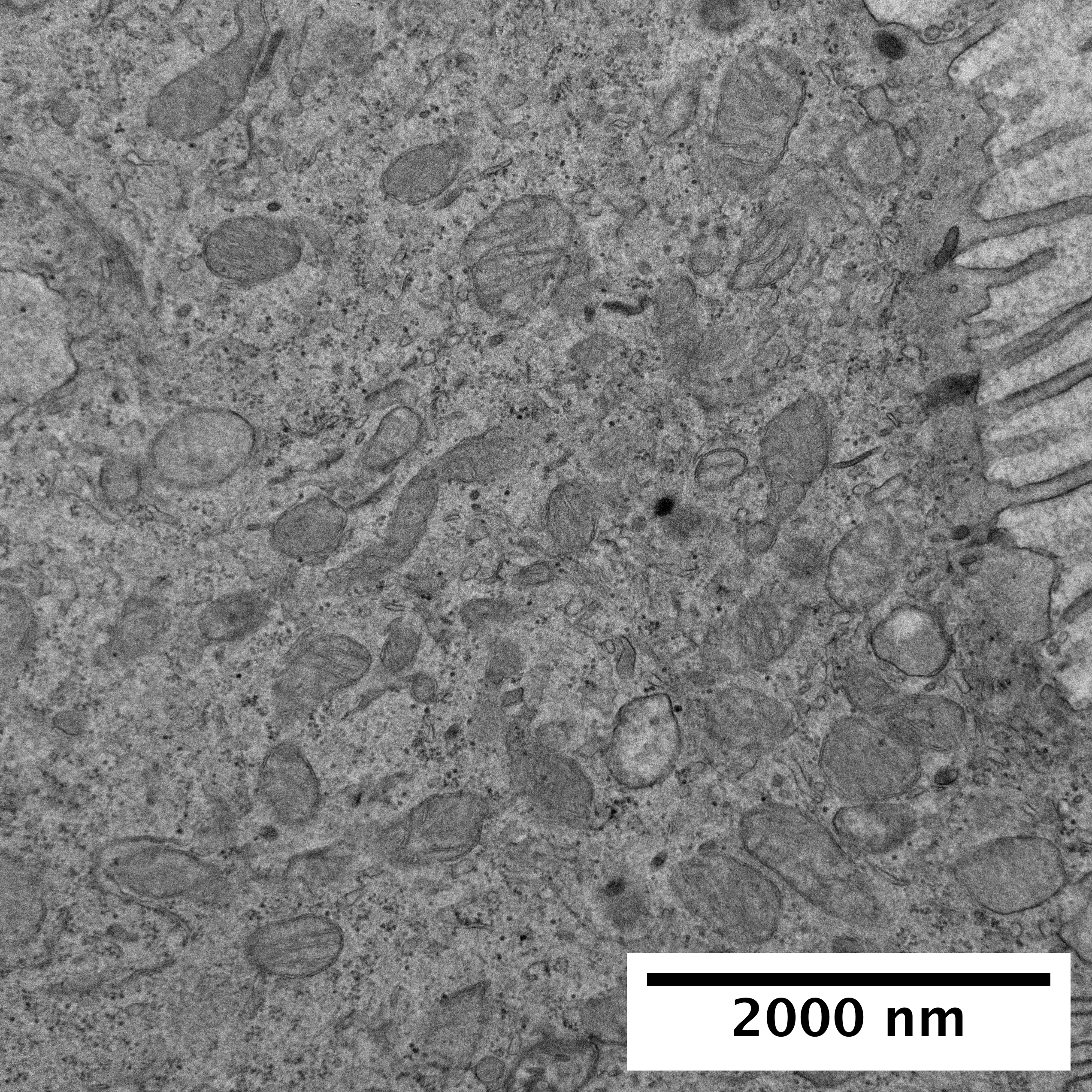

Supplement: Supplementary file 12 — Figure EV4-5 Source Data [file 44321_2024_188_MOESM12_ESM.zip › Expanded View 4/EV.4B/EV.4B_uninf_CRE14_x8K.png]

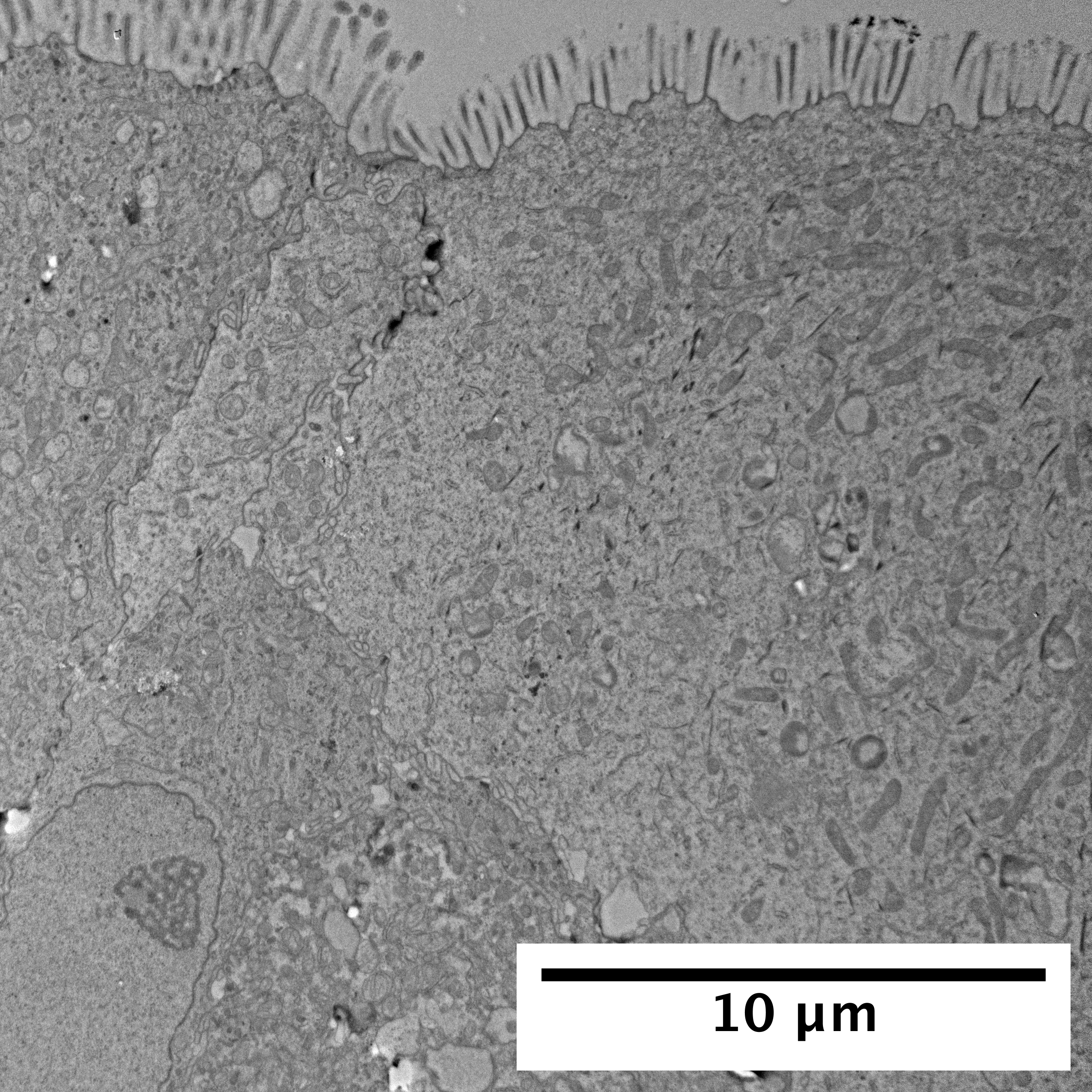

Supplement: Supplementary file 12 — Figure EV4-5 Source Data [file 44321_2024_188_MOESM12_ESM.zip › Expanded View 4/EV.4B/EV.4B_uninf_DMSO_x2K.png]

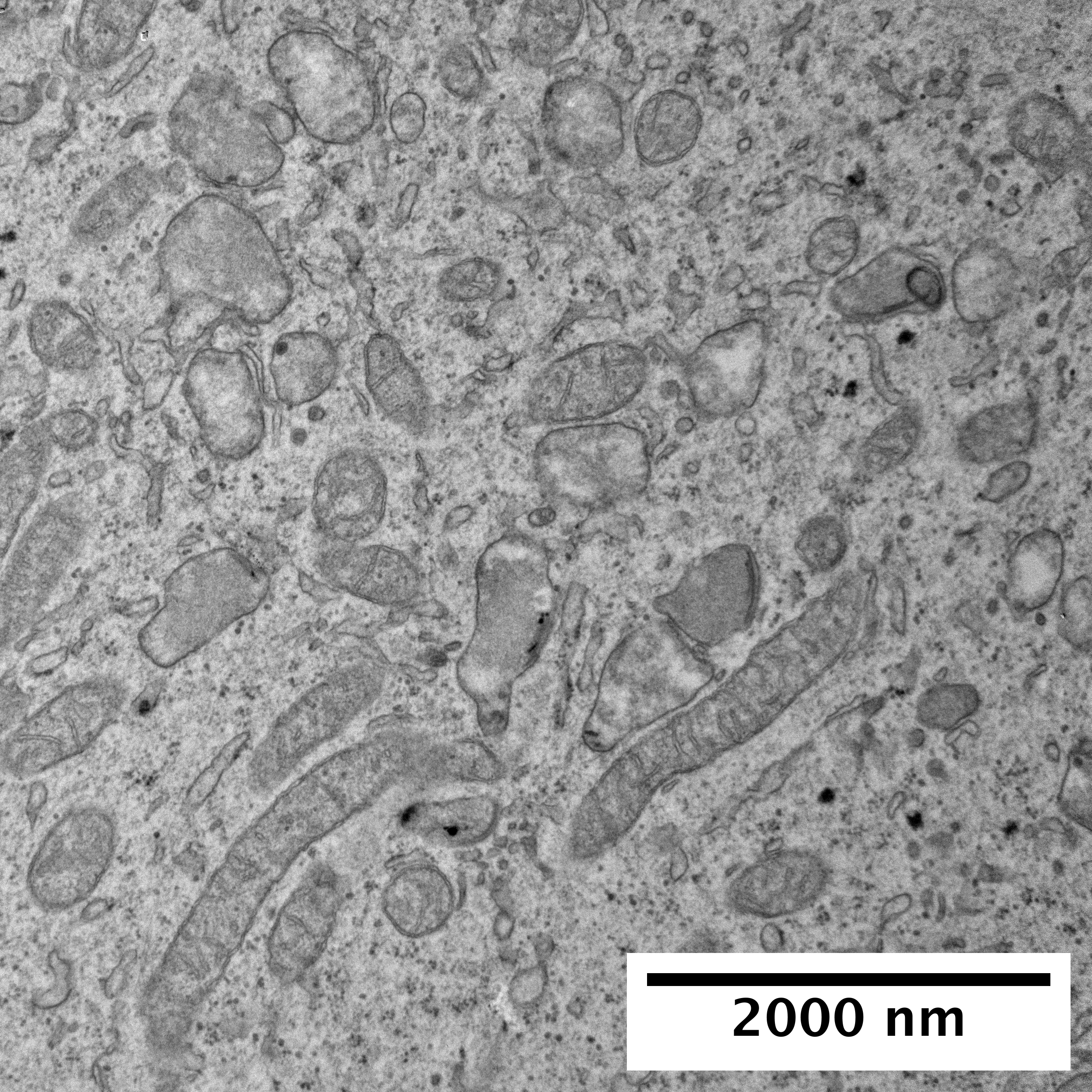

Supplement: Supplementary file 12 — Figure EV4-5 Source Data [file 44321_2024_188_MOESM12_ESM.zip › Expanded View 4/EV.4B/EV.4B_uninf_DMSO_x8K.png]

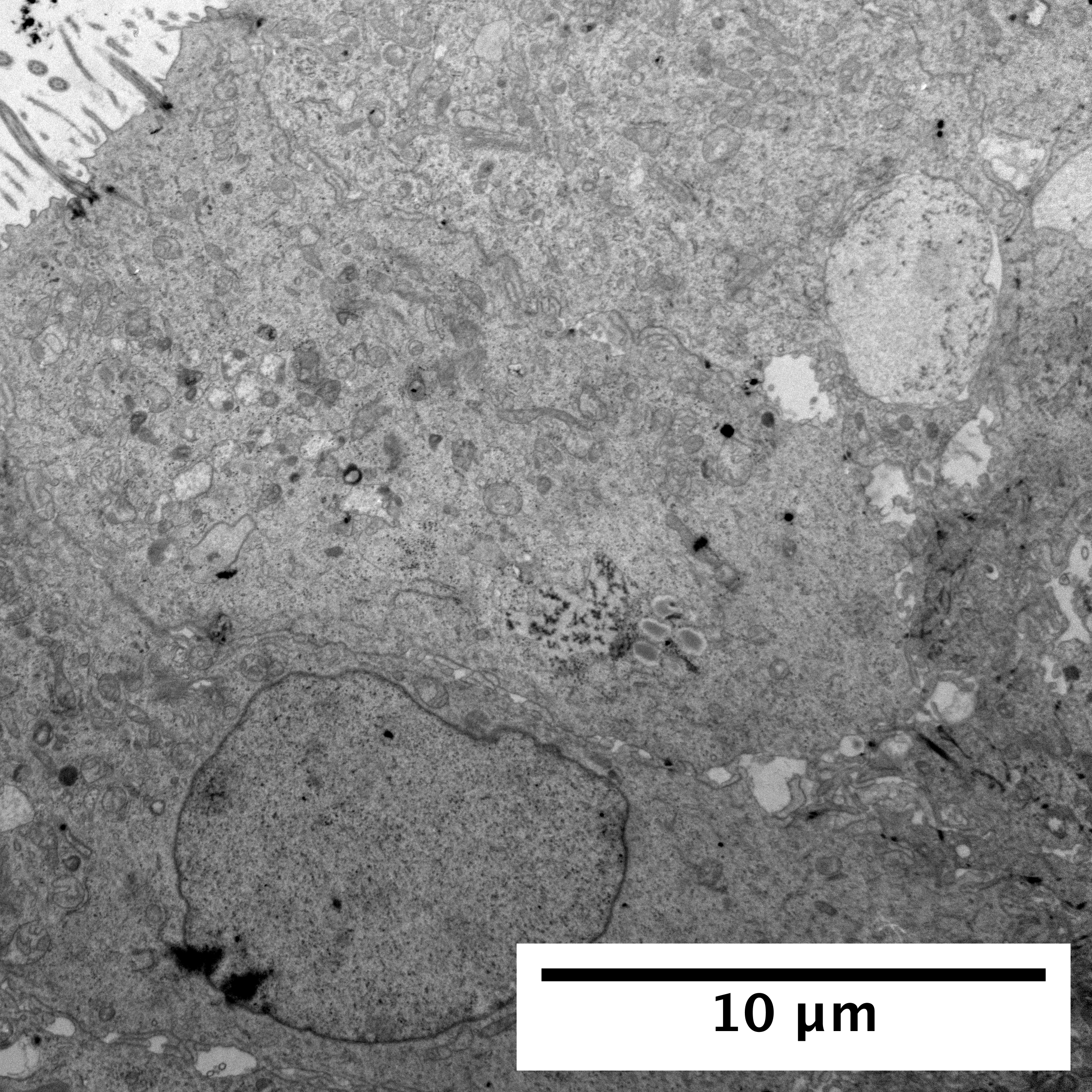

Supplement: Supplementary file 12 — Figure EV4-5 Source Data [file 44321_2024_188_MOESM12_ESM.zip › Expanded View 4/EV.4B/EV.4B_uninf_BMS_x2K.png]

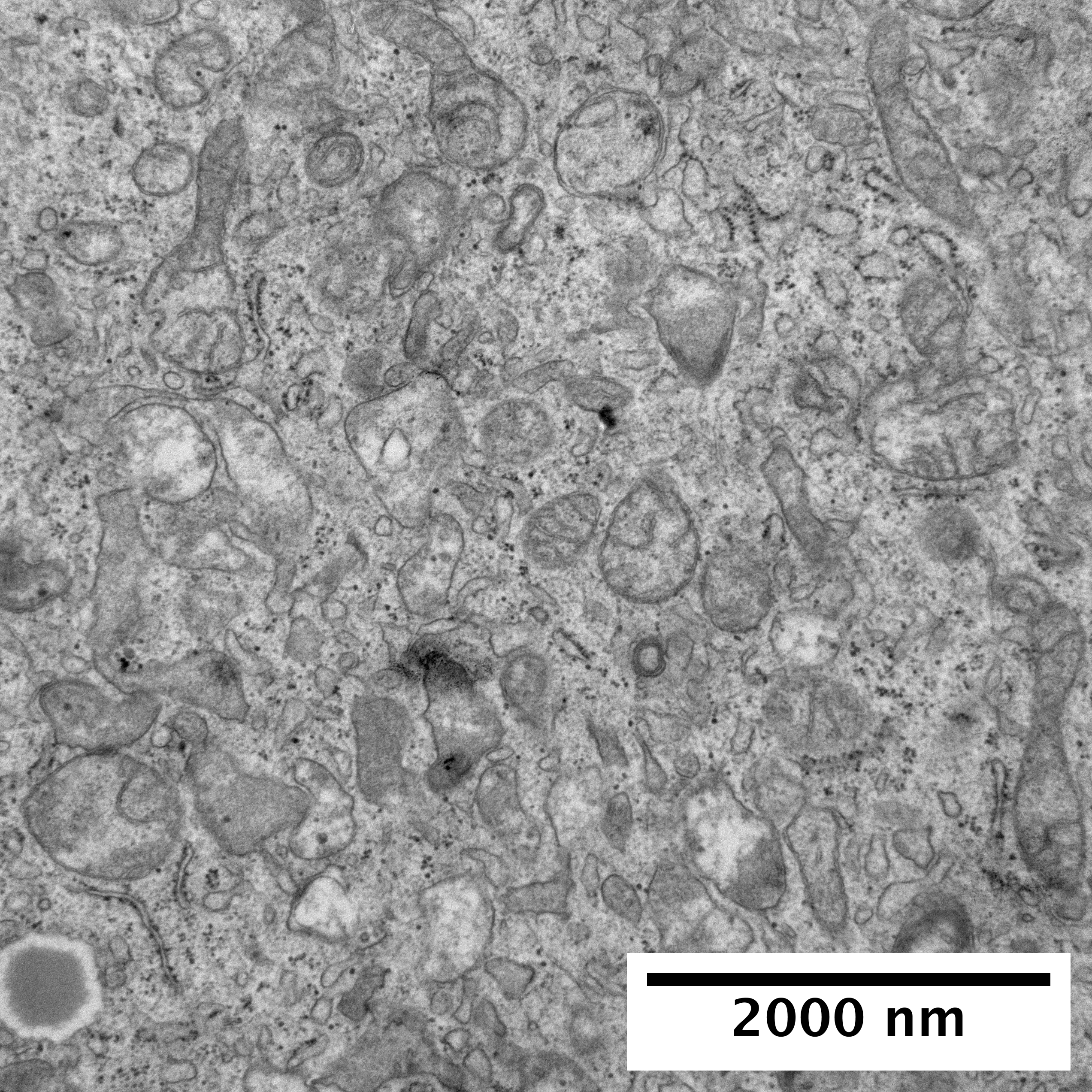

Supplement: Supplementary file 12 — Figure EV4-5 Source Data [file 44321_2024_188_MOESM12_ESM.zip › Expanded View 4/EV.4B/EV.4B_uninf_BMS_x8K.png]

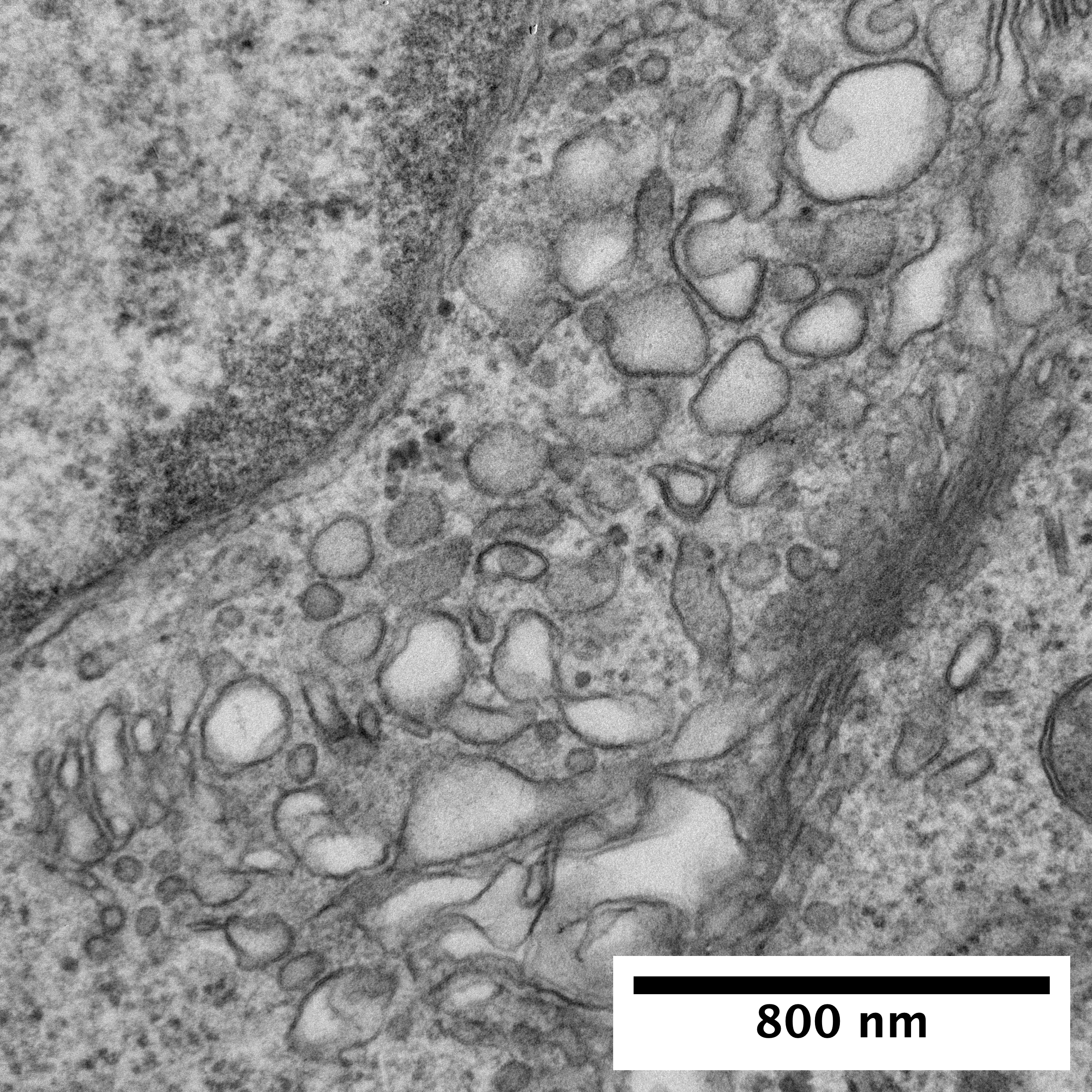

Supplement: Supplementary file 12 — Figure EV4-5 Source Data [file 44321_2024_188_MOESM12_ESM.zip › Expanded View 4/EV.4A/EV.4A_inf.CRE-14_high mag.png]

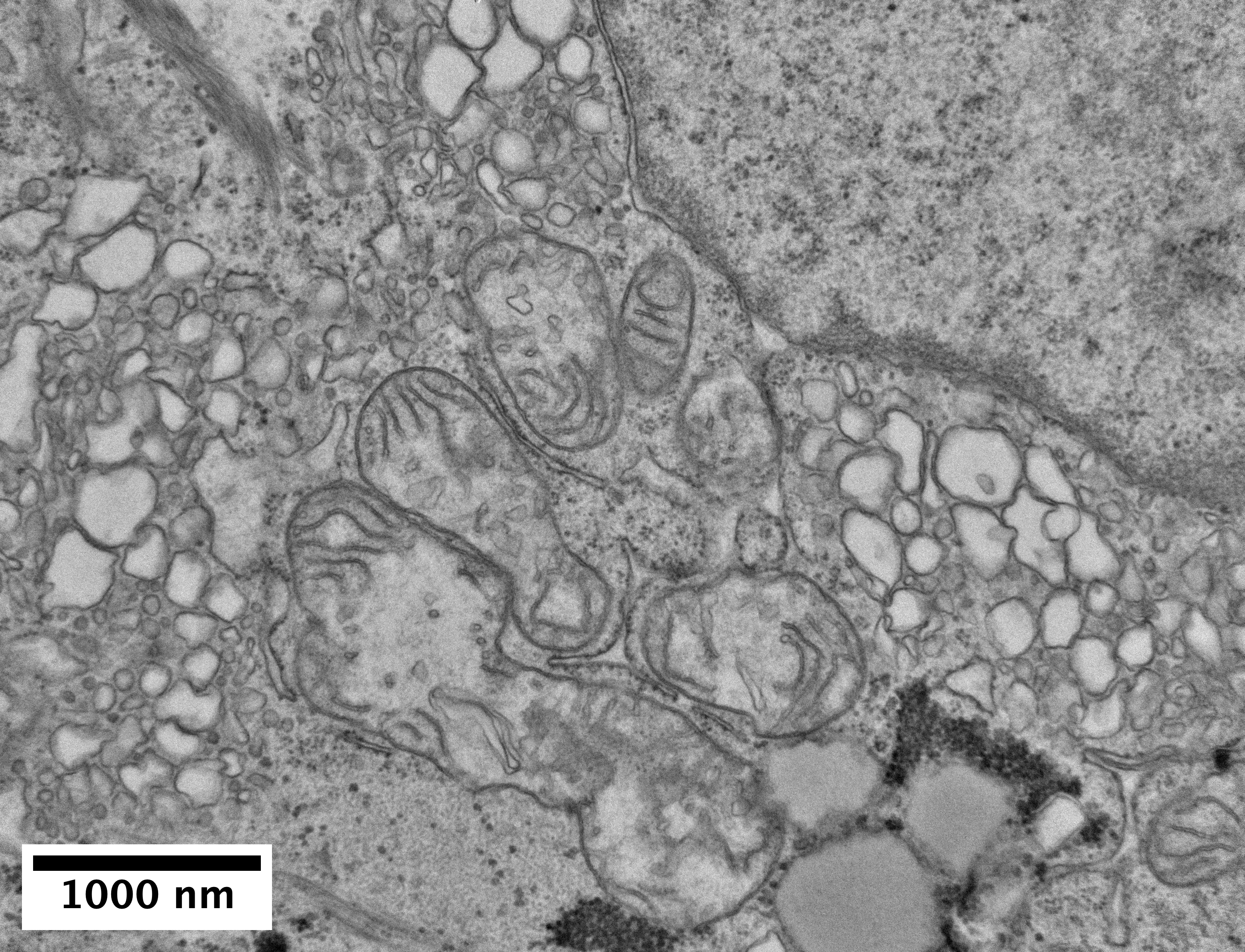

Supplement: Supplementary file 12 — Figure EV4-5 Source Data [file 44321_2024_188_MOESM12_ESM.zip › Expanded View 4/EV.4A/EV.4A_inf.DMSO_low mag.png]

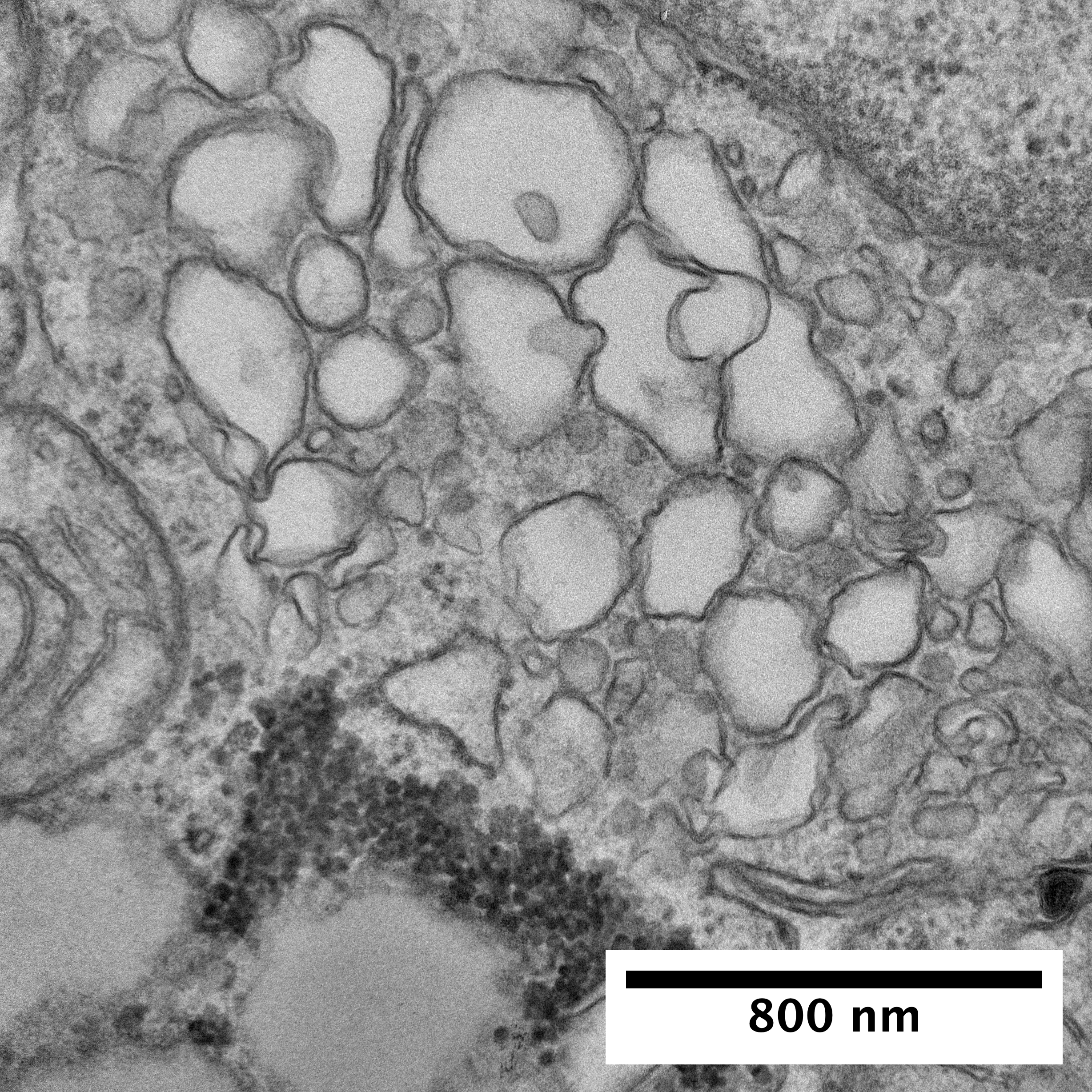

Supplement: Supplementary file 12 — Figure EV4-5 Source Data [file 44321_2024_188_MOESM12_ESM.zip › Expanded View 4/EV.4A/EV.4A_inf.DMSO_high mag.png]

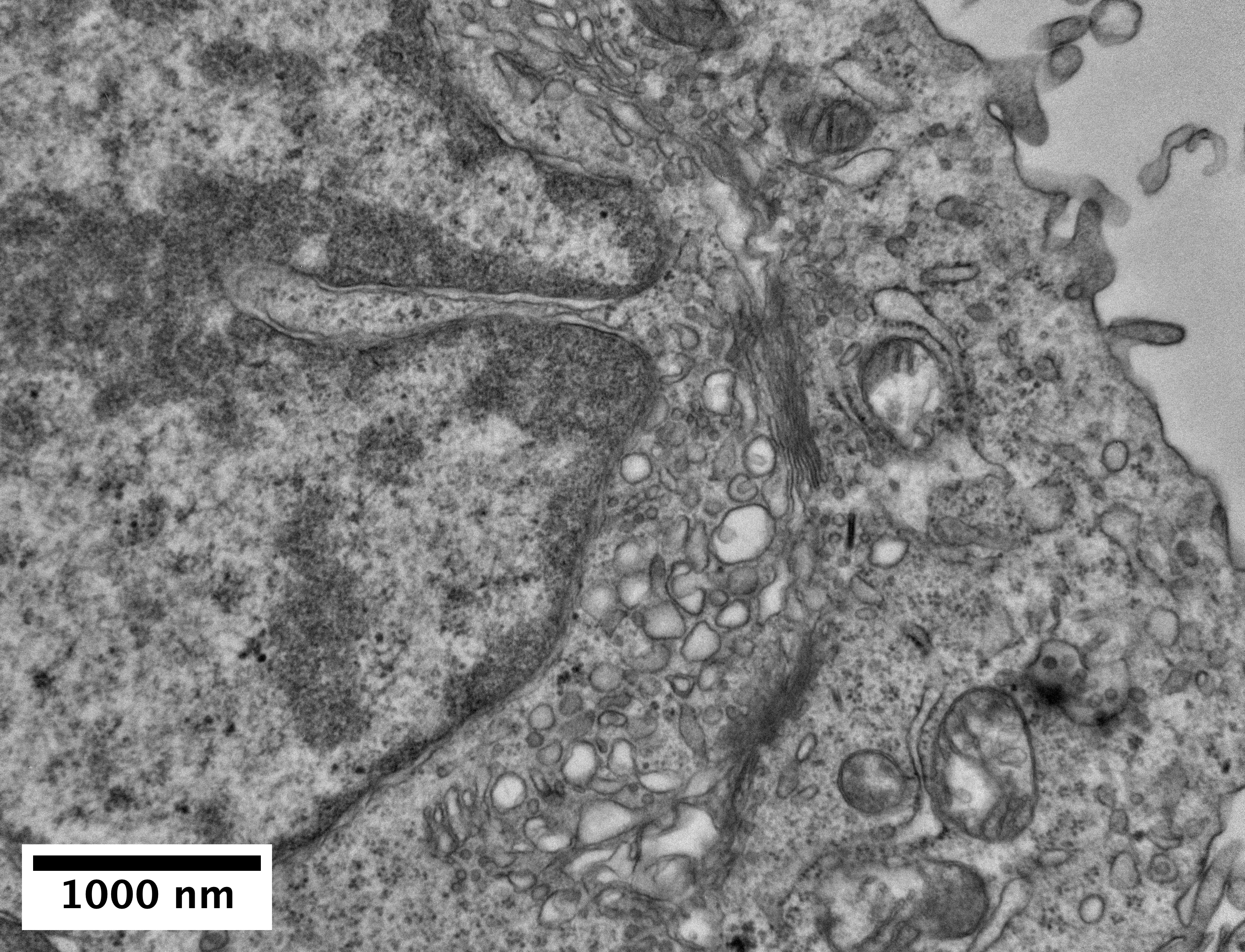

Supplement: Supplementary file 12 — Figure EV4-5 Source Data [file 44321_2024_188_MOESM12_ESM.zip › Expanded View 4/EV.4A/EV.4A_inf.CRE-14_low mag.png]
