# Supplementary material for: FABP4 as a therapeutic host target controlling SARS-CoV-2 infection
Source: EMBO Mol Med. 2025 Jan 22;17(3):414–40. doi: 10.1038/s44321-024-00188-x (PMC11904229; doi:10.1038/s44321-024-00188-x)
Supplement: Supplementary file 13 — Expanded View Figures [file 44321_2024_188_MOESM13_ESM.pdf]

Expanded View Figures

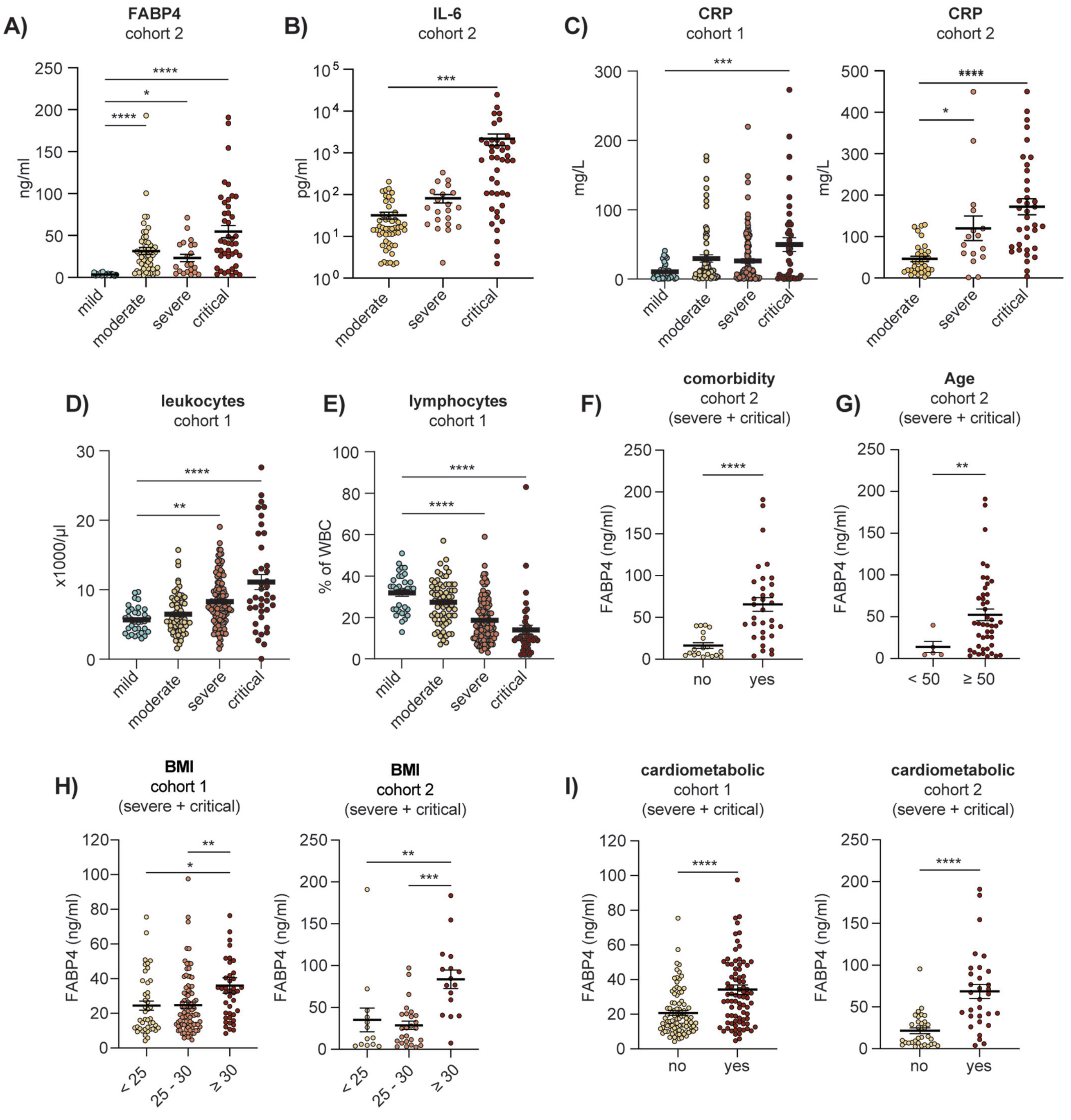

Figure EV1. Increase in FABP4 along with biomarkers of COVID-19 disease severity.

(**A**, **B**) Maximum concentrations of circulating (**A**) FABP4 (****$p < 0.0001$, *$p = 0.044$) and (**B**) IL-6 (***$p = 0.0005$) of cohort 2 of COVID-19 patients ($n = 166$) stratified based on disease severity (moderate: $n = 52$, severe: $n = 21$, and critical: $n = 42$). (**C**) Circulating levels of C-reactive protein (****$p < 0.0001$, ***$p = 0.0002$, *$p = 0.0221$), (**D**) leukocytes (****$p < 0.0001$, **$p = 0.0015$) and (**E**) lymphocytes (****$p < 0.0001$) of COVID-19 patients measured on the day in which the maximum FABP4 concentration was measured (day post symptom onset). Statistical analysis was performed using one-way ANOVA ($n = 283$ cohort 1, and $n = 116$ cohort 2). (**F**-**I**) Maximum FABP4 concentration pooled from severe and critically ill patients (cohort 1: $n = 176$, cohort 2: $n = 63$), stratified based on (**F**) the presence or absence of comorbidities (listed in Table 3 and Dataset EV2, ****$p < 0.0001$), (**G**) age (**$p = 0.0011$), (**H**) BMI (***$p = 0.0001$, **$p = 0.0098$ and 0.004, *p 0.0263), and (**I**) the presence or absence of cardiometabolic conditions (diabetes, hypertension, or coronary artery disease, ****$p < 0.0001$). Statistical analysis for (**F**), (**G**) and (**I**) were performed using Welch's t-test and one-way ANOVA for (**H**). Data are shown as the mean ± s.e.m. Source data are available online for this figure.

© The Author(s)

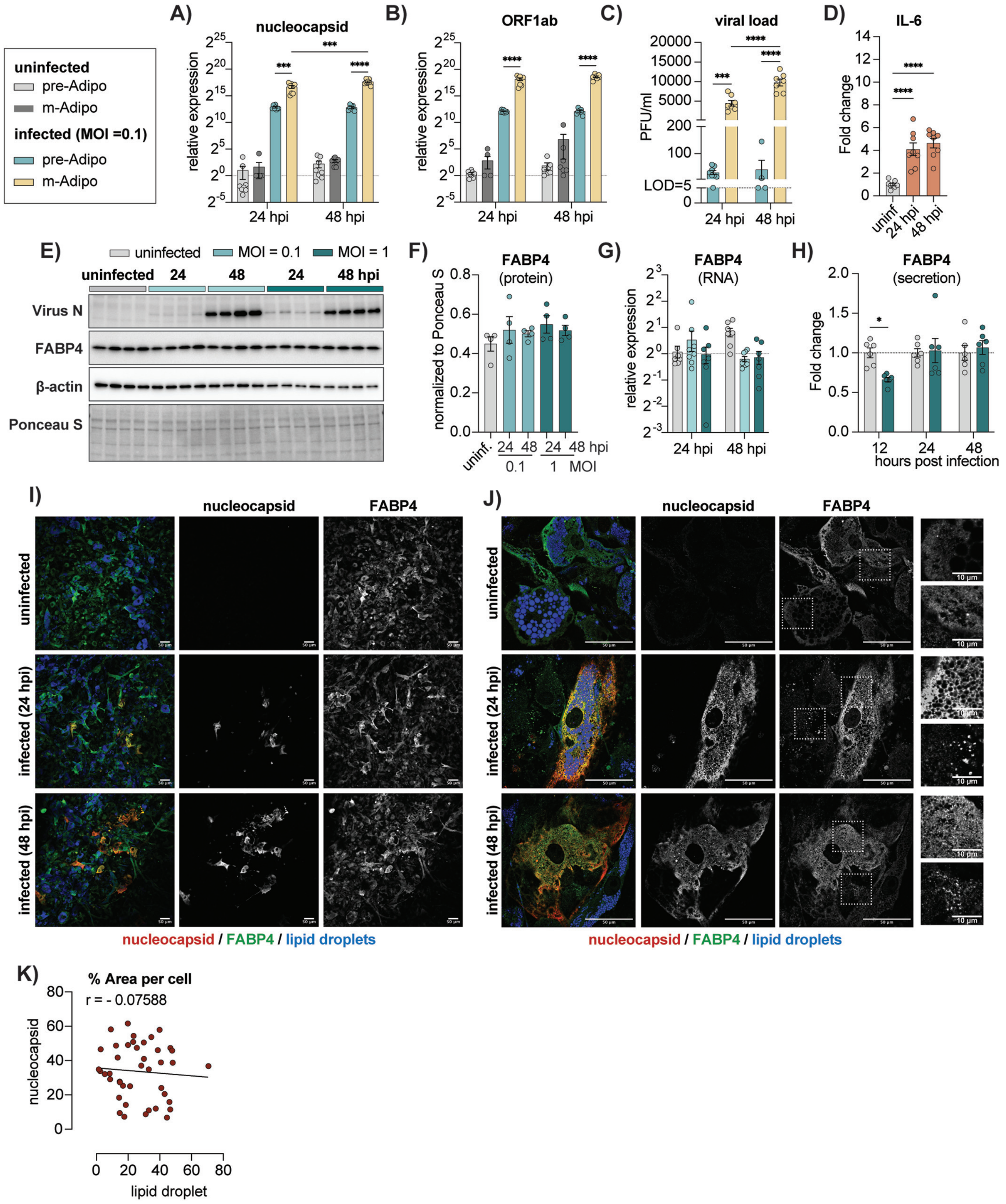

A)
nucleocapsid
relative expression
24 hpi
48 hpi
uninfected
pre-Adipo
m-Adipo
infected (MOI =0.1)
pre-Adipo
m-Adipo
B)
ORF1ab
C)
viral load
PFU/ml
LOD=5
D)
IL-6
Fold change
uninf
24 hpi
48 hpi
E)
uninfected
MOI = 0.1
MOI = 1
48 hpi
Virus N
FABP4
β-actin
Ponceau S
F)
FABP4
(protein)
normalized to Ponceau S
uninf.
MOI
G)
FABP4
(RNA)
H)
FABP4
(secretion)
hours post infection
I)
nucleocapsid
FABP4
uninfected
infected (24 hpi)
infected (48 hpi)
nucleocapsid / FABP4 / lipid droplets
J)
10 µm
K)
% Area per cell
r = - 0.07588
nucleocapsid
lipid droplet

 © The Author(s)

Figure EV2. FABP4 regulation during SARS-CoV-2 infection.

(**A–D**) Pre-adipocytes and differentiated adipocytes infected with SARS-CoV-2 (WA1/2020, MOI = 0.1). (**A**, **B**) Relative expression of viral (**A**) genomic RNA (nucleocapsid, ****$p < 0.0001$, ***$p = 0.0003$ and 0.0004) and (**B**) sub-genomic RNA (ORF1ab, ****$p < 0.0001$), normalized to β-actin. (**C**) Viral loads measured from supernatant using plaque assay (****$p < 0.0001$,***$p = 0.0001$). Data are pooled from two independent experiments ($n = 8$, biological replicates). Statistical analysis was performed using two-way ANOVA. (**D**) IL-6 measured by ELISA in the supernatant of differentiated adipocytes with or without virus infection (MOI = 0.1). Data are pooled from two independent experiments ($n = 8$, biological replicates, ****$p < 0.0001$). Statistical analysis was performed using one-way ANOVA. (**E**) Western blots of SARS-CoV-2 nucleocapsid, FABP4, β-actin protein levels, and total protein (Ponceau S staining) in cell lysates of differentiated adipocytes infected with SARS-CoV-2 (MOI = 0.1 or MOI = 1). (**F**) Quantification of FABP4 band intensity normalized to total protein, representative of two independent experiments ($n = 4$, biological replicates). (**G**) FABP4 gene expression relative to β-actin, pooled from two independent experiments ($n = 8$, biological replicates). (**H**) FABP4 secretion in the supernatant within 1-hour incubation at the indicated time points following infection. Fold change is calculated relative to uninfected samples. Data are representative of two independent experiments ($n = 6$, biological replicates, *$p = 0.0336$). Statistical analysis was performed using two-way ANOVA. (**I**, **J**) Representative confocal images of infected adipocytes stained with nucleocapsid (red), FABP4 (green), and lipid droplets (blue). (**I**) Low magnification and (**J**) high magnification images of the same samples (Scale bars = 50 μm, magnified regions = 10 μm) ($n = 3$, biological replicates). (**K**) Percentage lipid droplet area relative to nucleocapsid-positive area per cell in infected differentiated adipocytes. Pearson correlation coefficient is indicated as r. Data are shown as the mean ± s.e.m. Source data are available online for this figure.

© The Author(s)

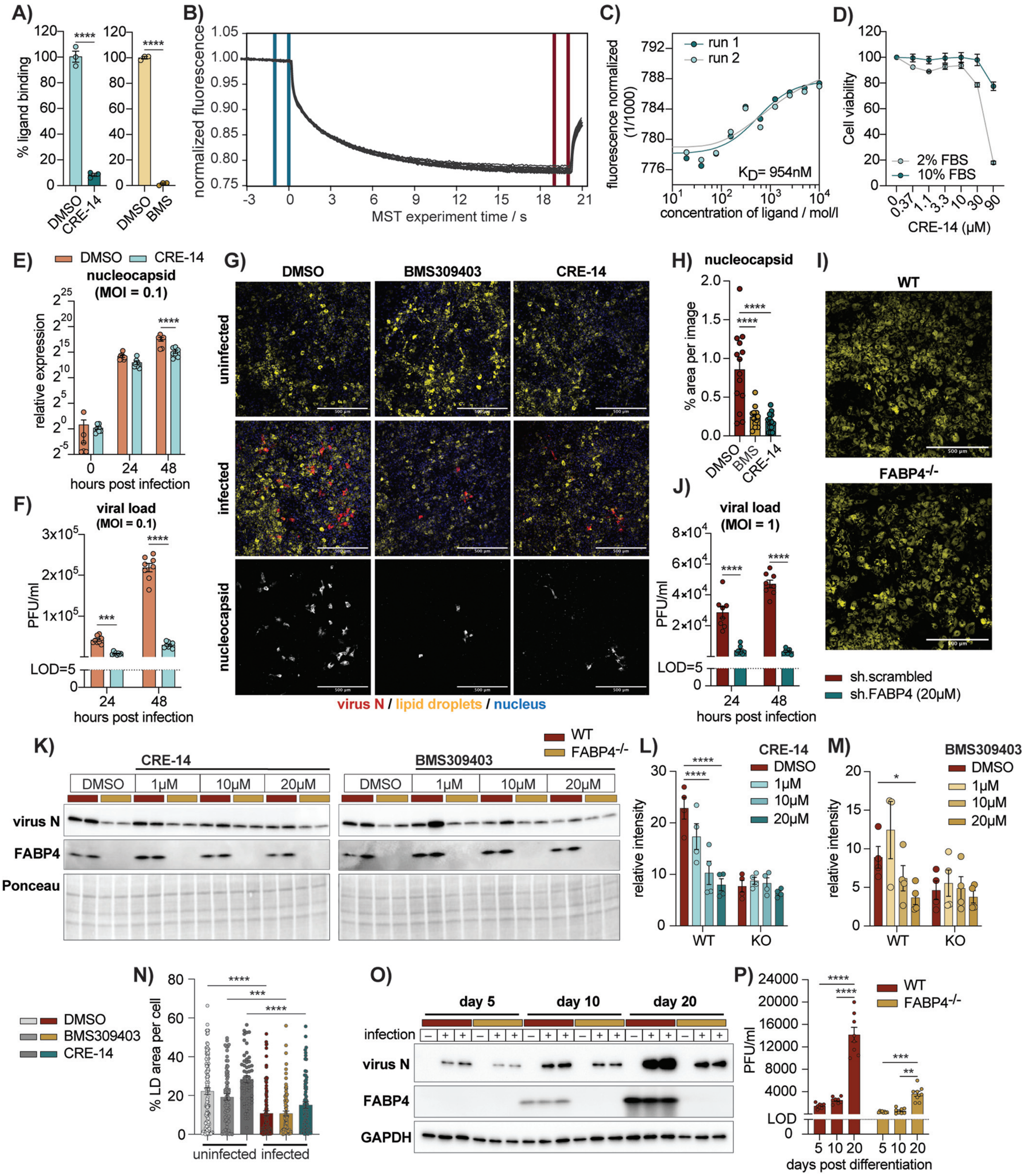

A) B) C) D)
% ligand binding
DMSO CRE-14 DMSO BMS
normalized fluorescence
MST experiment time / s
fluorescence normalized (1/1000)
run 1
run 2
KD= 954nM
concentration of ligand / mol/l
Cell viability
2% FBS
10% FBS
CRE-14 (μM)
E) DMSO CRE-14
nucleocapsid (MOI = 0.1)
relative expression
hours post infection
F) viral load (MOI = 0.1)
PFU/ml
LOD=5
hours post infection
G) DMSO BMS309403 CRE-14
uninfected
infected
nucleocapsid
virus N / lipid droplets / nucleus
H) nucleocapsid
% area per image
DMSO BMS CRE-14
I) WT
FABP4-/-
J) viral load (MOI = 1)
PFU/ml
LOD=5
hours post infection
sh.scrambled
sh.FABP4 (20μM)
K) CRE-14 BMS309403
WT
FABP4-/-
DMSO 1μM 10μM 20μM
virus N
FABP4
Ponceau
L) CRE-14 DMSO 1μM 10μM 20μM
relative intensity
WT KO
M) BMS309403 DMSO 1μM 10μM 20μM
relative intensity
WT KO
N) DMSO BMS309403 CRE-14
% LD area per cell
uninfected infected
O) day 5 day 10 day 20
infection
virus N
FABP4
GAPDH
P) WT FABP4-/-
PFU/ml
LOD
days post differentiation

 © The Author(s)

◀ **Figure EV3. FABP4 deficiency reduces virus titers and cell death following coronavirus infection.**

(**A**) Percentage of FABP4 bound with fatty acid (BODIPY FL C12) in the presence or absence of CRE-14 or BMS309403 ($n = 3$, technical replicates, ****$p < 0.0001$). (**B**) Representative MST time traces with blue and red regions indicating F_{cold} and F_{hot}, respectively, from which fluorescence measurements were normalized. (**C**) Dose-response curve showing FABP4 binding to increasing concentrations of CRE-14, represented as normalized fluorescence. KD value (954 nM) represents the average across two technical runs. (**D**) MRC5 cell viability following administration of titrated doses of CRE-14 at the indicated concentrations of FBS. (**E**, **F**) Differentiated adipocytes infected with SARS-CoV-2 (WA1/2020, MOI = 0.1) and treated with either CRE-14 (20 µM) or DMSO. (**E**) Relative RNA expression of nucleocapsid normalized to β-actin (****$p < 0.0001$). (**F**) Viral load measured from the supernatant using plaque assay (****$p < 0.0001$, ***$p = 0.0004$). (**G**) Representative confocal images of control and infected adipocytes (MOI = 1), fixed 48 h post-infection, stained for virus nucleocapsid (red), lipid droplets (yellow), and nuclei (DAPI, blue) ($n = 3$, biological replicates). Scale bar = 500 µm. (**H**) Percentage of nucleocapsid-positive area per image, averaging 4–5 images per sample (****$p < 0.0001$). (**I**) Representative confocal images showing lipid droplet content (yellow) in WT and FABP4-deficient adipocytes. (**J**) *FABP4*-shRNA knockdown and scrambled controls infected with SARS-CoV-2 (WA1/2020, MOI = 1), with viral titers measured by plaque assay from supernatants. Data are pooled from two independent experiments (n = 8, biological replicates, ****$p < 0.0001$). Statistical analysis was performed using two-way ANOVA. (**K–M**) WT and FABP4-deficient differentiated adipocytes infected and treated with either DMSO, CRE-14, or BMS309403 at indicated doses, with cell lysates collected 48 h post-infection. Data are representative of two independent experiments ($n = 3$). (**K**) Western blots showing nucleocapsid, FABP4, and total proteins (Ponceau S staining) in cell lysates. (**L**, **M**) Quantifications of nucleocapsid band intensity relative to total protein. Statistical analysis was performed using two-way ANOVA (****$p < 0.0001$, *$p = 0.017$). (**N**) Percentage of lipid droplet area per cell in uninfected and SARS-CoV-2-infected adipocytes with or without FABP4 inhibitor treatment (20 µM, ****$p < 0.0001$, ***$p = 0.0006$). (**O**, **P**) Adipocytes infected at 5, 10, and 20 days post-differentiation (MOI = 1). (**O**) Western blot of nucleocapsid, FABP4, and GAPDH protein levels in cell lysates, and (**P**) viral titers in supernatant measured 48 h post-infection. Data are representative of two independent experiments ($n = 3$, biological replicates, ****$p < 0.0001$, ***$p = 0.0008$, **$p = 0.0016$). Statistical analysis was performed using two-way ANOVA. Data are shown as mean ± s.e.m. Source data are available online for this figure.

© The Author(s)

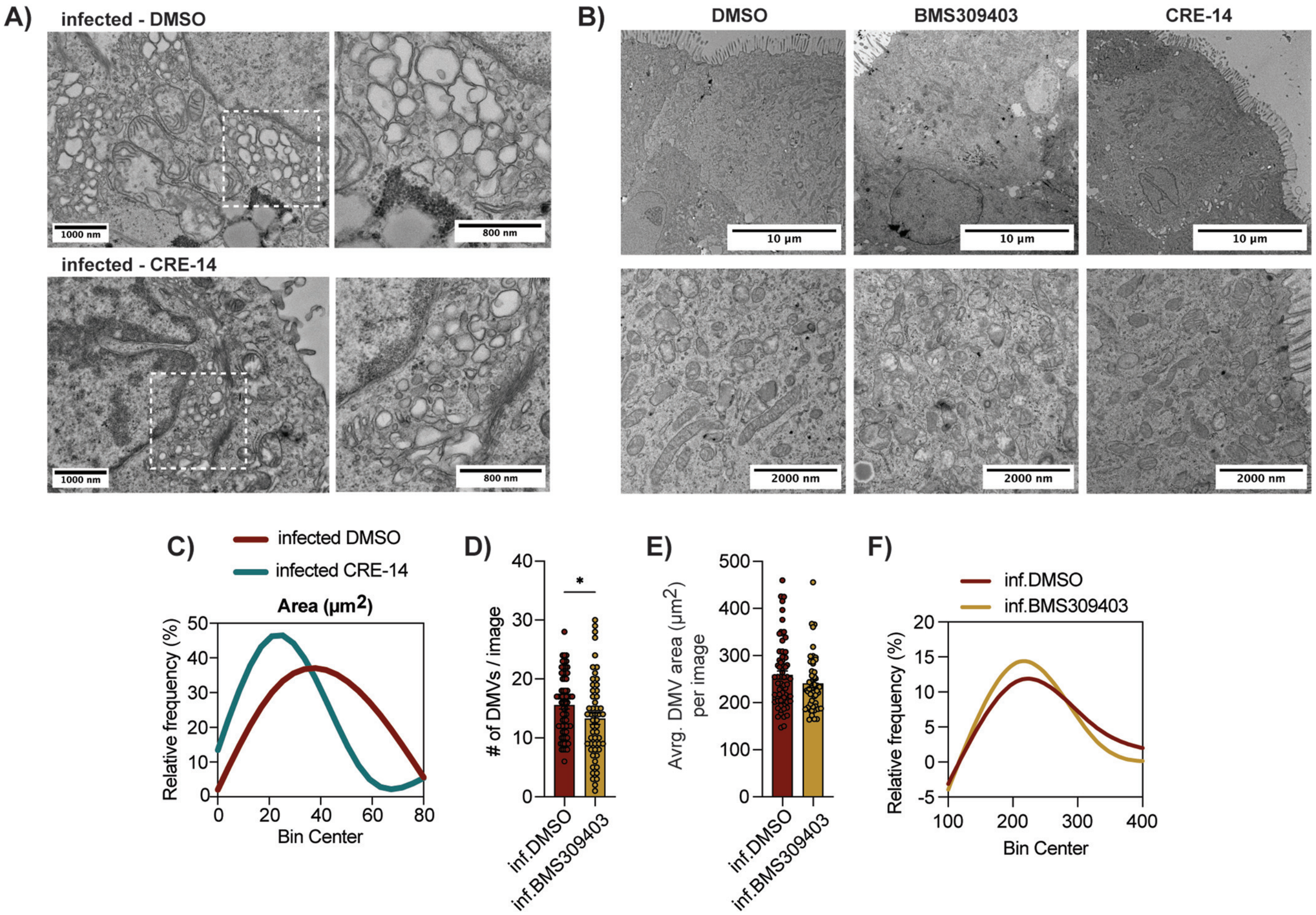

Figure EV4. FABP4 inhibition reduces viral titers across various SARS-CoV-2 variants.

(**A**, **B**) Representative transmission electron microscopy (TEM) images of (**A**) HBE cells 48 h after infection with SARS-CoV-2 (WA1/2020, MOI = 1) and treatment with DMSO or CRE-14 (10 µM) ($n = 3$, biological replicates). (**B**) Uninfected reconstructed airway epithelium 3D culture treated with DMSO, BMS309403, or CRE-14. (**C**) Area of double membrane vesicles in infected HBE cells, determined from TEM images in (**A**). Data displayed as the Fit Spline of the percent frequency distribution. (**D**) Number of DMVs per image ($^*p = 0.0242$), (**E**) average DMV area per image, and (**F**) its frequency distribution quantified from TEM images reconstructed airway epithelium cultures infected with SARS-CoV-2 and treated with DMSO or BMS309403. Statistical analysis is performed using standard t-test ($n = 3$, biological replicates). Data are shown as the mean ± s.e.m. Source data are available online for this figure.

 © The Author(s)

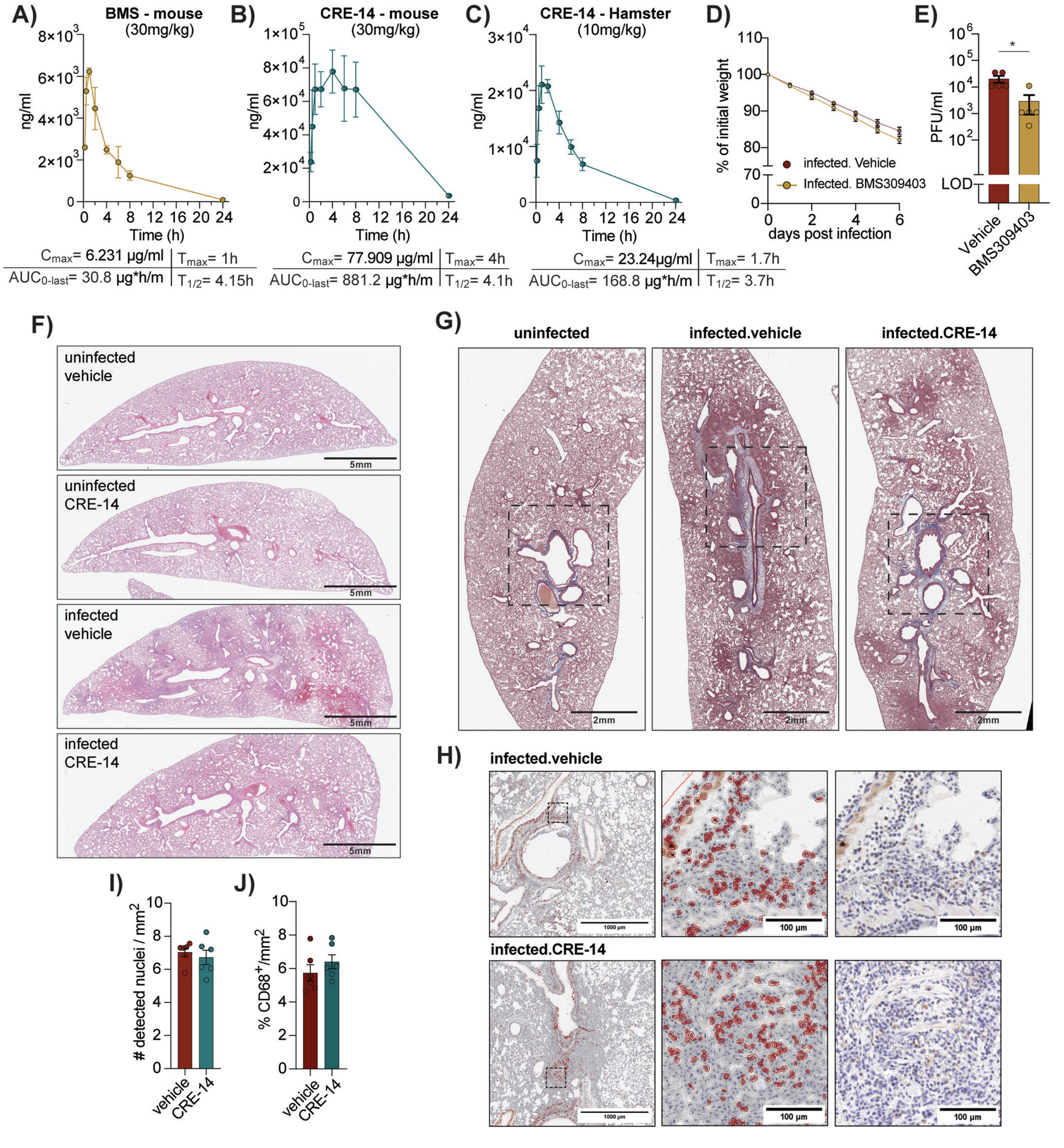

Figure EV5. Pharmacokinetics of FABP4 inhibition in mice and hamsters.

(**A**, **B**) Circulating concentrations of BMS309403 and CRE-14 following a 30 mg/kg subcutaneous injection in C57BL/6J mice. (**C**) Circulating concentrations of CRE-14 in Syrian hamsters following a subcutaneous injection of 10 mg/kg. Tables show (Cmax) maximal concentration, (Tmax) time to reach maximal concentration, (T1/2) half-life, and (AUC0-last) area under the curve from time zero to the last quantifiable time point ($n = 3$, biological replicates). (**D**) Percent body weight over time and (**E**) lung viral titer of hamsters infected with SARS-CoV-2 (Ank1 strain, 100 TCID50) with or without BMS309403 treatment (30 mg/kg) ($n = 5$, biological replicates, $^*p = 0.0215$). Statistical analysis was performed using a standard t-test. (**F**) Representative H&E staining (scale bar = 5 mm) and (**G**) Masson's trichrome staining (scale bar=2 mm) of lung sections from control and infected hamsters with or without CRE-14 treatment ($n = 4$). (**H**) Representative IHC CD68 staining of infected hamster lungs (Ank1-Dlt strain, 1000 TCID50) with or without CRE-14 treatment (15 mg/kg), shown at low (Scale bar = 1 mm) and high (Scale bar = 100 µm) magnification. The mid-panel highlights CD68-positive cells. (**I**) Number of detected cells normalized to lung tissue area per lung and (**J**) percentage of CD68-positive cells quantified from CD68 IHC staining represented in (**H**). (**H–J**: $n = 6$ lungs from 3 biological replicates – hamsters). Data are shown as mean ± s.e.m. Source data are available online for this figure.

© The Author(s)
